# A vertebrate-wide catalogue of T1R receptors reveals diversity in taste perception

**DOI:** 10.1038/s41559-023-02258-8

**Published:** 2023-12-13

**Authors:** Hidenori Nishihara, Yasuka Toda, Tae Kuramoto, Kota Kamohara, Azusa Goto, Kyoko Hoshino, Shinji Okada, Shigehiro Kuraku, Masataka Okabe, Yoshiro Ishimaru

**Affiliations:** 1 Department of Advanced Bioscience, Graduate School of Agriculture, Kindai University, Nara, Japan; 2 School of Life Science and Technology, Tokyo Institute of Technology, Yokohama, Japan; 3 Department of Agricultural Chemistry, School of Agriculture, Meiji University, Kawasaki, Japan; 4 Institute of Innovative Research, Tokyo Institute of Technology, Yokohama, Japan; 5 Graduate School of Agricultural and Life Sciences, The University of Tokyo, Tokyo, Japan; 6 Molecular Life History Laboratory, National Institute of Genetics, Mishima, Japan; 7 Department of Genetics, SOKENDAI (Graduate University for Advanced Studies), Mishima, Japan; 8 Department of Anatomy, The Jikei University School of Medicine, Tokyo, Japan

**Keywords:** Molecular evolution, Evolutionary biology, Evolutionary genetics

## Abstract

Taste is a vital chemical sense for feeding behaviour. In mammals, the umami and sweet taste receptors comprise three members of the taste receptor type 1 (T1R/*TAS1R*) family: T1R1, T1R2 and T1R3. Because their functional homologues exist in teleosts, only three *TAS1R* genes generated by gene duplication are believed to have been inherited from the common ancestor of bony vertebrates. Here, we report five previously uncharacterized *TAS1R* members in vertebrates, *TAS1R4*, *TAS1R5*, *TAS1R*6, *TAS1R7* and *TAS1R8*, based on genome-wide survey of diverse taxa. We show that mammalian and teleost fish *TAS1R2* and *TAS1R3* genes are paralogues. Our phylogenetic analysis suggests that the bony vertebrate ancestor had nine *TAS1R*s resulting from multiple gene duplications. Some *TAS1R*s were lost independently in descendent lineages resulting in retention of only three *TAS1R*s in mammals and teleosts. Combining functional assays and expression analysis of non-teleost fishes we show that the novel T1Rs form heterodimers in taste-receptor cells and recognize a broad range of ligands such as essential amino acids, including branched-chain amino acids, which have not been previously considered as T1R ligands. This study reveals diversity of taste sensations in both modern vertebrates and their ancestors, which might have enabled vertebrates to adapt to diverse habitats on Earth.

## Abstract (web summary)

A combination of phylogenetic analysis and functional assays reveals surprising diversity of taste receptors in the ancestors of vertebrates and their complex evolutionary history.

## Main

Taste is one of the most important senses that govern the feeding behaviour of animals. It is widely accepted that mammals have five basic tastes: umami (savoury), sweet, bitter, salty and sour^1,2^. Taste receptor type 1 (T1R, encoded by *TAS1R*), a G protein-coupled receptor family, consists of three members, namely T1R1, T1R2 and T1R3, which are encoded by the genes *TAS1R1*, *TAS1R2* and *TAS1R3*, respectively, and act as umami or sweet receptors^3,4^. The T1R1/T1R3 heterodimer functions as an umami taste receptor in mammals and detects l-amino acids and 5′-ribonucleotides^5–7^. The mammalian T1R2/T1R3 heterodimer acts as a sweet sensor^6,8^. Likewise, homologues of *TAS1R* family genes have been identified in teleost fishes^9^, and each of the heterodimers T1R1/T1R3 and T1R2/T1R3 can sense several amino acids in teleosts^10^.

A previous phylogenetic analysis revealed that all mammalian and teleost *TAS1R*s can be grouped into the *TAS1R1*, *TAS1R2* and *TAS1R3* clades^11^, suggesting that their common ancestor had only three T1R members derived from gene duplications that have been retained in present-day species. Lineage-specific duplications and losses of *TAS1R* genes have occurred within each of the *TAS1R1*, *TAS1R2* and *TAS1R3* clades, as exemplified by multiple *TAS1R2* genes in zebrafish and fugu, and loss of *TAS1R2* in birds^12^. A few genomic studies of vertebrates such as squamates, coelacanth and sharks have suggested the existence of taxonomically unplaced *TAS1R*s that may not be included in the aforementioned three clades^13–15^. However, the lack of comprehensive characterization and systematic classification has limited our understanding of the evolutionary history of *TAS1R* genes, the functional diversity of T1Rs, and the molecular basis of taste sense in vertebrates.

Here, we present an evolutionary analysis of diverse *TAS1R*s in jawed vertebrates, with an exhaustive taxon sampling encompassing all major ‘fish’ lineages. In addition to clades *TAS1R1*, *TAS1R2* and *TAS1R3*, we identified five novel *TAS1R* clades. The results suggest that the vertebrate ancestor possessed more T1Rs than most modern vertebrates, challenging the paradigm that only three T1R family members have been retained during evolution. Functional analyses suggest that the novel T1Rs have shaped the diversity of taste sense. We propose that the T1R family has undergone an ancient birth-and-death evolution that accelerated their functional differentiation, which may have led to the diversification of feeding habitats among vertebrates.

## Results

### Identification of novel *TAS1R* family members

**Fig. 1:**
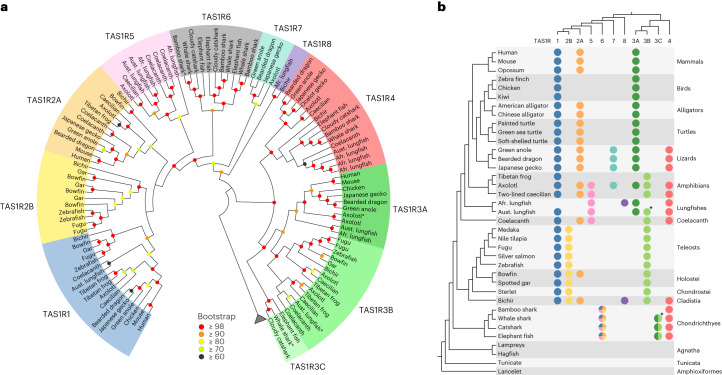
Phylogenetic tree and the revised classification of *TAS1R* members. **a**, Maximum-likelihood tree for amino acid sequences inferred from *TAS1R*s for 21 jawed vertebrates constructed with the JTT + G (CAT approximation) model in RAxML. Coloured circles in each node represent bootstrap values calculated with 1,000 replications, whereas those with low bootstrap support (<60) have no circles. Species classification is represented with coloured highlighting at the tips of the tree. GPRC6A was used as an outgroup (not shown), Afr, African; Aust, Australian. **b**, Distribution of *TAS1R* members among chordates. The colour of circles corresponds to the coloured highlighting in **a** and indicates the presence of *TAS1R* members in the genome assemblies of the various chordates. Phylogenetic relationships among species and among *TAS1R*s are shown on the left and top, respectively. *TAS1R6* of cartilaginous fishes is the orthologue of the *TAS1R1*/*2A*/*2B*/*5* clade and is shown as a circle with assorted colours. Similarly, *TAS1R3C* of cartilaginous fishes is shown with two shades of green that represent *TAS1R3A* and *TAS1R3B*. Circles with asterisks denote putative pseudogenes.

**Extended Data Fig. 1:**
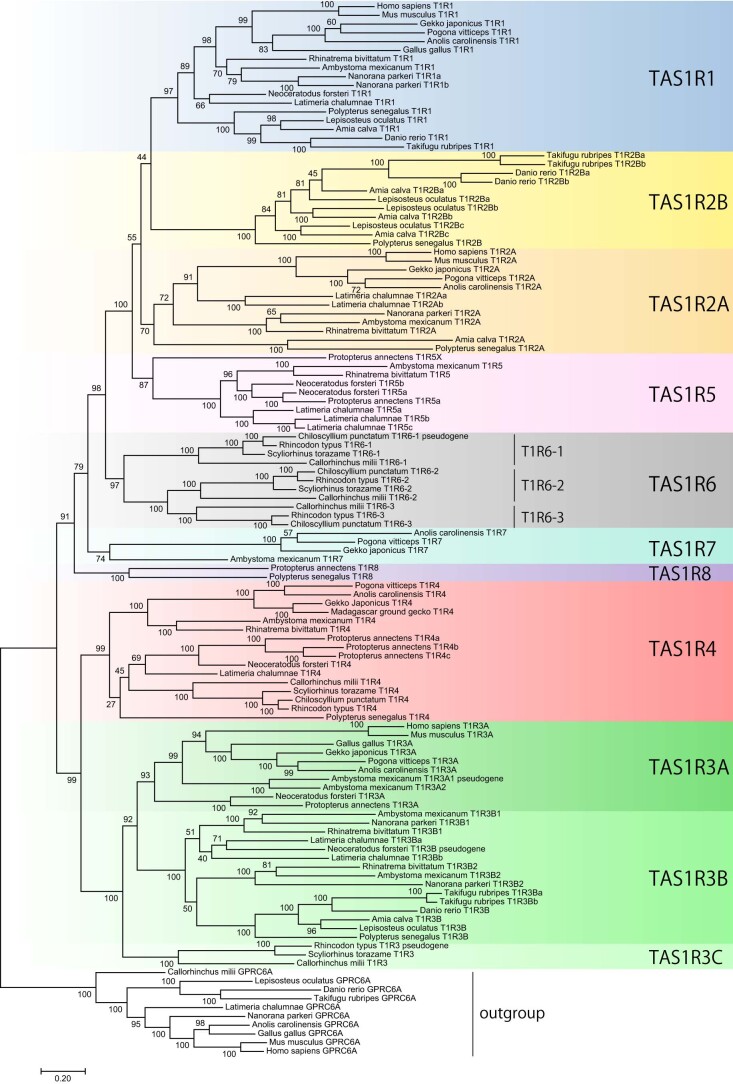
Maximum-likelihood tree of *TAS1R* members identified for 21 vertebrates. A maximum-likelihood tree was constructed from the amino acid sequences encoded by *TAS1R*s using RAxML with the JTT + G (CAT approximation) model. Branch supports represent bootstrap values calculated with 1,000 replications. *TAS1R* clade names are shown on the right.

**Extended Data Fig. 2:**
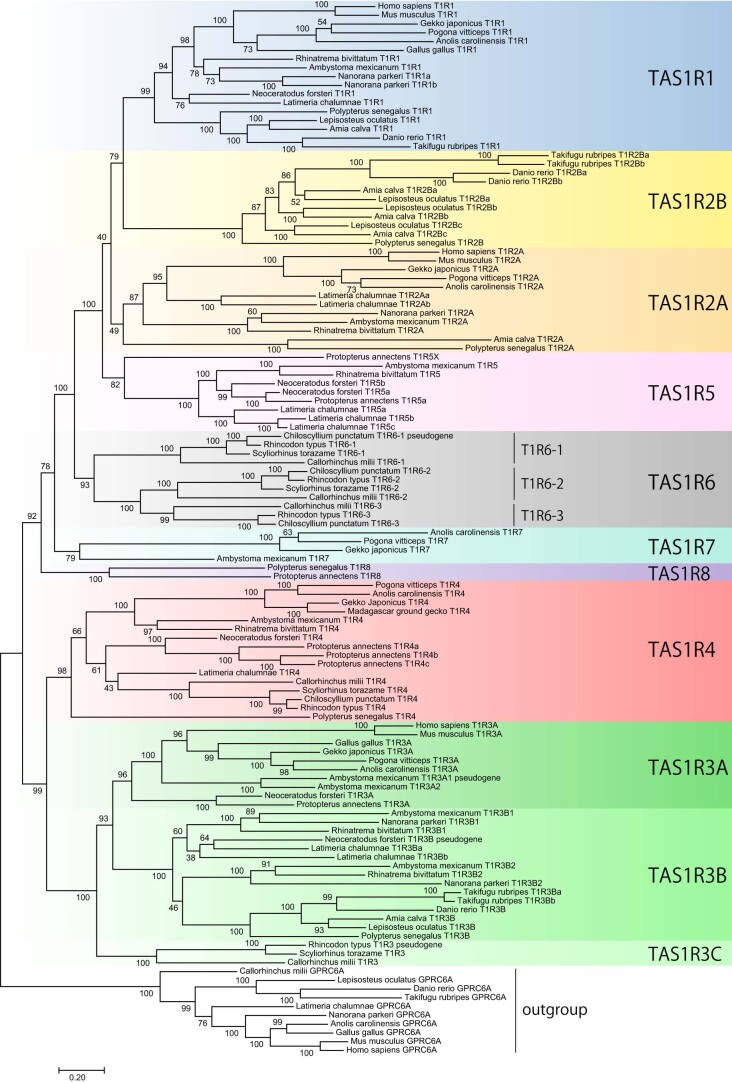
Maximum-likelihood tree with the mixture model for the *TAS1R* members identified for 21 vertebrates. A maximum-likelihood tree was constructed from the amino acid sequences encoded by *TAS1R*s using IQ-tree under the posterior mean site frequency approximation of the JTT + C20 + F + Γ model. Branch supports represent bootstrap values calculated with 1,000 replications. *TAS1R* clade names are shown on the right.

**Extended Data Fig. 3:**
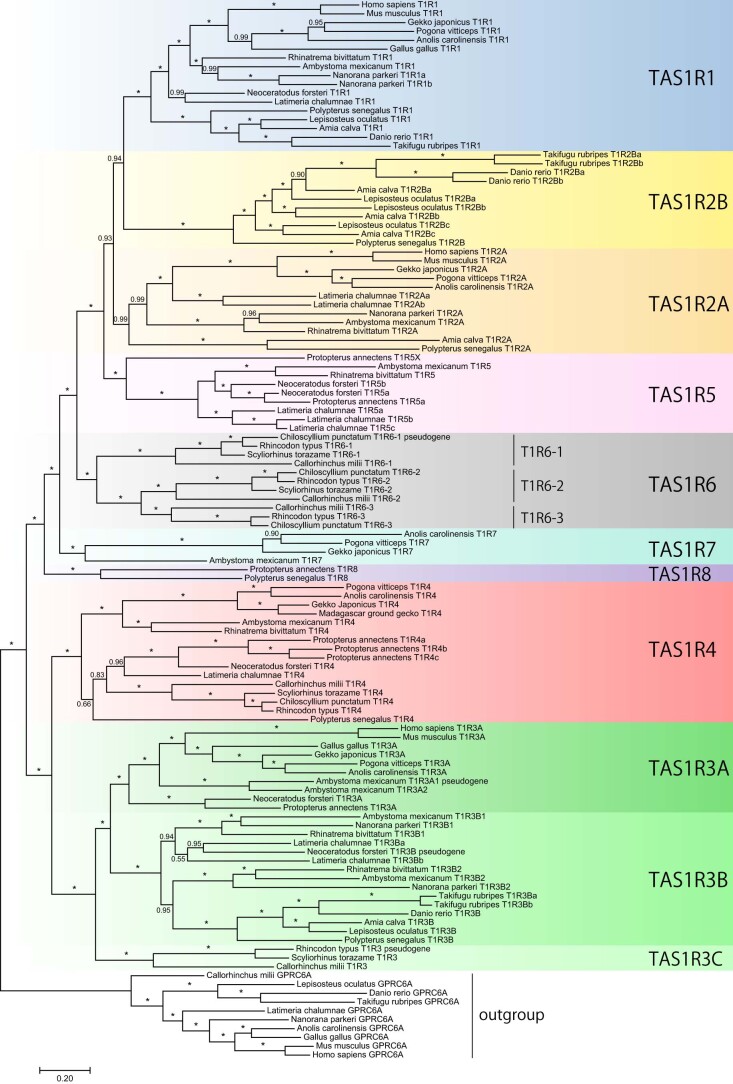
Bayesian tree of *TAS1R* members identified for 21 vertebrates. Bayesian tree inference was performed for the amino acid sequences encoded by *TAS1R*s using MrBayes with the JTT-F + Γ_4_ model. Branch supports represent Bayesian posterior probabilities, and asterisks indicate a posterior probability of 1.00. *TAS1R* clade names are shown on the right.

**Extended Data Fig. 4:**
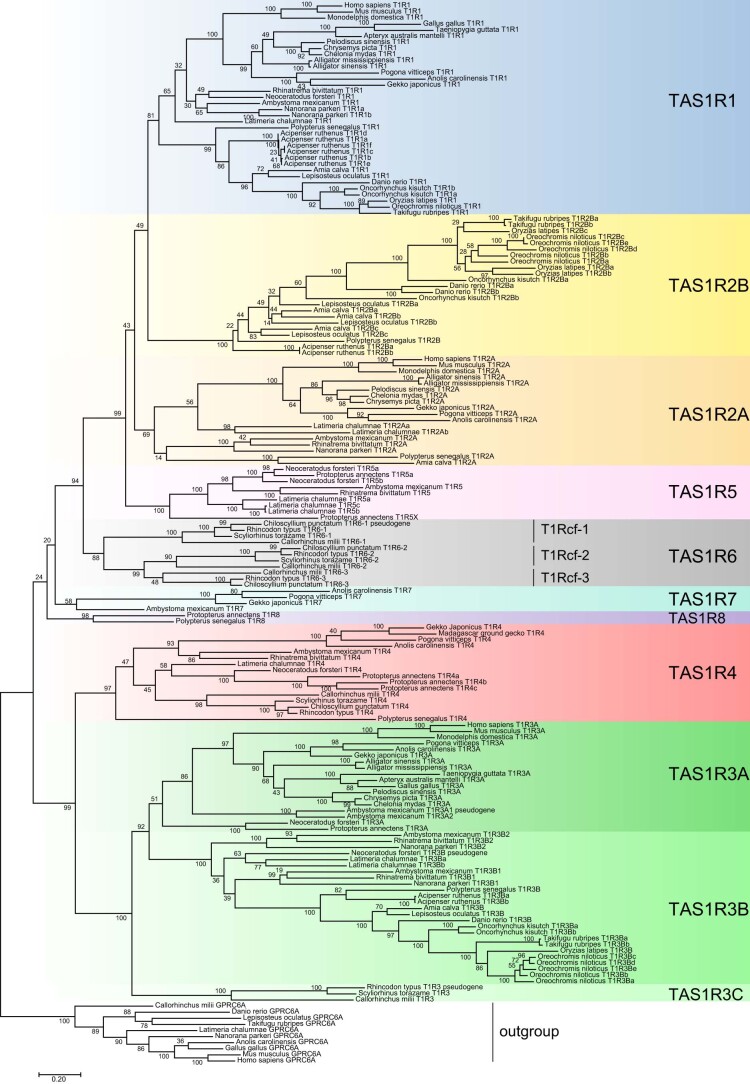
Maximum-likelihood tree for exon 6 sequences of *TAS1R*s identified for 33 vertebrates. A maximum-likelihood tree was constructed from the amino acid sequences of *TAS1R* exon 6 using RAxML with the JTT + G (CAT approximation) model. Branch supports represent bootstrap values calculated with 1,000 replications. *TAS1R* clade names are shown on the right.

We identified homologues of *TAS1R* genes that are included in public genome/transcriptome databases for diverse taxa of jawed vertebrates (Supplementary Table 1). Except for jawed vertebrates, *TAS1R* genes were not identified in any Deuterostomia reference genomes (lampreys, hagfishes, tunicates, lancelets, sea urchins, starfish, hemichordate, etc.) or the nr database, suggesting that the *TAS1R*/T1R family exists only in jawed vertebrates. All phylogenetic trees, as estimated using different methods and datasets, consistently revealed the existence of many *TAS1R*s that had not been categorized into any of the three known clades: *TAS1R1*, *TAS1R2* and *TAS1R3*. These previously undocumented *TAS1R*s were found in lizards, amphibians, lungfishes, coelacanth, bichir and cartilaginous fishes (Fig. 1a and Extended Data Figs. 1–3). The novel *TAS1R*s could be classified into five new clades. One clade, which is the sister clade of *TAS1R3*, was named *TAS1R4* and contains genes from all jawed vertebrates investigated except mammals, birds, crocodilians, turtles, frog, sterlets or neopterygians (Fig. 1b and Extended Data Fig. 4). Another novel *TAS1R*, named *TAS1R5*, exists in axolotl, lungfishes and coelacanth and is close to the clade comprising *TAS1R1* and *TAS1R2* (Fig. 1a).

The sister clade to *TAS1R1* + *TAS1R2* + *TAS1R5*, which was named *TAS1R6*, was identified exclusively in cartilaginous fishes. *TAS1R6* could be further divided into three subclades, namely *TAS1R6-1*, *TAS1R6-2* and *TAS1R6-3*, all of which were found to be present in elephant fish (also called elephant shark), belonging to the taxon Holocephali of cartilaginous fishes (Extended Data Figs. 1–3). Therefore, the three *TAS1R6* subclades probably emerged in the common ancestor of extant cartilaginous fishes. A thorough search of the genomes and transcriptomes of the four cartilaginous fish species identified only *TAS1R3*, *TAS1R4* and *TAS1R6*, but no orthologues of *TAS1R1*, *TAS1R2* or *TAS1R5* (Fig. 1b and Extended Data Fig. 4), suggesting that the *TAS1R1*, *TAS1R2* and *TAS1R5* genes in bony vertebrates are co-orthologues of the *TAS1R6* genes in cartilaginous fish.

**Extended Data Fig. 5:**
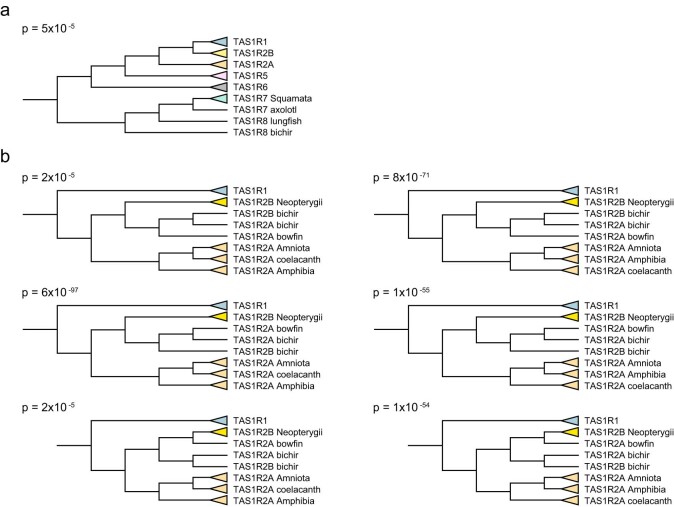
Tree topologies examined for the approximately unbiased test. **a**, Tree topology assuming the grouping of *TAS1R7* and *TAS1R8* and showing a species tree. **b**, Tree topologies for various relationships among *TAS1R2A* and *TAS1R2B* genes from amphibians, coelacanth, and ray-finned fishes. The *p*-values for the approximately unbiased test calculated with CONSEL are shown above each tree. Relationships within each collapsed group were fixed to be the same as in the maximum-likelihood tree (Fig. 1).

Another novel *TAS1R* clade, *TAS1R7*, was found exclusively in axolotl and lizards. Yet another new clade, *TAS1R8*, was identified only in bichir and lungfishes, and its monophyly was robustly supported (Fig. 1 and Extended Data Figs. 1–3), suggesting that *TAS1R8* emerged in the common ancestor of bichir and lungfishes. Indeed, the likelihood of an alternative relationship, in which *TAS1R7* and *TAS1R8* form an exclusive cluster and represent a species tree, was rejected statistically based on the approximately unbiased test (*P* < 10^–4^; Extended Data Fig. 5), suggesting that *TAS1R7* and *TAS1R8* are distinct groups. Among the vertebrates we investigated, the axolotl was found to possess *TAS1R*s from the greatest number (seven) of clades (Fig. 1b and Supplementary Table 2).

### Each of *TAS1R3* and *TAS1R2* consists of two paralogous clades

Remarkably, the phylogenetic analysis also revealed that *TAS1R3* of bony vertebrates could be divided into two clades, named *TAS1R3A* and *TAS1R3B*, with high branch support (Fig. 1 and Extended Data Figs. 1–3). *TAS1R3A* was found to be present in tetrapods and lungfishes but not other vertebrates, whereas *TAS1R3B* was identified only in amphibians, lungfishes, coelacanth and ray-finned fishes. The sister clade to *TAS1R3A* + *TAS1R3B* was identified exclusively in cartilaginous fishes and named *TAS1R3C*. This distribution suggested that an ancestral *TAS1R3* gene was duplicated in the common ancestor of bony vertebrates, with subsequent independent loss of *TAS1R3A* in certain lineages such as coelacanth and ray-finned fishes, whereas *TAS1R3B* was lost in Amniota (mammals and sauropsids). Therefore, the *TAS1R3* genes in mammals and teleost fishes are paralogues. Axolotl and Australian lungfish retained both *TAS1R3A* and *TAS1R3B* although the lungfish *TAS1R3B* has been pseudogenized. Furthermore, the amphibians possess two groups of *TAS1R3B*, named *TAS1R3B1* and *TAS1R3B2* (Fig. 1a), suggesting that *TAS1R3B* was again duplicated—at the latest—before the common ancestor of amphibians.

**Extended Data Fig. 6:**
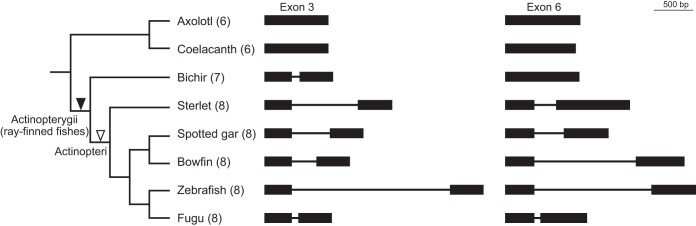
The history of intron acquisitions for the two exons of *TAS1R3B* in ray-finned fishes. Filled boxes represent exons, and lines represent introns. The phylogenetic relationship among *TAS1R3B*-containing species is shown on the left. The closed and open arrowheads indicates the deduced timing (*that is*, the common ancestors of Actinopterygii and Actinopteri) for the acquisitions of introns in exons 3 and 6 of *TAS1R3B*, respectively. The total number of exons of the *TAS1R3B* genes is shown in parentheses.

A distinguishing feature of *TAS1R3B* in ray-finned fishes is the presence of additional introns. In contrast to other *TAS1R*s, which consist of six exons, exon 3 of *TAS1R3B* in ray-finned fishes has been altered during evolution such that it now comprises two exons, suggesting the acquisition of an intron in the common ancestor of ray-finned fishes (Extended Data Fig. 6). Furthermore, exon 6 of *TAS1R3B* in non-bichir ray-finned fishes acquired an additional intron, resulting in a total of eight exons of the gene. Thus, this intron is likely to have been inserted after the divergence of bichir. Except for these two instances, the exon–intron structure is conserved among the *TAS1R* genes we investigated.

Also, *TAS1R2* does not form a single clade in the tree (Fig. 1). The *TAS1R2* genes in ray-finned fishes form a clade with *TAS1R1*, and the other *TAS1R2* group from tetrapods, lungfish, coelacanth, bowfin and bichir forms a sister group to the clade comprising *TAS1R1* and the ray-finned fish *TAS1R2*. The paraphyletic relationship of the two *TAS1R2* groups is concordant with previous reports^13^. Hereafter, we refer to the major vertebrate group as *TAS1R2A* and the ray-finned fish group as *TAS1R2B* (Fig. 1). Notably, we found that the anciently diverged ray-finned fishes such as bowfin and bichir retained both *TAS1R2A* and *TAS1R2B* as well as *TAS1R1*. We assessed the likelihood of other phylogenetic relationships in which *TAS1R2*s have a single origin, and the hypotheses were significantly rejected (*P* < 10^–6^, approximately unbiased test; Extended Data Fig. 5). These results suggested that the *TAS1R2* genes in mammals and teleost fishes are paralogues. Thus, the *TAS1R* phylogenetic tree comprised a total of 11 *TAS1R* clades: *TAS1R1*, *TAS1R2A*, *TAS1R2B*, *TAS1R3A*, *TAS1R3B*, *TAS1R3C*, *TAS1R4*, *TAS1R5*, *TAS1R6*, *TAS1R7* and *TAS1R8*. This unexpected gene diversity challenges conventional conceptions about the evolution of the genetic basis for umami and sweet receptors.

### Birth-and-death evolution of the *TAS1R* family

**Fig. 2:**
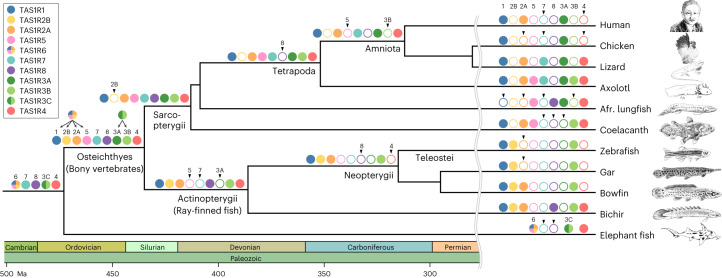
Birth-and-death history of the *TAS1R* family genes during vertebrate evolution. The colour key indicates the names of the various *TAS1R* members. Filled, coloured circles on the branches indicate the presence of *TAS1R* members, whereas open circles indicate their absence, as estimated based on the phylogenetic tree (Fig. 1a) and distribution among vertebrates (Fig. 1a). Arrowheads above open circles indicate that the *TAS1R* member was lost at the branch. Geological periods and ages (Ma) taken from TimeTree^16^ are shown at the bottom. Taxon names are shown below branches. Species-specific gene duplication events for each *TAS1R* were ignored. Illustrations of the species, including humans (Kikunae Ikeda, the discoverer of umami), are shown on the right.

Some of the higher-level relationships among the *TAS1R* clades were supported with relatively high branch support, as exemplified by the exclusive cluster of *TAS1R3* + *TAS1R4*, the clade of the other *TAS1R*s, the clade of *TAS1R1* + *TAS1R2B* + *TAS1R2A* + *TAS1R5*, and the sister relationship of this latter clade to *TAS1R6* (Fig. 1). Based on the phylogenetic relationships and the distribution of all *TAS1R* members (Fig. 1b), the most parsimonious evolutionary scenario could be deduced as follows (Fig. 2). The first *TAS1R* gene emerged in the ancestral lineage of jawed vertebrates during the period 615–473 million years ago (Ma) according to TimeTree^16^. This ancestral *TAS1R* underwent multiple duplications to produce at least five *TAS1R* genes: *TAS1R3C* (the ancestral gene of *TAS1R3A* and *TAS1R3B*), *TAS1R4*, *TAS1R7*, *TAS1R8* and *TAS1R6* (the ancestral gene of *TAS1R1*, *TAS1R2B*, *TAS1R2A* and *TAS1R5*). Owing to speciation between cartilaginous fishes and bony vertebrates ~473 Ma, *TAS1R6* and the ancestral gene of clade *TAS1R1* + *TAS1R2B* + *TAS1R2A* + *TAS1R5* diverged. This speciation probably also led to the split between *TAS1R3C* and clade *TAS1R3A* + *TAS1R3B*. In the stem lineage of bony vertebrates (473–435 Ma), *TAS1R1*, *TAS1R2A*, *TAS1R2B* and *TAS1R5* were generated via additional gene duplication events. Simultaneously, *TAS1R3A* and *TAS1R3B* were generated by gene duplication, resulting in a total of nine *TAS1R*s in the common ancestor of bony vertebrates (Fig. 2). After the divergence of ray-finned and lobe-finned fishes ~435 Ma, a portion of the expanded *TAS1R*s began to be differentially lost during vertebrate evolution. For example, *TAS1R8* was lost in the tetrapod ancestor, *TAS1R3B* and *TAS1R5* were lost in the amniote ancestor, and *TAS1R4* and *TAS1R7* were lost in the mammalian ancestor (Fig. 2). Thus, gene expansion before the common ancestor of bony vertebrates as well as the subsequent loss of a subset of genes have resulted in the rather dispersed distribution of *TAS1R*s in extant species (Fig. 1b).

### *TAS1R* gene cluster revealed by scanning understudied genomes

**Fig. 3:**
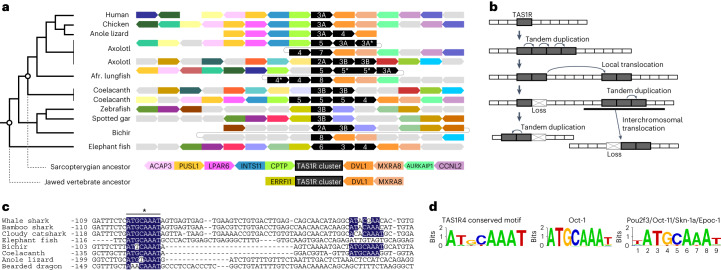
Synteny around *TAS1R*s and conserved Oct-like motifs in the *TAS1R4* upstream regions across vertebrates. **a**, Synteny around each *TAS1R* gene cluster is partly conserved across representative vertebrates. *TAS1R*s are represented by black polygons, and those with asterisks are putative pseudogenes. Coloured polygons indicate genes shared among species, and grey colour represents genes not shared among the species or unknown. The species tree is shown on the left. The deduced gene orders in common ancestors of Sarcopterygii and jawed vertebrates are shown at the bottom. **b**, Proposed model for the expansion of *TAS1R* genes across distant chromosomal regions during evolution. **c**, Conserved motifs located upstream of *TAS1R4*. Sequence alignment of the upstream region of the *TAS1R4* open reading frame revealed two conserved Oct-like transcription-factor binding motifs (blue shading). Numbers represent nucleotide positions from the *TAS1R4* start codon site. The asterisk indicates one of the motifs that significantly resembles the Oct factor binding motif. **d**, Sequence logo for the conserved motif denoted with the asterisk in **c**. Known binding motifs of Oct-1 (retrieved from TRANSFAC) and Oct-11/Pou2f3/Skn-1a/Epoc-1 (retrieved from JASPAR) are compared.

**Extended Data Fig. 7:**
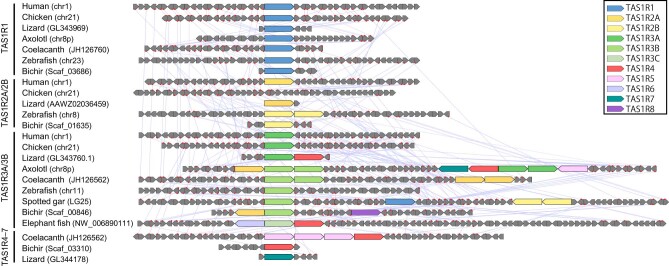
Comparison of synteny among vertebrates. *TAS1R*s and non-*TAS1R* genes are represented by colored and grey polygons, respectively, with each pointed end indicating the direction of transcription. Genomic regions are categorized according to the *TAS1R1*-, *TAS1R2*-, *TAS1R3*-containing regions as well as the other *TAS1R*-containing regions. Orthologous non-*TAS1R* genes between the closely represented species are connected by light-blue lines.

The simplest model for gene amplification is a tandem duplication that produces multiple genes located side-by-side^17,18^. However, *TAS1R1*, *TAS1R2* and *TAS1R3* are located far from each other in both mammalian and teleost genomes. In human chromosome 1, for example, *TAS1R1* is 12 Mb distant from *TAS1R2A* and 5 Mb distant from *TAS1R3A*, with many intervening genes in each case. In zebrafish, each of *TAS1R1* and *TAS1R3B* is located on a different chromosome from the two copies of *TAS1R2B*, prompting us to hypothesize that *TAS1R* members may have undergone expansion by tandem duplications in the ancestral genome, followed by subsequent translocation to distant regions during evolution. To address this possibility, the synteny of *TAS1R3* and *TAS1R4* was investigated among vertebrates, particularly those having the novel *TAS1R*s (Fig. 3 and Extended Data Fig. 7). Indeed, the novel *TAS1R*s were found to be located side-by-side in anole lizard, axolotl, lungfish, coelacanth and elephant fish (Fig. 3a). Even *TAS1R2A* and *TAS1R3B* are located next to each other in axolotl and bichir. This result suggested that a *TAS1R* gene cluster had formed in the common ancestor of jawed vertebrates.

A comparison of neighbouring genes revealed that the *TAS1R* cluster is flanked by two genes, namely *DVL1* and *MXRA8*, in the genomes of human, chicken, axolotl, lungfish, coelacanth, bichir and elephant fish (Fig. 3a), suggesting that these two genes were adjacent to the *TAS1R* cluster in the common ancestor of jawed vertebrates. On the opposite end of the *TAS1R* cluster, the gene order of *ACAP3*–*PUSl1*–*LPAR6*–*INTS11*–*CPTP* may have been established in the sarcopterygian ancestor based on conservation among coelacanth, axolotl, chicken and partly in lizard. Furthermore, the presence of other *TAS1R*-proximal genes is also conserved even across distant chromosomal regions (Extended Data Fig. 7). This suggested that a chromosomal region containing both *TAS1R* and multiple neighbouring genes—rather than the *TAS1R* gene alone—had translocated to a different region in each lineage. Based on the inferred ancestral gene order, the unique distribution of *TAS1R*s among present-day mammals and teleost fishes may have been a consequence of a combination of several events (Fig. 3b): (1) tandem duplication that produced a *TAS1R* cluster in the ancestor of jawed vertebrates; (2) local translocation of a subset of *TAS1R*s within a chromosome, as seen in multiple clusters observed in axolotl and coelacanth (Extended Data Fig. 7); (3) translocation of entire *TAS1R*-containing regions to different chromosomes, as observed in zebrafish; and (4) gene loss(es) in each lineage, as partly observed as the presence of pseudogenes (Fig. 1a). Moreover, lineage-specific duplication events have occurred such as *TAS1R2B* in zebrafish and fugu and *TAS1R2A* in coelacanth (Fig. 1a and Extended Data Fig. 7)^12,13^. Finally, we found that some of the *TAS1R*s identified have been pseudogenized; for example, the whale shark *TAS1R3C* and the lungfish *TAS1R3B* (Fig. 1). These observations also support the evolutionary model of the *TAS1R* family presented in Fig. 3b.

### Conservation of a possible Oct-binding site in *TAS1R4*

Because *TAS1R4* is shared among a wide variety of vertebrates in contrast to the other novel *TAS1R*s, we expected that a transcriptional regulatory mechanism might be conserved among the species. To explore the existence of a possible regulatory element, sequences upstream of the open reading frames of *TAS1R4* from various species were aligned, and MEME^19^ was used to search for transcription-factor binding motifs conserved among the species. The most significant hit was the binding motif for the Oct family (*P* < 10^–12^ for Oct-4, *P* < 10^–7^ for Oct-1). At least one sequence of the known Oct-binding motif ‘ATGCAAAT’ is conserved among cartilaginous fishes, coelacanth, bichir and lizards in the region upstream of *TAS1R4* (Fig. 3c,d). Although little is known about the transcriptional regulatory network in taste-receptor cells (TRCs), one known transcription factor responsible for TRC differentiation is Skn-1a, which is an Oct factor also known as Oct-11, Epoc-1 or Pou2f3 (ref. ^20^). In mammals, Skn-1a is exclusively expressed in umami, sweet and bitter TRCs, and loss of Skn-1a results in the complete absence of these TRCs^20,21^. This finding suggested that *TAS1R4* expression is governed by a conserved regulatory mechanism involving an Oct transcription factor, possibly Skn-1a. Although Oct-binding sites were not observed in the other novel *TAS1R*s, these findings may help to elucidate the molecular mechanisms underlying the conserved and/or lineage-specific expression of a variety of *TAS1R*s in TRCs, which will enhance our understanding of the evolutionary origin of TRCs.

### T1R diversity expands the range of taste sensation

**Fig. 4:**
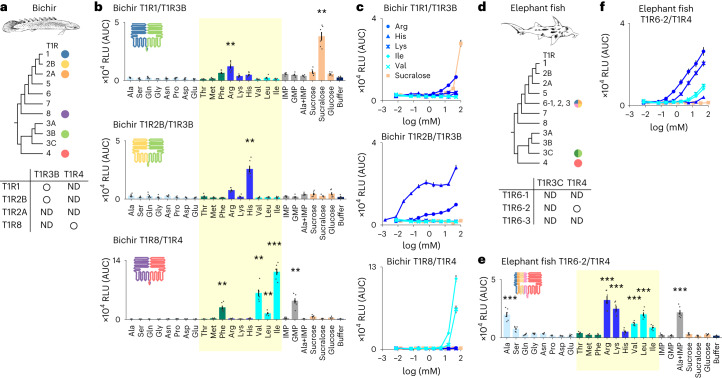
Functional analysis of T1Rs from bichir and elephant fish. **a**, T1R repertoire in bichir and their combinations used for the functional analysis. ND, not detected for any ligands tested. **b**, Responses of three combinations of T1R1/T1R3B (upper), T1R2B/T1R3B (middle) and T1R8/T1R4 (lower) to each of 17 amino acids (50 mM), nucleic acids (10 mM), sugars and sucralose (100 mM). Values represent the mean ± s.e.m. of six independent experiments performed with duplicate samples. **, >10,000 RLU with *q* < 0.01; ***, >10,000 RLU with *q* < 0.001 by one-sided *t*-test (T1R1/T1R3B Arg, *P* = 0.0012; sucralose, *P* = 0.000094; T1R2B/T1R3B His, *P* = 0.00015; T1R8/T1R4 Phe, *P* = 0.00063; Val, *P* = 0.00030; Leu, *P* = 0.00057; Ile, *P* = 0.000013; GMP, *P* = 0.00047). Amino acids that are essential in fishes are highlighted in yellow. AUC, area under the curve. **c**, Dose–response curves for T1R1/T1R3B (upper), T1R2B/T1R3B (middle) and T1R8/T1R4 (lower) to three basic amino acids (Arg, His and Lys; blue), two BCAAs (Ile and Val; light blue) and an artificial sweetener (sucralose; orange). Values represent the mean ± s.e.m. of six independent experiments performed with duplicate samples. **d**–**f**, Same as **a**–**c**, respectively, for elephant fish and the functional analysis of T1R6-2/T1R4 (Ala, *P* = 0.00015; Arg, *P* = 0.00013; Lys, *P* = 0.000094; Val, *P* = 0.000011; Leu, *P* = 0.000045; Ala + IMP, *P* = 0.000069).

**Extended Data Fig. 8:**
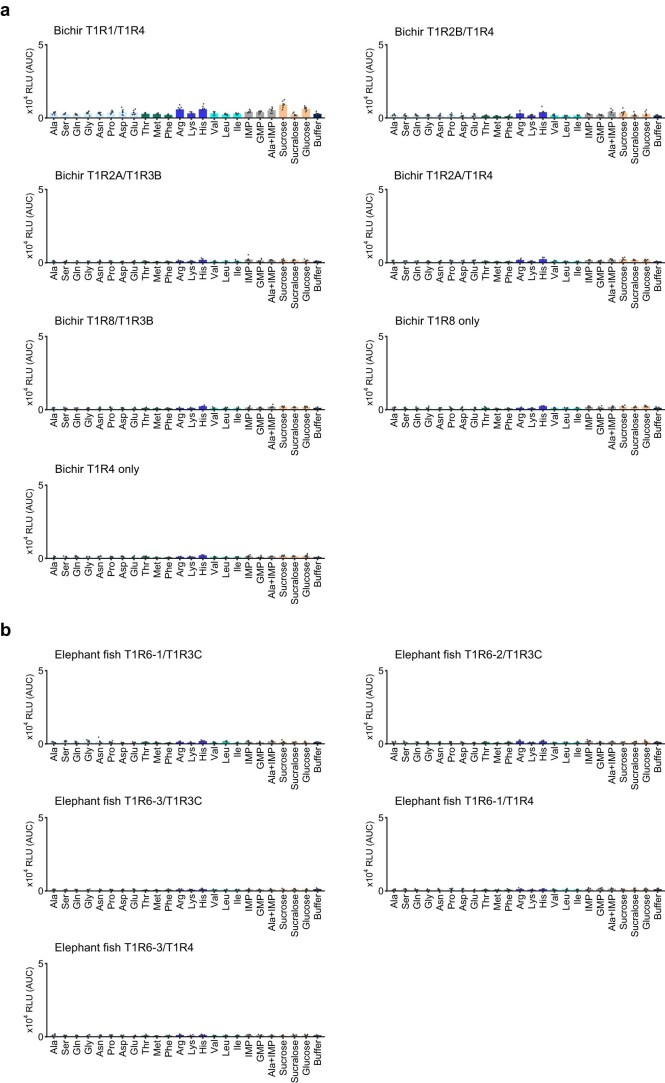
No significant response of various combinations of T1Rs. **a**, Five T1R combinations, T1R4-only, and T1R8-only from the bichir were coexpressed in HEK293T cells, and their responses to each of the 17 amino acids (50 mM), nucleic acids (10 mM), sugars and sucralose (100 mM) were tested. Values represent the mean ± standard error of six independent experiments performed with duplicate samples. **b**, Same as (**a**) except using five T1R combinations from the elephant fish. A lack of response may be due to technical issues with the heterologous expression system, or there is a possibility that they were functional and could respond to ligands not used in this study.

Because receptor responses cannot be predicted from sequence analysis alone, functional tests using cultured cells heterologously expressing the target receptor are useful. We previously established a high-throughput screening system for the T1R receptors using a luminescence-based assay^22^ and have used it to identify ligands for both mammalian^7,23^ and non-mammalian^24–26^ T1R receptors. To examine which T1R receptors can form heterodimers and which ligands they respond to, we performed the functional analysis for the T1Rs of bichir, which possesses two newly discovered T1R groups (T1R4 and T1R8) and four known T1R groups (T1R1, T1R2A, T1R2B and T1R3B). Because *TAS1R4* is the sister clade of *TAS1R3* and is present in all vertebrates that harbour the other novel *TAS1R*s (Fig. 1b), T1R4 could be assumed to form a heterodimer with another T1R. We combined either T1R3B or T1R4 with another T1R (T1R1, T1R2A, T1R2B or T1R8) in the functional analysis (Fig. 4a). Among these receptor pairs, strong responses to amino acids were detected for T1R1/T1R3B, T1R2B/T1R3B and T1R8/ T1R4 (Fig. 4b and Extended Data Fig. 8). For bichir T1R2A, its combination with T1R3B or T1R4 did not yield a response to any of the tastants examined (Extended Data Fig. 8a). Responses were not observed when T1R4 or T1R8 alone was used (Extended Data Fig. 8a), suggesting that these newly discovered T1Rs function as obligate heterodimers in bichir.

The bichir T1R8/T1R4 responded strongly to Phe and to branched-chain amino acids (BCAA; Ile, Val and Leu), whereas T1R1/T1R3B and T1R2B/T1R3B responded strongly to basic amino acids (Arg and His) (Fig. 4b,c). Fishes have 12 nutritionally essential amino acids (Cys, His, Ile, Leu, Lys, Met, Phe, Arg, Thr, Trp, Tyr and Val)^27^, 9 of which are included in the 17 amino acids that were tested in the T1R functional analysis. Notably, all six amino acids to which the bichir T1Rs responded are essential amino acids (*P* < 0.05; one-sided Fisher’s exact test), suggesting that the bichir T1Rs may sense essential amino acids in foods by taking advantage of the ability to perceive BCAA via the T1R4-related receptor.

Bichir T1R1/T1R3B also responded to sucralose, a structural analogue of sucrose. Although only T1R2A/T1R3A is responsible for sugar perception in mammals and lizards^26^, we previously demonstrated that T1R1/T1R3A of birds has gained the ability to detect sugars^24,25^. Also, T1R2B/T1R3B of two teleost fishes, namely carp^28^ and gilthead seabream^29^, can detect sugars at high concentrations (100–200 mM). Our assay was unable to analyse sugars at concentrations greater than 100 mM because of non-specific responses caused by changes in osmolarity. Although the sucrose response at 100 mM was not significantly higher than the thresholds we set in this study (>10,000 relative light units (RLU) with a false discovery rate (*q*) of <0.01), combined with the fact that its structural analogue, sucralose, could elicit a clear response, higher concentrations of sucrose may be able to activate bichir T1R1/T1R3B. In addition, we found that bichir T1R8/T1R4 could respond to GMP, although a previous study reported that neither T1R1/T1R3B nor T1R2B/T1R3B of medaka fish nor T1R2B/T1R3B of zebrafish could be activated by 5′-ribonucleotides^10^. Therefore, the origin and evolution of sugar and nucleotide taste perception may need to be reconsidered based on results from future genetic and functional analyses of T1Rs.

We also performed a functional analysis of elephant fish T1Rs. Three genes of the T1R6 clade, namely T1R6-1, T1R6-2 and T1R6-3, were tested in combination with T1R3C and T1R4, and only the response of the T1R6-2/T1R4 pair could be detected (Fig. 4d–f and Extended Data Fig. 8b). This combination responded to a relatively broad range of amino acids, including both BCAA (Val, Leu) and basic amino acids (Arg, Lys). The T1Rs of mammals and teleosts have little or no response to BCAA but can respond to basic amino acids^5,10,23^. The observed strong response of bichir T1R8/T1R4 and elephant fish T1R6-2/T1R4 to BCAA may reflect functional characteristics of the novel T1Rs involving T1R4 and possibly that of ancient T1Rs in the vertebrate ancestor.

### Expression of the novel T1Rs in TRCs

**Fig. 5:**
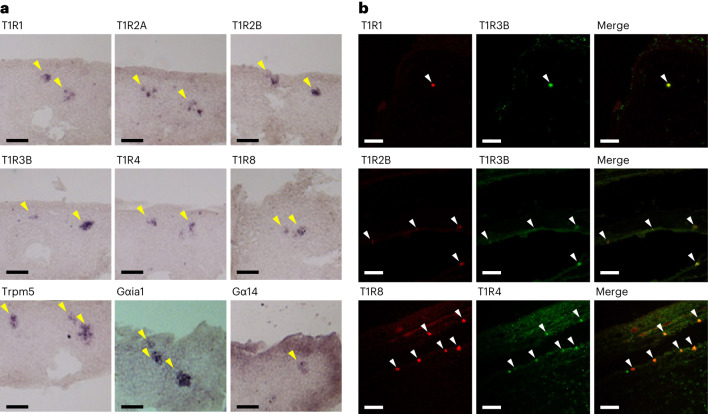
In situ hybridization of T1Rs in TRCs of bichir. **a**, Expression of six T1Rs and three marker genes in sagittal sections of the lips. Yellow arrowheads indicate TRCs that expressed the various genes. Scale bar, 50 μm. The experiments were repeated at least three times. **b**, Double-label fluorescence in situ hybridization for the combinations of T1R1/T1R3B (upper), T1R2B/T1R3B (middle) and T1R8/T1R4 (lower) in the sections. White arrowheads indicate co-expressing cells. Scale bar, 50 μm. The experiments were repeated at least twice.

To investigate whether the novel T1Rs are indeed expressed in TRCs, we performed in situ hybridization with sections of the lips and gill rakers of bichir (Fig. 5a). T1R1, T1R2A, T1R2B, T1R3B, T1R4 and T1R8 were expressed in subsets of TRCs. Genes encoding downstream signal-transduction molecules, such as TRPM5, Gαia1 and Gα14, were also highly expressed in subsets of TRCs in the lips and gill rakers. The signal frequencies for TRPM5, Gαia1 and Gα14 were higher than those for T1Rs.

To examine the localization of T1Rs in TRCs, we next performed double-label fluorescence in situ hybridization. This analysis confirmed the overlap of the signal for T1R1 with that of T1R3B, T1R2B with T1R3B and T1R8 with T1R4 (Fig. 5b). These results suggested that T1R1/T1R3B, T1R2B/T1R3B and T1R8/T1R4 function as heterodimers, in accordance with the results of our functional assays.

## Discussion

The complex evolutionary history of the T1R/TAS1R family includes ancient gene expansions followed by independent lineage-specific losses, which contrasts with conventional wisdom that essentially only three members were retained during evolution^11,30^. The evolution of certain other chemoreceptors, such as the T2R (or TAS2R) bitter-taste receptor family and olfactory receptors, followed a birth-and-death process^31^. In this mode of evolution, tens or hundreds of the receptor family/superfamily genes have undergone extensive lineage-specific duplication followed by frequent gene loss by deletion/inactivation^30^. Our results suggest that a similar process—although less extensive than what occurred for other chemoreceptors—contributed to the phylogenetic and functional expansion of the T1R family early during vertebrate evolution. *TAS1R*s were not subjected to extensive birth-and-death evolution, possibly because T1R ligands are limited to amino acids, sugars and nucleotides in contrast to T2Rs and olfactory receptors that respond to a wider range of ligands/stimulants. In line with our discovery, many chemoreceptors, including *TAS1R*s in teleost fishes, have recently been reported to have undergone dynamic evolution including lineage-specific expansion and gene losses^32^. It is also possible that the ancient expansion might have contributed to an alternate use of T1Rs in tissues other than the sensory organs because certain G protein-coupled receptors (including T1Rs) are expressed in the gut of mammals and teleost fishes^33,34^ although their functions remain unresolved.

The functional combinations of the bichir T1R8/T1R4 and the elephant fish T1R6-2/T1R4 suggest that T1R4 may have a similar role to T1R3 by forming a functional heterodimer with another novel T1R such as T1R5, T1R6, T1R7 or T1R8. This model is supported by the fact that species with either *TAS1R5*, *TAS1R6*, *TAS1R7* or *TAS1R8* also have *TAS1R4* (Fig. 1b) and that *TAS1R4* is phylogenetically the sister group of *TAS1R3* (Fig. 1a). Therefore, the common ancestor of bony vertebrates, which had at least nine T1Rs, probably had two types of heterodimeric T1R receptors, namely T1R3- and T1R4-dependent receptors. This relatively wide variety of possible T1R combinations involving two duplicated genes of T1R2 (A and B) and T1R3 (A and B) might have contributed to the diversification of taste sensation.

Our findings provoke new questions, one of which is why many *TAS1R* genes—particularly the T1R4-related receptors—have become unnecessary in each lineage independently, and many species have come to rely predominantly on T1R3-dependent receptors (Fig. 2). One possible explanation is that dietary changes could have rendered one or more T1Rs unnecessary, and therefore, gene loss might have had little or no effect on survival. This is plausible because previous studies reported losses of *TAS1R*s and *TAS2R*s in many land vertebrates, presumably in association with specific dietary shifts^32,35–37^. Also, the behaviour of swallowing foods whole, without mastication, could have diminished the essentiality of taste sense in certain vertebrates, as previously discussed with respect to mammals^36,38^ and squamates^39^. Alternatively, it is possible that T1R3-dependent receptors have acquired greater functional flexibility and/or evolvability than other T1Rs; various tastants might have been detected via the evolutionary tuning of sequences and structures of the T1R3-dependent receptors rather than additional gene duplication. Such cases are indeed known for land vertebrates such as primates^7^ and birds^24,25^. To address these issues, it will be essential to carry out functional analyses of the newly discovered T1Rs in addition to the known T1R1/T1R3 and T1R2/T1R3 for a broad range of vertebrates, as our current results demonstrate. For example, the response to BCAA is a previously unreported characteristic shared between the bichir T1R8/T1R4 and elephant fish T1R6-2/T1R4 (Fig. 4). This type of result provides insight into the sensory characteristics of an ancestor of vertebrates. We also found that bichir T1Rs responded to other essential amino acids, a sucrose analogue and a nucleotide. Future analysis will resolve whether the functions indeed reflect the characteristics of the ancestral species.

Thus, by demonstrating the unexpected diversity and unique evolutionary process of the T1R family, our results set the stage for understanding the evolutionary-scale changes in taste sense in vertebrates. The remarkably broad range of tastants detected by the T1Rs reflects the latent diversity of taste senses in vertebrates, and this may explain their successful expansion across diverse feeding habitats on Earth. Our understanding of taste sense will be further enhanced by clarifying T1R repertoires in each species, their tissue-specific expression, transcriptional regulatory mechanisms and protein structures. Revealing the functional and structural diversity of the novel T1Rs will also help us elucidate the molecular mechanisms by which human T1Rs recognize palatable tastes.

## Methods

### Identification of *TAS1R* genes from genome and RNA sequencing data

We used genome and transcriptome data as well as related raw sequence reads for a broad range of vertebrates (Supplementary Table 1). First, a tblastn search was conducted against the 33 genomes using amino acid sequences of exon 6 of the *TAS1R*s of human, chicken and zebrafish as queries. Hit sequences meeting the E value threshold of 1,110^–40^ were used to construct a phylogenetic tree using RAxML v.8.2.12 with the JTT + G (CAT approximation) model. The G protein-coupled receptor family C group 6 member A (GPRC6A) genes, which are the closest relative of T1Rs^40^, were used as the outgroup. Identified valid *TAS1R* sequences were used for subsequent iterations of the tblastn search. RNA sequencing data were assembled using Bridger v.r2014-12-01 with default parameters and were used as a database for the tblastn search^41^.

We also conducted a NCBI tblastn search against all reference genomes of Deuterostomia excluding jawed vertebrates (Gnathostomata), and did not find any *TAS1R* orthologues. In addition, an NCBI blastp search against the nr database, excluding Gnathostomata, yielded no hits for *TAS1R* orthologues. Subsequently, we performed comprehensive annotation of *TAS1R* exons in 21 organisms, including model organisms and species that were presumed to possess novel/unclassified *TAS1R* members, as identified via the procedure above.

The exon regions were predicted using AUGUSTUS v.3.2.3 (ref. ^42^). followed by an evaluation of the exon–intron boundaries by aligning the genome sequences with the human and zebrafish *TAS1R* sequences and by the GT/AG rule. Because a certain degree of base errors was observed in the genome assembly for axolotl, sequence correction was needed for our *TAS1R* identification. We retrieved the raw reads of the public genome data and RNA sequencing data corresponding to the *TAS1R* exons using bowtie2 (ref. ^43^) and blastn and used that data to correct the *TAS1R* sequences by checking the alignment. The *TAS1R* amino acid sequences identified for axolotl, coelacanth and bichir were used as queries for an additional tblastn search of other vertebrates.

### Phylogenetic analysis

For the full-length amino acid sequences, non-homologous residues were masked using PREQUAL^44^ and the sequences were aligned using MAFFT v.7.427 with the ginsi option^45^. The phylogenetic tree was constructed using RAxML as described above. In addition, a maximum-likelihood tree was constructed under the posterior mean site frequency approximation^46^ of the JTT + C20 + F + Γ model with 1,000 bootstrap replicates using IQ-TREE v.2.2.2.6 (ref. ^47^). Bayesian tree inference was conducted with MrBayes 3.2.6 with the JTT-F + Γ_4_ model^48^. Two simultaneous runs were carried out with 10,000,000 generations, of which 2,500,000 were discarded as burn-in, and convergence was assessed with Tracer^49^. Trees were visualized with iTOL^50^. Alternative tree topologies were evaluated with the approximately unbiased test with 100,000 replicates using CONSEL v.0.20 (ref. ^51^).

### Synteny analysis

The synteny of genes proximal to the novel T1Rs was analysed using annotations available in Ensembl 97 (ref. ^52^) for human (GRCh38), chicken (GRCg6a), anole lizard (AnoCar2.0), coelacanth (LatCha1), zebrafish (GRCz11) and spotted gar (LepOcu1). For bichir, annotations were conducted using Cufflinks on a draft assembly. The gene annotation for axolotl was obtained from the Axolotl-omics website (AmexG_v6.0-DD)^53^. NCBI annotation was referred to for the West African lungfish (PAN1.0) and elephant fish (Callorhinchus_milii-6.1.3). Novel *TAS1R*s were added to the gene list in our synteny analysis if they were not accurately identified in the public annotation data.

### Conserved motifs in the sequence upstream of *TAS1R4*

Sequences up to 300 bp upstream of the *TAS1R4* open reading frames were collected for whale shark, bamboo shark, cloudy catshark, elephant fish, bichir, coelacanth, axolotl, two-lined caecilian, Japanese gecko, anole lizard and central bearded dragon. The sequences were aligned using MAFFT^45^ and then used for MEME analysis^19^ to search for a maximum of three conserved sequence motifs. The motifs discovered by MEME were then used for comparison with known transcription-factor binding motifs in TRANSFAC v.11.3 using STAMP^54^. The known Oct-11/Pou2f3 motif was obtained from JASPAR^55^.

### Experimental animals

This study was carried out in accordance with the National Institutes of Health guide for the care and use of laboratory animals (NIH Publication No. 8023, revised 1978). Both male and female bichir (*Polypterus senegalus*), ~5–7 cm body length, were purchased from a local commercial source. We found no differences in the expression of genes encoding T1Rs or downstream signal-transduction molecules, such as TRPM5, Gαia1 and Gα14, between male and female bichir by in situ hybridization.

### Cloning *TAS1R*s of bichir and elephant fish

*TAS1R1*, *TAS1R2A*, *TAS1R2B*, *TAS1R3B*, *TAS1R4* and *TAS1R8* were amplified by PCR from the genomic DNA or cDNA of bichir. *TAS1R6-1*, *TAS1R6-2*, *TAS1R6-3*, *TAS1R3C* and *TAS1R4* were amplified by PCR from the genomic DNA of elephant fish (*Callorhinchus milii*). PCR and Sanger sequencing for the coding sequences of their *TAS1R* genes were performed using specific primers designed based on the annotation from the whole genome assemblies. The PCR products of the exons were assembled into one full-length sequence using overlapping PCR (In-fusion cloning; Clontech) for each *TAS1R* and were then subcloned into the pEAK10 expression vector (Edge Biosystems).

### Functional analysis of T1Rs

Responses of the T1Rs to various taste-associated stimulants were measured using a cell-based luminescence assay, as described previously^22,23^. Briefly, HEK293T cells were transiently co-transfected with an expression vector for an individual T1R along with a chimeric rat G protein (rG15i2) and a calcium-binding photoprotein (mt-apoclytin-II). Cells were seeded in 96-well plates and assayed 2 days after transfection. Cells were exposed to each taste stimulant individually, and luminescence intensity was measured using a FlexStation 3 microplate reader (Molecular Devices). The response in each well was calculated based on the area under the curve and expressed as RLU. Data were collected from three independent experiments, each carried out with duplicate samples. We adapted a strict definition for the positive response as >10,000 RLU along with a statistically significant difference against control (buffer) with a false discovery rate (*q*) of <0.01 (one-sided *t*-test). A limitation of this assay is that concentrations of amino acids and sugars were presented at a maximum of 50 mM or 100 mM to avoid receptor-independent calcium increases, caused for instance by changes in osmolarity^23^, which can prevent the accurate assessment of responses to higher ligand concentrations. The osmotic pressure of each of the Arg and His solutions was higher than those of the other amino acid solutions because large amounts of HCl or NaOH were required for pH adjustment; this may have caused the higher response to 50 mM His of bichir T1R2B/T1R3B (Fig. 4c).

### In situ hybridization

In situ hybridization was performed as described previously^9^. In brief, fresh-frozen sections (10 μm thick) of bichir jaw tissue were placed on MAS-coated glass slides (Matsunami Glass) and fixed with 4% paraformaldehyde in phosphate-buffered saline. Prehybridization (58 °C, 1 h), hybridization (58 °C, two overnight sessions), washing (58 °C, 0.2× saline–sodium citrate) and development (nitroblue tetrazolium/ 5-bromo-4-chloro-3-indolyl phosphate; NBT-BCIP) were performed using digoxigenin-labelled probes. Images of stained sections were obtained using a fluorescence microscope (DM6 B; Leica) equipped with a cooled CCD digital camera (DFC7000 T; Leica). Double-label fluorescence in situ hybridization was performed using digoxigenin- and fluorescein-labelled RNA probes. Each labelled probe was detected sequentially by incubation with a peroxidase-conjugated antibody against digoxigenin and peroxidase-conjugated anti-fluorescein (Roche) followed by incubation with tyramide signal amplification (TSA)–Alexa Fluor 555 and TSA–Alexa Fluor 488 (Invitrogen) using the tyramide signal amplification method. Images of stained sections were obtained using a confocal laser-scanning microscope (LSM 800; ZEISS). The entire coding regions for the six T1Rs and two G protein α subunits as well as the partial coding region for Trpm5, all of which were amplified from bichir cDNA synthesized from lip tissue, were used as probes for in situ hybridization.

### Reporting summary

Further information on research design is available in the Nature Portfolio Reporting Summary linked to this article.

### Supplementary information

Supplementary Information Supplementary Data 1 and 2.

Reporting Summary

Supplementary Tables Supplementary Tables 1 and 2.

## Data Availability

The *TAS1R* sequences and phylogenetic trees are provided in Supplementary Data 1 and 2, respectively.

## Code availability

No code was generated in this study.
