# Supplementary material for: A vertebrate-wide catalogue of T1R receptors reveals diversity in taste perception
Source: Nat Ecol Evol. 2023 Dec 13;8(1):111–20. doi: 10.1038/s41559-023-02258-8 (PMC10781636; doi:10.1038/s41559-023-02258-8)
Supplement: Supplementary file 1 — Supplementary Data 1 and 2. [file 41559_2023_2258_MOESM1_ESM.pdf]

nature ecology & evolution

Article

https://doi.org/10.1038/s41559-023-02258-8

A vertebrate-wide catalogue of T1R receptors reveals diversity in taste perception

In the format provided by the authors and unedited

>Homo_sapiens_T1R1

MLLCTARLVGLQLLISCCWAFACHSTESSPDFTLPGDYLLAGLFPLHSGCLQVRHRPEVTLCDRSCSF
NEHGYHLFQAMRLGVEEINNSTALLPNITLGYQLYDVCSDSANVYATLRVLSLPGQHHIELQGDLLH
YSPTVLAVIGPDSTNRAATTAALLSPFLVPMISYAASSETLSVKRQYPSFLRTIPNDKYQVETMVLLLQ
KFGWTWISLVGSSDDYGQLGVQALENQATGQGICIAFKDIMPFSAQVGDERMQCLMRHLAQAGAT
VVVVFSSRQLARVFFESVVLTNLTGKVWVASEAWALSRHITGVPGIQRIGMVLGVAIQKRAVPGLKAF
EEAYARADKKAPRPCHKGSWCSSNQLCRECQAFMAHTMPKLKAFSMSSAYNAYRAVYAVAHGLHQ
LLGCASGACSRGRVYPWQLLEQIHKVHFLLHKDTVAFNDNRDPLSSYNIIAWDWNGPKWTFTVLGS
STWSPVQLNINETKIQWHGKDNQVPKSVCSSDCLEGHQRVVTGFHHCCFECVPCGAGTFLNKSDL
YRCQPCGKEEWAPEGSQTCFPRTVVFLALREHTSWVLLAANTLLLLLLLGTAGLFAWHLDTPVVRS
AGGRLCFLMLGSLAAGSGSLYGFFGEPTRPACLLRQALFALGFTIFLSCLTVRSFQLIIIFKFSTKVPTFY
HAWVQNHGAGLFVMISSAAQLLICLTWLVVWTPLPAREYQRFPHLVMLECTETNSLGFILAFLYNGL
LSISAFACSYLGKDLPENYNEAKCVTFSLLFNFVSWIAFFTTASVYDGKYLPAANMMAGLSSLSSGFGG
YFLPKCYVILCRPDLNSTEHFQASIQDYTRRCGST

>Mus_musculus_T1R1

MLFWAAHLLLSLQLAVAYCWAFSCQRTESSPGFSLPGDFLLAGLFSLHADCLQVRHRPLVTSCDRSD
SFNGHGYHLFQAMRFTVEEINNSTALLPNITLGYELYDVCSESSNVYATLRVLAQQGTGHLEMQRDL
RNHSSKVVALIGPDNTDHAVTTAALLSPFLMPLVSYEASSVILSGKRKFPSFLRTIPSDKYQVEVIVRLL
QSFGWVWISLVGSYGDYGQLGVQALEELATPRGICVAFKDVVPLSAQAGDPRMQRMMLRLARART
TVVVVFSNRHLAGVFFRSVVLANLTGKVWIASEDWAISTYITNVPGIQGIGTVLGVAIQQRQVPGLKE
FEESYVQAVMGAPRTCPEGSWCGTNQLCRECHAFTTWNMPELGAFSMSAAYNVYEAVYAVAHGLH
QLLGCTSGTCARGPVYPWQLLQQIYKVNFLLHKKTVAFDDKGDPLGYYDIIAWDWNGPEWTFEVI
GSASLSPVHLDINKTKIQWHGKNNQVPVSVCTRDCLEGHHRLVMGSHHCCFECMPCEAGTFLNTS
ELHTCQPCGTEEWAPEGSSACFSRTVEFLGWHEPISLVLLAANTLLLLLLIGTAGLFAWRLHTPVVRS
AGGRLCFLMLGSLVAGSCSLYSFFGKPTVPACLLRQPLFSLGFAIFLSCLTIRSFQLVIIFKFSTKVPTFY
HTWAQNHGAGIFVIVSSTVHLFLCLTWLAMWTPRPTREYQRFPHLVILECTEVNSVGFLVAFAHNIL
LSISTFVCSYLGKELPENYNEAKCVTFSLLLHFVSWIAFFTMSSIYQGSYLPAVNVLAGLATLSGGFSGY
FLPKCYVILCRPELNNTEHFQASIQDYTRRCGTT

>Gallus_gallus_T1R1

MPPPRAALLRVLLCARLCAAAFRSPGEFRLAGLFQIHALRPGRPLAHGCGVAAAFRSHGYHLSQMMR
FAVEEINNSSALLPNVTLGYEIHDTCTEAANLHGTLRALGREGRHDVEVLSAPQRYEPRAVAVIGPDS
TQLALTTAAILGVFLVPEISYEASLEMLSTKRFYPSFLRTIPSDGQQVKAIGLLLQRFGWTWVALVGSD
NTYGRDGLNALSELLAATDVCVAYRGVIPTTKDAGSPELRKLIQTLVDSRVNVTVVFSNRRNAQPFF
EAVVQENITGMVWVGSEDWSLAQTIWQVPGIQNIGSVIGISVEQAEPTMLKRLESWENARERAVSGS
AGSTGVGGGNGASSSDGIQLNCTQHCPGCHLLADTPDIYDIQASYNVYSAVYAVAHGLHNLLGCAS
GVCSKGRVYPWQLLQKIKQVNFSLHKSYISFDANGNIRKGYNIIAWNWRGQSWAFDVVGAFTVNPN

RLHIDQSKILWHTKDHQVPVSVCSWPCAAGEMRLQQNRHRCCFSCVACPAGTFLNRTALYACQAC
GRDEWAPVGSETCFNRTVEFLSWADPLSWVLLIPTVLLLLLMAGLAVLFARNASTPVVRSAGGKMCF
LMLGALACTCSSIFFNFGEPTWLSCLVRIPLFTISFAVFLSCVATRCFQIVCIFKLSTRWPALHEAWQRR
GGPALFIAGSTVAQAVLSVAAVASGPAGPRRRYSVAAERVVLECGAGSAPGETAAILYNLLLSLGCFAL
SYAGKDLPADYNEAKCLTCSLLLHLACSAAVLCTRSYFRGRSAAVTAALGALGTLAPLLGGYFLPKGF
VVLLRPHLNTAERFQQEIRSYTRRRDE
>Gekko_japonicus_T1R1
MGHSLPPFLFLSVLVLSGYISVAQDLSSPLRKDGDYTIAGLFRIHRITFEMKSRPEVDICASMGNRSSH
GYYLAQAMRVSIAEINNSRCLLPNVTLGYEIYDICSQLTNLYATLGLLSQGAEGCHSHQHIPVADNYT
GYLPKAVAVIGPDSSENALITASLLGIFLMPEISYEASSPILSRKRAHPSFLRTIPSDRLQAEALVRLLKDF
QWTWVAAVGSDNTYGRQGLHTLHEVAIKEGICFAYQGIIFMETSSQELVENVHAVLASKTEVVILFA
NKRGATMFFQEVVRQNVTGKVWLGTEDWSLSREIWDIHGIRSIGTVIGVTIMQAPLPEMWDFEAAS
RHSESHRPQASCFQGCNKTCSQLCSQLYVPALQPLLEPSPYDTQAAFNVYSAIYAVAHSLHRLLGCRT
GVCRKNTVYPWQLLKEIKEVNFSVKGRQIYFDSNGDPLTGYDIVQWKWADQIWSFDVIAIFSSNPVN
LTIHWEKLQWHTRDNQVPVSVCSKDCAVGEQKVLQGIHQCCFHCVTCSPGTFLNKSNPYTCQMC
QEDEWAPAGSETCFPRATIFLHWSDRISQALLSAATLLLVLLAGALFVFAQKANTPVVRSAGGWLCF
VMLSALAGANASLYCYFGVPSRYTCLLRTSMYTVSFNICLACMAARSFQIILIFKMASKAPGLYEAWKN
HHGCGLFIGACTGLQGAIFLIYLCATPPFPHKNYDTSDQVTLLECKERSSVLSLLGFVGNGLLGIFCF
VISYMGKDLPNSYNEAKCTTFSLIIYFASLISYSTTFSVYRGKYLPAIHVASLLLPLFGIFGGYFVPKIYPL
QI
>Pogona_vitticeps_T1R1
MAPSSLLFLHLPLWALWGGSSQGLSTPLTLEGDYMIAGLFPIHRKNIIRSQKSKPEVDVCKSPYDNRN
VHGYHLVQAMRFAVEEINNSSCLLPNLTLGYEIYDSCTSSTNMYATLSLLSKDREGCIGHQQVEVDA
NYAQYLPKAVAAVGPDSSDEAVLTASLLGIFRIPVVSYEASSPVLSLKYLYPSFLRTIPPDYMQVCALIH
LLEAFNWTWVAVVASNNNYGIQGLQKLHERSARVGICIAYQGILPAEPDANRSELSTIVHNLVSAGAS
VTIVYANRQSIGSFFQEVIRLNVTGKVWLGTEDWSLASDISMIHGIRDIGTVIGVALKEAHQLGMKAF
EAALVGCKEATGGLNDIGSARSSCQHCREACSQLCTQFCRPDEWEPSHSGLEISPRDSRAIFNVYSAV
YTVAHGLHRLLDCQTGECRKDTIYPWQLLKEMRQANFSLFHRQVRFDTNGDILDGYELVLWNWA
GQTWNYSVIGSYDNSGGLSIDKEKLLWHTKDNQLPVSVCSLDCGPGEEKIQQGTQRCCFHCLPCSS
GTFLNKSSFSTCQKCKEDQWSPAGSEACFDRSVEFLAWDERVSLVLLTCISLGLLLMVGTGALFIWH
LQTPVVKSAGGWLXXXXXXXXXXXXXXXXXXXXXXXXXXXXXXXXXXXXXICLSCMAARSFQIVIIFK
MATKTPGLLEAWRRHHGSSVLIGSLTAVQGVITLVHLSIRPPIPQKNANAYDNLTVLECSVGDTTLW
LSGLLYNTLLGIVCFMISYMGKDLPSSYNEAKCITFSLLIYFACFVSDFVTRRLYTGQYLTAIFVTSQLIN
LCGIFGNYFVPKVYIILFHSERNTSEHFQMSIQSYTKRINAAG
>Anolis_carolinensis_T1R1
MRMGLFVLFALSLSFTCGQVCSSALNLEGDYSIAGLFPLHRSSRAGQGQVGRPQVEVCLRAPSRSAH

GYHLVQAMRFAVSEVNRATDLLPNVTLGYEIYDACGPLANLYATLRLLSEADRAGRVAVPVAANYTF
YRPRSLAAIGPDSSQEALTTAILLGAFLVPEVSYEATSPTLSQRRDFPSFLRTVPSDRLQVEALVLLLSSL
GWTWVGLVGSDNDYGRQGLRMLQEVAPRHGICFAYQGFLPASGADTELVGIVRDVSSCGANVAVV
FANKNSARAFMEEAVRQNVTGKVWVGTEDWSLSTEVWSVGGVSGVGTVLGVSVEQAHLPGMQDF
EAVSATSETEDSQPAGCSRTDAAVSRNEGEMCSQACSRLRRPKSLTDWEMSPHDIQGAFSVYSAVYA
VAHGLHRLLGCHTGPCRKQTVYPWQLLREVREVDFNLSHRRVRFDENGDTAAGYDLVQWVWEGQ
EWGYRVIGSFEQPDRLDLDQEKIRWHTQDNQVPTSVCSRDCGAGEQKVPQGTHRCCFHCVACLPG
TFLNRNDLYTCQKCRREEWSPSGSESCFPRETVFLPFSDPVSLALLTAACLLLSLLAFTAALFVRHLGT
PVVRSAGGPFSLVMLGALAGATLSVYGYFGPPGRLGCLLRMPLYNVNLSLCLSCMLTRSIQIALIFKA
AGGLYDTWRRVKGPALLIVTLTGLQGLMVVISFATNHPTPRRDYSLSATSIFLLCSSEESIPVVAGGVY
NGFLGVFCFGLSYLGKDLPSSYSEAKCLTFSLMVYFTSSISYVIASSVYVGRYLSAMYVASLLVTLGGVS
TGYFSPKVFLILFRPELNTNQHFQMSIQSYTKRINAGD
>Ambystoma_mexicanum_T1R1
MLGSTVVLGLLLFSRANLAFGCQDAAPFSEFRRPGDYIIAGAFPIHRGKTVSKSKPEIDICQSVSTFDD
HGFHLFQAMTFAIEEVNNSSDLLPNVTLGYEIFDTCSESASVFATLKILTQCAQPYVTIGHSFLGYRPK
ALALIGPDRSTFSSTTASILSNFLMPQIGYEATSENLASKNLYPSFLRTIPSDELQVNVMLLLLRKFKWS
WVAVIASGNEYGRQGLQSLSRLAAKNGICVAYQGIIPDNGEDNRLAIQQMLRQINQARVQTVVLYSN
RHTAKTFFEVVIVENITDKVWLAPEDWSGSPLITRLQNIERIGSVMGVSVKHSVDISLKEFEVTYLNTV
KQHGQPVGGREGCNQVCEKCRSFIAENISLASGSRSPAVFNVYSAVYAVAHALHDLLDCKKECSKET
VYPWQLLEKIKKVNFTLHNQSISFDDNGEPLTGYDIVMWNWNEQNVSVRVIGSYSQRPSKLHINEEG
LKWHTKDNTIPVSICSKECNAGERKILTGVHLCCYSCKACPKMTFLNASNPFSCEPCERDQWSPPSSV
TCFHRTVEYLSWSNPLSLVLLPAITIQLLVTLAIAVVFMHNLDTPVVKSAGGIRCLIMLASLACACGSL
YLYFGEPTQETCLLRQPLFAVSFTICFSCIVVRSFQIVCIFKLASKAPKLHDFWVKKNGPNIFIGVSSTA
QLLISILWVSLKPPERVADYMKFENQILLECSESSSVGSVLGILYIGLLSMFCFIFCYIGKDLPANYNEAK
CITFSLLVYFVSWIAYFAIYIAYKGKYITAVNILAILSSLLGILSGYFLPKCYVILFRRELNTTEHFQSSIQN
YTKKRSSN
>Nanorana_parkeri_T1R1a
MVVVSALDRCFALLLHLCAALGCQISNYKSEYRLPGDYVIGGLFPLHKADSTQFPMLGLNYCDGSTF
NILGYHLLHSTRMAIEEINNSSTLLPNVTLGYELYDTCSDPTHIYGVLRNLYRCEEPYLQMQNNFTNY
KTKAIALVGPSSSSFAFVAGRVLGKFLVPQISYSASNEQLGNKRTYPSFFRTIPSDKLQVEVILNLLQKF
KWTWVAIVGSDDVYGRQGVQDLNTLASKNGICIAYQGLIPLSTDTTNVKKMVANIVQTRVKATVVF
SAYFNARIFFQEVLNANVTDFVWIGSESWIMDTQITAIANIKRIGSVLGVSVGQVFFPKLLEFETEYVTS
PKIKTMGQNGCNQVCLDCQAFTLQTMPIQTQFSMSASFNAYSAVYAIAYALHDLLDCKSGQCSTNT
FYPWQLLQKVKKVNFTMYNQTINFDSNGDPATGYDIVMWSWSEDTPTFKLIGSYSKTTQQLQLND
KLQWYTKDNTVPESICSKECETGERRVQTGSHTCCFECLPCPQMSFLNASDLYTCQPCGTDQWSP
MKNDICYNRTVEYLPWTDPLSLVLLFLVTLLLLVTIAIAVLFVINLNTPVVKSAGGKMCLGMLLSLAL

SLCTLYCYFGEPGIITCMVRQPVFAVSFTICFACIVVHAFQIVCIFKMATHMPRIYDIWVKKNGSDVFIA
ASTAIQVLISVVWIVVKPPRPIADYSTFSDQIILKCSETVSIGSIAEIIFIAFLSMICFIFCYMGKDLPANYN
EAKCISFSLLVYFFSWVGYFTTYIIYQGKYLAAVNVGAVLLSVFGILAGYFTPKCYIILLRPEMNTTEHF
QSAIQDYTKKQSAHD
>Nanorana_parkeri_T1R1b
MPFLYSLFQLCLASDCGDVTYNTEFSSPGDFVLGGLFKVHDIEDVSTRSIPLIDVCHRGTFISLRFHFL
QAMRLAIEEINNSTILLPNITLGYEVFDTCSEAANIYGTLRMLSQCSQPYIKIQNNFTNYKPNIIALIGPY
SSSFSFVTANIMSLLLVPEISYSASNKHLSLKQMYPSFFRTIPSDNLQVQIMLSLLKTFNWTWVAMVGS
DDVYGRQGLQDLIALTTKNGICVPYYGIIPNTNDRTYIKKMVDNIVQTHVGVIVVFSAYNSAQILLEE
VIKANITGRVWIGSEDWLVYPEIAALPNIQSIGTIFGVSVSEIYFPKLLDFETAYVSSFKSQDVSEYNCNQ
VCDKCRSFTLQNMSIPGQATMSASFNIYAAAYAIAHGLHGLLNCRSGQCSNGTYYPWQLLQHVKKV
NFTLYNQSISFDANGDPAMGYDIVMWSWIRESPTFRVVGSYSKTTQRLQLNERLPWNTKNSTVPESF
CSTECGKGERRVQTGSYVCCFNCFPCPAMTFLNTSDLYSCQPCGSDQWSPMRSDSCYNRTLEYLSW
TDPLSLILLSVITLLILVITATATMFVMNLSTPVVKSAGGKMCLGMLLSLALSCLTLYFYFGKPEVVTCI
VKQPIFALSFTVCFSCILVHVFQIVCIFKMSSLMPKLYDLWVKKNGSDIFIAVSTAIQVLISVVWIAVKPP
RPTADYSTYPDQIILKCSETVSVGSIAEITYIGFLCMICFIFCYMGKDLPASYNEAKCISFSLLVYFFSWIA
FFTTYIVYQGKYLAAVNVCAVLFSVLGILIGYFVPKCYIILFRPELNTTEHFQAAIQDYTKKQSAQE
>Rhinatrema_bivittatum_T1R1
MLFPSELIFPLYLYVALDLAPRCQATDHPSEFSVPGDYIVAGAFPIHDASANIKSSPEVDECDRSALND
HGFHLFQAMRFSVEEINNSSSLLPNVTLGYEIYDICSESSNLYATLDFVSQRSFPFVTVQNNFTNYQPQ
AIAVIGPDDSDLAFTMASILGLFLIPQISYEATNELLSVKDVYPSFLRTIPSDKFQVEVLALLLRHFNWT
WIAILGSDNDYGRKGMQSLYDLTDEFDICVAYQAVIPSSADGQEAEMKHIVSNLAQYEVQVVVVFAT
KNLAKVLFEVTIQENATQMVWLGTEDWLMSPLIFSIPNIQRLGTVMGISVKQVGLPGLREFEIAYLNS
EKSRRNSQTVRESCNQICRECQSSTPQSMPVPSAFDLQASFNVYAAVYAVGHALHGLLDCESGACNR
GTFYPWQLLERIRNINFILHNQTIRFDEKGDPYIGYDIAMWEWTRQRWSSVVIGSFTRNLRRLDIIKD
NLKWHTEDNKVPVSVCSKECPAGEQRVQKGPHPCCFDCKPCPKMTFLNKSNLYDCQFCTSREWSP
PKSHACFSKTIEYLSWTEPVAMVLLFAIMVLFLLALAIAAVFALNLNTPVVRSAGGKMCFTMLGSLAC
ACCTLFCYFGMPTKMTCMLRQPMFAISYTICLSCLVIRSFQVVCIFKLAAKFPTFCDFWVKKNGPNVF
LCVSSVFQVMISIIWISSQPSVPIENLENFDSQIVLECSESISKEAIIEILYIGLLSIFCFVFCYLGKDLPENY
NEAKCITFSLLIYFISWISFFTIYIVYKGKYVTAVNVLAVLASLFGILGGYFVPKCYVILLRKDLNTPEHFQ
TSIQNYTRKKNSD
>Neoceratodus_forsteri_T1R1
MLPVVAVLLLLQSVCPVSLAIDCKGLGGSSSEFKTPGDYIIAGLFPIHSSNTTSSSSRPEVGICQGTAPF
NKHGYHLFQAMRFSIEEINNSSTLLPNVTLGMDVFDTCSESANLQATLDLLSRDVQPKAVEVYRDLS
EYQPKVMAVVGPDNSGYSFTTSSILSLFLLPQISYESTSEMLSIKRLYPSFLRTIPSDKLQVELMVLLLQ
TFAWSWIAIVGSDNDYGQQGVQSFYDLATKRGICIAYWGIIPANAKDDKGALQQMINSITQTRVNVV

IVFSTKRLATQFFLVVVEQNVTEKVWIGSEDWSISTMVSSIPNIQRIGTVIGVSVKQAPLPGLQQFEDA
YVESANLKIYHKFQQCLNPTQTMKSNNDTVNGCNKVCTECQLFTPRSLPILDMSDVQASFNVYSAV
YAVAYALHALLGCDSGRCSNEAHYPWQLLGKLKEVNFTLYNKTIFFDENGDPPTGYDIIVWDWTAN
WSFNVIGSFTPNPGRLEIDKAVIKWHTPNNMVPLSMCTAECEPGQRRKQTGIHPCCFNCEDCTEGT
YLNKSDLYVCQSCDMEHWSSLKSEICLSRTIQYLEWTDPISVGLLFAMAVVFLLTVSTIVIFMLNLDTP
VVKSAGGKMCFVMLAPLACACCGLICFFGRPTHTTCVLRQPIFAVCFTICLSCMTIRSFQIVCIFKMTA
KMPRFCDYWVKGKGPNVFILTSSAIQTLISVTWIAVQPPVPFSSYTIYKDQIILECSQGTSVGVMLEILY
IGCLSMFCFVFCYIGKDLPANYNEAKCITFSLLIYFISWISFFTTYIVYKGKYITAVNVLAILASLFGILLGY
FIPKCYVILLRPELNTTEHFQMSIQNYTKQRNAN
>Latimeria_chalumnae_T1R1
MRFNLPQANEQTLIPNLCCDCEVQLFAPSCLVLDLPGLKCSFLDFSLSGDYLIGGLFPIHTNVNVGSS
SRPQVDKCQGNAQFNAHGYHLFQAMRFAVEEINNSSTLLPGLTLGYDIFDICLDSANLQATLDLLSQ
EAWPYVEVKKDLSSYRARVLALVGSDSSTYSFTIASILGHFLMPQVSYESTNERLSIKRLYPSFLRTIPSD
KIQVEMIISLLHKFGWTWIAVIGSDNDYGQQGLQNLRYRASKNGICIAYQGVIPASAQQMRETVKSVL
QTRVSIIVVFSTKQLATTFFKEIITQNITGKVWIGTEDWSIATLVSSLHDIKNIGTVIGASVKQAQLPGF
LSFEATAVKSQTGEFQDTCDSVGSENYGAGTMGCNQECHECQWLNLDNMVTPNIQAAFNVYSAVY
AVACALHVLLECDLGECSKEAPRPWQLLKELKQVSFKLYNTTIMFNENGDPPGGYDLVIWDWTTA
QWSVKVIGSYSLNQGFEIDADKIKWHTEHNKVPRSVCSEECQPGAKRKQTGVHACCFDCETCPGGT
FLNRSNLYSCQPCDREHWSQEGSEMCWNRTIEYLSWTDTISLMYLPGMALVFLLTLLVAVTFALNLD
TPVVKSAGGRMCFIMLVSLAASCCITLCFFGKPTKLKRLLRLPVFXISFTACLSCIAVRAFQIVCIFKMA
AKLPKVYDYWVKKNGQTVFIIVSFLIQVVICMARASSRPPVPHQNYEIYKDQIILDCSENTSFGSVLVIL
YIGILSVLCFTFCYMGKDLPENYNEAKSITFSLLIYFISWISFCTTYIVYQGKYIAALKAMAILASVLGILG
GYFLPKCYVILLKPQLNTEHFQTSIQSYTRKRSDQAS
>Takifugu_rubripes_T1R1
MQLQMKMLVAVLSATPLMLQLVTGELDYSTHGQGMQLHGNFSIAGFFPLHYGEKLDGSLPALELC
KDGEINKHGFHLLQAMKLAVDEINKDAGTQALLPGVVLGYQLYDTCTVSAGILASLDVLEYWSPSAS
GKVPNFDISQRPLAVIGPDSSSNSFTPATLLGAHLIPQISYEASNEMLSNKVLYPSFFRTIPSDKNQVAA
MIQLLVRFNWTWIALLGSDNSYGLEGMQSLSQQAPEFNICIAYQGVIPGYTQDTVQVMRNIVDSILK
TKVTTIVVFSSKSKLSKFMPFVIEQKVTGKVWIGTEDWSPSSLISGIPGIHTIGTVIGVAVKYTIIPGFEK
RLVEASLHQSNDGNASNVTANLSNTCLQSRDLYSLAEMNFPLDNYDITSAINVYKAVYAVAHALHQV
LDCDSGECQRKRVYPWELLSRLKQVRFLMANSSVYFDSNGDPPTGYDIICWVWHGTEWSVRRVGSF
SPNPISLTIDADKIEWHISGDSRSVPQSICSPPCEPGHRRLLTGQHSCCFDCQACSEATFLNTSDPTSC
QDCLPEEWAPKSSERCLKRTPLLLEWDHHLSIALLFFLACCLLMTSSSAVILLLNINTPVAKSAGGRTC
LLMLAALTAAAMSSLCHFGQPSPLACILKQPLFTFSFTVCLACITVRSLQVVCIFKFASRLPPAYDKWS
KKQGPEVTIFIVSVTILCISVLRVAVGPPEPSQDLDFYMDSIVLECSNTLSPGSFIELCYVCVLSVLCFFF
SYMGKDLPANYNEAKCVTFSLMVYMISWISFFTVYLISRGPFTVAAYVCATLVSVLAFFGGYFLPKIYII

VLKPQMNTTAHFQNCIQMYTMSKQ

>Danio_rerio_T1R1

MGTMLSNFVFLCLLRFSDSTGLHLQGDYILSGWFALHNSDSTIPPTPYLNDCKKGLTNKHGYHLVQ
AFRYAVDEINNGTQDKQLLPGVTLGYRTYDICSLPASNLATLDLLAQQLHPSAVDSRAVAIIGPDSSS
YAFTPAAALGVFLVPQISYEATNELLSNKLLYPSFFRTIPSDKNQVNAMIQVLVKFNWTWIALLGSDN
SYGIQGMQSLSEQAPLNNICIAYQAQIPAVTDSTKKYMQDMVKNILKTKVNTIVVFANKRRAAGFFPF
VIAQNVTGKVWIGTEDWSVASTVSSIPGISMIGTVIGVAVKYTEFDGFDNYERLSVPGLKNPGQFNLS
VPCMQNTNLYDIAINGFSLSQYDITSSFNVYKAVYAVAHALHNVLDCDSGLCQKYDVQPWQVYEQL
RQVRFSIRNASFYFDKNGDPPTGYDIVTWEWTNGLWSFKVVGNYSPGANDLHLDDSQIDWSGQVSP
VTNVPESICSPECPYGFRKLMTGQHKCCFDCMACPAATFLNKTGYTSCQPCPIDDWSEAESEVCLPR
AELYLSWGAPLTTALIIYLAVTLFVTLGTTLVFLLNLSTPVVKSAGGKTCLLMLVSMIVACCSTLCHFS
RPSRVGCLLKQPLFVISCTVCLACVTVRSFQVVCIFKWSSKLPRSYETWAKNRGPEMFILITTVVETFIS
VLRMLLVPPFPSRDYDFYHDSIVLECSKTLSLSAFAELFFVCALSLVCFCLSYMGKDLPANYNEAKCIT
FSLMIYMISWITFFTAYCISRGSFVMALNVGAILLSVLGILGGYFLPKVYIILIKPQLNTTAHFQNCIQMY
TMAKQ

>Lepisosteus_oculatus_T1R1

MAVCGRVVPLQAFGLVTWAAVLQCWDLAAAGQFGPLSQFQLGGDYVIAGLFPIHNTGMSDKSVPE
LDDCQNGIFNKHGYHLMQAMRFAIEEINNGSGGENLLPGVTLGYQIYDICSEPASILATLDVLVQQY
EYNREPESPSTSRTRAIAVIGPDSSSYAFTSAATLGYYLIPEISYEATNEMLSNKQLYPAFFRTIPSDRNQ
VRAMVQLLVRFKWTWIALLGSDNDYGQQGVQSLSELASEYGICIAYKGIIQTYTDSRREEMLQMVRSI
VDTRVNTIVVFSSKRIASGFFPLVVGQNVTGKVWIGSEDWSISTRVSGLPGIERIGTVLGISTKTATLP
GFEGFEALTVEKTRRPVANESEDERQRALGCLQGSDAFGTLTPGEVPLKEFDVQSSFNVYKAVYALA
HALRSLLDCDSLACSRVEALPWRLLEKLRQVNFSVGSEPVYFNEHGDPPAGYDIMAWDWAGGNLSL
RLVGMYSPDPENLDIDASLINWNTGRESVVPESVCSPECPEGYRRLQTGAHACCFDCQACPPSTFLN
RSGYTFCQPCKQREWSLPESEHCVNRTVVYLPWEDSLAVALLVSLVLTLLLTLATALLFLLHLNTPVV
KSAGGKTCLLMLASLAGATCSSFCHFGVPSGLGCALKQPLFTFGFTVCLSCMAVRSFQIVCIFKMASK
LPRAYEVWARKRGPQAVILSSSVVALFVSLLHVLLARPVPTEVYNIYPDTIILECSGMESVSSILQLVYIA
MLSFLCFAFSYMGKDLPANYNEAKCITFSLLIYLISWLTFFTVYIISRGKYLIVVNVMAILASVLGILGGY
FMPKVYIIVLKPQMNTTAHFQNCIQMYTTKKSEN

>Amia_calva_T1R1

MGLCHPCLIAWLLFWACWDQGGAERPNLLSQFWLEGDYVIAGLFPLHNADRSTKPVPEMDDCRRG
TFNKHGYQLMQAMRFTIEEINNGSSRDGLLPGVSLGYQIYDTCTEPASILATIDLLVQQSNYYNVKKL
NGTLESRAIAIIGPDSSSYAFTSATTLGYYLMPQISYEASNQMLSNKLLYPAFFRTIPSDNNQVRAIVQL
LRRFNWTWIALLGSDNDYGQDGLQSLSTVASDNNICIAYQGIIPKYTDSTREQMITMIKKIMQTHVN
TIVVFSSKRIATGFFQLVVEQNVTGKVWIGSEDWSVSTMVSGLPGIQTIGSVLGIGVKTAKMLGFQEF
GVLAMEAAAYATGNSSNDTGEVAGHGLRCLQGCDHIQTLTTRDIPLELYDIQSSYNVYKAVYALAH

ALRSSLGCNSGKCAKEDVQPFNLLENLKQVNFSVGGTSMYFDQNGDPPTGYDIMAWDWAGGQLSL
RVVGTFSPDPADLKINASLINWNSDSKGTVPQSLCSPDCPKGSRRLQTGSHVCCFDCQICPVDTFLN
KTGLTFCQPCLIHEWSLSGSERCTNRTVVYLPWADWVSVVLLLASALTLFLTLVTAALFLVNLSTPVV
KSAGGRTCLVMLASLAVATCSTLCHFGVPTLLACILKQSLFTLSFTVCLSCMAVRSLQIVCIFKMSAKL
PRAYEVWARNNGPSIVIIVSAATELFISLMHVLEEQPSPTEVYNIYPHAILLECSGAQAIGSMLELGYIT
MLSFLCFAFSYLGKDLPANYNEAKCVTFSLLVYLISWLAFSTVYIVSRGKYSIIVNVIAVLASVLGILGGY
FLPKVYIIVLKPQLNTAAHFQNCIQMYTTKKSEN
>Polypterus_senegalus_T1R1
MHHSVFCVILCLVLPSTPDPLCSPTSLFEQEGDYKLAGMFPIHNIDSKAELTLELPNCKLGKFNKHGY
YLLQAMRFTMEEINSVRNTLLPNVKLGYRAYDICSETATLLATMDLFSRPAFPADQLLLNTTDYEPK
AIALIGPDSSTYAFTSASILGYFMYPLISYEATNEMLSNKKLYPSFFRTISSDNNQVLAILQLLATFRWT
WVAVLGSDNDYGKQGLQSISELAETYNICIAYQGIIPSYSSSTRPEMIKMINNIIQTKVNTVVVFSSKRIA
SGFFSLVMELNITGKVWIGSEDWSISTKVSGLPKIKSIGSVLGISTWALQMPGFANFVLNSFEKNRTQT
ACQRDLEKLKCIQDCDICASLTAAEAPVDLADIQSAFNVYLAIYAVAYSLHDLLGCSSGNCSQSKVQP
WQLLDKVRHVNFSLHNIPMFFDQNGNPPFGYNIISWDWIGDKVSYKVVGSYSPGHRHLEVNTTLIN
WNAQNSKTVPTSLCAPDCPSGYRRLQRGAHICCFDCEICPAMTYISDKDLYVCEPCQKWQWSPAAS
QTCFARTLEYLEMSDSLAVLLLVIAALTILLIAAVAAVFLRHLDTPVVRSAGGRTCLVMLASLASACSSI
YFHLGLPSHISCILQYSVFCISVTVCLSCIAVRAFQIVCIFKMSSNFPKAYEWWAKNNGPQMFILISSFTE
LFICILHVLLDHPLPIEEYSKFPDKIVLECSGSQSPVTIVELSFVALLSCICFALSYIGKDLPANYNEAKCI
TFSLLIYLLSWLAFFTTSSVYQGKYINAVNITATLASVLGILGGYFTPKCYIILLKPHMNTAAHFQNCIQ
MYTTKKSDY
>Takifugu_rubripes_T1R2Bb
MGCSLACLCLLGCVLYPLAQCTVPASEFRLEGDYLIGGLFEIHYEAFPTFHGRPQTMDCSSKFLILSNY
RRFQLMRFAVAEINNSTSLLPNVTLGYEVFDHCTDAQSFSDVFKLLSVNNVIQPWNVPNKNVSKVVA
VLGLFSSTSTLTVAPLFMSDFIPLISYGSSSSIFSEKAKFPSFLRTVHSSKKVMEVIVKILQYFKWHWVAF
LYSSDDYGTDGLKLFIEKIKDTEICLAYTHSLDSSHYLSVFNQIDAQRINLIVVFAPHWHAIPLVQAAIR
HNVTGKVWIAGEAWSLNKELPKEKGIKNIGTVIGVSQPVVTIPGFEDFLYSVKKGNRCESTEQQFCN
QIYNCSQESADILAADPSYSFSVYSAVYSTAHALHNVLNCDFGKCDNGIPISPPVLLAQLKKSNFTLLT
ERVQFDENGDPRFGSYAVLFWNQTGDPENFGTCCFYPSVKIFINDSKIQWHSKEVPISQCSKDCKEG
YAKRIEGIHQCCFKCEICPNGTFVNRTKDPFNCIDCEEHQWSAAGSTSCLPRTVEYVAFTDTAAVVIL
VGAGLLLALTLAMCVLFAINYNTPVVRSAGGPMCFLILGCLSLCSISIFFFFDKPTVAFCVLRFLPFVLF
YTVCLACFVVRSFQIVSIFKIAAKFPQAHSWWMKYHGQWLVISVTFVIQAFLIIVSVSSDPPSPFRDIVSY
PDKIILGCFMNLKTSSVSFVLLLLLCLLCFIFSYMGKDLPKNYNEAKAITFCLLLLILTWIIFTTASLLYQ
GKYIHSLNALAVLSSIYSFLLWYFLPKCYIIIFQPQKNTQKYFQGLIQDYTKTISQ
>Takifugu_rubripes_T1R2Ba
MAAVTMGPFLVCVGLLASVVHAAAHCHLPASEFVLHGDHLIGGLFDLYHVSSPIHHHRPEAVDCTS

QNFLLPNYRRFQLMRFSVEEINNSSDLLPDVSLGYEIFDICSDLQSFPGVLKQISVDGSVQAWAGSHAS
LSKVIGVVGPFSSTQTLTVAPLFMVDFISMVSYGASSSVFSSNENFPSFLRTVHPNKEVIDVIIRILQHFN
WRWAAFLNSDNDFGIDGRDLFIQGIKDTEVCLAYTQNLNMLTDFTQTFRQIEQQKIGVIIVFAPKMI
VEALIDSAIGLNVTNRVWIADDGWSMNKNLPKKKGIRSIGTVIGVSQPVVTIPGFDKFVFSDKSQNQC
KNSEQQMFCNQFCKCSHLSAEEITSADPTFSFPVYSAVYAIAHALHRVLQCGAGSCNRSITVHPHMVL
AELKKSNFTLLNQTIQFNEYGDPKFGSYSIVYWNAKGIPEEVGFFHFHPFMHFYINGTKVQWFTNGE
VPTSLCSTECQEGYIKTHNGIHQCCFTCEVCPNGTYVNSTDQVSCHHCEEHQWSAAGSTSCLPRTV
EYVAFTDTAAVVILVGAGLLLALTLAMCVLFAINYNTPVVRSAGGPMCFLILGCLSLCSISIFFFFDKPT
VAFCVLRFLPFVLFYTVCLACFVVRSFQIVSIFKIAAKFPQAHSWWMKYHGQWLVISVTFVIQAFLIIVS
LSSDPPSPFRDIVSYPDKIILGCFMNLKTSSGSTVLLLSLGSLCFIFSYMGKDLPKNYNEAKAITFCLLLL
ILTWIIFTTASLLYQGKYIHSLNALAVLSSIYSFLLWYFLPKCYIIIFQPQKNTQKYFQGLIQDYTKTISQ
>Danio_rerio_T1R2Ba
MLLDSNYIFLLGFINSLFFNFCSLASDFSLEGDFLLGGLFPLHEIDQVTPVFTPETTECFRYTASPSGFQ
MLQVMRFAVEEINNSSTLLPNVSLGYELFDHCSNTRNFGSLLSFISKNGSIKPKVKRINYQSDVIALTG
PYGSTRTISIAPLITMDLIPLVNYGASSSVLSNKLQYPSFVRTVPSNKDMINMIIHMIRWFGWNWVAFL
GSKDAYSTDGLNLFNTYINNTGICVAYQERLNLDANYSQTLKKIDMLNINVIVVFAVPQYAVNIINTAI
ADNIRDKVWIASETWSMNQQLPREPGVEKIGTVIGITDRLLTLPGFNEFILKDMGTANVNASKFQTN
TCNQNCDYCPSLTAEDIINENPSFSFAIYAAMYTIAHALHKVLQCDTKGCPKNTPFEPYMLLGEMKK
LDFPLNGRQVKYDKNYDPPISYAVVLWHTDENSPQFEMVGTYDTYPKTVFTIDNSRFPWRNDSIPFS
NCSVECKPGFARQPEGFHSCCFTCKKCPRNSYVDYSQDPYTCFPCAVSDWSDEGSTACKTRAVVYLE
FTEITSIAVMISVSFLIILLIGIFGLFAYNFNTPVVRSAGGGMCLLMLLCLTISSISVFFFFGKPSSVHCLVR
NAIFAFFFTVCLSCLTVRSFQIICVFKMAAQFPRLHSLWVKHNGQWLFIAFSSFIHLISCVIWTTVSPDI
PIADSWTFKDQTLLMCEMVNTITFTVVLFISWFLGFLCLVFSYMGRDLPKNYNEAKSITFSLILYYLSW
IVYFTAYLSIKSKYIMLLNAMAQISSIYGILFSYFIPKSYIMIFQPQKNTAAYFQTSIQNYTQTISRS
>Danio_rerio_T1R2Bb
MLPCYSFLLAFINVVSCLESEFSLKGDYLLGGLFPIHEGKPATHLFTPEAIECFRYTFSKSGYQMLQVM
RFAVEEINNSTTLLPDVSLGYEIFDHCSDTKNFPSVLSFISKNGSIKPKLKLNNYEPEVIALTGPYGSTT
TITIAPLITMDLIPLVNYGASSYSLSNKLMYPSFVRTVPSNKDLIHMIIQIIQWFGWNWVAFLGDQDDY
SEDGLRLFNTFISNSGICLAYQEALSQKTNYSLTFEMIDMLNVNVIVVFAEQQYASNIIKAAIANDVRD
KVWIASETWSMNQQLPREPGIEKIGTVIGITERFLSLPGFNEFIYKERRSVDDAGHNHGMGEVKSQTC
NQDCDCCTLLTAEEIITENPTFTFGIYSAIYTIAHALHKVLQCDVNVCDKNTTAKPYMLLEQIKKLDF
PLNDRQVKYDANGDPTISFAIILWHTETDPPHFDKVGMFDTYPEVTFTINNTLLPWHNNVPFSNCS
AECKEGFAREHDRFHTCCFLCKKCPRNSYVDYTRDPYTCFPCAESEWSDEGSTACKNRSVAYLQLTE
ASSITVLFSATCLMTVLIAIFVLFACNYNTPVVRSAGGSMCFLMLACLIMSTVSVFFFFGEPTFEHCILR
NVIFAYFFSVFLSCMAVRSFQIVCIFKMAAKFPSMHSLWVKHNGQWLFVGFFSIINIVSCVLYMTVSPP
KPFRDLVTFKDQLILSCEIGNTVTISMVMFIAWFLGFLCLVFSYMGRDLPKNYNEAKSITFSLILYYLS

WIVYFTTYLMLKSKYIQLVNAMTELSSIYGILFSYFIPKSYIIIFQPHKNTPAYFQTSIQSYTQTISRT
>Lepisosteus_oculatus_T1R2Ba
MQGSDFFFVLLGLYFLRINSTLCCEDSDFKLQGDYILGGLFQIHFALNEDTPSIHPEDLQCSRLNFLPS
GYQQLQVMRFAVEEINNSTSLLPNVSLGYEIFDYCSRTQNFPAVFDLLSTNGSVPVRNTIREYHPKIIS
VTGPFGSTKTITIAPLLMSSFVPMVTYGATSTQLSDKIKFPSFFRTVPSDKHQVGLIVRLLRMFKWNW
VAFIGGDSDYSRDALQFFSEEIPSANICLAYQGTVTRTAPEIDEMIDVIDDLQIGVIVLFAEQSDVTLFI
QQAVKRNVRNKVWIASEAWSLNQQLLSSAGIDSIGTVIGITIQGLGTLPGFKEFVSRTMTRRKTQTCP
SEQRVSPMETCNQLCPQCSGVTPQDIINEDPTYSFSIYSAVYAIAHSLHQALQCSVNGCSGSGPVYPY
MVFEKLKKVSFTLYGHTIEFDEYGDPSPYYEIVVWDKTSPELFKKIGSYSPQTQPEFILNKSLIHWHTN
GVVPVSVCSPECPLGYKKSQVGHHECCFECEACPNGTYVNYTADPSTCNKCEVDEWAEEGSSYCQK
RILVYLDYTELLSIGLMLSASFLISISAAVAIVFACSFNTPVVKSAGGKMCFFMLGCLTVSCCSVFFYLGI
PGREKCLLRNPVFVVFYTACLSCLAVRSFQIVCIFKMAAKLPKAYDFWVKHNGQWLTIMSCVGIQVIL
CAIWIGVEGPQPLNNTATYEDQIIFHCSMGNEVAFNIVIMFVGFLSIACFIFSYMGTDLPKNYNEAKSI
TFSLLIFFLSWISFLTAYMVHKGKYIQAINAISVLASLYGIVFGYFIPKCYIILYKPEKNTTAHFQTCIQNY
TQRISSM
>Amia_calva_T1R2Ba
MKSFGLFLLLGLCVPGAPAMASCASSDFRLQGDYILGGLFEVHNSLNYQSEHNRPEALQCYPLDFLT
SGYQQLQVMRFAVEEINNSSSLLPNVSLGYQIFDHCSDTLNFPSVFDFLSVNGSVPVKKKNRDYRPKV
ISVTGPFGSTQTITIAPLFMANLIPMINHGSSSARLSDKTVFPSFLRTVPSDRNQVQLIILLLQKFGWN
WVAFIGGNNDYSRDALQVFSEEIKSADICLAYQGTVLQKSPSIGAMLDQILQLKINVIVVFAPQEYAVA
LIETAIQNNVRDKVWIASDSWSLNPHLPRKEGIRAIGTVIGITSKSLSLPGLEEFIYRTMSRTDQGPCAK
TPSNASAQTCNQDCPSCAVVNPKVIVDEDPTYTFSIYSAVYAVATALHQSLGCGEKGCAGPGTVHPY
MVFEKLKKVNFTLHGRVVEFDGNGDPPAHYDVLFWDWTSSPLFKKIGSFSKQPRTSFFLNESLIRWH
TNRTVPVSVCSEECKVGYIRRVTGYHSCCFQCEVCSNGTYVNHTADLYTCKKCEDDEWSNHGSTSC
KKRSVEYLHFTEFVSIGLILSAGFLIIVSVAVSCVFACNYNTPVVKSAGGKMCFFMLSCLSVSCISVFAYI
GVPGWGSCIFRNPVFALFYTACISCLAVRSFQIVFIFKMAAKLPRAYDFWVKHNGQWLIIVTSVVFQIL
LCGIWIGTEGPHTFNNTSAYKDQIILICDMGNLYAFLTVIAFVGCLSVACFIFSYMGTDLPKNYNEAKS
ITFSLLIFFLAWISYFTAQIVYSGKYIQAINAVSVLASLYGILFGYFIPKCYIIIFTPEKNTPAHFQTCIQSYT
KTISSM
>Lepisosteus_oculatus_T1R2Bb
MRGFRFLIFALLYCRWINTSNPCKRKDFSLPGDYILGGLFRLHSAKEYKFLYRIPAVLDCDVCPFLPGY
YQQLQVMRFAVEEINNSTSLLPNVSLGYEIFDYCSDSHNLQAALDFLSKKLTRVIPVVDRITEYLPKVL
TVIGPFGTTESLVIAPLFGSYGVPMISPGASSLRLSDKINNPSFLRTIPSDKNQVRVIIQLLRRFNWNWV
AFVGSGDDYSNDALREFDKEARLVGICVPYQTTLPQDTSKIGALFDSIATVNVSVVVVFSTRIYAIPFIK
AAVANNVRDKVWIASEAWSMNEELARMPGIDTIGTVIGITTGGLAPLEGFEEFVRKTLAHRDGEICS
DSSRSAVEETCNQDCPACANVTLENLLSPDPSYRLRVYSSVYLAAHALHQLLGCDAKYCRRSDPVLP

HMVLKKLKTVNFTLYGRTIKFDVNGDPIPSYDFVVWDRTSPRFYKKIGSYTSDPEPIFVLNASLIRWFS
NGTVPVLTCSAECEAGYRRTSTGAHSCCFDCEPCLNGTFVNHTADPYKCVKCETDEWSENGSSCTK
RSQEYLHYTDLVSIGLFLSATILISLSIAVGAVFVWNRNTPVVKSAGGKMCFFMLGCLIVSCTTIFSFHG
VPHGWTCMLRFPVFTVFYTACLSCLTVRSFQIVFIFKMAAKLPKAYDFWVKHNGQWLTIMACVAIQI
ILCTIWIGVEGPRPFNNTVAYKDQIIFQCSLGNLYGLFTVTSFTAFLSILCFGFSYMGTDLPKNYNEAK
SITISLLVLFISWISYFTSRMVYQGKYLEVINAGSVLSTLYGILLCYFVPKCCIILFKPDQNTPAHFQSCIQ
NYTKTISTK
>Amia_calva_T1R2Bb
MQGFTFILSISLFFYPWINALKHCATKDFSLSGDYVLGGLFRIHIADQSNVSYRKPEVLQCSTWQFLPE
YYQQLQVLRFAVEEINNSTSLLPNVSLGYDLFDYCSDSLNLQVAYDFLSSSLDRSVPVLDRFLGYRPK
VISLIGPFGSTESLIIGPLFMFYDIPMISPGASSIRLSHEPFFNSFLRTIPSDLYQVRAIVQLLQRFKWNWV
AFIGSDNDXXXXXXXXRAGICVPYQNTIPQDPTGMLSIFRKIEALKVSVVVVFATQDLAVPFIQAAVAN
DIRDKVWIASEAWSTNQFLFKLPGIEAIGTVLGISISGLGPLPGFKEFVYRTIMQSDHEACAQTQRPVT
EEMCNQDCPECSSLTPEALLEADPTYSFNVYSAVYAVAHSLHQLLGCGANTCTRSGPVLPHMVLEG
LKKVDFMLKDRVINFDQNGDPTPYYDIVVWDRTSLEFPKRVGLYTSDPAPRFTLNESLIPWYTNGRV
PILICTAECEEGHRKLRTGVHDCCFQCEVCPNGTYIDKKADPYNCTKCEDDEWSGQGSTSCKKRTL
QYLHYTDHFSIALVVSTVIALALSIAITILFARNRDTPVVKSAGGTMCFFMLSCLSVACMGIFSYIGEPV
LVKCTLRNPVFAVFYTACLSCLAVRSFQIVFIFKMAAQLPKAYDFWVKHNGQWLVIVASVVIQIFLCGI
WIGTEGPQPFSDAAVYIDQIIVSCTMGGLRASLPVTLFVSCLSVACFIFSYMGTDLPKNYNEARSVTLS
LLIFFLSWISFFTAYIVYSGKYIQAFNAVSVLVSLYGILAGYFFPKCYIILFKPEQNTTAHFQTCIQSYTKSI
SSK
>Lepisosteus_oculatus_T1R2Bc
MKGFVILFNILPLSFLKANGGNVCGLSDFRLHGDYILGGLFKVHVDRKFNHVHSKPEAVQCREFDFS
ASGYQELQVMRFAVEEINNSTSLLPNISLGYEIYDYCSDFLNFMVAFDFLSTNFTGSLYVTKYFKDYQP
KVTALIGPFGSTENIILSPVFMAYLLPVISHGASSILLSDKQRYPSFLRTIPSDRNQVRAILQILHRFGWR
WIALIGGNDDYSRNGLQALKEEMSPYDICVAYESTIPTEGPAMGSLFSNITILKINVVIVFASKVSAVPFI
KAAISNKISDKVWIASETWSLNQQLLQDTQIHTIGTVIGITIKVVALPGLREFVHSTMTRGWNGTCVN
SPHIGTWETCNQVCSECSSTDPQSIADEDPMYSFSIYSAVYAVAHSLHQFLGCDAEGCRKSGTIIPYQL
LQTLKNVNFTLNNRTVSFDKNGDPAPNYGIVVWDKTLPLYHREIGSYVSDPSIAFHLDEELIRWHSN
GTVPTALCSLECKEGHRRKVSGVHTCCFDCEICPNGTYVNKTDDPYTCQSCEDDEWSDYGSTHCK
KRTLEYLHYNEGISIGILLLAGLIFLLSGAIAIVFARHHDTPVVKSAGGKMCFFMLSCLSVCSLSIFCYFG
WPQREKCTLRNPVFVVFYTACISCLAVRSFQIVCIFKMAAKLPKAYDFWVKNHGQWLVVATAIVLQIL
LCGVWIGSQGPRPFNNSALFKDQIIFDCNMGNLYTFVTVIAFVAALSVLCFMFSYMGTDLPKNYNEA
KCITFSLLIFFFSWILYLTAYMVYQGKYIAAINAFSVLASLYGILLGYFTPKCYIILFKPEHNTPAHFQNCI
QSYTKKISSE
>Amia_calva_T1R2Bc

MGELERSVTATQCLALLLCLVAVCGLTVNAREVCGASDFSLCGNYTLGALFQIHVARINTAYNRPEV
LQCHQYEFSPSGYQELQVLRFAVEEINNSTSLLPGVFLGYEVFDYCSNFLNLEAIIDYISVRSTGSVQVP
RYYIDYQPKVEAVIGTSVSSKNLVFTPIFAPYLLPVLSHGASSIRLSTRNLYPSHFRTIPSDKHQVQAILR
VLQKFKWNWVALIGDGNDYSNDGLQALKQQVDSFGICVAYEAIIPQEPAAVTNLFSNITFLRINTIVV
FASTESAVSFIQAAIAHNVSGKVWIGSETWSLNRQLLMDSKIQTIGTVIGITITIVPLPGFREFVLRSMA
QSSRVACINVAQEDRERCNQECLLCSKANPEDIAAEDPMYSFSIYSAVYAFAHSVHSLLGCDNNGCQR
SGQVLPYMIFSQLKTVKFDLLNYTVQFDENGDPPPNYEIVVWDKSMPQLYKTIGSYSADPQNPFILD
EKLIRWHTNGTVPISVCSVECKTGYRRKTAGVHQCCFDCEICANGTYLNITADPYTCKECREDEWAD
PGSTSCNKRTVEYLHYGETLSVLVLMSAGTLFTLSGAVALVFALHCNTPVVKSAGGNMCFFMLGCLS
LSGLSIFFYFEQPRWEKCILRNPMFAVFYTACLSCLAVRSFQIVCIFKMASKLPKAYDFWVKNHGQWL
AVVTITSLQALLCAVWIGTKAPQPFNNTRSYKDQIICECDIGNPYAFVFVIAFVALLSVLCFVFSYAGT
DLPKNYNEAKCITFSLLIFFFSWILYLTAYLIYKGKYISAINAGSVLASLYGILLGYFXXXXXXXXXXXXXX
XXXXXXXXXXXXXXXXXXXXXXXXHFQTCIQSYTRNISSM
>Polypterus_senegalus_T1R2B
MPGLQLLAIRVWISTFTALFVQNVCTKSEFKLEGDYILGGVFPVHYISQPDPEHFMPEVPQCQRYDVS
AQGYQQLQMMRFAVEDVNNQSWLLPNITLGYNIFDSCHIFVDIISVLNFLAMNTSHFINVSHRIEDYK
PQVLAVIGPYTSTEAFAMAPLLSSSFIPMVSHGASSIRLSDKKQFPSFLRMIPSDQYQARGITLLLHKFK
WKWIALIGSNDDYSQDGLEAIQKETAAFNICVAYQETIPRKTTEIHELFRNITMLKVSVVVVFASEDFA
VPFIKEAIAKNIRDKVWIASEAWSMNPLLLAEPGISSIGTILGMANKVISLPGFSDFVQRLNTGMKEQQ
PDPTTHPSLADEYCNQDCLNCCRTMASAILQENPTYSSYIYLAVYAVAHALHAVLGCNATHCERIVP
VMPYELLQELKKVHFSVNDYKLEFDSKGDPPPLYDIINWDFRSQPFKFTIVGSYSSDPKPVFLIHDHLI
QWHDNGTIPTSVCSSECQSGYRRLLKGIHDCCFNCELCPKGTYINITADPQRCQQCGPEEWSEKGST
SCLRRTLEFLDYSENVSIGLLFLTAVIFACLLAVIAIFAAHHNTPVVKSAGGPLCYFMLVCMATCCFSIV
SFFGKPNQVKCIMRNLVFSILYTSCIACLTVRSFQIICVFKMAAKLPRVYSFWVKSKSQCFFVMTCVCIQ
ISLCTLWVGIEGPSPYNNTVSFKNQIIFSCHMGNISIFTAVLIFIGFLGSLCFVFSYMGIGLPKNYNEAKC
ITFSLLISFFSWISYFTAYMIYYEKYISAFNVLSVLVSLLGILIGYFIPKCYIILIKPEHNTTAHFQSCIQTYT
MKSTVTSTS
>Homo_sapiens_T1R2A
MGPRAKTISSLFFLLWVLAEPAENSDFYLPGDYLLGGLFSLHANMKGIVHLNFLQVPMCKEYEVKVIG
YNLMQAMRFAVEEINNDSSLLPGVLLGYEIVDVCYISNNVQPVLYFLAHEDNLLPIQEDYSNYISRVVA
VIGPDNSESVMTVANFLSLFLLPQITYSAISDELRDKVRFPALLRTTPSADHHIEAMVQLMLHFRWNW
IIVLVSSDTYGRDNGQLLGERVARRDICIAFQETLPTLQPNQNMTSEERQRLVTIVDKLQQSTARVVV
VFSPDLTLYHFFNEVLRQNFTGAVWIASESWAIDPVLHNLTELRHLGTFLGITIQSVPIPGFSEFREWG
PQAGPPPLSRTSQSYTCNQECDNCLNATLSFNTILRLSGERVVYSVYSAVYAVAHALHSLLGCDKSTC
TKRVVYPWQLLEEIWKVNFTLLDHQIFFDPQGDVALHLEIVQWQWDRSQNPFQSVASYYPLQRQLK
NIQDISWHTINNTIPMSMCSKRCQSGQKKKPVGIHVCCFECIDCLPGTFLNHTEDEYECQACPNNE

WSYQSETSCFKRQLVFLEWHEAPTIAVALLAALGFLSTLAILVIFWRHFQTPIVRSAGGPMCFLMLTLL
LVAYMVVPVYVGPPKVSTCLCRQALFPLCFTICISCIAVRSFQIVCAFKMASRFPRAYSYWVRYQGPYV
SMAFITVLKMVIVVIGMLATGLSPTTRTDPDDPKITIVSCNPNYRNSLLFNTSLDLLLSVVGFSFAYMG
KELPTNYNEAKFITLSMTFYFTSSVSLCTFMSAYSGVLVTIVDLLVTVLNLLAISLGYFGPKCYMILFYP
ERNTPAYFNSMIQGYTMRRD
>Mus_musculus_T1R2A
MGPQARTLHLLFLLLHALPKPVMLVGNSDFHLAGDYLLGGLFTLHANVKSVSHLSYLQVPKCNEYN
MKVLGYNLMQAMRFAVEEINNCSSLLPGVLLGYEMVDVCYLSNNIQPGLYFLSQIDDFLPILKDYSQ
YRPQVVAVIGPDNSESAITVSNILSYFLVPQVTYSAITDKLRDKRRFPAMLRTVPSATHHIEAMVQLM
VHFQWNWIVVLVSDDDYGRENSHLLSQRLTNTGDICIAFQEVLPVPEPNQAVRPEEQDQLDNILDK
LRRTSARVVVIFSPELSLHNFFREVLRWNFTGFVWIASESWAIDPVLHNLTELRHTGTFLGVTIQRVSI
PGFSQFRVRHDKPEYPMPNETSLRTTCNQDCDACMNITESFNNVLMLSGERVVYSVYSAVYAVAHT
LHRLLHCNQVRCTKQIVYPWQLLREIWHVNFTLLGNQLFFDEQGDMPMLLDIIQWQWGLSQNPF
QSIASYSPTETRLTYISNVSWYTPNNTVPISMCSKSCQPGQMKKPIGLHPCCFECVDCPPGTYLNRSV
DEFNCLSCPGSMWSYKNNIACFKRRLAFLEWHEVPTIVVTILAALGFISTLAILLIFWRHFQTPMVRSA
GGPMCFLMLVPLLLAFGMVPVYVGPPTVFSCFCRQAFFTVCFSVCLSCITVRSFQIVCVFKMARRLPS
AYGFWMRYHGPYVFVAFITAVKVALVAGNMLATTINPIGRTDPDDPNIIILSCHPNYRNGLLFNTSM
DLLLSVLGFSFAYVGKELPTNYNEAKFITLSMTFSFTSSISLCTFMSVHDGVLVTIMDLLVTVLNFLAI
GLGYFGPKCYMILFYPERNTSAYFNSMIQGYTMRKS
>Gekko_japonicus_T1R2A
MRLASVVAACLLGALVAAGSFNGTPPSHFHLAGDYILGGLFSLHAEVVGKAHLSSSLVPICKEYKLKG
VSYSYLQAMRFAVEEINNSSALLPGISLGYEMVDVCYLTNTIHPVLYFLSDNRSQLEIQTNYTHYHPRV
LAVIGPDSSSAAVAAAHLLSLFLVPQVTYSATSQALSNPKVFPVAFRTIPSSEQQITVILRLLRRFRWNW
VIVLSSDDDYGQQNMQALRAQASWPCIAFQEIIPVQTTNQEDRGIRQRMEGIVRKIVGSTAKVVIVLT
LELPLPAFFQEVLLQNVTGLVWIACEAWAIDPSLHSIANISSIGTIFGVAAQDVLMPGFADFRVRSPSSP
AEGARERDAAETCNQVCDQCLPKMLLYDKILRGTGNRIDFNVYSAVYVVAHALHQLLDCNSRKGCR
KQKVYPWQLLQQVAQVKFTLRSTTIRFDKNGDPPSGFEVIQWQWGTPGFPFKQIAAYDPVKKELDV
FTGAITWHTPNNTAPRSVCSEQCEPGHKKKLIGSMACCFECVPCEAGTFFNTSDNFICQKCPPDMW
SDPGQEHCFKRLVKYLEWDDLTSILLIFCSASGLSMTLGILALFLRYSETPVVKSAGGRLCFLVIATLSV
GFCSIPFYMGVPTEAKCLCRQTISSLCFTVCVSCITVRSFQIICAFKMASRLPAAYDFWMKHHGQQVFI
GVVSAAKFVLVVATVYIHHPPLAEPTVSSDPAVMNLMCNTNYKSNMIGHNIFDMVLCFLCFCFAYIG
KALPKNYNEAKYITLCMTCYFSTWVALCLVRSLFEGMVVTVFDAGTVLTNLLSITVGYFGPKCYVILF
HPERNTAAFFQTAIQSYTMRHD
>Pogona_vitticeps_T1R2A
MGAAAAGGALVIACLGAWIARAAPLATPSSHFHLAGDYILGGLFSLHMEASTHPYFSQSWVADCKKY
PTKAMGYSYLQAMRYAVEQINNSSLLLPGISLGYEMVDICYHSNAVHPLLFFLSDNRSLLEIQASNTFY

RPRVMAVIGPDVSSTAVTAAYLLSNFFVPQISYSATMESLSNPRTFPAAFRTIPSTEQQIVILLNLLRSFR
WNWVIVLYSEDEYGQTNLHQLRAQATWACVAFQESIPFWTNDRPALAKARIQNLVERIKGSSAKVVI
VLSTEVPLWRFFEEAVWQNVTGFVWIAAEAWAMDSMLRDLPGISRLGTFIGIAPEKVAIPGLDDFRV
RPSGDPPDGAEARADPAMCNQECDDCLFKAQEMDPILRATGSRIEFNVYSAVYAVAHALHRVLGCN
ATGCRHRTVYPWQLLKEMSQVRFSLLNITINFDEKGDPPNGFEVLEWLWDTPGKPFQRIASYSELNE
LHIDAESILWHTENNTVPKSVCSEQCEPGQMKKHLASLSCCFECVECKPGTFLNQSSIFSCQSCPPDM
WSHAGQEECFLRSVRYLQWDSPVAIFLLFFTFLGLLTAGGLLLIFVRHANTPVVRSAGGPLCFLMIAS
LIVGFCSLFFYIGQPTEVKCICRRMVFSLCFTICMACMSVRSFQIMCVFKMASRLPRAYNAWVRYNGQ
WIFVATAFTLKVVIVGMNLYFFPPLPLQSAVSSSDPAVLILTCNPNYLSAIVFNNVLEMVLSFACFCFAY
TGKALPKNYNEAKYITLCMTSYFTCWVVLVVVMAVCEGVVVTAFDVVTGLLNLLSVCIGYFGPKCYV
ILFRPEQNTLAFFQTAIQSYTMRPA
>Anolis_carolinensis_T1R2A
MGLAAVAVAACLGALVALGSSGGTPRFSNFRLDGDYLLGGLFSLHAEAVGTPFLSQAMVPICKNYRL
RTIGFSYLQAMRFAVEEINNSSALLPGLSLGYEIFDVCYLTNIIQPLFSFLSDDHLRVEIKRNYSDYKTRV
VAVIGPDAPSSAVTAAYLLSQFLVPQITYSATTEALSSPKIFPVAFRTIPSTEQQVALLLNLMKYFRWN
WVIMLSSEDDYGQQNAQLLRSKIGTTYSEASWICVAFQETIPVQNDSQEEPDGAALLRFQLIIAKIKRS
SAKVVIVLSTELSLRPFFGEVIRQNLTGLVWVASEAWATDLSIFAMHNISSIGSVFGVAVQDVPIPGLD
EFRVRSPEVREAAPPMCNQECDDCLSAVRHYDASLRSSGNRIDFNIYSSVYVVAHALHTLLGCNKTG
CSKRTVYPWQLLREVGRVNFTLLNNIIQFDEKGDPPNGFEIVQWQWDIPGKPFRRIANYKFPQEELL
VYTENIVWHTENSTPPRSVCSAQCGQGQRRKVLGSSACCFVCVECEAGTFLNKSDIFTCQTCPHNM
WSNSGQEECFTKTVIFFSWHDPVSPVLLSFAFLGLLATLGILLTFICHHDTPVVRSAGGRLCFLMLAA
LAAGFCSVGFYIGELSEFKCVGRRSAFGLCFSLCIACITVRSFQIICVFKVAARLPRAYDLWRKYHGQEV
FVGTVLALKMTIMGCNVYFYPPVPALTTMRNNPAVLLLMCTPDFKASMVLSNSLEMFLCLLCFSFAY
MGKAMPKNYNEARYVTLSMTGYFSCWATLFLVMSLCDGLVVNKVDTSVMLTNLFNICLGYFGPKC
YIIFFHPERNTAAFFQTDIQSYTMRQD
>Ambystoma_mexicanum_T1R2A
MLAFLFLLNLAPQFLDSSDTGSGSDLVSLGDYNIGGLFTFHAPAASVPPRTTPVVENCASLQINLPGF
HYLQAMRFAIQEINNSSALLPGITLGYTSYDSCYIYNNIQPALDFISRDGTIDIRSHYATYMPRVIAVIGP
DNIDAAVATSNIFNVFLLPQINYYAPIKALSGLDLPSCFQTIPSVAKQQKAIQHILTAFKWTWIAALGT
ADDYGKEGLQELYNAAPSAGICIGYQAIIPKKEKAYEIEWEQDIIRIVKNITTANVNVIVVFGLDIIAVDF
FKFVVNTNLTNKVWIATEAWSVAKNIYNIPNISRLGVVFGIAIEHVEIPGFNEYLKNLIKDYQSGAISPA
KGSCNQDCSDCLNATVEDILGPPESRVCFNVYSAVYAIAHALHETLACTQNQCTKRTIYPWQVTEAL
KHLNFSLINRSIRFDRFGDSAAGFDIVFWDWNASDGGGFLNVGAYKSTGHLQINAQKIKWNTINNTI
PVSQCSPECSTGQEKIQLGVHTCCFTCYSCAPGTYLHLNGSCAPCSADQWSLERSVLCYNKTKEFLE
WTSPLAIPLVAGVVLGMLLTFVMMVAFAINFQSPVVKAAGGRMTFIMLAALATAYISVLAFIGQPTVL
RCILRHPIYSTALAVCFSYIAVKSFQIVCIFKMAATLPKTYDYWVKRNGQYVCVWLLSGVQLVISCGW

VIYSPPEVIWKSINRNRMMMDCSEFSSAGHILQFSYNGLLSMLCFVFSYMGKELPKNYSEARCITFAML
VYFAVCVSFFTLQIIQVPEYITPINATLALVSLFGIMGGYFFPKCYVIFWKPEHNTAQHFQTAIQSYTKR
KSGSSRR
>Nanorana_parkeri_T1R2A
MWHRIFVSGLVVIFTHVGSICEENVESEFIYKGDYNLGGLFSLHASGADWSSHYTPVVESCSSLSVNLA
GYLYFQVMRFAVKEINNSSSLLPTITVGYNVFDTCYVYNNIHPALTFISRDHIIDTEESYTSYIPIVIAVIG
PDNSDAAETTADLFNLLHLPQINVFATSKRMSELSLPACFQTIPSSRVQHQAFVDILSYFNWTWIAVL
GSFDEYGMDGIQQFVKATSDLNICLAYKDFIPIKVSGNEAMWKSTIRQIANNITFTNVNVILLFSLDIV
LVDFFKELVDMDFPPKIWLATETWSLSDDIYNIPNINTLGVIFGITLKYVKIPGFDEFLTNVYYQNKNS
SGGSRLEENCNQNCDSCLNTTLSEFLSYSAKRSSFSIYAAVYAVAHALHDVLGCNKTYCDKKDVYPW
QVTEALNRVNFLLLNNEISFNVYGDSPTGYDIVFWNWLDGTPFLNVGSYTILEKLDINHQKIIWQTS
NNVPSSVCSSECLPGQKKQPKGHYKCCFACISCSAGTYLHTNGTCVPCREDQWSTEKSTICLDKTRIF
MTWNNAITISLFTVTILGILLNIVIMGTFFAKLSSPVVKAAGGKMCFLMLSSLTVSYLSILSYLGEPGTL
KCIMRLPIYSIALTVSFSYISIRSFQIVCIFKMSSKLPATYDYWVKQNGQYICQAFLSGVQVFISVIWIITN
PPKATTKELSSDQVLVECSQFSSVYNILQYSYNAILSLLCFTFSYMGKDLPKNYSEAKCITFAMLIFFVV
CISFFTAQLIDVGEHITAINAGLAWLSLMGLTGGYFFPKCYIIFCRPQFNTTKYFQSTIQSYTKRGSGSS
K
>Rhinatrema_bivittatum_T1R2A
MRSFPLFIYVTTSLLAASEQFALSEFAAQGDYTIGGLFTFHASGAGTAFRETPEVEHCASFRINIPGYRH
FQAMKFAVQEINNSSKLLPNITLGFEVFDACYKYNSIQPALDFLSRDRLINIQESYTSYTPKVIAVIGPD
SSDAAITTGNIFNLLLIPQMNYYAISSVLSNMKLPSCFQTVPNYKKQKHVIVDILLRFNWTWIAILGSG
DDYGQEGLQQLYDAASAEGICVAYQGLIPGKIPDRKKEWEEEVSLIIQNITMTKVGVIVVFALDVIAID
FFQAVVDTDLPRKVWIASETWSVANIIYDLPNAHRLGVVVGIAMKHVEIPGFTEHLVHLVNHSRGGP
VKVAKENCNQNCDSCLEATLSELAGPPERRVIFNVYSAVYAVAYALHDVLGCNHTHCNKRAVYPWQ
VTEALKRVNFSLLNNILNFDQYGDSPAGFDIVFWNWTGRTPFVKVGSYSNFGNLQIQADLITWNTQ
NNTVPPSICSQKCLAGQEKKPKGAHKCCFICVNCAEGTFLNDNGTCTNCGADQWSPERSISCFNKT
RIFIEWNGKAAIVLLTAAAVGILFTLITIVAFALQIQSPVVKAAGGKMCFFMLSALSLAYLCIFSFIGEPSS
LKCILRHPTINTALTVCFSYIAVKSFQIVCIFKMAAKLPKAYDYWVKKNGQYVVVFLLTSIQLFISCFWV
SLRPPSVSKTNFDRDKLLLECNEVSSVMNVLEFAYNSLLSLLCFAFAYVGKGLPKNYSEAKCITFAMLI
YFMVCISFFTAHLIDVSYYITAINAILALVSLFGITGGYFLPKCYVILWKPEYNTTQYFQTAIQSYTRRKS
TLSSR
>Latimeria_chalumnae_T1R2Ab
MLFVTVLSVYAITQCAGIHLTNVSTEFRLPGDYILGGLFTLHASSDRTMTTSLGHLNCQNYDVYLPG
YHYLQGLRYAVEEINNSSVLLPDVSLGYEIFDTCFSSIINPTLRFLTANASRRVEVREKVMEYHPRVIAVI
GPDSSTAAMTSAEIFSSFLMPQISYYATTDALSDKHYPSFLRTIPPSKYLIEAHLSLLLEFGWTWIAVLG
TDDEYGNEGIQSLYEAATTQGICVAYQRAMPLQGEQPAASKRIMTEVIDNIIRNRVNVVVVFSFDIFL

SSFLKEVIARNVTGKVWLASEGWAASEVISKLPNIKSVGTVLGTAVGYTKIPGFLEFIKKELLLSQQLNL
SVPSRDGSNGSSDDACNQACDQCQLLTPSELLTSLDRRVTFNVYSAVYAVALALHQVLDCASGTCSR
LKVYPWQMLDQIKQVNFTILDHPIYFDENGDLPIGFEIVFWDWSEGNVSFKKAGMYHPNLRGLEIDR
SLIKWNTQDNMIPKSVCSEECASGHWRKQTGLHPCCFDCMECEAGTFLNKSDLTDCQKCGITQWS
PAGSETCHDRRIEVLEWDAAAAILLVIITTVGLALTVAVLVIFMLNFETPVVKSAGGTMCFIMLLQLTI
SYCSVFSFVGKPTTTSCIVRSVLRTSFVISLSCLMVRSFQIVCIFKMAAKLPKAYDYWVKYNGQYIFVSV
CFGIQVLICASEIFVASPRPKPIYEANCPVIIMVCSNFTDAAEILRAAIGIFISFMCLLFVDMGKELPANY
NEARCITLVVVLNLISQLSILFIKTLHYGQLLILFEAAANVITFYGLIVGYFFPKCYVILFKPDQNTTAYF
QMTIQEYTRRRSNHFH
>Latimeria_chalumnae_T1R2Aa
MPYVAFFSLCCFIRLMGTTLPHVPTKFQLPGDYVLGGLVTVHANSTGGTPVSRLKIFNCEDLQIYYP
GYRFLQGLRYAVEEINNSSILLPDVLLGYEIFDTCFGDAINPILHFLTTNKSRWVEVKDSSVEYQPKVIA
VIGPDSSSTALISAEILSSFLIPQISYYVTAQGLNDITRYPSFFRLIPSAQYQIQAIILLLKEFGWKWIAVISS
DDDYGIQAAESLADAISKEEICIAYSEVIPLVQEESKPTNSLKAKLAEIVDGIVKSQANLIIVFSFDAFLSP
FFEEVVAKNVTGKVWLASEYWAVSESVSSVSNIKSIGTVLGTAVRHIEMPGYVDYILKETEEHSELRAM
VKEQESPKDTCNQDCTKCYSVTTQQLLNPPERRITFTIYAAVYSVAHALHNLLGCESGTCKKLKVYP
WQVLQKMKRVNFSLLGNPIYFDEDGNPPTGFDIVFWDWSKGDVSFKKVGIYSPNSDHIINRDLVYW
NAPENKVPVSVCSEECAPGHWKKLSETRSCCFSCIKCEKDTFLNKSDLTTCQKCDFTQWSPIGSEVC
YDRHIEYLDWPDASAIVLLSVTAVGLMVTVAVMLIFVLNFQTPVVKSAGRIMCFIMLLNLAFAYCSMT
TFVGKPTTATCIFRKSVFSIILVTCFSCLTVRSFQIICIFKMAAKLPKAYDYWVKYHGQYITVFIIVLVKIV
ITFAWIATDPPGYIPYKTYDQHLVLLECSTPSPTGTAMNMAIDFSSSIFCFIFAYIGKELPQNYNEAKCI
TFSVMIYYISWIAYVTIAAVYYGDLIITIEYVASLASLLGITIGYFVPKCYVVVFRPDHNTTAYFQTTIHC
YTNRRSDRSH
>Amia_calva_T1R2A
MWGLPLCCLCLGLVDSSMLEDIWVESKGDYYIGGIFPVEHLDKNLDKCDMLPGFVMAEAMRFAMQ
EINNSTTLLPNVRLGYRMLDSCSDAWGIHSMLHFLSPQDTTVLDLQRNFSELQPNAVAIIGQGMKPL
DDAMTGLHQIVQVPVISYYSVPLTLMARYRTLFSVVPAMENQLLAIQALLLDLGWRWVAIVGSKTNY
GQENIRTLYSQVTGEDICVAYFGTINDTEVGPIISKIVDTRVNVTVILAEPETVRAFFAGVMEQNVTG
KVWVASSSWSSAFQEVSVPSSIGTVLGVGEWGTSLPGFQDFLQASLPNQPPLSTTASPSDCQGSDAG
KILNMTGTQGAFSVYAAVHSAAQALHFALGCQRSACDPGPVYPKEVMEAVERINFSIGDHVIKFGVS
GTMEARLSLVSWVWKSDGTVSATTVGHYHTGSKDLQLHKGEIAWGGVLGSKVPMSVCSQECRSGE
RKILGTHHCCFLCDPCPWGTYLNYTDEYSCKTCPAGQWAPPASEACLQRSTEYLDWNSVAGLALM
CVVSTSLLLNVAIAALFLLHWQSPMVRAAGGCMSLAMLVSLIVAYSSILAYSFRRPTQVSCAVGITLFSI
CVSVSLSCMVVKSIQILCIFKYSTRLPHFYSYWVKNHGPPLTVLGLTLLATLIQGAQLLCLPPKVMEAE
DTFSNATILMCNQDQQWADSIFKLLIAMLAFSLSYMGKGLPRAYSEVKYIAFSSLIFIVGWITYLTTHF
MESGTISAALQVAGMLVSLMGISAGYFLQRCFIILFHPEQNTTAYFQSMIQEYTMSGH

>Polypterus_senegalus_T1R2A
MLLPLCWMILMVPPSAGRFLAVSPGDYIIGGIFPFSGQLFLNYQCNWRNVQQFLMVEAMRFAVDEIN
NSSNILPNITLGYQLLDNCSMEETFHSILHLLSDTEVDVLYVWHDPSKYLPTVLAIIGPGDSTAATAIM
GLPSWYWVPMIDFFDSVQMLANKRLYPSYFCTVPSTLEQTIAILGLMQQFSWTWVALVGSSTDYGQ
ESMQLFLEMSATYGVCVPYFSTITTSPQDTTRILGEIMAVNVTVIFAEDTVVESFFQVAMNQSIEGKV
WVASNTWSVSTRVAAVSGILKTGTVLGIAVKERPMPGFEEYLSKRFQSPTPHTGSNSPMTSCIGLTAK
QILSYTGTRVSYGVYAAVHAASEALNLALGCKPNLCERKRIYPWQVLNAMKTMNFSISNITGDPNRL
VESEYDIITWHAKAQLPSVTIVGHYSRSQGVKLNKTLIGWGTTGQQEPFSVCSEECGLTARKERGSH
MCCFQCIPCAPGMFANKSNPYTCQSCNVNEWWNPFEKRCLRREVEFLRWSDPTGMLLACVSWLAVI
LTLGIIVLFLWYQDTPVVKATGGRMSLVMLVCLTVAYLNISLYIGKPFNLVCLIRHPIYAITSSICLSCM
AVKTFQLVCIFKLARRLPSAYNYWAHHSGPVFTIVGFTAVDIVIQVTHSILTHPKATADTSSFPDLILL
NCSHSLDLLDIFYKTLLSLLAFLLAYLSKDLPPSYSEVKCIAFSALIFLVGWGFYLTVHALELGKMSSVL
KVLSILLSLLGITAGYFLPRCYIILIQPERNTATFFQNMIHEYTMGS
>Ambystoma_mexicanum_T1R5
MAFSRGKSVVALTALWALFPVSVADGLFLDASTEFSRPGDYILGGLFPIHFGSRVYSAPYRPDAMHCE
GMPLNLQGYRHFQAFRFAIEEIDASGSVLPNITLGYDVYDDCHEQAGIMATLGFLASPSHLLVNGEV
DGSRPPQQVVAVIGPTVSDTAVITATLLGSFNLPQVSYSAASEALSDKKNFPSFLRTLPSDRLQAEAMI
ALLKAFGWTWIAVFGSSTEYGQRGKQVLIRLAALHAICVAYHQTVTHGVDEASNALAMVQMLRANV
IFVFGETSFALEFFKAVAQSNLTGRVWIASTVWATDRSITEIPNLPNIGTVLGMAPLPGSMPGFEEFLA
KEVGKYHWTSSAPGGTVASCTQKCEQCQTLLYGNVVDIPEKRASYRTYAAVHAVARALHKLLQCKL
TGCVPRDLYPSQLLGILQDIDFSLHGQRVHFDGNGDAPTGYDIVAWNWNDGRASFDTIGQYTVEP
QRLHVNSSLVTWHTPDGMVPGSICSEECAPGQRRKQAGIHFCCFMCLMCPEGTFANRSDQYGCQP
CQRDQWSPVGSPLCFQRTAIYLTWDSKVSLGVLLASSAGLVLVFVVSAVFYRCLTTPVVKAAGGKLC
FLMLSMLAMSYSSMCCFLGKPTQLICVLRQLLFPLGFTVFLTCLLIRSFQIVCIFKMVTKIPKSFAYWM
KPKGQCLLLASCFAFQALLSGVWVAMGAHQPRQDYRFRDAIILKCGQDPSLAVATQDAFPGFLSSLC
FLFSFLGKDFPKTYNEAKSITITVVIYFFTVVCFFAVNVGENVKHSTLLLALCILARLYGVLSTFFLPKCY
ILIFRPKQNTPGYFQNCIQNYTLGGPT
>Rhinatrema_bivittatum_T1R5
MASLAAVLASLGTLLRQPACCLGWKLQGEFRLPGDYTLGALFPIHFGTKTFHPPYRPEAMHCEGLPF
NLQGYRHFQAMRFAIEEINNSSSLLPNVSLGYEIYDDCYESAGIMATFSFLTAPPLPYVKVGEDAGKYC
PRVLAVIGPTVSDTALITSNILSSYHLPQVSYSAASEALSNKRLFPSFLRTLPSDLLQAEAMIALLQEFSW
TWIVVIGSNTEYSRKGKQVLMKLAAGRRICVAYHRTLTQVATDTPQVLDTVARKNANVVLVFGEAS
FAFDFFSTVAASNVTGKVWIASSVWATDRRVSEIPNLRQMGTVLGVTALPGSMPGFEQFVSREVSKY
SQTGSAGGSIAASCNQMCERCQSLAAADVLDIPERRTSFRTYAAVHALARALHRTLNCDSGACTEAA
LCPWQLLEPLRQVNFSLHGRRIHFDANGDMPSGYDIVLWTWSHGRASFQTIGVFSAQSRQLEVDSG
EIPWHTADRMVPVSVCSEDCQPGQRRKQTGTHFCCFLCLTCPEGTYVNQSYPYECQPCQRDQWSH

KGSPVCYQRALHYLLWDSSISIVLMTFSSLGLLVLLMVSIIFYRNLDTPVVKAAGGRLCFLMLALLTVC
YCSVYCFLGRPTTLSCILRQLLFPLSFTAFLFCLLFRSFQIVCIFKMASRLPKSLSYCMKPNIQNLLLGIG
CLLQALLCCGCFAMGSHLPWLDYRFREAIILKCGQQLLLAEITQEAFPCLLSCLCFIFSFVGKDLPKNY
NEAKCITVAVIIYFFTLVCYFSVHVGENERYSTVLQAACFLVRLHGVLAGYFLPKCYIILFNPKQNTTSY
FQTCIQNYSISSSRT
>Neoceratodus_forsteri_T1R5a
MISLLLLVILLHRMLFAARHLNNSFDEFNQPGDFIIGGLFPIHWASKNVTLPYKPQRLSCEGFPFYFRG
YRHLQGLKYAIEEINNSSSLLPNVTLGYEAFDDCFEAAGVMAAFDFLSSHSQPYVKILRNYSTYQPKVI
AVIGPTVSDTAVITSSILSNFLVPQISYSAASGALSNKRIFPSFLRTFLSDQKQAEAMILLLKKFQWTWVI
VIGSNSEYGRRGKQQLVVLAAANGICVAYDRTITQGSSTIKDEIAHVTYMISRRNVNVILVFGEDLFAA
AFFKEVIESNITSKVWIASSVWAIDRKVTEIPNIWKIGTVLGIAPQHGIMPGLKQFVSKAVGLKSKYQAS
FEALNKWEGAAGSCNQICDECNSLTAEAILDSPEIRAVFRTYSAVYVVAHALHKLLNCDSGLCKEMTF
FPWQLLEKMKQVNFSLHGKWIYFDQNGDAPTGYDIIMWNWTNRRVSFDTVGIYSSNPGRIDLDTD
LISWHTGNKTIPTSVCSEECLPGQKRKQAGPHFCCFFCLTCPEGTYLNSSNRYECQPCHKDMWSPE
GSQVCFPRTLEYLAWSNEVSILLIVLSAAGLFLTGVIMIVFFRNLDTPVVKAAGGKACFLMLTFLMFCY
CSVYSFIGKPTEVTCILRQLMFPLSFTAFLSCLVLRSFLIVCIFKMAARLPKSFDYWVKRRGQSLFLGVS
TVIQIIISSFWITTGTYKPILDYTFKEVIIFKCGKNQSLALITQDAFTGLLSILCFVFSYMGKDLPKNYNE
AKCITVTILIYFFSLASYFLFNAAQNERYSTILLVVCVLARLYGILGGYFFPKFYIITFRPKQNTTGYFQSC
IQNYTNRRSSLTS
>Neoceratodus_forsteri_T1R5b
MVCCLLFLVLQIHCLLFSIRSMNIDDYEFMLQGKYVLGGLFPVHFATKFPTPPYRPEPPNCNGLPFNA
QGYRHLQAMRYAIEEINNSSFLLPNVTLGYEIFDDCYEASGIMATFNFLSQRLETHVKIQNDYIKYQPK
VIAVIGPTVSDTAVITSSILSSFLVPQISYSAASEVLSNKRIFPSFLRTFPSDRKQAEAIIMLLKRFQWNWI
AVIGSNTEYSRRGKQQLVELAIVNEICVAHDKTITQIGAQMRNDVAQIAHLIIRRNVNVILVFGEVKFA
FTFFKEITELNITAKVWIASSVWATDRKISELPNLRKIGTILGIAPLPGDMPGFERFVSNAVYNVKYSKH
LSATQLNGSVAGSCNQLCEECLSLTAEDIINIPERRATFRTYSAVYAVAHSLHQLLDCESGTCKNETVF
PWQLLAKVKHIHFHLQGTPVFFDEHGDSPTGYDIVMWNWTNEKASFDTIGMYTPNPGRINLDANFI
KWHTENNKIPVSVCSEECLPGQKRKQAGPHFCCFFCMTCPEGTYLNQSNPYNCQPCHKELWSPEGS
QVCFPRTLEYLAWSNEVSIFVTVLSVVGFFLTGVIGMIFFRNLNTPVVKAAGGKVCFLMLILLMFCYC
SVCCFIGKPTEVTCILRQLVFPICFTAFLSCLVLRSFLLVCIFKMAARLPKSFDYWVKQHGQSLLLGVC
TVTQVISSSFWIATGTCKPMLDYTFKEAIILKCGKNQSLALITQDAFTGLLSTFCFVFSYMGKDLPKNY
NEAKCITITILIYFFSLASYFLFNVAQNERYSNMLLVICILARLYGVIGGYFFPKCFIIVFKPKQNTTEYFQ
TCIQNYSNKRSSHTNEH
>Protopterus_annectens_T1R5a
MLFVYLIIHLYCTSFFLHCLNNNVEEFKLPGDYIIGGLFPVHWATKNFTHPYKPQRLSCEGFPFYFRGY
RHLQGMRYAIEEINNSSSLLPNITLGYEAFDDCFEPAGVMAIFDFLASHSQPFVKVLSNYSAYKPRVIA

VIGPTVSDTAVVTASILSNFLVPQVSYSAASEVLSNKRVFPSFLRTFLSDQKQAQAMILLLKTFHWNW
VIVIGSNSEYGRRGKQQLVTLASAYGICVAYDKTLNQGSSTFKSDIADIIYVISKQNVNVILLFAEDLFA
AAFFKEVIPSNVTSKVWIASSVWAIDRRVTEIPNIWKIGTVLGIAPQHGDMPGLKQFLSKFVGQKPSEY
NASFSASEEWYDVSGNCNHICEECSSLTPEAVLDSPEIRAVFRTYSAVYAMAYALHNLLDCDTDLCKE
TNIFPWQLLEKIKQVNFTLHGKQIYFDQNGDAPTGYDIIMWNWTNSRASFDTVGTYTSIPGRIDVNT
ALITWYTGDKMVPKSVCSEECLPGQKRKQAGPHFCCFFCLTCPEGTYLNSTDPYECLPCLKDMWSP
EGSQVCFPRTLEYLEWNNEISVLLMILSATGLFLTGLTMLVFLRNLDTPVVKAAGGKACLLMLTFIIF
CYCSVFSFIGKPNETACVVRQLMFPISFTAFMSCLVLRSFLIVCIFKMASSLPKSFDYWVKQHGQSLFL
AVGTATQMTISSFWLTKGTFKPALDYTFKEAIILKCGTNQSLALLTQDAFTGFLSIICFVFSYMGKDLP
KNYSEAKCITVTILVYFFSLASYFLFNVAQNERYSTILLAAIILVRLYGILGGYFFPKFYIIAVRPQKNTTA
YFQSCIQNYTNSSLTN
>Protopterus_annectens_T1R5X
MSLFCLIWCLLFPGFLLPRLTVAFSIPEKDFFLQGDFSIGGLFSLHENADLQTLPRIPEVENCNKYSFYS
PGYVSFQTMRFAIEEINNSSSFLPNVTLGYKIRDVCSNTGATQATLALLSSKLNNSIQLQDNYTNYLP
QVMAIIGPDSSDTAASSARVTSFFLVPQISYQATSMTLSDRKSFPSFFRTIPSDENQVKAMVALLRFFQ
WSWVSVIGSDTEYGQSGMKSIINVADGQGICVPFSETLPSDHNFTEQQIKHITDGISSKGIKVVIVFGE
DVFAATFFRSVMKTNLTGIVWIASEAWSGAPAVKQLSGIERIGKVIGLSIRQVQIIGFQQFLDNKIKESS
WKTTPTTALGVSRSSIVWKSTPATTKAEVTDQSIACNQACEDCRIVTTTSQLLGNSETVAFNVYLAIY
SVAHSLHRLLACGSGTCQKSGNIYPWQLLKELQEVNFTVYNQTVYFDEKGNPPIGYDIIEWIWVNGA
VSFQTVGSYSISSKGIEIHTSLVSNTVKNHKSVCSENCTKGHQRKTKGYFKCCFECIPCPAGTYVTEQE
PDQCKACLSYQWSPKESSTCLNRTVEYLLWDSGTSIAILSSGSLGIILSIAVACLFVIKLDTPVVKAAGG
KMCFFMLAALTMSFSSVSSFMGRPTHVSCFLRTSLFQIGFIAFTSWLLVRSVQVVCIFKMTGRLPKFM
DYWVKDNGQYLFVFISTASQVIFTFTLLATSIVKPKSDNYEGVTVLNCGDVSVLLPLAQWLQGLLLSI
VCFIFSYMGNDLPKDYNESKCITFSMLVYFACVVCSLTMDFAQGPRDSASRNALAYLVISYGNLGGYF
IPKCYVILLKPEHNTTAYFQNSIQNYTKRQSSIAD
>Latimeria_chalumnae_T1R5a
MALNKITCLLLSILGFPVASCLSQDTEDVPDCIYEFKLAGDYIIGGLFPVHFGIKGLSTQYKPEAMECK
GLPFNIQGYRHLQALRFAVEEINNSSVILPNVTLGYDLFDDCYEAVGIMATFNFLSKPLWSNVKIQKD
YITYQPKVSAVIGPTVSDTAVITSNMLSYFMVPQISYSAASEVLSNKRVFPSFLRTFPSDEKQAEAMVEL
LKEFQWSWIVVIGSDTEYSRRGKQELIKLAPFHELCIAYHRTVSTVISKQREEIAQVISIIVGRNVKVVLV
FGEVVFALNFFTQVTQANVTGRVWIASMVWATDQSVADIPNFREIGTVLGIAPQPGIMTGFDQFVS
KAVYQRSRCLPFLKARWVNDSAKGICNKFCEECQSLTAENILDIPEKRSSFRTYSAVYAVAHALHQLL
GCDSGKCQNKTFFPWQLLEKVKTVNFSLYDKLIYFNEKGEAPTGYDIVMWNWTGESVSFDTIGTYS
VKLGKVDLDARLINWPTEGNTAPVSVCSEDCLPGQKRKQAGPNICCFYCLTCPEGTYLNQSNLYEC
RSCHKDQWAPEGSPMCYERTKEYLSWKNNISLVLVSISTLGLVLTAAASIFFTVNLNTPVVKAAGGKL
CLFMLVCLACCYCSVCLFIGKPTYVTCILRQLVFPISYTACLSCLVLRSFQIMCIFKMAARLPVSFDYWV

KHNGQYIFIYMVTAVQIFLACFWIGISDNEPLFDYHFKDAIVLKCGEYTTRAIITEVAYTGLLSALCFIFS
YTGEELPRNYNEAKCITFTMLIYFISIICYFMFDKAQSERNTTMWLVICILARLYGILGGYFFPKCYIILF
KPKQNTAAFFRACIQNYTN
>Latimeria_chalumnae_T1R5b
MFHHILLIFQLLLANFILHGKENLNDCTGEFKLQGDYVIGGLLPVHFAFNDLYLKDKPDKVDCKGLP
FFIRGYMHLQALRFAVEEINNSSSLLPNVTVGYDLFDDCYEAPAIMATFSFLAQRLESDIKIQKDYISYH
PKVIAVIGPMVSDTAVTTSSVLSYFMVPQITFAATTEVLNNKRIFPSFFRSFPSGKHQAQAMVLLLKKF
QWTWVIAVGSDTEYGRSGKQSIVQLAAENGICVAYHKTILDTGSKVTDDINEVLNVSIKQNVKVILIF
SELPLARKFFQEFIKLEIPGRVWIGSSVWGIDGGTYNMIKSEKVGTVFGVAVQPGRMDGFDQFMTKA
LYDRSKCINFLTPPWTNDSAEGVCNKFCEECNSVTPEEIINIPAKREAFLTYSSIHAVAHSLHQLLDCD
SGSCQKSIAFPWQLLEKLKNINISLQDKLINYDDRGEAPTGYDIVLWNWTTGKPTFDTIGEYSVTLKS
VDIRKELINWHTEGNRIPVSVCSEDCLPGQKRKQAGPYICCFYCLTCPEGTYVNKSNLYECQPCDKD
QWAPEGSPVCYQRTVEYFSWTIDVSIALISISTIGLFLTVGVGIIFIINRDTPVVKAAGGKLCLPMLTSLT
CCFCSIYFFIGKPSLVTCILRQLLFPVSYTACLSCLLLRSFQIMCIFKMAARLPVSFDYWVKHNGQYMFI
CIVTAIQVFLCSFWIGIGNNEPLFVYRFKDAIVLKCGEYTALAIISEVAYTGLLSALCFIFSYTGEELPKN
YNEAKCITFTMLIYFISVICYFMFDKAQSERNSTMWMVICILARLYGVLGGYYFPKCYVILFKPMQNTT
AFFQACLQDYNNRKSSISN
>Latimeria_chalumnae_T1R5c
MFYLILLVFQLLLAICTLRDKENLSDCTDEFKLHGDYIIGGLFPVHYATKGLYLKYKPDAVDCQELPF
YKRGYMHLQALRFAVEEINNSSSLLPSITLGYDLFDDCYEAAGIMATFSFLSANLESNVKIQKDYISYQS
KVIAVIGPMVSDTAVTTSSILSYFMVPQISYTASTEALSNKRIFQSFLRSFPSGKHQAQAMVLLLKKFQ
WNWIIVVGSDTEYGRSGKQSIVQLAAENGICVAYHKSISDTGSKITDDITEVINVSIKQNVKVILIFSEVP
LASGFFKEIIKSNIIGRVWIGSSVWATDRSIYDMTKTGSIGTVFGIAAQPGRMDGFDFFVTKALYDQSK
CINFLTAKWINDSTMGICNKFCEECKSLVPEDFINIPEKRESFRVYSAVHAVAHSLHQLLDCDSGSCQK
SVYFPWQLLEKVKQVNFSLHDKLINFDERGEVPTGYDVVLWNWTTGEPSFDTIGEYSARLKSINIHSG
LIDWHTEGNTIPVSVCSEDCLPGQKRKQAGPYICCFYCLTCPEGTYINKSNLYECQPCDKDQWAPEG
SPLCYQRTVEYLSWTIDVSIALISISTIGLFLTVGVGIIFIINRDTPVVKAAGGKLCLPMLTSLTCCFCSIY
FFIGKPSLVTCILRQLLFPVSYTACLSCLLLRSFQIMCIFKMAARLPVSFDYWVKHNGQYMFICIVTAIQ
VFLCSFWIGIGNNEPLFVYRFKDAIVLKCGEYTALAIISEVAYTGLLSALCFIFSYTGEELPKNYNEAKCI
TFTMLIYFISVICYFMFDKAQSERNSTMWMVICILARLYGVLGGYYFPKCYVIVFKPMQNTTAFFQAC
LQDYNNRKSSISN
>Callorhinchus_milii_T1R6-1
MWLLLAVVLCLVAQAANTSNIAAAFSLPGDYVIGGLFPIHYAQPNGVNQSEPITPDCQRFPFYMNMY
VNSQAMRFAVEEINSDRRLLPGVRLGYNIADTCFQSVDVQTTLHFLSARHLPSPGIPTPATNTDYQP
GVPISSNYTSYQPNVIAIIGPASTTITVTIARFLGYFLVPLISYAASGEVLGNRIRFPSFFRTIPNDIKQAEA
MALLIQEFGWNWIAAVGSDNEYGRQGINKVVELVTGAGTCVAYQAIIPSSRPELVHMLNEISASVNVT

VLFSSRLATERLFRVLTELNVTGKVWIISETVALSEDIARIPGVAAVGTVLGIAIKEGHMEGFREFLAKG
AASEPRGSCDQECRRLLAPAGRSAESPEYHVDTRVSYNVYVAVYAMAHALHQLLQCEQDRCYHPRA
VYPWQLLSSLKKVRFLLSNETFYFDETGDGPSGFDIVTWQSQGHGIHFQTIGSYLPLTHQLDIDRSLI
HWPHNSTYVPVSHCSESCGPGQRRIPRGFHSCCFDCEDCPAGTFRNQSDPYECAACPWWQWSPAQ
SERCLDRAVQYLSWQNPLTVLLLLAANSGLALTVTISVVFAWQLDSPVVKSAGGRMCFAMLASMLC
GFSCVYLFIGKPSTTLCRVQHPIFAISFSLCLSCILVRSFQIVSIFKMASSLPRAYKFWLRYSGPGLFVVLS
TALQLTMCVAWLVAEPPSPRTLLTGSIIALDCSTGNVAGLALSFLYIVLLTGACFLLAYIGRDLPENYN
EAKFIAFSMTLCFVYYILYMASLVTPTQQVYTSSIRTFLTINSAASVALGYFLPKCYIILFQPQLNTAAHF
QSCLQDYTKKQSEGQ

>Scyliorhinus_torazame_T1R6-1

MWLLLSLFVYSIYNIPTSLSFTSPGEYVIGGLFPIHLTPLEDVDRLTPAVPDCEKSKFRKDMWTAFQA
MRFAIDEINNSTKLLPGITLGYDITDSCLEVVDIQASFRFLSGKRRTELEILNNYTAYQPRVIAVIGPPST
DVAITIAKMLGLFLVPQISYLASGEILSDKMRFPSFFRTIPSDKNQANAMSLLIQKFHWNWIAVIGSDN
EYGRKGVNKVVELASAAGTCIAYQGIITTSPEVVIQVINNISSTVNVTVLFSNELTAQVFFSLVVAQNL
TSKVWIVSEAISLSQEIIKIPNIESIGTVMGIAIKEGQMAHFKEFLDKRVNFTNPRIMPDPGLEENTDSR
GSCDQACSQFDWLSAKQLNSILYRLEKRVSYNVYSAVYAIAHALHNLLQCNAAQCDKTHRILPWQLL
SSLKMVKFNLHNHTLYFDEHGDPPTGYDIVLWDWTRIHVHFKIIGSYLPTARQINIDSSLIHWSWGD
NLTPISNCSAMCDPGQRKIQRGIYPCCFDCEDCPSGTFHNQTDPYHCMDCLWQQWSPPKSPLCLER
ELYYLEWNSAFSIFLLILANGGIVLTITIAIIFTLNYNTPVVKSAGGRTCFLMLVSMLCTFSCVYFYIGKP
SKVFCQIRQPFFLVSFTICLSCILVRSFQIVCIFKMAAKLPKAHDYWVKYNGQYLFIFVSTCVQIVICGV
WLIADPPTPKGHIIEKAIILDCNIGNVIAFSLSFLYTVLLTVACFLFAYMGRDLPKNYNEAKFIAFSMMI
CFVYYILYMTSMVTPNQERYTSSIQTFLTVTSAFSIAIGYFLPKCSIILFIPQNNTMTYFQFCIQDYTKK
QNGPN

>Rhincodon_typus_T1R6-1

MCLLLSLFVIAICSFPEHSSAQFTLSGEYVLGGLFPIHLTPLDDVDRLKPVVPKCEKSKFRTKTFTAFQ
AMRFAVEDINNSTDLLPSITLGYDIADSCFEVVDIQAIFRFLSGKQGAELKILSNYTVYQPKVIAVIGPP
STDVAITIARLLGFFLVPQISYQASGEILSDKMRFPSFFRTIPSDKNQAKAMALLIQEFNWNWIAVIRSD
NEYGRKGVNKVVELTSAAGICIAYQAIISTSPELVIQMINNISNTVNVTVLFSNEIMAQVFFSLVVAQNL
TSKVWIINEAISLSQGLTSIPNIESIGMVMGTAIKEGQMAHFEEFLDKPLSLTNPRMLPDSELGESNHC
RGSYEXACSQFNWLNAKYLNSSLSRLEKRLSYNVYLAVYAVAHALHNLLQCNVAQCEKNQSILPWQL
LSSLKEVKFNLHDDIFYFDEYGDPPSGYDIVLWDWMRAQVRFKTIGSYLPNDGQIKIDSSLIHWNWE
NNLTPISNCSVTCDPGQKKILRGIHSCCFICEDCPSGTFQNQSDPYHCTDCRWQQWSPPKSHRCLER
DLHYLEWNSIFSIFLLILANGGIVLTITIAIIFTLNCNTPVVKSAGGRICFLKLMSMLCGFSCVYFYIGKP
NNFCKIRLPFFMVRFTLCLSCILVRSFQIICIFKMATKLPKAHDYWVKYNGQYLFFFLSTFVQIVICSVW
LNTDPPTPKGHIIEKAIIMDCGNGSMTTFLLSLFYTVFLSVACFFFAYMGRDLPKNYNEAKSIAFSMMI
CFVYYILYMMSMVTPNQERYTSSIQTFLIVTSAFSIAMGYFLTKCYIILFMPQKNSTAYFQSCIQDYTKR

LNGAN

>Chiloscyllium_punctatum_T1R6-1_pseudogene

MANISIFTYAELPLLPHNKICLLQSLSVLAICGFPESSSEPFTLSAEHVLGGLFPIHHTPLEDVDRLKPV
VPNYEXXXXXXXXXXXXXXXXXXXXXXXXXXXXXXXXXXXXXXXXXXXXXXXXXXXXXXXXXXXXX
XXXXXXXXXXXXXXXXXXXXXXXXXXXXXXXXXXXXXISYQASGEIVNDKMHFPXFFQTIPSDKNQAKAM
ALVIXEFNWKWIAVIRNDNEYGHKGVNKVVELTSTAGICIAYQGIISTSPELVIQMINNITNTVYIIILFS
NVLTTQVFSSLVVAENLTSKVRIISEAISLSQGITNISNIECIGMPMGIEIKEGQMAHFEEFLDKPLSLTN
LRMMPDSELGKSNNSRGSYDQACSQFNRLTAKYLNSSLSRLEKWLSYNVYSAMYAVAHALHNLLQC
NAAQCEKINSTLPWXLLSRLKTVKFNHLDDILYFXXYRDPPTGPDTVLWDXKRAQVRFKTIGSYLPT
AGQINFDSTLIHWNLXNNLTXISNCSMMCGQGQKKVXRGVHSCCFNCTDCPAGTFQNQSDPYHCT
DYLXQQWSPPKSHHCLERDLHYLEWNIIFSIFLLILANSEIVLTITIAIIFTLNCNTTYVHSTGGRMCIFV
LMSMYCGFSCVYLYIGKPNNLFCKIRQLFFMVSFTVCLSCILVKSFLFICIFKMATKLPKAHHYWVKYN
GQYLFIFLCTFIXIVIYSVWLITGPPIPKGHITGKAIIMDCDNDNITASSFSLFYTAFHSVACFFLFAYME
RDLPKNYNEAKSIAFSMMICFVYYTLYMMSMVTPNQERXTSFIQTFLTVTSAFSIAMGYFLPKCYIILI
MPHKNSMAYFQSCIQDYTKRQNGAN

>Callorhinchus_milii_T1R6-2

MQCVVLMALSFAAQGWAVGEGWSSTLGGEGRLPADYVIGGLFQIHMEAQSDWSKPEIPKCESFSAD
GYQHFQAMRFAMAEINNSTSLLPNVSLGYEVFDDCQINIDTLASFAFTRAPRCSHHPAVAAVIGPWQ
SQSAIITATILGLDLIPQISYGATVELLSDKRSFPSFFRTIPSEETQIDAILLLIRRFQWNWISVIGSSNQYG
VNGKLKVIAGALANGICVAFQGTIPEHGLDSEREIAEIVGNIMHSRANAVVVFSEIVSAELFFRVAVGR
NVTGKVWILSEDVSLQRLLSDIPNIWKIGTFLGIGVKRGQTDAGYMQFVQEAVYENLRYSELQNVSR
ASGDLTCGPINHRNLSIPSESLRLVLGYLDWRVAFNVYTAVYAVAHALHQLLQCNTGECNRSTEVYP
WQLLEALHAVRFSLHNTSVYFDRNGNPPMGYDIIHWAWNNQSVSFRVIGTYTSSPRELHIDETAILW
HSEGNTIPTSNCSNECEPGQFKEIVGFKACCFTCKDCPAGTFQNQSVCTACEPSQWSTTRSVVCHNR
TLLYLSWGSNISIALGALSGAGILLIAGIAALFVANLDTPVVRAAGGRLCLLVLASLACVYGSACLSIGK
PNAAVCRVRQLVFGVSFTLCLSCLLVRSFQLVCIFKLATRLPRAYDYWVKYGGQYVFIAASTAVQFVF
CLIWLLCDPGKMQPNYSFSETKMLLECANSTLLYVLLSNVGLVLLSLLCFSLAYWSKDLPKNYNEAKA
ISLSMALCLLAWTLWLLVLFSTSASFAVVGEASAILLCSYAITGGYFLPKCYVILFKPQHNTTAFFQTCI
QAYTRSRSALDP

>Rhincodon_typus_T1R6-2

MMFYLVLLVRCFTAQVFANRYDAECCRLQLQLPGDYIIGGLFQIHVESYRSQSKPEVPTCKMFSDNG
YILYETMRFAIEEINNSSSLLPNVTLGYEIFDDCAITIDIQGSFAFVSKPSEHAVKTVQFDHRYQPLASVL
IGPLKSDAAVITSNILSLLDVPQISYGASSEQLSDKTEYPNFMRTIPNDENQIEAIILLIQKFRWNWITVI
GSSSDYGEIGKRKMISGATAVGICVASYGTIPQHELESKQEIINIIHTITANKVNVIVVFADVHHAEHFF
QEVVAMNITGKVWILSEAMSVKRTLSQIPNIKRIGTILGIAIMQGHMPGYDKFVLRDIIALNSGVSQSIK
YSEKQRERRDCPDNNFLATCNQCIPCTTDGVKSLLQYSEWRIEFNVYSAVYAAAHSLHQLLQCDSGN

CKKESAYPWQLLKTLKTVNFSLNGVPISFDENGNPPTGYDIIYWDWKHEVVSFNVIGSYKPKPGRLQI
DSSLIKWNSPEGKIPTSNCSTECEPGQIKVTVGFQPCCFICKDCVEGTFQNESECIPCNPNQWSPSKST
ICHNRYMVYLRWNENISIVIALATGIGLVLKVLIGAIFVIYFDTPIVKAAGGKLCFVLLLALAFSCSSVYS
FIGEPSPGICITRKLLFTVSYTTCLACLLVRSFQLVLIFKMASKLPKAYSYWVKYNGQYIFVFIMTVIEIT
SFFIWIIRKIPVFERINDISQTESILTCGPTNFLFSLLSFCGPIFLCLLCFMVSYLSKDLPKNYNEAKYITFS
MAVVLISWIFLFLVIFSSPNSFIPCVEAAVVLLNTYSITVGYFLPKCYIILLKPERNTTAFFQTCIQQYTR
NRVTPQP
>Scyliorhinus_torazame_T1R6-2
MMFHLVLLAMCFSAQGFANGRDAECCRMQLNLPGDYIIGGLFQIHIESYWNHSKPEVPLCKRFSDY
GYILFQTMRFAIEEINNSSSLLPNVTLGYEIFDDCEISIDVQGAFAFVSRPPEHAVEITQFEYRYQPLVTA
VIGPLTSDAAIITANILSLFDIPQISFGASSEELSDKKLYPNFLRTIPNDESQIEAIILLIQEFRWNWISVIGS
STDYGENGKRKVISRAAAVGICVASHGTIPQKASESKQEVINIIDNITNNRANVVVVFTNIQHAEYLFQI
VVDMNITGKVWILSEAMSVQRILSEIPNIKRIGTVLGVAVMQGKMPGYKEFVLRDITAWNPGINQRV
KYSDPQEERQECPADSFMPTFNQCVPCTSDGVRSLLRHSDWRVEFNVYSAVYAGARGLHQLLQCDS
GECKKEGAHPWQLLETLKRVNFSLNGMPIYFDENGNPPTGYDIIYWDWKNEIVSFNVIGSYTSNPGQ
LQIDGSLIKWNSQEGEVPTSNCSSECQPGQIKIAVGFQPCCFICKDCVEGTFQNESECVPCSLNQWSP
SKSTICYNRHLVYLRWNESISVAIALGTGIGLILIVLIGAIFAIHLNTPVVKAAGGKLCFVLLSALTVSCTS
VYSFIGEPNVELCFIRKLVFTVSYTTCLACLLVRSFQLVLIFKMASKLPKAYRYWVEYNGQYIFVFIATV
TEIGVSFIWIFSKFPVFERNYDISKTEVILMCGPTNHLLSLLSISGPISLSLLCFVFAYLGKNLPKNYNEAK
YIAFSMALVLFSWIFLFLVVVSSPESHIPGVEAAVVLLNSYSITVGYFFPKCFIILLKPERNTTAFFQTCIQ
EYTRNRETQQP
>Chiloscyllium_punctatum_T1R6-2
MMFYLVLLLRCFSAQVFAIGHDAECCQMQFQLPGDYVIGGLFPIHVESYRNYSKPEVPMCKMFSDN
GYILFETMRFAVEEINNSSSLLPNVSLGYEIFDDCAITIDIQGSFAFVSRPPEHVVETDQYDRRYQSLAS
VLIGPLKSDAAIITANIVRLFDVPQISYGASSEELSDKTEYPNFMRTIPNDENQIEAIVLLIQKFRWNWIT
VIGSSSDYGETGKRKVILSATAVGICVASYGTIPQHESESKQEIINIINTITANNVNVVVMFADVQHAEH
FFQVVVAMNITGKVWILSEAISIKRRLSEIPNIKRIGTILGIAVMQGHMPGYNEFVLRDIITLNSSINQSI
NYTDPQEERRDCTDNIFMATYNQCVPCKTDGVKSLLEYSEWRIEFNVYSAVYAAAHGLHQLLQCDS
GNCKKEGALPWQLLETLKTINFSLNGVPISFDENGNPPTGYDIIYWDWKNEVVSFTVIGSYKSKPGRL
QIDGSLIKWNSEEGKIPTSNCSVECEPGQIKVTVGFQTCCFICKDCVEGTFQNESECIPCDLNQWSPP
KSMVCHNRYTVYLRWNENISIVIALVTGFGLILKVLIGATFVIYFDTPIVKAAGGKLCFVLLLALFVSCS
SVYSFIGEPGPGICITRKLLFTVGYTTCLACLLVRSFQLVLIFKLASKLPKAYNYWVKYNGQYIFIFTMT
VIEIISSFSWIFSKIPIFERINDISKTGSILMCGPTNLLYSLLSVCGPVSLCLLCFMFSYLSKDLPKNYNEAK
YITFSMAVVLVSWIFLFLVIFSSPNNFISRIEAAVVLLNTYSITVGYFLPKCYIILLKPERNTTAFFQTCIQ
QYTRNRETPQP
>Rhincodon_typus_T1R6-3

MDTFWPNFELPGDYIIGALFQVHSDARYNSDRPETPRCINISVNGYRHLQAMRFAVEEINNSTELLPN
VTLGYQIFDGCATSINIKAALEFILGKSTQETELCHDFKGCWPEFLAVLGPWSSDDAIAIASILQLFHIP
QISYGASSDELSDKMHYPTFFWTIPSDMKQAEAMVLLIQKFGWNWIAVIASDNQYGKNGKPRVVELA
SERQICVAFQGLIHAPRLNQAIINMVNHMSLSKANVIILFAGQFYARAFFQAAVEVNVTGKVWILSESS
FGNITASDILNAKTTGTFLSIGVKQGQMPGFGEFLMQSMATQSLAVDQHHLISVKETESSKVSREEN
CAQVCTECQYFTSASVQXVLGSLVWSISFNIYAAVYAVAHALHQLLSCHSGSCDKSRAFLQSQLLEGL
KRVNFTLHNNLIYFDENGNPPMGYEIILWDTQNESSPMKVIGSYKPNPGQLIINESLIKWNSPTDSLP
MSNCSRECDPGQWKKKDTFHSCCFVCADCPAGTFEMNDECVNCSLQQWSPQKSFTCYDRAVIYLR
WGSNISIALSVMASAGLLTMAGITLVFVVYLNSPVVKAAGGRQCFLMLFTLASGCCSVFLFIGKPNEV
TCRVRWPLFTVSFTVCLACIFVRSFQIVCIFKMATKLPKACDYWVKYSGQYVFVFASTSISAIDCFVWM
VQAPVVTVSNYNLSQKEIFPLCTTDNNISIFLSGFLYNGLLGCLCFVFAFLGKDLPKNYNEAKCISFSM
VLYIASWSCCILVIVSGFQQYLPLAQALAALLSLFGILVAYFLPKCYIILFKPQCNTAVFFQSCIQDYTKN
REV
>Chiloscyllium_punctatum_T1R6-3
MLIFVLIVVPTYTLEHSETKEGDTDTFWPEFELPGDYIIGGLLQIHSDAHYHSDLPETPRCMNFSVSG
YRHLQAMKFAVEEINNSTELLPSVTLGYQIFDSCATSINVKAALEFILGNSTQGAESCHDFKGCRPELL
AVLGPWSSDDAIVVASILQLFHIPQVSFGASSDELSDKVQYPSFFRTIPSDLKQAEAMVLLIQRFGWN
WISVIASDNQYGQNGKRRVVELASERQICVAFQGLIHDPGLKQEIINMITYMSLSEANVIILFASQLYAT
AFFQAAVEVNVTGKIWILSESSFGNITASEILKAKTTGTFLSIGVKQGQMPGFGEFLIKSMATQSPVIN
QKHLISVKETENSKVNRQENCAQLCSECQYFTSATVQALLGSSVWSISFNVYAAMYAVAHALHQVLN
CHSGSCDKPRAFLQSQLLEGLKKVNFTLHNNLIYFDENGNPPMGYEIIAWDTQNESSPMKVIGSYRP
NPGRLIINESLIKWNSPTGLVPMSNCSSECDPGQWKKRDTFHRCCFVCTDCPAGTFEINDECVNCSL
QQWSPEKSLTCYNRAVVYLQWGSNISIALSVMASAGFLTMVGITLVFVIHLNTPVVKAAGGRLCFLM
LFTLASGCCSVFLFFGKPNNFICRVRWPLFTVSFTVCLACIFVRSFQIVCIFKMATKLPKAYDYWVKYN
GLYVFVFASTSISAINCSVWMAQEPVVTVSNYNLSQKEIFLLCTTDNDLSIFLSGFLYNGLLGCLCFVF
AFLGKDLPKNYNEAKCISFSMVLYIASWSCCILVIASGFRQYLPLAQALAALLSLFGILVAYFLPKCYIILF
KSQCNTTVFFQSCIQDYTKNRAA
>Callorhinchus_milii_T1R6-3
MWCFVLLTVSLFIGPIETAGEWDSSSTDFELPGDYILGGLFPIHTEAFSTLSRPIVPTCKNFLRASYCIF
QAMRFAVEEINNATHLLPNITLGYQIFDDCTSSIYIKAMFAFVLRRAQTSSGATHNLCGYQPRVTAIIG
PWSSDGATIVANILHPFLLPQISYGASSEKLSDKVYFPSFLRTIPSDKNQAEAIILLIQRFKYDWIAVVGS
DDQYGQYGKHEVTTLASERNICMAYQGIIKAKEGTLKEAIIQVIKHIDSSRVNITVLFATLQLTVMYF
QTAIELNIRDKVWIISEAISANNILSEIPNISKVGLILGIAVREGQMPGFQDFLSNALAAQSLSPSNLSQA
SAESCAQTCIECQSFTNAMVQSILGSNKWGISFNVYSAVYSVAHSLHQLLNCDSGTCNKSRTFLPSEL
LESLKRVNFTLHNRTINFDANGNLRTGYNIIIWENKSSIDPFRVIGSYTPNPGRVVINQPFNTWAPGLK
KMPTSKCSSECEPGQRKNLDGFHHCCFKCTDCPKNTFESNHDCVECFKWQWAPSKSSVCYNKTIVY

LRWEDGISIALCAMAGAGILAKATITVIFIVNLKTPIVKAAGGKLCFVMLLALVSGSSCVFAFIGLPSEL
VCKVRQPVFAVSFTVFLSCILVRSFQLACIFKMASRLPKAYAYWIKYNGQYVFVLLSTMVSVFHSLMLL
INHPSSFMRRDSLSDLELILHCNSDLFIVFLKSYIYNILLSLLCFLFAFLGKDLPKNYNEGKWISQSMVVF
IFSWSLFALISFSASQRYIPIIQAVVILSSSYGIVGMYFIPKCYIILFKPQQNTNAYFQMCIQEYTKRRST
>Gekko_japonicus_T1R7
MPVWLLVAVLLVDHGGAYDPCESLFSLAGDFIIGGLFPVHYDSTRRSGARGPELPSCAGLNSWGYSQ
FQAMRLAVEEINNSSHMLPNVTLGYHIWDTCSESIYLQAALQLAPDRLHSFQKGSSNKMVAVVGPDE
ADMTLLTSRVLTFYRLPQISYSAKDQRFTDTNLFPLLFRMVPNENHQIYGILSLIEAFDWKWVSAVGS
GTKSSQKAIQTLVAEASARNICITYQGVMTWDNEVTKIQLQRIVSNTVKAKTNITIVFAEEDITYKFFK
TVVELKVTGKVWIALESWVLSDMVASISGIQETGTVIGLTIKPIKLPQFTRFVERTMRCNTLSNRTSLV
LSASEKLFETESCGQSCEECHLLSQESLAYILQSSIWHWSFYTYAAIYAVAEALHLHLGCETGSCKRDE
YFEPWQLYELLYHVNFPLQNNTIRFSSQGDLFLGYDVLTWIWTNGTVIAKTIGSFSSETLDIASGHIK
WSTPDGQVPQSICITECDEGQIRSQQTLDECSCRCEDCLEGTYPNQTYLDACIPCPSGTWSPKKSQE
CFPPTLTFLDVKDTIISALLILTIVDFFLLCSCGLVFATHWQTPVVKAAGGKLAFVMLGSLMACCATT
SLFVGRPTSLSCLIRQPIFAISFTLCVSCLLARSFQIIFIFKMAHKLPMIHKYWIKYRGTYFFVAISCGVQG
LVCFLWLFFSPPSLQGDTVSKKEIFLRCSEGHYLGLGSVLGYITLLCATCFVFAFWGRNLPKNYSEARL
QTVSMLVFLMAWGCFMLIYTTTEGKGKQIAGLQMFTVQTSVYAILCTFFLPKCYIILFKPQANTVAH
FQTCIQAYTTTTRNSAK
>Pogona_vitticeps_T1R7
MSAAWVVAAALLLVSPGRGAHTSCPSLFHLPGDLMIEGLFPVHSNSTGRNRSMGQLPVCRGLNSWG
YSQFQAMRLAVEEINNSSHLLPNVTLGYHIWDTCEESLYLQAVLQLDPDGEHNSRAGYSDRVVAVV
GPDTTDMTHLLSRILTFYQFLQISYNAKDQIFTDKRQFPLLFRMVPNENHQTRGLLSLVQQLGWKW
VSAVGHGTQASQTSLQMLISEARAQGICVSYQGLLANRDWTLVSREQLKKVIENIERTKTNVTLLLV
DNSVAQSFFRVVVELKVTRKIWIAPETWVLSEEVSNLPGIETVGTILGLTIKPVILPEFLPFVKKTLRCN
PWNGLSPSEQQALKNVGGCYQFCSDCHSLSLEILEDVLNSRIWHWSFYSYAAIYALAQALHQLLGCD
HESCPTKEVLTPWQLYEILAQVDFSLQNNTIKFSNQTDLFLGYNVITWNWVNGSLDHKTIGNYSAQS
LIIDQSKIKWPTPDGLVPTSTCVTECKAGQIRSKHTLDECTCRCDSCPEGTYQNQTNSDFCLPCPPQ
MWSPKRSSTCFYPVITFLSIRDTTVAALIIVSLVDFSLLCGCLLVFALHRQTPVVRAAGGKLAFVMLVS
LLASCTTTLLFVVEPTPLGCLVRQPIFALSFTLCISCLLVRSCQIVFIFKLARRLPWAHKLWLKYQGAYA
SLGLSVLLQGGLCALWLSLSPPSLQADVVSPREIFLRCSEGHVLGLGSVLGFLGLLGAACFALAFWGR
NLPKNYSEARLLALSMLVFLMGWGSFMLIYATTEGKGRQIATLQMFTVQTSVYAILCTFFLPKCYIILF
KPQHNTVAHFQTCIQTYTATPQTMTP
>Anolis_carolinensis_T1R7
MRPRWLLLAGLFLAGNGAAHPGSCKLPFHLPGDFLLGGFFPIHNNGTCSSLDPWGFTQSQAMSLAV
EEINLSSHLLPNVSLGYHIWDSPTDTLFLRGLFRLAPGQRESPHGAADWARKVFGVVGPYESVLAPLA
SHILSLYGLPQISYSIKDGNFDNSSQQSLLFRTVPSTAHQLDGIVALVRAFRWEWVSAVGSGTKATQK

SVRILIDRAAAQGVCISYQGLMPGGPVSTLSQLRKVVENIKRAGTNVTIVLGNDATVQQFFQVVVAL
GITGKVWVATESWVLTDAVATLPGMERVGTVLGLTIRPIELPHVRRFVEEALSSGPPHGSSCPQSCFK
CPPPSPESLTGLLDSTLWNWSFYSYAAVYAFAHALHRSLACSQEACPSSQEPQPWQLLEALHEVDFPL
LNNTIKFSGQQDLFLGYDVMTWAWKDGGGGVDYVKIGSYMAQSLDINWTQIRWPGPEGQVPSSSC
AKECAAGQSRSKQLLDECLCRCDDCVEGTYQNQTNSDDCLPCPNGTWSPPRSSRCFPPSVTFLRLS
DSNVVALLAVASVGFLLLLGCLLVFAAHRHTPVVRAAGGGLAFIMLVALLASCAAAVLFVGQPSSRTC
LIRQPIFALCFTACVSCLLVRSLQVVVIFKAAGRLHRGRQCWRRFQGPYLAVGLSCGVQAALCAAWLS
LSPPALMQQASGPREVSLRCWEGPFLGLGAVLGYITLLAGACFLLAFWGRHLPENYSEARLLTASML
VFLMGWGAFLFMYATNGSQQTAALQMFTVQTSVYAVLCTFFLPRCYVILFRPERNTVAHFRTCIQA
YTTTAADPETPRPGTQGV
>Ambystoma_mexicanum_T1R7
MWLWMVPGLLLLFMGLVQPVQCAINRLASQFSSPGDYVIGGLFAVHSSVQKLQDGTHIVPICTSFNI
PGFRRFQAMRFAVDEINNSSDLLPGIRLGYRIFDVCSDSAAVSAILEASCGGLACSVPVLGSYSTHSPQ
FLGLVGPQSSDAAMTMARALAFYSIPMVSYAASSETLAQKNRYPTFFRTIPNDQNQAQAQVLLMKYF
KWHWIAVLGSDSQYGRQGLERFITLAPKSGICVAYKAHIPKDTIGNLAMAKVKEIVSQLRRSAVNVTL
LFSEPEYLESFFSIVVEQNLTGKVWVASESWVQSTRISFMNNIGNVGTVLGVAVKQVLMPGFKDFIAK
ALGAADQEKADVLPEGGLNLGEGNPISALQVSGGSFRTGCYQTCSECNRLTVEDLPRIWSPPERRKS
FNSYAATYALAHGLHNLLDCSLGKCNTSSSIFPWQVLQELRKVNFTLQGQNIYFDEFGDPPTGYEILA
WNWTIPNVSFVTIGHYEPLSGELHINESLIKWAKNEIPLSECSPQCQPGQIKRLKGYHSCCFDCEDCP
EGTYQNPDQDKCLECHLDQWSMPRSAACSSRTVVYLAWPDPVSCTLSMLGLLGLLIIAAIAVIFKIHL
DTPVVKAAGGKLCYLMLSSLTVTCLSMGLFIGEPRTMLCNIRQPLFGVSFTVCVSCILVRSFQIVCIFK
MAARLPKAYDYWVKYSGPYLFIATATFLQIIICVLWMLFDRPMLQSDYEFSKTEIFLGCQEGHFIGFG
VMLSYNSLLCVICFLFAFMGKNLPKNYNEAKCITISMLAYFISWIFFILTYSTAHGKYIPAFQVFAVLSST
YGILGAYFFPKCYIIIFQPSNNTTAYFQNCIQMYTTKRREEKD
>Polypterus_senegalus_T1R8
MASLLPLSLLISACPPLLWGQLTSKDFYAGQLRAPGDYILGGLFPLFRASARVNQSEQMVTCDRFNPL
GFNWLQALRFAIEEINNSSDLLPDIQLGFEVWDTCQQFNRVITPTLHFLTVPLDDELQPKCNYTSLV
PRTLAVIGPSTSEEALTSATMLSYFMVPQVSYSASSSSLEDRTHFPSFFRTIPSDSQQTLALVELVERFR
WDCISILAADSDYGKGSLATLQELFRYRNVCISYSDVIPGVSQDRESLLQQMVIKLDMFLVNVTIIFAD
ISSAMALLSSVITWQTAGRNRVWIASEAWSTSNEIASLSNIHSIGTIVGLAIQTHIFPGFQEYLQSSKVIH
TPAPTQLTNLSTASLESCQVSMADDTQIELLKMAIVEPEERISLNVYTAVYSVAHAIHSARQSIGADAT
LHPWQILEALKSVSFEVHNISVSFNSKGNLHAGYDLLVWKAIDGTIQFVPVGQYTAIGSLRVDTEEIW
AAQGVQIPESKCYEDCLPGQIYRKRFSAWSSCMFICENCPEGTYQADLVTCLPCTLEEWSPARSTACL
ERDAQYLSWGDPYVMVLVVIACFGLALIIAVFVLFAIRLDTPVVKAAGGRMSLLMLFSLACSSLSLGSF
VGRPNDFHCKMRQPLFAISFTVCISSILVKSFQIICIFKMAAKLPMAYKYWMRYNGPYMCILGSTLMQ
ATICLIWYSIKAPYLVNNYKISSNQVYLQCYEGSFVGFTLMLSYIGLLGVLCFIFAFMGRKLPKNYNEAR

FIVAGMLIYFISWFSFFLTYATSEGKYVAAVQIFAVLNSTCGILFTYFMPKCYIILLKPESNTTAYFQNCL
RSHGVARHSSHPSSTEATEPKDTGDCT
>Protopterus_annectens_T1R8
MSLLIHINLLFALSIFSCDSTVGFILGQSKKSGTYIIGGLFSVSQHGNGTNTCQDSKARTLPEIPVCNMY
QPDIYGCMQAMKFAIEEINNSTLLLQNVTLGYEVYDSCADPINTIQPTLSFLSHGLSACLHMQCNYT
DYNTHAIAVIGPATSGEAEISGRIFSSFMVPLISYSATSSTLDDRQKFPTFFRTVPCDEFQVDAILQLVK
KFSWDCIAAIGSGSAYSTQLISDLENQAGDRNVCLAYKKMVTFPSVNFTGVISKVLQEMENFKIKVIVV
VAEGGWAIEFFKTVILCNFTNKVWLASEAWATNMDVHAIFPLKTDSVLGLSLKSEKMPKFKEFLLRSL
QQDMNQEAGVLPWCGKCSITKSMNKSECQKKLLNDSQKLEIVSDYPQWRLSYNIYAAVYAVANALD
NLLRAIPGQKNIYAWQVSHELTKINITLPNNNILYFDKYGNAPSGYDVVFWDSTNSSITTIGQYSARN
KTLDINEKMIRSGGNNTVPIASCLRSCPPGQVKRSKGLNSCLFDCENCSKNTYQKDDSECANCGEDF
WSDPGSPTCTEKNELYLDESDPFVIALFVTSSTGLALTVAIFIIFVIYLQTPVVKAAGGKMCLLMLFSLA
CSCATMFLFIGKPNDIICKLQQPIFAVSFTICISIIVVKSFQIVCIFKMAAKLPKAYTYWLKYNGPYMFIFI
SASLQLLICIVWLTIKAPALKKHASQTEKEVEIHCDNGAFAGFVMMLVYIGVLAGACFIFAFMGRKLP
KNYNEAKYIVSGMLIYFMAWLSFIMAYYISTTRYVSAIQAFAALTSVYGIICTYFIPKCYIILLKPENNTTA
YFQDCIRNHGNKKKETSQKN
>Homo_sapiens_T1R3A
MLGPAVLGLSLWALLHPGTGAPLCLSQQLRMKGDYVLGGLFPLGEAEEAGLRSRTRPSSPVCTRFSS
NGLLWALAMKMAVEEINNKSDLLPGLRLGYDLFDTCSEPVVAMKPSLMFLAKAGSRDIAAYCNYTQ
YQPRVLAVIGPHSSELAMVTGKFFSFFLMPQVSYGASMELLSARETFPSFFRTVPSDRVQLTAAAELL
QEFGWNWVAALGSDDEYGRQGLSIFSALAAARGICIAHEGLVPLPRADDSRLGKVQDVLHQVNQSS
VQVVLLFASVHAAHALFNYSISSRLSPKVWVASEAWLTSDLVMGLPGMAQMGTVLGFLQRGAQLHE
FPQYVKTHLALATDPAFCSALGEREQGLEEDVVGQRCPQCDCITLQNVSAGLNHHQTFSVYAAVYS
VAQALHNTLQCNASGCPAQDPVKPWQLLENMYNLTFHVGGLPLRFDSSGNVDMEYDLKLWVWQ
GSVPRLHDVGRFNGSLRTERLKIRWHTSDNQKPVSRCSRQCQEGQVRRVKGFHSCCYDCVDCEAGS
YRQNPDDIACTFCGQDEWSPERSTRCFRRRSRFLAWGEPAVLLLLLLLSLALGLVLAALGLFVHHRD
SPLVQASGGPLACFGLVCLGLVCLSVLLFPGQPSPARCLAQQPLSHLPLTGCLSTLFLQAAEIFVESEL
PLSWADRLSGCLRGPWAWLVVLLAMLVEVALCTWYLVAFPPEVVTDWHMLPTEALVHCRTRSWVS
FGLAHATNATLAFLCFLGTFLVRSQPGRYNRARGLTFAMLAYFITWVSFVPLLANVQVVLRPAVQM
GALLLCVLGILAAFHLPRCYLLMRQPGLNTPEFFLGGGPGDAQGQNDGNTGNQGKHE
>Mus_musculus_T1R3A
MPALAIMGLSLAAFLELGMGASLCLSQQFKAQGDYILGGLFPLGSTEEATLNQRTQPNSIPCNRFSPL
GLFLAMAMKMAVEEINNGSALLPGLRLGYDLFDTCSEPVVTMKSSLMFLAKVGSQSIAAYCNYTQYQ
PRVLAVIGPHSSELALITGKFFSFFLMPQVSYSASMDRLSDRETFPSFFRTVPSDRVQLQAVVTLLQNF
SWNWVAALGSDDDYGREGLSIFSSLANARGICIAHEGLVPQHDTSGQQLGKVLDVLRQVNQSKVQV
VVLFASARAVYSLFSYSIHHGLSPKVWVASESWLTSDLVMTLPNIARVGTVLGFLQRGALLPEFSHYVE

THLALAADPAFCASLNAELDLEEHVMGQRCPRCDDIMLQNLSSGLLQNLSAGQLHHQIFATYAAVY
SVAQALHNTLQCNVSHCHVSEHVLPWQLLENMYNMSFHARDLTLQFDAEGNVDMEYDLKMWVW
QSPTPVLHTVGTFNGTLQLQQSKMYWPGNQVPVSQCSRQCKDGQVRRVKGFHSCCYDCVDCKAG
SYRKHPDDFTCTPCNQDQWSPEKSTACLPRRPKFLAWGEPVVLSLLLLLCLVLGLALAALGLSVHH
WDSPLVQASGGSQFCFGLICLGLFCLSVLLFPGRPSSASCLAQQPMAHLPLTGCLSTLFLQAAETFVE
SELPLSWANWLCSYLRGLWAWLVVLLATFVEAALCAWYLIAFPPEVVTDWSVLPTEVLEHCHVRSW
VSLGLVHITNAMLAFLCFLGTFLVQSQPGRYNRARGLTFAMLAYFITWVSFVPLLANVQVAYQPAVQ
MGAILVCALGILVTFHLPKCYVLLWLPKLNTQEFFLGRNAKKAADENSGGGEAAQGHNE
>Gallus_gallus_T1R3A
MIPWVLLCMSFGCAAALKPSCLSAQFRRPGDYIIGGLFPFGMDTINLTARSEPTLIVCERLFVDGLIWA
LGMKFAIDEINNSTSLLPGVELGYDIYDTCFEPLAALQPSLLFVTQNGTTGIGIACNYTDYQPRVTAVI
GPHKSDLCLLTAKLFSFFLIPQVSYGASSEKLSNKELYPSFYRTVPSDKNLVEAVVLLLDEFGWNWIAT
IGSDDEYGRGAQELFLSTIGNSSICIAYEGLIPSDLTDPRAEKQLEETIQYINKTNVNIIVLFAFRQPAQA
LLEQSIKMRLSKKVWIGTEAWLLSDIAASIPNIQNIGTVLGFIMKASTVPGFQKYVANLLSSVQQDEFC
QKSREFYRHVSSDTLGTQCQQCDHISLNDISSTLSHSQIQPVYIAVYSVAYALHRALGCTHQGCPRASI
RSWQLLHFMNTVPFTVNGQSFRFDESHGTNSGYNLIFWHWENGSLTHLPVGDYQESLYLNKSLIQF
HTTDQKEPTSECFRECEPGQIRQIKGFHLCCYDCTDCPENTFCSSKDSSTCTPCLEHQWSPARSTQC
YDRSERYLRWNEPLTAGLLISMSIIISLICLTAVLFVKNLNTPLVQAAGGNLNLFALFALTLMCLSSCLF
IGKPTNNLCMMQQIVCALCLNACFSTFFIKSLEIVLLTEFPRCARTALRWVTPSRSWLLVALCLLTECL
FCFCYLHLGPDYVLPDYSSLPTEVLLMCSTASWPAFALMHGYNGCLAFVCFLCTFMVQSSGKKYNM
ARGITFTILIYFIIWIFFITVFATLRTVLMSVIQISTILMVSLGIVGTYYIPKCYILLLKPDLNREDYFQYST
KEEPEGD
>Gekko_japonicus_T1R3A
MAAFLVLGIGLGWTLAREERCISSQFRKPGDYLLGGLFPLRVLTTSAMDRTLPDVYMCERLYATGLV
WALGMKFAVEEINNSTTLLPGIKLGYNIYDDCSEPIVALQPSLLFLTRTGSNRIGVLCNYTDYQPRVL
AVIGPHSSQLCTVTAKLFSFFLIPQISYGATSQKLNSEEMYPSFFRTVPSDKIQLDAMVELLTSFKWNW
IAVIGSDDEYGREGLSLLSSMAVSKSICVAYEGLIPADVLSPDFDKTLVSTIESVNKTNVNVIVLFSNDR
TVRALFKECLRLGLGKKVWLATEAWVMSDVVTSLGRVDSIGTVIGFIIKAGNVSSFHEYAIRLFELSQQ
KSFCEASQKEANEVGSDVLGSQCPQCDQASLETIKGVLEHRQTFAVYTAVYSVAHALHNALGCESGR
CNKSSVKPWQLLEEMKTLNFSTRNQSFQFDRYRSINKGYEVIGWSWKDGQIKYTTLGEFNGKLNINK
SLLLFHTENQQKPTSQCLTTCQPGQIRRMKGFHLCCYDCIDCEKGTFCSSREDSACTPCPEHQWSP
KRSTRCYDRSEKYLFWLEPLSLILLGLLFLALALTCLTGALFLKNLHTPVVQAAGGAMSLLALSSLAM
MCVSTSLHIGKPSPTICKLSQPFFALCLNVCFSTILVKAFQIVLVHDFANSRRTFLHTLIQKQPWSIVAS
CLLAESLFCYWFVYDVPPVVVRNYALLPTQVLIQCKIESWPAFALIHGYNGILAFTSFLCTFMVQMPA
KKYNVARGITFTMIAYFIALVFFIPTYTSVKQEYQPAVQMAAILLCTLGLLACLYLPKCYIIWFKPDRNT
TEYFQDYTQERLEEKDCQD

>Pogona_vitticeps_T1R3A

MAMLFLLTVPIDWAFSQEKYCMSSQFRRPGDYMLGGLFPFTVYTGNNTDRILPDTYTGNSLRRMYY
AGLIWALGMKLAVEEINNSTALLPGITLGYDLYDTCREPMVALQPSMLFLSRTGTHSIGVLCNYTDY
ETRVMAVIGPDNSELATVTAKLFSFFLIPQISYGATAEKMNNKELYPSFFRTVPSDKRQLEAMVQLLR
VFQWNWIAIIGSDEEYGREGISLLSSMAASYKICIAYEGIIPAEVTDPDLQSKLKKIIRSINDTNVNVIVVF
AMDRSVRELFKAALALGLKKKVWLATEAWVMSDVVTTVKNISAIGTVMGFVIKAGEIPCFKDYVYRL
LEETQRDGFCQASQEEANKVGPDVLGPQCGVCDNISRENVEVVLHHQQTFAVYAAVYSVAHALHA
ALRCSTGQCLRKSIKSWQVLEELRTINFTVNNQSLHFAEDQSINLGFEVIGWKWKNNRIEHISLGEFN
GNLTINMSNIQFHTEDRKAPRSECLTTCLAGQIRRMKGFHFCCYDCIDCESGTFRSSSDDSTCTPCPE
HQWSPARSMQCHNRGEKYLFWSEPMTVALVLVLCFIVALICLLGTLFLKHLQTPAVQATGGGLCLV
ALLGLALLCTSTVLYIGKPTATVCRVQQPLLALGLNLCFSTIAAKALQIMLAHDFADSRPNVLHRLIR
THPWALVAWSFLVEASFGSIYVYHTPASLVKNYKLLPTQVLFQCLTTSWPILFLLHGHNACLAFTSFL
CTFMVQQPPKKYNAARGITFAMITYFITLVVAVPSYATVKPINQPAIVICSVLGGTLGLLVTYYLPKGY
ILWFKPEWNTSDYFQDYTKEKLQEKASQD

>Anolis_carolinensis_T1R3A

MFLLFLLGIGWDGASAQDFRCMSSQFRKPGDYILGGLFPFTVLTTNMSDRTLPDIYSCDRLYAAGLI
WALGMKFAVEEINNSTTLLPGITLGYDFYDSCMEPVVVLQPSLLFLSEMNTSSISVHCDYTDYWTRV
LAVIGPHSSELSMVTAKLFSFFLIPQVSYGATSEKLNNEQYPSFFRTVPSDKIQIDALSQLLIAFKWNWI
AVVASDDDYGREGLSLLSSTMNGKSICIAYEGLIPTDLSNANVQEKMSQVIHSINETKVNVIVLFSKDR
SVREFFKMWFKLGLGQKVWLATEAWVMSDVVLTLKGVQNIGTVIGFVIKARNVYAFEEYTSDLLELT
QQESFCRESREQAGQLGSVLGPQCPQCNNISHHQVTAVLGHRQTFAVYTAVYGVAQALHEALQCQ
NGQCQKHRVKPWQLLEKLNDLNFSIHNESFHFDEQHSINMGYEVLSWTWPHKKTEIVSIGSFHGNL
SINESMVHFHTVDQKAPLSECLTKCSSGQIRRMKGFHLCCYDCIDCESGTFHSSSDDSTCTPCPEHQ
WSPKRSTQCLDRGEKYIFWSEPLAVCLLGLLLVAFVLTCLSGVLFLKNLQTPAVEASGGGLCLVALLG
LAATCISGVLFLGKPSPTICRIQQPFFALCLNLCFSTILVKALQIMLVNDFADSRPNVLHTIIQRHPSVL
ATVSVLAETVLCVVYLYATPTLLIQNYKLLPEEVLLQCQVQSWLTFATIHGLNGIVAFVSFICTFMVQ
SSPKKYNIARAIAFAMLTYFITLIIFIPTYATVKQVDQPAVQIGAILLCTFGMLTAYYLPKCYIIWFKPEW
NTQNYFQDYTQNRIQGKDT

>Ambystoma_mexicanum_T1R3A1_pseudogene

IRPPQSSALRSHPPRSHEINNSTSLLPGIKLGYEIXDTCFEPLVAMQPTMLFLTSANSTRVDVLCNYTD
LPRVIAVVGPMTSQLCLTVGKLFSFFRIPQVSYAAGRDSLNDREFFPSFFRTIPSDRNQMEAIVEVIRFF
KWNWVAVIGSDDDYGHGGLELFSSLTSSQDICTAFESTIPMSMERLVETLKPINKSEANVIILFADEVA
ACPLLTEWLRLGXSVIAPLILSIPGIQNIGTILGFTAQGGHIPGFADYVVNFLSAASSESLCWTSRDGFF
PSADSGTSQIPQCRGCDYISLQNISSSLESNYTYRTYTAVYRIAHSLHELLKCDAGACLRMQNSKPWQ
LLEEGKKVQFRLNNDSFYYDVYGNLNTGYNILFWYQSNMSVQYSTVGAYTQGTLHLDHHRIRWHT
RDNXAPRSMCNDKCSVGQIRRVRGFHSCCYDCVDCEQGFFQRGPDEVCTPCPAQTWSPAKSSQCY

NQVLQYLFWKDPLAVLLLALTSLAILLILAALIVKNPHTPLVEALGGKMCXSATLLSLMAACGTGGTF
IGKPNEYTCVVQHPVLAISLTACLASLLVKSLKLFLASEFPHSPRSYXEWLRGKGGWLIFSLCLLIQCAI
SFXYANVVPAKVQTTHRETVQEIQLHYASQSWLGFGLLLLFSGLLACFCFLCTFMVHASPRRYNLAR
GIACAILMYFIAWIFFLPTYSTVQPSFQPAVQVCVILLCTQGILGSFFLPKCYLLLFRPERNTAACFGSS
TEEPPNTDVPERAARITNS
>Ambystoma_mexicanum_T1R3A2
MRTLSPLLFLAFWGPWASGNLCTSSLFRAEGQYMLGGLFTFGASSKDLSKRSQPELVPSDRFYAPGF
VLAFGMRFAIDQINNLTSLLPGIQLGYEIYDTSFESLVALQPTMLLLTRKNSSGVDVLCNYTDYEARVI
AVVGPMTSELCVTTGKLFSFFMVPQVSYTASSGSLSDRLLFPSFFRTIPSDKNQTEALVELVRLFKWN
WIAAIGSDDAYGQQGLSMFSSLASSQDICIAFEGIIPNTLTSPLALVSLQETIQQINRSQVNAIVLFSSEG
AARLLFEEWIRLGMGSKVWLASEDWVTSSIIAAIPAIQSIGTILGFDVQGRQIPGFHDYMSHTLAAAPR
QSLCWTSSDGFFPSTDSDSPETLQCQACDYISLANVSSSLGSSGTYRVYTAVYSIAHSLHKLLGCDSRR
CRLTPVPRGWQLVEELKKVQFQINDQQFYFDAFGSINTGYTVVLWSWRNRSLHYLTVGNYTQTLYL
DKGAIRWHTKDNMVPQSMCNDKCSEGQIKKVKGFHSCCYDCVDCKEGFYKNDSEGEVCVACPALT
WSQPRSSQCYNQVLKYLSWDHPFAVLLLVLIFVVILHILAIAGLFFKNRHTPLVEVSGGKMCFATLLSL
LLACVTSVTFIGEPSEHTCAIQHPIMALSLTACLASLLIMSLEIVLTADFHFSARSYIEWMSGKGRWLIW
ALCLLIQCAICFWYAIMVPPKVQTTHGKSNQETLLHCAIQSWLGFGLLLSFSGSLACVCFLCTFMVQ
KPPRKYNLGRDITCSILMYFIAWIFFLPTYTTVEPSYQPAVQVCVILLCTQGILGAFFLPKCYLLWFRPE
RNTAASFEIESNDSPRMDATK
>Neoceratodus_forsteri_T1R3A
MFFGGFMLCFCIMRTIAAENVDIFKEFKSPGDYVLGGLLTFLMSPVNIANRSTPELVTCERLYITGFRA
GIALKFAIDEINNSSSLLPNIRLGYEMYDTCSESLVTMQPTMLFVTKNDTNEVQMLCNYTEYETRVIA
VIGPAQSKLALITAKLFSFFRIPQVSHGATSDKLSDRQQFTSFFRTVPSDKNQSQAMVQVIKQFQWN
WIAAVGSDDEYGQQGIQTFASLASTDGICIAYQGLIPVQLSDSQMYDPQIAEILNNINISQVNVIVLFS
GDQPAQALIRQVINIGLNKKVWIGSEGWVTSELVFSLPGVENIGTVIGFVTKGSPMPGFDEYLLNTVA
QLGLKNNFTGTFTAVKRQAFNVYTAVYSVAHSLHMLLNCTSGACRQQPKVKPWELLMKLKKVQFPI
NNYIFHFDDYGNPNTGYEIITWNWQEMNVLFPMIGEYNNELFINTSLIQWHTDDNKIPESRCSKKCE
AGQIKKVKGFHSCCYDCINCLPGQFQHITDDEACSPCPVYQWSPEKSTICYNRSLTYTFWSDLAVIVL
LILIATSLTLIFIVTVLFFKKLDTPIVQASGGKMCFITLLSLFSLCTSSFSLIGKPSDFICKVQQPWLFISLT
LILSTTLIKSLQIVLSIEFPSLSRSYIKWLKGPGTWIIITVNICIQNVIWVWYLNTGPPFLNQDYDMSPSEI
FVYYDINSFLSFCVMLGHNGCLAFTCFLCSFMVQNPVRNYNMARGITFSMLGCFICWIFFIPTFAIIQY
KFKSAVQMYTLTFCSLIITGAYFVPKCYIIVFKSELNTAAYFQRYILDSHAITEDGPTTKEDSHTTNESA
ITVDHPH
>Protopterus_annectens_T1R3A
MKLACGFMLCIYFRWTNAVDDAEICKQFTTPGDYILGGILTFLMSPVNLDNRSVPELVTCGRLYSTG
LRAGIAMMFAIDEINNSTSLLPNIKLGYELYDSCSESLVAVQTTLLFLTRNGTNKVPMLCNYTDYNSR

VIAVVGPAQSTLALITARLLSFFLIPQISYAASSEKLSDKEDFPSFFRTIPSDKNQSAAMIEIVHHFHWN
WVAVIGSDDDYGQQGIQMFASLASDASICIAHQGLIPVQASNTQTVDSKIPDILYNINKSQVNVIVLFS
GDQAAMPLIKQFIKSGLDKKIWVGSEGWVSSDLISSIPDVETIGTVIGFVTKGSVMPGFDDYITYTVA
QLGLKSNITGMYDAVKRQPFNVYTAVYSIAYALHELLNCTSGVCRQWPKAKPWELLEEVRRVQFSVY
NDTFQFDYNGNPNTGYDVITWKWNNGELQLTTIGEYNSELYINQSLIHWYTGSNQAPQSVCSQKCE
AGQIMIEKGYHSCCYDCISCMDGYFQNLSDPLDCTACPDSQWSPKKSTVCYDRTLIYTFWGDPAVIG
LLIMLSISLILIISVIILFMKKLDSPMVQASGGNMCVLTLFALISLCTSALSFIGRPTDTICKIQQLWFSTS
LIVVLSTMLIKSLQLLLSTECQSLPVSYIKWLKGPGTWFIIFFNICIHYAIWGWYFYTGPPQVKEDYSTS
PSAIVLYCDINSFVNFCIMFGHNACLAFICFFCSFMVQPPVKTYNMSRGITFSMLGCLLVWTFFIPIFAT
IQCKLKAAVQMNTIMLCSLIITGLYFLPKCYIILFKPEKNTISYFLQHSMNLRSVNEGATTSSEESHRSN
STMTENQPQ

>Ambystoma_mexicanum_T1R3B1

MSLSPVHALAFFLGFEMLPGPIQSNDTLAYQIFHSSGDVMLGGLFPFHTGVSNASNLWRPEPLTCINL
NYVGVVQALAMRFAVEEINNSSMILPGRRLGYEIYDTCLQARVLMHSAMLLVSEGLTRDIWAICNLT
TYNPRVMAVIGPGSAGIATPMVKLLSTFLIPQISYAITAAKFSNKNTFPSFLRTVPSDQHQVQGMIELV
AHFGWNWVASLASDDEYGRDALCQLANLALLRNICIAYEGLIPTYGATSKTPVVIRDIIQEVHRKNIN
VVILFAPASLSRILFQEVLQLNMTKVWIASSAWVLSEPVLSLPGIREIGTVIGFSHRGSSVPGFLEYLVSV
NAELKHHKSTLSSVGFGTSTLMGYDEEGDGSSPNFAVEEMSFIRLGQYADAVYTAVYSVAHALHEVL
NCSSGKCRDVDSKIYPWQLLEAVKRVNFTVLNTSFYFDEYGNPNSGYDVVTHSMDNMGYKKIGRYS
GQLDINDSLVNWRTNDNQVPTSQCFSRCEPGQVKLFKGLQFCCFDCDDCSEGTFQASYDDFYCAP
CPVGQWSHKRSTHCSYPTFVFLKWSDAPVIILLIFAAVLLVLIASVALMFFRHRHTPVVQAAGGNMSF
LTLFALSVECCSAVVFVGWPSDTMCRVQQPFLFLSYTVLLSTFLLKSLQVALVADFKKVPRTYLHWL
KTKGTWTVLGAIILGEGLLSVWHISKATAPWPSANEKVTFLSRYLECGLWPLASWGFMFAYNAILALL
SFMCSCVSQKPLKQYNLARDITFSMLGYLVIWFSFIPIYGQVEREFNNILQFTTTLACTTWIIVSYFFPK
CYILLFKPDLEMTEYFRIYLE

>Ambystoma_mexicanum_T1R3B2

MPGWVYLLIAAFHATGYGSEAWDLINDELFTLPGQIRLGGLFAIHTKANITYNESQLGPLTCHSFNL
HGFVEALAMKFAIEEINNSSSLLPGVRLGYEIHDTCLESVVILRSVIRFLGEGNSTEIQVSCNYTDYRTR
VAAVIGPSTTEMVTATGKLLGFFHIPQISFSASSERLSKKIVFPSFLRTVPSDRLQAQAMVQLLNSLQW
HWIVIVGSSDLYGQEGLYQFSLKANQEGVCIAYEDYLPEDGSADAFNKSMQIIEKIRKTRANVTVLFAS
HDQSQAFLAIALSAHLQMVWIASTGWSLSSTIRLMPGVHRLGTVIGFAVKNQPMPGFYGYVTGILSL
LAAQAQMASQNRSTGECNDGPYIKYEQQEFQTTCMECSKLTPANISMINDSMTQSLACNVYTAVYS
VAHALHQMLHCSSGAHCQLDTYSRAWQLLNTLKNINFTINDRAFSFDENGNPNIGYDIVTWTASRS
GVLFNTIGSYTDQLNIHNDQIQWHTHNEKPNSTCSKVCMEGEIEVVKGSHSCCFDCIPCPEGSFANT
TAFSPVCSPCPDGQWSRPGSIACLPPSYTSFTWTHFFIITLLGVKVAVLLLVIAVALLYLNHRCTPLVH
ACGRSLSVLALAGLAAMCCSIVLFVARPSDLICQLQQPFFAVALSACLASFLVQSLRIAACTSIPWQSGS

RIHWLVTRGAGLLVLGLLLVQVFLCGMFVQASPVLSTKMAAMSVKSLNISLNCSIDRFVEFSFMFGYN
ALLVLFSFICSFLSEKPVRQYNLARDITFAMLIFLSAWIVFIPCYASATGQVKALIQATVILFSCLGVLAT
AFMPKCYILLFKTELNTPEYFAAYLPHSPGQKDLDSEGPGTQ
>Nanorana_parkeri_T1R3B2
MSTFGALAILLLLTCPLVCPLLKTGDSTSQTFFKIPGDFKLGGLFAIHDEADTLDNWGKNGTTKCKN
FNIHEFIGLLAMKFTVEEINNSSTILPNSSLGYEIYDTCCNTEATLHAALKFLSEREDSVIQVVCNYTYY
EQNVVAVIGPSTSETIAATARLFGFFRVPQVSYQVSSERFSNKVIFPSFLRTIPGVITLAQGIVNLLKEFK
WNWVAIVASKNDYGDQGLFLFMTLAAQAGICTAYQAYIPNDSTNSNFNTSLQNNILELQNTGVNVT
IVFSTEQESKLFFKAVIDSQLKMVWIATTTWSQSTSLQQMAGMQSIGTVIGFSETSKALPGFEDYVQH
ILHLIQQQRQLLLNGSTSNPNISQQYVFQTKGLLEQCESCSLLTPNNITILQDPVVLGLAYRVYIAVYC
ISQAIHNIVHGLDGQCKDVYGILPWQVLQELKSPNFTFNNITFDASGIISMGYDILTWRYNTGKPFYT
VGTFWKKLTIKRSQIAWYSIKVPESTCSRQCSEGQVKLIKDFKSCCFECLTCPEGTLVNSTECTPCPSG
QWSRSGSKACQDPTFIFLTWKNYYVIALLIFMGLIMVIIGTVAVILFQHCHTPLLVASGEIESFLTLLGI
ACMCSSIFFYIGEPSDMICLLQQPVLSLSFTMFLGPILVKSMQLQFSSFSIGSCLYWLLYPGRWIILVCAF
LGQFFLCAMYVKSSQPFSVKVASLDVSSLTIFLSCKYEPLLQFGLMFAYNGLLVLLSFLCSFMAEKPVH
QYYMARDITIAMLTLILDWIIFIPTFVSTNVAYKSIIQMIFILSSCLGVLCTVYFPKCYILLYKKEMNSSGY
FSTYITNSQSEKANE
>Rhinatrema_bivittatum_T1R3B2
MFPFLRLGVIFVAVLKTSVPGLRKNSTCAELLTLPGEYRLGGLFAIHNQANVLYNRSRPELVACESFN
AYGFSCALAMKFAVEEINSASSLLPGVQLGYEIYDTCMEPVVILNSAMRFLQQENDSAIHVLCNYTDY
RTRVMAVIGPSTTEMIGTIGKLLSFFHIPQISHIASNEKLSDKIVFPSLFRMMPNDRAQAQGMVQLIRE
FQWNWIAVVGSSDEYGRQGLHQFSRQAVQNGICIAHMAYIPGDQSKGTIQEILGRIRDARVNVTVLF
ASPYQTHAFLTQTLAAELKMVWIASTGWSLASGIQQIPGIENIGTVLGFTVKSNIIPGFKAYMTKVLSL
LEVEQFSVESNVSNACKKDQGLSKIQDFYTSCATCSKFSADHASMMWDLMTQRLTFNVYAAVYCVV
HALHRVLQCSETKCEKGFNLYSWQLLKEVKKVNFTLHNSSFSFDENGNRDAGYELLTWLAGKDRAQ
VAVIGEYQDKLTLNKSLIQWHGNGKEPQSTCSKECSAGQIRIVKGFHSCCFDCMTCPEGTLVNSTDC
TPCPAGHWSEPGSTTCSLTSFSFLSWDQGTVSILLAFMAALVLLTIVIGWLFISHWHTPVVQASGSYL
NLLMLVSLAAMACSICLFIGEPTDLLCQLQQPFVSIILSTCLAIFLVNSLQILISTDLKSLSRTRLHWFLH
KGMGLLILCIVLGQALLGGLYVKASRLFSEEQANMPVQSLHIFLSCTINPLMEFSFMFGYNGFLVLCSF
MCTLLSEKPSHQYNMARDITFAMLSFIFTWIVCIPTYAGAALEHKPVVQMAIILFSCFGILLANFLPKC
YIIVFKQELNMSEHFEAYVTGGPAKSDLQ
>Nanorana_parkeri_T1R3B1
MMLYAVLSLGFSVRAILGDEPVAYQVFRKPGDIMIGGLFPFHTTVNGLDNYWKPEPLTCLSLNPTGF
LQAIAMKFTLEEINNSTTLLPGWTLGYEIYDTCVNTLVALHPVLLLLTKNGTEHMEMKCNLTYYRTR
VIAVIGPSSNEVATVIMKLFSAFLMPQISYSVTSDVFSDKSTYPAFFRTVPSDSKQVNGMVDLMTQFK
WNWIAAVASEDDYGVSALQQFSSSAMGKGICVAYEGLIPEITSSSDTSTVIEDILDKIDQADVNVVLVF

SSLTQSIALFKEVIMRNMTKVWIGSASWVLSEAIFSLPGIERVGTVIGFFPNGNYVFGFEEFLKNAISQIY
PSQSALESLSLGTSEGYQSIDLNPNIISSILNPLTALYAHSVYTAVYAVAYALHSLLNCTSKNCNNQDST
LYPWKLLDEVKKVNFSIFNTSFRFDSNGNPNTGYDIVTHSLSKMGFITIGSYDTKLKLNSSLINWGTK
ENQVPVSQCSGDCLPGQIKRVKGTHSCCFDCIDCQEGLFQSSGDDFQCQSCPAGQWSSVRSTSCSY
PTYLYLQWSDTSVIWLLLLSFVLLCFVLGTLILFFKHRHSPLVQASGGCMCFIALTSLVAVLSSMVLFI
GKPAHVVCLLQQPFLAMSLTCCLSTFSIKAVQVMLVTDFKDVPTKFIQWSKTTGTWVSFLSGILIQG
LFCTWHIVSTVNSPENNEVTFLYKYLKCEIPNILAFFLMFGYNGSLALISFMLNCVAQAPPGQYNLAR
DITFSTLSYLLIWIVFVPAYAEVTDGSQSLLQMAVTIISSFGIMLGYFAPKCYILLFNPQMASEEYFKIYN
N
>Rhinatrema_bivittatum_T1R3B1
MHLCSILQVVAALLGCGFVTEVTGAKEQVAYQIFQQAGDCTLGGLFPVYSSADSLETHWKPEPLSCR
SLNSAGFVQALAMKFTVEEINNSSTLLPGIQLGYEIHDTCLQSVATMQSSLFFLTSHGTQSIEVLCNFT
QYRTRVVAVIGPSMSEMVLASVKLLSFFRIPQISYAVTSETFSDKKTFPSFLRTVPSDRRQMEGMIQLI
NHFHWNWIAVIGSDDDYGRGIRQLSTLATPKGICIAYEGLIPLYLASPKTEQVIREILNRVQQTRVNV
TVVFASVTQAKALFQEVMRQGLTKVWLASASWVVSELILSLPGIDRIGTVIGFSPTTQTVPGFEKYVS
HATEKLRQDAPANPSTMASKKSNYYEGPNPFLGLPGTLYPLLGHYAHSVYAAVYSVAHALHRVLKC
QAGRCQKASSKLYPWQLLEGVKKVNFTILNTSFYFDENGSPNTGYDIVTWTYRGKELGFEKIGQYPA
ELDNINSSLINWKTAEKKVTVPPSQCSKVCKRGQIKIIKGFHSCCFDCIDCLEGTYQTGEDDLKCTPC
PDGEWSNQGSTSCNKPTFSYLEWSSTLAIIVLILTAGLLLLITAIILLFWKHLHLPIVQATGGNMSFLTL
LSLMALCCSIGLFIGKPTHLICQIQQPFLALGITTCLSTFLVKSLQIIIVTDLKYLPPFYLHWLKTRGTWI
IIILSILGQCLLCIWNIRTSTPLPSEYFPVTFLERYLTCDKSPLLNFACMFGYNGILGFSSFVCNCLAKKP
LKNYNLARDITFSMLGYLVIWVIFIPVYAKIKDETRSVLYMVTVLTSTFIITAGYFLPKCYIIIFKLESDM
VDYFQIYIN
>Neoceratodus_forsteri_T1R3B_pseudogene
MFQLSPQILLVFLLLGLHGPTHSPVMKPPQKCLKLXGDYILSGLFAIHSTANDLDKQQKPEPIGCSSF
NPSXLAAALAMKFAVEINNSSSVLPDATLGYEIHDTCLESLVASARHVVLNXEQKPRVEILCNYTDYR
THVVAVIGPPTPEMASITGKLFSFFLIPQISYAATSDKFSDKNAFXSFFRTVPTDKKQAEGMVSLITQF
QWSWIAVVGSADEYGKQGLKQFSALASQSGICIAYEGYIPVYLPEVKTNEVMLXIIEQINLNQVNVVV
LFASAIQSQALLEYVLKCNVTKVWIIAHHGAVXRQLHPYXESAXVGTIIGFFHKTESMPTXEQYVRCW
VTVMSRAGIWHYLQYIQQLFHFCSINCXTGGHDLQMGHLFXSFSSKPLQHFKTICNEENAYNVYLAX
AIVAHALHKLLNCDSTKCQQTSKIYSWQLLQKXKNVYFMMNNTEFYFDGXWNXNFGYDIIVWTSTS
KXESVYKIGEYNSTLSMNQSLINWHTENNTVPQSQCSRDCXAGQIKIVKGFHSCCFDCIDCPEGXFQ
NVKEDFQCTECPAGQWXSSIKSTNCSYPTFIYLRWDNHYSIVLLVATVVILVLIAAISLLFLKICTPQLF
XASGGIMSLVILLMLALACCSMIFFIGKPTDLICQVQQPLSAISLTICLSIFLLKSLQIVLTTEFTSSSEVYI
QWLRSTGSWVIVTVNVMVQISVCVWYLKQTVSLSXIYLNILSSSKFLICDARPLLGLGIILGHNVLLAL
VSFMCMCLAKKPVKKYNMARDVTFSMLGYLVPWTVFILAYAGTVSLNKSLVQMTVTLVSCFGIITTY

FFPKCYILMLKPDLNIVEYFSIYI

>Latimeria_chalumnae_T1R3Ba

MRGPSAIGFIALMAYGFLRSGATEEESTFTRLPGNYTLGGLFAFHRSAVDLEKRQKPVPIACESFNPSG

FLQMLAMKFAVEEVNNSSALLRSVKLGYEIHDTCREPVVAMQPAMLFLSQNGSRSIGILCDYADYRS

WVAAVIDPSTSELAEITGKLFSFFLIPQISFSATSEKFSDKSLFPSFLRTVPSDKNQVEGMIKLVAEFKWT

WIAAIGSDDEYGKQGMRQFSKQASEKGICIAYENFIPVYSSDSETKQVIVDILDQLNKTQVNVVVLFAS

EPQALTLLEEAMRRNTVKVWIAGRSWVVSDPLVLLPGVGQVGTVIGFSQRSEEVEGFEQYVKNTFAA

LEQDSPSRGYCNLSINSLSSAPLNDEEDWTIMQSIICQGSFSDNMAMVLNLPVRHYIYNVYLAVYSAA

HALHKILNCNARNCQQKSSMYSWQLLEEVKKVQFTLDNVTFHFDKNGNPGSGYDIIVWSMGSSAVH

FTKVGQYNGMLVINKSKILWHTQNNAEPQSQCSKECQKGQMKRVKGVHSCCFDCIGCPEGTFHAT

NDNFECKPCPVGQWSTAESTSCSDPTFLYLSWDHYLTVILLLGMVNLLVLIATISLLFFKHLHSPVVQ

ASGGRLSFFTLFSLASFCCSVCFFFGKPNELICWVQQPYFAISLTACLSTFLAKLLQIMFAAESTGSPSA

VLCRLRARRPGLIIPLTVLGQLLICIWYSTQTSPLSSTSVIIRSLSKFIMCDVSPLIGLGLMIGYNGVLAL

TSFLIAFMVQKPAHYYNLPRDITFAMLGALVAWIVFIPTYAGATASNQCIVQGAVILASSFAMTVAYFL

PKCYILKLKPELNTVEYFQIYI

>Latimeria_chalumnae_T1R3Bb

MELSRDSLVKLSMLRMMVSLSAMACGSLQVEAKECDKFSPLIYTKEGDYILGGLFPVHEGISNISQRM

KPDQITCKSFYISGFAQALAMKFVVEKINNSTSLLPGVKLGYEIYDTCQESVVAIQPTLLFLTKDTSQG

IEVLCNYTDYRTRVAAVIGPSASHVTSVTGKLLSFFLIPQVSYDSSSVIFNNKINFPSFFRTVPSDSLQSE

GMISLIKMFNWTWIAAIGSNDVFGKTGLQEFSKQALRNNVCIAFQDFIPVYETQIGTDQAVKKMVKE

IEKVNVAVVVLFASSFPARVFLQEVIQKGMKMVWIAVSSWSLSELTTMLPGVESIGTVLGFAVKANKL

SEIEDYIKEAFSPENQGAEWKSSSPAAAGDNTGLPPNSTTAVLKDCSYRLNLSSVNMSILLGSNVNHL

MYHVTTAVYSIAHALHNLLKCNSTTCQKLSKIYSWQLLDEVKNVNFSINNSYFYYDKNGNPNIGYDIL

SWTFEKDGKDSGFQFIGDFQRKLNINKSLIKWHTKNKTVPLSQCSKECSPGQIKRVKGFHSCCYDCI

NCQAGTFQSSGDYNQCKDCPEGQWSAPKSTSCSPLTFIFLTWGNVYTVIILIAVAVLLALIAAVSVLFF

KHLDSPVVQASGGKLNFFTLLSLASLCCSVCFFFGKPNDLICWIQQPYFSLSLTACLSAFLVKSIQIVFLI

EFPNLPKSYIHWLSNSGVWVIISANLLIQAIICASYLHGSERLSTYLSSLQIRSLSKFMTCTRDFLSVGLM

IGHNGVIALVSFMCTFMAQKPAQQYNMARDITFSMLLFFVAWIIFIPTFAAAVDAGKSFSQIGAILGSA

LGILVSSFLPKCYIILFKPDLDVVEYFQIYIQ

>Takifugu_rubripes_T1R3Ba

MAVSPTLLVLFWVFKMTSATPAWFQNISTSLFNLPGDIKLGGLFPLNRLTSNLSQRTEPDQISCDRIN

TYGLGMAIAMKYTVDEINANQILLPGIQLGYEIYDTCLQSAIIVRPTLSLLSAKHDNTFSVQCNYTNYE

TSISAVIGPNNSEMVSVIGKLLGFFLMPQISYGATSEKFSDTALYPSFFRTVPSDKWQVEAMVLLLEEF

NWNWVAVVGSDEEYGQRGVQDFSKLAANKSICVAYQGLIPVYTDPEPMVKTILSNINSTKARVVIVFS

LSNQAEIFFKEVIRMKLKGVWIGSTSWTINDAVTSLPDIQTGWTILGFVEQTQSVDLLRAYTYALLNK

LSEERAHTRSSAQNSNYPSNPCPQCWNLSPANISLVTDLVIQRKAFSVYAAIYSVAQALHNFLQCNST

ACKNTSEVKIYPWKLLKTLRHTKVDINGTMLEFDSNGNPNVGYNLIELIWKNSTLEFVEVGSFNKILN
INVSLFKWHTETSEVPQSTCSAACGEGQVHRVKGFHSCCFDCIDCLPGTYQAQDGDIQCTPCPPRQ
WSLARSSRCTDPIYDYLSWDTPEALLLTLAIVLVVLFMGSVVVVFVNHRETVLVTASGGTLSIVVLLG
LMGACLSLLLFLGQPGDTVCRLQLPLISAFQTVPLSIIMSISLQIFFVSEFPNLAASYLHVLRGPGTWLL
LLTCCAVQAGICGWFVQDGPSLSEYLADRRVDFVRSFLACPVSPLSGFALMQGFSAAMALMSFMCTF
MATKPLHQYNLARDITFSSLIYCVIWVTFIPIYIGLEEKRRAIIHVSFILASDLGLVAVYYIPKCYFLLKTP
ELNTADHFCTFLEGLQPTPAQEEPQTQTESEQ
>Takifugu_rubripes_T1R3Bb
MAVSPTLLVLFWIFKLTSATPAWFQNISTSLFNLPGDIKLGGLFPLNRLTSNLSQRTEPDQIICDRIDT
YGLGMAIAMKYTVDEINANQILLPGIQLGYEIYDTCRQSAVIVRPTISYLTAKSNNLLSVECNYTNYET
SISAVIGPYGSEMVSVIAKLLGFFLMPQISYGATSEKFSDKVLYPSFFRTVPSDKWQVEAMVLLLEEFN
WNWVAVVGSDEAYGQRGVQDFSKLAANKSICVAYQGLIPVYTDPEPMVKTILSNINSTKASVVIVFSL
SNQAEIFFKEVIRMKLKGVWISSTSWGIHNQVTSLPNIQSVGTILAFTDVTQNVDLLDAYTHALLTKL
SEERADTPPPAQISGNPSNPCPNCWKLSPANISLVTAPVVQRSAFSVYAAIYSVAQALHNLLQCNSTA
CKKPVKIYPWKLLEALKNISLNLSGTQIEFDASGNPNVGYNLVEWVWTESGLDFKDIGSFNEKLEINK
SLFTWHTENSEVPQSTCSAACGEGQVHRVKGFHSCCFDCIDCLPGTYQAQDGDIQCTPCPPRQWSL
ARSSRCTDPIYDYLSWDTPEALLLTLAIVLVVLFMGSVVVVFVNHRETVLVTASGGTLSIVVLLGLMG
ACLSLLLFLGQPGDTVCRLQLPLISAFQTVPLSIIMSISLQIFFVSEFPNLAASYLHVLRGPGTWLLLLT
CCAVQAGICGWFVQDGPSLSEYLADRRVDFVRSFLACPVSPLSGFALMQGFIAAMALMSFMCTFMA
TKPLHQYNLARDITFSSLIYCVIWVTFIPIYIGLGEKLRSIVYVSFILASDLGLVAVYYIPKCYFLLKTPEL
NTADHFCTFLEGLQPTPAQEEPQTQTESEQ
>Danio_rerio_T1R3B
MLLLRMKNKWTFLVLCGILGSGLGDNPSWFNNITTNFFKSPGDILIGGLFPINQLTSELSQRVKPDDL
QCDSISTYGLSLSLVMKFTVDEINSKKHILPGITLGFESYDTCMQPAVIMKPVLQLLTQESTDELDIYC
NYTNYKPRVMAIIGPDSSDVVPDAGKLIGFFLMPMISYGATSDEFSNKQTYPSFMRTVASDQWQVVA
MIQLLKQFGWNWVSVIGSDEEYGQMGQQQFSSMANDESICVAYQGLIPVYSDPGPTIQDMLNRIVD
AKVGVVVVFSIPIPAKAFFTEVIKRNITAVWVASTAWSLNDGVSTLPGISSIGTVLAFADITRPLDLFTP
YIRELFTKIEGMAIPQQPDADISPLDNPCPRCSYVSQANVSMVEVDLVQRSAFSVYAAVYCAAYALHD
LLGCNATSCTRNPKRDNVYPWQLLRKLQKLSLDLEGVNIQFDDEGNPNFGYDFMQWIFNDTTVTF
DVIGYFYQNLTIESNAIKWHTKNGEVPMSTCSSDCGIGQVRRVKGFHSCCFDCIDCLEGTFLNNTDD
IQCKSCPNGQWSTLRSTSCVYPIYTYLDWTNYESIGVILGGIVVLASHVWVGALFFKHRGTPLVKTAG
GSLCGLTLLSLAGGCMSLVLFLGQPGDTVCRLQEPLNAFFPTVALSVILSSSLQIVCVTEFPEQSSEHL
ENLRGRGSWFVILGCCGLQAGLCGWYGLEGPSLTQYVASLDVTYVKTFLRCPVEPMLNFGLMLGFN
VILALMSFMSTFMALKPPGQYNLARDITISTLSYCVMWVMFIPIYTSLDDKNKSLAQVGVSLLSNMGL
VAAYFFPKCHLLVKQPELNTDDHFRTFLEGVPPTPPEES
>Lepisosteus_oculatus_T1R3B

MEVMLRMLFLPSVLWTSPTADPLHWLKNISTDLFRSPGDIILGGLFPIHELTSNLSERSQPDDVYCDR

LNTYGLSRALVMKFAVDEINNTPDLLPGTRLGFEVYDSCKQASVIMRPTMLFLSEKHSRGIRVLCNYT

GYSTRVLAVIGPSTSEMVSITGKLFSFFLMPQISYGATSEKFSDKMLFPSFLRTVPSDKLQAEAMVHLV

LEFGWNWIAVVGSEDEYGKQGVLQFSMLAAQQAICIAYEALIPIYSDPREAVDDILNRINQTKAGVVV

LFTLTVATKALFSQVIQKKMKAVWIGSTAWALSEDITTIPGIQTVGTLLVFSNKNTQLSHFEIYAQQL

FTLLEQERLQANSLDPPTSPSATETEFPLPEPCPDCWNLSQANMTMIREPQLQRTAFSVYSSVYSVAH

ALHQLLGCSATLCRRKDAEFFSWQVLEVLKKVSFTINNTKVTFDVNGNPSIGYEVLTWRWDRDVLR

FQNIGTYEGKLVLNRSLIHWHTENSQVPNSTCSPNCTAGQVRRVKGFHSCCFDCIDCKEGTFQNNT

EDFQCTDCPDRQWSTLRSTRCTDPTYHYLKWNQYESLGLILGAVLVLAGQAAVGVLFLRHRGTPLV

QASGGPLCGVALLSLTGGCASLSLFLGKPGDAVCRLQQPFNAFFPAVALSIILAISLQVIYVTEFPGVT

PSRLDSLRGPGSWMVVLTLCSVQAGLCGWFVQEGRPLSEYVSRMRITFVETFLRCEVEPMVGFGLM

YGFNGLLALISFMCTFMAQKPAKQYNLARDITFSTLAYCVVWVVFIPIYTGLGEMNKSLAQMAAILLS

NLGLVAAYFFPKCHLLLTHPELNTVDYFRTYLEGAPPPQDGQEQ

>Amia_calva_T1R3B

MQWVLRMLLLSSVLSPGHAEDGFPAWFKNISTDLFWSKGDIMLGGIFPINELGNSSSSQREQPDDVH

CDRVLSYGVSQVLVMKFAVDEINSSPLVLPDTSLGFEIYDSCRQPSVIMQPAMLFLSEGKSQSIRVLCN

YTGYSTRVAAVIGPSTSEMVSITGKLFSFFLMPQISYSATSDKFSDKKQFPSFLRTVPSDRRQAEAMVQ

LVLRFEWNWIAVVGSEDEYGKQGLRQFSLLASQQSICIAYEALIPVYTSSKATIKDILQRINQTDVGVV

VLFALPSAAMDFFSMVISSKMQKVWVGSTAWALDKNLVSMEGIRTVGTVLAFADRNQKLELFENYT

YAFLSRLEREQLNGTSNANSTKTVDFPLADPCPSCRGLTLQNISMATDARLQRTAFSVYTAVYSVAH

ALHKLLKCNQTRCLKKGSVLYSWQLLEVLKTISFDINGTQFKFNEEGNPSIGYTVLTWIWGKDQVEF

QDIGSYVEKLELKKELIKWHQSTSNEVPKSTCSANCQTGQVRRVKGFHSCCFDCIDCNEGTFQNRTE

DFQCTDCPVGQWSTPRSNQCIDPTFHYLEWNQNESLGLVLGAVVVLACHAAVGWLFLRHWGSPLV

CASGGSLSALALLSLSGGCASLSLFLGQPGDVVCRLQQPLNAIFPTFALATVLAISLQVIYVTEFPGAT

PSRLESLRGLGSWLVVLAVCSVQAGLCGWFVEEGQPHSQYVARMKVNFVESFLRCEVEPMVGFGLM

YGFNGLLALVSFMCTFMAQKPAKQYNLARDITFSTLAYCVVWVVFIPIYTGLQEKTKSLAQMAAILLS

NLGLLAAYFFPKCHLLLTTPDLNTLDYFRTYLEGAPLKQEDSQENQGDQGGANGQGAQEK

>Polypterus_senegalus_T1R3B

MSMQNDSCINGNLSAQLFSEPGDYILGGLFPIYSSIIDMSTSVKPGQFKCQSLNWYGFIRALVMKFAV

EEVNDMKDLLPGIRLGFEIFDTCMESVVIMQPSMLFLSEQWTQAIRVQCNYTSYQTRAIAIVGPSNSE

MMAITGKLFSFFLIPQVSYGATCDIFSNKNLYPSFLRTVPSDKLQAMGMVKVVSHFKWNWIAVLGSE

DEYGKQGLREFSNAASQASICIAYEGLIPVYTDPQSAINEILDRIKQMEVEVVVLFSTARTAQAFFTQVI

RRGMKKVWVASSGWSIYQDVSLLPNIQTVGTVLGFIYKGVQLGRFELYVQKLYKQLKMEQKSSWSN

DSSLLAEYFGVSGQVCPECVCSFNLSVLSQPLVKLTAFTVYAAVLSVAHALHRLLDCSATRCKGVEQQ

FYSWQLLKQLQNTSFQLNGTLFQFDANGNPNIGYEVITWVWGETPIFTKIGEFNGNLSIQSESIQWH

TTNKKVPESQCSKDCQSGQVRRVKGFHSCCFDCIDCAAGTFQNQSDDFQCTACPQGQWSLMSSTN

CSYPVYTHLDWSDCASVVLLLLTSALLVGTTIVTLLFIKYWRTALVQASGGPLNILMLLGLKASFCSLA
LFLGKPNDLVCRLQQPLTSFISTVMMSSLLASSLQVTCVTEFPGIAASYYSLVRGVASWLVVALALIVQ
AGICSWHILNSPLLSVWITNKYVHFLTAFLRCQVDPMTTFCLMLGFNGLLSLLSFMGTFMTQKPTK
QYNMSRDITFAALAYCITWVIFIPVYTALSEMTKSLAQMVAIILSNTCIIASYFVPKCHLLLSRPDLNTSE
YFHIYIEGVTPKSTEDESK
>Callorhinchus_milii_T1R3
MSPLRLLTLLGLIPCGCSHPKTQGLGVTMAHFSLPGHYTLGGFFPIYSVAVNLANRTRPEPVSCEMFS
LDGYEWALAMTFAIDEINNASSLLPGVTLGYEIYNSCHDFLVAVQPTMRFMSGADNSIAVLQDYTRY
QSTAIAIIGPATSDLAIILGTVFGLMLIPQISYGASSETLSNKDLFPSFLRTIPSDKNQAEGMVALVKTFG
WNWIAVVGSDDEYGRQGIELFSVRAAVEGICIAHIHLVPSYNSVSSVELKMATIIGHIQQSQVNVIVLFS
NEKVALTLLRYALKHNITDKVWIGSEAWVTSNVMVHTPGLGNVGTVIGFVIQSSTMPGFQQYISNLL
TAMDRQQSSVHTHSPTISLCFSCLNVSAESLIKRLDYLEKRQSFNVYTAVYSVAHALHQLLGCDSGGC
RDSPRPYPWQLLDEVRRVAFEVGNRSIQFDNNGNPKTSYEIILWKVSVEKLLLSVIGSYSGSLDISPSHI
DWHTETEEVPESYCSKDCVRGQMKKAKGLHSCCSECIDCPEDTFQNASDVSQCINCHYEEWSPARS
SACFPRSPQYLAWSSPMVVLLLLLVSLSLALTGLMTYLFVCHSGTPMVKASGGRRTFGTLTSLAMAS
CSSFLYLGEPTEHTCKIQQPVTSMCLSFCMSTLLGESLQILLTLELGSLTKGCLGVLKHRGNQLVVLL
SLVIQVSLCYWWLTTPGPFLIKLRESNKLLLFCKNHEETSFWLMLAHSGFQSLSCFMCTFLIQNPANT
YKLARDISLAMLLYFLAWVAFIPTYSAVSSIYAPVVEVFTVLGSTFGILGAYFVPKCWIIHFKPHCNTEA
YFQVYKQEPPTSMSNEKEKGVDSL
>Scyliorhinus_torazame_T1R3
MICCLLQLFFYLLFVKGSVTETQRLHEINVPYNLPGDYMIGALFPFHMIPVGLAHRLKPEEVTCDSFY
TFGYGLFLAMMFAIDEINNSTSLLPGVQLGYEVDDDCLETMVSLLPSLLFLSKDKNNGIDVLCNYTK
YRNRVIALMGPLTSEQAIVTAKLYGLFLIPQISFGASSEQLSNKKSYPSFLRSTPSDQNQAMAIVSIIRTF
NWNWIAGIGSDDEYGRQGIELVQQYASLQGICISYIEMIPTYLSLPSTKRKISEIMGQIRQMNVDVIVLF
SNEGPARELLKQVVKVNITGKVWIGSEAWVEAVTISHTPDIERIGTVLGTVVNNGSMPGFVDYVLNH
LTNIRDTREWSLSAEHGGNQLFPFWPYPTESSGQLLRDIQHLGPDNLSVVLDQPARRTAFGVYTAVY
SIAHALHAMLKCYNGRCQSTDKWYNWQLLEEMKQVNFTINNTTIYYDAAGNSQRGYDIITWIQHAG
QLKQPVIGNYKDHLTIHTSQIQWKTVDNTVPTSHCSKSCGLGQKKIAISLFSCCFECEDCPGGTFQN
FTDGFSCSDCQSDEWSPAKSTVCFKKILQYLSWVSPVGVAMLLFMVLELTLILAVMGIFLLNSSAPIVE
GTGGKMNMVTLTSLALLCCSTLLYLGEPTELTCKSSQPVTAFSLTICISTLLVNSIQILLSTEFTGLTKS
LLHRYKRSGWYITICASLGGQGAICYFWLNTDGDFLMKTPGSSEDALVLFCKSDSEVIFWLMLGYSG
LQALACFMCTFLIQTPAGSYNLAREISVSMLLYSATWICFIPIYPAVNKKHAPAIQISASLLSSFAILSAYF
IPKCSVIWFKPQHNTPEYFQIYDLQTSTRNVNDTKSLS
>Rhincodon_typus_T1R3_pseudogene
MNCCLLQLIFYLVMGKGSVTETQGLHGINAQYMLPGVYNIGDLFPFHTVPVGFAHRSKPEEVTCDS
FYPVGYGNFLAMTFAIDEINNSTSLLPGVQLGYEIYDDCFETLASILPSFLLLLKDKSNGIDVLCNYTEY

RNRVIALIGPWTSEQAIVTAKLFSLFLIPHISYGSSSEKLSNKKFFPSFLRTVPSDQNQAMAIVSIIRKFN
WNWIVGIGSDDEYGRQGIELVQKYALFHNICIGYIELIPFHLNLSSTKQKISDIVDRIXQMNVNVIILFSS
ERPAHEFLKQIVKANFTGKVWIGSEAWVKSAIIYAPDVGQIGTVIGTIINSGRMPGFESYVLNHFTNIR
DAKEQGIIAKHGDNQTFPFPYPTESSSQLLNAIQHPVPNNLSMVLDQPVTFSVYIAVYSIAHALHTLL
KYHNGRCQNTAGWQNWQLLEGLKQVNFTIGNTTIYYDAAGNLQQGYDIITWIQHAGQLKLSTIGS
YKDHLTIDASQIQWKTVNNKVPPSHCSKSCGLGQKKIAISLYSCCFECECLPGGTFQNVTDSFSCNEC
QPDEWSPSNSSSCFKKTLQYLSWISPVGVTLLLFVVLELMLILAIMVIFLVYSSTPVVEGTGGKKNMVT
LTSLAVLCCSTLLYLREPSEFICKSRLPVTAFSLTLSISTLLVNSVQILLSTELTGLTKSLLQRHKHSGWY
ITICISLIGQGTICYFWLNTDGDFLMQTTVNSKDALVLYCKSDSEVMFWLMLGNSGLQALACFMCTF
LIQTPPQTYNLAWEISVSMLFYIIIWLCFIPSYPAVSKKHASSIQIAATLLSSFAILSAYFVPKCFVIYFKPQ
YNTPDYFLIYGLQTQTRKECQ
>Gekko_Japonicus_T1R4
MEPCSLFKLLAFVLCTRPDKAKTNSLAVQFNAPGDFVIGGLFAFHSKVRYKKSRPEIPTCYSFSTHGFK
RYLGMRFAIDEINNSTALLPGVKLGYEIHNTCNNVVVATKPAVAFLSKYPGNAGLEAQCSYADYKPR
VIAVVGPDSSELSVVLSRLLNFLSIPQISPASTVENLSSKVLYPSFFRTVPSDRNQAEAMIQLLQKFHWN
WVAAVATDDEYGRRALETFVQLALSRDTCIAYEAILPSAPTKRERWEQLFKITSQLVVAGINTTVIFA
QSINAKELMMVVVERGITGKVWIASECWATSATIRFIPNLASVGTIIGLAVKSGRMPGFSEYVQRILPG
PEQDQTSSIIAGQNEQCPECGKLTLANLSAILYDTKFQATFNVYKAVYAIAHALHQLLQCNATSQKC
NMGREVYPWQLLAEIGKVNFTVESQPVYFNTKGNPPTGYDLILWRWNATGDFEFVPIGGYDALERE
LTVNESKIQWHTRDKKAPLSRCSQDCKSNQKRHIRGEHNCCYQCEDCPTGSFQDKTDPSQCTQCP
PNQWFLEENGTCEERAVEYLEVTDQLPLIMLALTMLFLAQMAVISGIVTKYRSTPAMRYIGIFPTFLIV
VSSTGLCGSCFFFIIKPSARVCMVRQPFFFISLTVVLATLLGKVVQSSGLNQALKSPLLRRHSLALCVLL
NTSVQSLLCVFWYLWDPPSLEENTNMEKTILLQCQDISFPGFGLLLAYNYCLALICCICSFWGSSSQQ
ARNQATKAIGFAMVLIIIIWTLFVPTYATSQGKYVSLFQVFAGLASVFAVFGSCYYQVCYVALFAPQM
NTDSRFCSLPQNPPAEDKSSVAEMK
>Madagascar_ground_gecko_T1R4
MELRLLLKFITLLLSTRPNKAKTNPLTVQFSAPGDFVIGGLFDFHSRVRYTTPLEIPTCHSFYVHGYQR
YLSMRFAIDQINNSTSLLPGVKLGYEIHNTCHNVVVATKPAVGFLSRYPGNDGLEAQCGYTNYKPRV
MAVVGPDSSELSVVLSRFLNFLSIPEISHSSTTDSLSNQGLYPSFFRTVPSDKRQAEAMVQLLQKFHW
NWVAALASDDEYGRRALETFVQLALSTDTCIAYEDILPSAQTQEERRAQLVKITSRLVEAGINTTVVF
AQPTRARELMTVVAERGITGKVWIASECWATSSTIRLVPNLASVGTIIGMGVISGKMPGFLEFVQHIL
AGPEQGQNNSGLAGQPEQCPECGNLTLANLSTIVYHTKFYTTFNVYKAVYAVAHALHQLLRCNATS
QKCSMDREVYPWQLLTEIAKVNFTVESQPVYFDQKGNPPTGYDLLLWSWNATGDFDLVPIGGYDA
LRRKLTVNESEIRWHTRDKKAPLSRCSQDCKPSQKRHLRGEHNCCYQCEDCPTGSFQDKNDPSECT
QCAKHQWFLKLNSTCQDRTTEYLEVTDLLPLITLALTVLALVQVAVIGGIFAKYRSTPAMRHIGTFPT
CLLLLSSTCLCSSCFFFLAKPSGPVCMARQPLFFISFTVSLATLLGKVVQSTGQPQTPTRAWVKRHAL

ALSVLVHSAAQSFLCVFWYLWDPPSLEENAGLQRTLLLQCRDVSFPGFGLLLAYSYCLALLCCLCSFW
GSSSQRVPSRATKSVSFAVVLIIIIWTLFVPTYATSQGKYVSLFQVFAGLASVFAIFGSCYYQVCYVVLFA
PRMNTVGHFCSLPGTPPAEEESDVAGTK
>Pogona_vitticeps_T1R4
MAPKLLLGLLAPILSLRMSKAKEDAPATRFNAPGDFIIGGMFAFHSKVQYLTVDGKPQFPACYSFYLI
GYLQHLAMRFAIEEINNSTSLLPGIQLGYEVHDTCNNEAVATKAALAFLSRDPENDNAAQCDYANAK
PRVIAVVGPSSSSLSMLVTRILNFLSIPEVSFGSSSSYLSDRQTFPSFFRTIPSDSCQADAIVQLLNKFNW
NWVAALATDNLYGRQALEIFTQEALLKDICVAYEAILPDGLNPSEQNAKLIDIADRLENSHINATVVF
ASPEEAEALLRAVVATRITRRVWIASECWSASPGVALIPGISNIGTLFGIAIKSGAMPGFAEYVQNLLAQ
PQPGRTTEEEEVNEQCPECGSLTYANFSRAIQNSRFYTMFNAYKAVYAIGHALHQLLHCDTASRTCD
ADREVYPWQLLEEIARVNFTVESEPVYFTETGDPPTGYDLILWDWIPPKNHTFGIVGSYDALERQLT
VNESKIKWNTEDGKLPVSRCTQECKPGQKRWLRGEHNCCYECEDCPAGSFQDQAHPTECTPCPQH
QWSPAQSTKCFNRAVEYLEVTDLLATIMVALTGLALAQMAAVTGVLLRHHASPAIRYVGRFPASAVI
LSSACCCTSCFLFVTKPTDTICKLRQPIFFGSFAVCLAALLGTAVRRSGLEQTLKRRWLRKHHMGFSIL
LNTAVQGLLCLLWYYWNPPFLEENTELDKILLLQCEDSAFPGFTALLAHVYCLALFCCFCSLVGQGG
NQMDRRATKAINFAVVLILIIWTLFLPAYVTSQGKFVALFQIFAGLASILAIFGSCYYPVCYVVLFAPHL
NTDDYFSCLPQDPPADKEKPSAAEAKG
>Anolis_carolinensis_T1R4
MTSKAFVGLLVWILFIRVDDGKADSLAVQFTAPGDFILGGLFALHSQAEFQFRDGRPETHTCYSFYSP
GYQQQLAMRFAIDEINNSTALLPGITLGYEIHDTCNDELVTTKLAISFLSEDPSSSLAMQCNYANYKP
RVVAVVGPNVSGLSMLVAQILDFLSIPEISAGSSSIKLSDKQLYPSFFRTIPSDQNQADAMVQLLNYFN
WNWVAALVTDNEYGHQVLEVFVQLALSKDMCVAYEAIFPRGQDEIQRKQELVKIANQLEVSRINAT
VIFSHSGEAEELLQVVAGRGITGKVWIASECWSTSSAIALIPNLNKIGTVLGMAVESGEMPGFQEYIQK
VLANTELDQKSPNVLEACPECSYLSLANFSKFFEVFNTYKAIYAIAHALHQLLNCSTTHKTCDADRDI
YPWQLLEELPKVNFTIESQPVHFSKTGDPPAGYDLLFWDWILPNNHTFAVVGRYDASTHKLNINESK
IRWSTENGKAPVSRCTQGCKPAQKRWLRGEHNCCYQCEDCPAGYFQDTNKPDECTPCSKNEWSTA
RSTTCQPRAVEYLEITDLIAIIMMALTVLVFAQMVAVTGIFATYRGTPAMRYVRGFPLQSILFSTTCFC
VSFPFFVMKPTKEICKVRQPLFFGSFTLILATLLGKTLQSVGLDQILRRPWVRNHLMVLCIVVNAALQ
GLLCLFWYYWEPPVLEENTDIERTILLQCHDSKFPGFGILLAHDYCLAVICFACSLVGYNTEKKKDNV
AAKSINFAMLLILIIWIIFVPTYSTTKGKFVALFQVFAGLASIFAIFGSYYYPVCYVMLFAPHLNTDNHFC
LSQDLPVDKEPSARDSK
>Ambystoma_mexicanum_T1R4
MWLWMVPGLLLLFMGLVQPVQCAINRLASQFSSPGDYVIGGLFAVHSSVQKLQDGTHIVPICTSFHP
PSYRRLLAMRFAIEEINKSSRLLPNITLGYDIHDTCSDTLVALRPALGFASTLHCGGHAVEVRCNYTDY
QPRVLAVIGPGSSEICVILARLFGFFGLPQVSYSASCEILSDRARFPSFFRTIPSDKGQVRAIAQLVRYFR
WNWVAVLGTDDEYGRKGIESLTELVSVTETCLAYQDLIPVHVSEEEAAQRLSSIVDGLVQSRVNVTIL

FASETHLAPLMGALIARSVTEKVWIASADWSTAASIAGISNISSLGTVVGFAFKSNHMEGFVEYVEQVL
SDQGSGPSGVSRSSDANKCPECGSLTLANVSTILDRSLHRRTFTTYTAVYTVAHALHKLLRCSGESLQ
CDKGAPVYPWQLLEELVRVNFTVGPEAVYFSKDGDLPAGYDLITWDWSGKGTTPDFRVVGQYDAL
QSLLQVNASRIQWFTGSKEVPPSNCSQECEPGQMKRVKEHNSCCYECESCPAGFFQNISDDPLKCTR
CPSHQWSNEKSTDCQERTLEYPNWADAISILMGITVILGLLLTAAVTTVFAHNYHTPAVQDAGGLSS
LLILFSLACVCSTFCLFIGKPSLLRCQIRQPLFSSSFTVCLSVLLGKSVQVSGLCHPVIPVCGLLSVYICVL
LNVSIQAVLCFSWYFWNPPFVVQNPNISASSLVVECREDALTGFGLLLGHNGLLALTCFMLTFMGRS
SGKSYPSARNITFSALIYLLAWIFFIPTYVTSTGKNLPFFQLGSGLISVFGMLGAYFIPRCYIMVCKPELN
TLFHFQNLTQETLSIPEVVKSEGHGWTGTQKKPLK
>Rhinatrema_bivittatum_T1R4
MLGVELLGFFCLLFPVGGRSQVVTPINDQAVRFSSPGDFVIGGMFPVHSSVHYPPLDIPTCTSFYAPG
YRKLLAMRFAIDEINNSSSLLPKVTLGYDIYDTCFETRVAMRPAMSFLSRHPGGDGLQVQCNYTDYR
PRILAVVGPESSEECIILARLFNLITLPQVSYSASSDILRDRTRFPSFFQMVPSDRSLVQAMAQLVSRFQ
WNWVAVVGSDDEDGHRAVERFTELASIMEICVAYQDLIPSYASEPESAKRVAEIADGLVQSHVNVTL
VFANEINVMKLMKALLQRNIINKVWLASEKWATSPSVAAIPDISSLGTVIGFAIKSGPMPGFREYVQRIL
ANQGLGSEPPAVCVPGISRGKQCPECEMLTLANISSILDSTVYRTTFNTYTAVYALAHALHQLLQCD
AGELNCTKDAQIYPWQLLEQMAKLNFTVNSHPIYMSNSGEPPVGYEIITWGWRSSNITPEFLPVGNY
NPMEGKIDVDRSKIQWCTEHRQVPQSNCSQQCEPGQMKHVKDDQSCCYECESCAAGSFQNLSEDP
YKCTPCPSRQWSEEKSTACRDRALEYMDWVEAPALLAAALTVLGLLLVAAVSLTLLRHQHTPAVQA
AGGRSCLLMLLSLASVCCSIGLFLGKPSWLHCQVRQPLFSISFTVCLSVLLGKSLHLSGLCFPDAVSFL
CVLLNLLIQGGLCSFWYHQSPPFVSENPNISASAFVIECSEGSFTGFGLLISYNGFLAVLCFMCTFMGR
SVENSYKRARCITFSVLIYFLAWIFFIPTYTTSKGKTVPLFQVFSGLISIFGVLGAFFTPLCYIILFKPELNT
LSYFPSTAEEPPGEQQDSRSTQLQN
>Neoceratodus_forsteri_T1R4
MMCGFFVLSLWILVIHASGSAASAVFSGPQKMAVQFSSPGKYVIGGLFPIHSTVLNITSRTKPESLVCD
RLYLLGYQYALAMKFAIDEINNSTQLLPDVDLGYEIYDTCNEPLVAIQPTMRFMSATPAQSIEVQCNY
TDYTPQVLAVIGPDSSDLSAVLNRLFSFFVIPQISFSASSEILSDKDRFPSFFRTIPSDHNQVEAMAQLV
EKFQWNWIATLATDNEYGRRGIESLSELISEKNVCIAYEDLIPLYASATDLSQKVKGMIANLVNVNVN
VTIVFGSSIYVEAFMKVVAQEKLKGMVWIASESWATTETIASIQNISSIGTVLGVAVKSGKMPGFEEYV
ANILSNTGNTGSYSRSPQSNSMSGVCRPLNDNEEDTCMEQCHECVILTHENLTDILGKRIWRSTFNV
YTAVYAAANALHELLKCNSGKCDITTHIYPWQLLEELGRVNFTISNAHIYFTSTGDPPTGYDIVQWK
WNGTGVPTFQEIGEFRPVQRSLDINTDMIRWHTMNNTIPGSNCSKTCEPGQIKKVTGYHSCCHECE
DCPAGSFQESEDKCTPCLKYQWSAAKSQACSNRTVQFLEWTDTPSILLATLTAVGLLLILAIAIVFAR
NLSTPVVQAAGGYISFLMLFSLACACCSFYFFIGKPNQFSCSIRQPLFSISFACSLSVLLGKSFQVANICSS
QKMCIQCHLEFILILLNIAVQISICTICRQWNPPKVVANYDLIDAIVLECKEGGSLEFMLMLGYNGILA
VICFICTFLGRSSDSTYNMIRCLTFTMLIYFLAWIFFIPTRATATGKFVPCLQVFAGLITAYGILGGYFAP

KVYIIVFKPEYNTVTYFQSFIQKASPSTEPKSNVVASQVNNAEPQSNNVSSQSNNIEPQSN

>Protopterus_annectens_T1R4a

MHGFCLANFWILIILTHGERPLVKNQEKTVQFSSPGKYVLGGVFPFHSTVLHIDSRTEPEPFICDGFNS

IGYQYALGMKFAIDTINNSPELLPNVTLGYEIYDTCCDPLVSIQRTMNLIAASPNETIEVCCNYTYYNP

KALVVIGPETSTIAVMMNRLLSFFLIPQISYAASSETLSDKATFPSFVRTIPSDAKQVLAIVHLIAKFQWN

WVAIVASDDDYGQRGTQGLIEQLSIKRICVAYSDFLPQALSENQFTQYRIIIDNILAKNVNVVVIFANA

PEDFIHTVVQSNVTNKVWIASETWSTSFTVASMANISSIGTVLGTSVKSGQISGFEDYITNVISGTEKS

HCVLKSSQSMNMTDVCSLQERDNDICMDRCTECMTLTSANVTDIIGDGVWRVTFNVYVSVYAAAH

ALHRLLKCSSNECNTNIKVYPWQLLEEVKEVNFTVSDTQIYFNSFGDSTVGYDIMYWQWNAAKPFLK

IGEFQAGQERMIIDASIIHWFTANNTIPVSNCSKNCEEGQIKVVRGQIICCYDCENCPAGTFQENEYKC

TPCLKHQWSLVRSQECYERTIEFIKWTDALSIVIMTLTAVVFTAILSAVIIFIKNFSTAAVQASGGYMSF

VMLFSLVCECCSFFLFINRPTQIFCFISQLTFSVSFTISMSVLLGKSFQVANIYLSRKSCVQHYQGYILIL

MNIVIQTGICYIWKYFSDDLVGESYEDPTVVLLRCTAGNTPLFVVMLGYNVLLATICASCVFLRPNVG

NAYNMARSLAFILLIYFLALIFFIPVRITTFGITVSYVQVFSGLMTVYGIFWGYFAPKIYIIIFKPDCNTVT

YFQNFIKNSSPDIDTKNNDAVSHVINVETEISTVSSQNN

>Protopterus_annectens_T1R4b

MYCVGMTILWILIILTRVNGSSLKNPPVITNFSAGNYVLAGLFPLHSNVLYIENRTKPEPFVCGSFNPIG

YQYALAMKFAVEEINNSSRLLPNVTLGYDIHDTCLDPLVSVQKTMNFMSAGPNETIQVLCNYTNYSP

RVLAVIGPKNSDVAVVINRILSLFLTPQISYSASTATLSDINEFPSFFRTVPSDKNQVNAIVQLIVKFQW

KRIAVLTSDDEYGKQGLKEFQEKLSKKDICIAYNDVIPQTQAQSGNQFTKIINNIIYDLKSANVGVVVL

FASNATDFMQIIAQKNVINMVWIASEAWSTSTTVARILGNSNIGTVLGTALNSAYISGFEDYVRKVVL

DIEKSQYFCTSSQSSNITDICSVQEHSEQDRNTCTVQCTECMTLTHKDIAAILGDGVWRETFNVYTA

VYAAAYALHGLLNCDAGECDTAVKVYPWKLIEKLKQVNFNVSDTRIYFDPFGDSPAGYDIIYWQWN

RSDVPSFLKIGEYKQEQEVLDINTSMIQWHTVNNTIPESSCLSKCKDGQIKIAKRQVPCCFYCEDCPA

GTYQQNEYTCATCSWQEWSLVNSKACYNKTVDFLQWTDVRSIILMIFTGALLVVILSAVLIFARNYST

AAVQASGGYLSFLMLFSLVCSCCSICLFITRPTQFSCLLRQVVFSVSFTISMSVPLAKSFQLANIYMFQR

ACVQHYQGYILMVINIIIHTGICTIWWYLSPDLVNEVYESPDAISLMCARGSIFGFVMMLCYSGILATIC

AACVFLVANSENTYNMANSIRFVILIYFLAWIFFIPIYATANGISVQYAEAYSGLMAAYGIFWGYFAPKIY

IILFKSEYNSFIYFQHFLDSNSSGMYPGNNDSISQVNQVEIESNTVSSQRNI

>Protopterus_annectens_T1R4c

MHCICLISLCILIILTAVHGPPVKNPAMVTQFSLAGNYVLGGLFSLHSRVLYIDNRTKPEPFICDSFDPA

GYQSALAMKFAVEMINNSSSLLSNVTLGYEIYDSCNDPLVAVQRAMNLLSVNPNETIEVQSNYTYYSP

QVLAVIGPKSSDNAVVTGRLLSLFLIPQISYSASTATLSDINEFPSFFRTVPSDKNQVNAIVQLIVKFQW

NWIAVLTSDDEYGQQGLKEFKEQLFGKHICMAYSGVIPQDNSQDQFSQLINNIVTNLLSTNVSVIVIF

ATNPADFMQSIVEKNVTDMVWIASDAWSTSATTARNIKYIGTVLGMALKSDNIPGFEDYVTKVVLDI

EKSQNFCTSSQSSNITDICSVQEHSEQDRNTCTVQCTECMTLTHKDIAAILGDGVWRETFNVYTAVY

AAAYALHGLLNCDAGECDTAVKVYPWQITDKLKHVNFTVGDTQFYFDSVGDSPAGYDIIYWQWNA
TDVPSFLKIGEYEQNKNVLNINTSMIQWHTANNTIPESNCSKKCGDGQIKKVNAQIPCCFYCVDCSP
GTYQQNEDTCALCSQQEWSPAKSKACRERTIQFLQWSDAGSILLMIFTGALLVVILPAVIIFARNYSTA
AVQASGGYLSLVMLFSLVCKCCSFCLFISKPTLFICFLRQIIFCVSFTISMSTLLAKSFQVANIYVSQMAC
IQHYQDCIVIIMNIIIQSAICTLWWYVSPAIFIENYESPDAIELLCTSDNTTGFVVMLGYNGVLATICTV
CLFLGENSGSTYNMARSISFVILIYFLAWIFFIPIHATSAGVYFSYAQAYSGLMSAYGIFWGYFAPKIYIIIF
KPECNTAMYFQNCIESTSLRIDPSKNDTVPQVSNVERDTVSSQRNE
>Latimeria_chalumnae_T1R4
MLKAIVLSSWLVILYATSIDLLVPDHQRQVVQFRSPGDLMIGGLFSMQSAALNVTNRTKPQTPPCER
FYPMGYRAHLAMRFAIDEINNSTKLLPNVKLGYEIYDICLESLVAMRSTMMFLSKYQSDVIEVQCNYT
DYSTRVTAIIGPGSSELAIVISRLFSFFLIPQISYSASSELLSDKKKFPSFFRTIPSDKKQAEAMALLVKEFQ
WNWVAAIGTDDEYGRRGMEKFVELASEKGVCITYEDLIPIHETTDGFSEKIEQIVNNLVETEVNVTVI
FADDRYVDAFMNVVIQQQVTDKVWIGSEAWITDANVARIQNISSVGTVIGVGMKSGEMPGFEAYVS
KILSDPAIKESISFSLYSNNTSGFCMSGDEDEVCRSQCKECETLTAENFTTIIDSPSMRVAFNVYSAVYA
VAHALHKILKCDNGDCNREIKFYPWQLLQQLAKVNFTINSRQVYFTSSGDPPTGYDIMNWQWDTG
AMPKFQVVGEFKTLDSSLHVDKTKIHWHTKDQQIPVSRCSKQCEPGQIKNVKGYHTCCYECDDCPA
GFFQNVTGDPNKCTQCKKHQWSLPRSQACQEREEQFLEWTDAVIIPMVILSIIGLLLIIAIVILFAKNH
NTPVVKAAGGNICFLMLFFLFCSCCSFYCFVGRPNWVFCTIRQPVFSIGFTGCLSVLLSKSFQVAALYS
CQSTCLQNYGRYFFFLASILIQVVLCFVWQYWTPPQVIANYNVTEKIIVIECEEGSLAGFGLMLSFNGA
LAVICFMCTFMGRSSAKTYNMARSLTFSMLVYFIAWIFFIPTYATSEGKFVPCLQFLSGLISMYGIIGAY
FIPKCYILLFKVQCNTLAFFHTFIEGSSPKTNEQKNDLSK
>Polypterus_senegalus_T1R4
MNWHVYQTVLALLWHMGNPQETLFDNNEQFRLPGDYVIGGLFSLHSRAVNLDSRTKPEFITCDSFY
PSGYRYFLTMIFAIDEINQATDLLPGVQLGYEVYDSCMEMLGTVPPSLRFVSKGDSNGVAVQCDYM
NYQPRVIAVVGPTTSEETIPIARLLGHFHIPQIGYSASSEALTNRQRFPTFFRMIPSDNVQTEAILQLLH
QLGWNWVSLLCTDDEYGRGGCTKFINLAEQHSICISHWQLLPQRVEAKSNAQLGQILSTLKSVQSNS
TVLFANDLYATALLQEAVSQGYGSGKVWIGSEGWTASRRVADIPNVGKAGGVLGLALQKGRMPGFE
PFLEKWLKRMSSRPRQECQAGGDLSQGVCFPNCAECYSFTLQNYSYLLGGTQRRTSFATYASVYVIA
HALHSLLQCNQSGCLKSKVCTQKLVEELWKVNFTLETSPISFNSKGDPPSIYEIVNWQWHQGGTPSI
VTVGKYINGKLSLDLPNVYWNTPDNRPPGSNCSSQCEDGQVKRGSGCCSKCDDCPAGTFSNISDDS
NICSPCPLEQWSYPRSSNCRDRIVEMRSWTNFSTIFLLFFSILAMVVLLSVALVFVCQHQSHMVHEAG
GASCFLMLLSIFSSCTSLPFFLGPPSPSTCLARQPLFSAGFTVCIAAMLAWPLRPPHSSTLCCQVSLQSC
TGRILVFTISLLPQVVNFFLWLWLNPPAVIPNYDILDNLVLLECFEGSNIFFGFSICYNCILAALALALTV
GRLDLWSYNTGRQATFSLSVFIISWIFFLPTYATSKGLNVPCIQVFSGLVCIYSIQGSYFLPKCRLLLFKP
NIGTNGFLPRASLDQPGSIRTVDSQ
>Callorhinchus_milii_T1R4

MSPLRYWMLASLVLVGHNGAEPQGSDRRVGEYLAPGDYIIGGLFPIYSSKLDLQSVNKSEHISCESFYP
QGYHWLQAMRFAIEEINNGTELLPGMRLGYEIYNTCFESTVAIESAIALLSNSEQDSIEVTCNYTEYNT
RVVAAIGPSQSELSIVVARLFSFLLLPQVSYASSSNVLSNRRNFPSFFRTIPTDEVQADAMASIVYMFQW
NWIAIIGTDNDYGRQGIELFSVQASKRKICVAYEDLIPLHLTGVQFKAKLVSIVNNINYSKINVTIVFCD
DRFALALIDTVLQLNITGKVWIASEGWVTSTSFQTLTNVASIGTILGVAVKTGNMPGFDQYVLAEDP
GSCINPTPTEICHDSKKLHTELAAQDCLSHFVQTHNGVVDTQPQRITFNVYSAVYAVAHALHALLEC
DSGSCKKKSIYPWQLLETLAQVNFTLNGHDIYFTPNGDPPTGYDIVNWHWNGKDQEPEFRKIGEYR
ALWKELDINVSQIQWNTPGNQIPVSNCSTSCKMGQVRMLKGYQSCCYECVDCPKNTFQNQMNKCI
ACRSDQWSKERSVECHNKSIAFLEWTDSPTIVLSTMAVLALLVIISAVAIFIVHFHTPIVQFAGGSLCFV
MLVSLAISCCSIFCFVGCPTQLSCTIRQPIFNIGFTGCLSVMLTRAFQVGSLCGTSSIPPTLCPKLIIASM
KYLVVAFIIMCQAALCTWQFSLPPTVLNNYNVSETQIVVECSESSLSGFGLLVAYNALLGLACFLCTF
MGQRSGQAYNLARCIMRSTLIYFAAWIFFIPTYTSFSGKLVPCIQMSTGMISIYGILVAYFIPKCYIILFKP
EYNTQSNFQSSPDNLSSKEREQ
>Scyliorhinus_torazame_T1R4
MFHLHFWMVFCFVPNGWCNSSVSGPHRDPVSWFQAAGDYILGGLFPLYSTDVNWNNETKSESREC
ERFQLDGFHWLQAMKFAIEEINNSSTLLPGVTLGYTIQNTCLKSSVAMQSAISFLAPKNGNRFELKCD
YTDYSTRVLAILGPSNSELSRVTARLFSFLLIPQISYAASSTLFSDRMSFPSFYRMIPTDEVQAAAMVSIV
ETFQWNWMAVIGTDNMYGRRGIEHFTNLASKSGICIAYEELIPLNLPGSELQRKMVSVINNIVYSRVN
VTAVFADEEYAKTLMTIILEQNVTGKVWIASEAWITSKIVARSPNISSIGTILGVAIKSSYIPGFKYYTALA
HSHLKSQKCRSDHKSLEEEECKEQSVSEGAAIVEDMQRISFNIYSAVYAVAHALHHLLRCDIGICTRRT
VYPWQNQCLPCLKNEWSLSKSVECQKKTIEFLQWTDTLAILLASLTAVGLFIIAAITGIFIINLNTPMV
QLAGGTTCLVMLISMAISCCSLYCFMEKPNWLLCTIRQPIFSISLTGCLSPMLVKSFQVSGLFRISGSTP
QWWPGIMRHIGRYLMVCSLFLLQIVVCAVWLSTSPPSVFANYNISVTAIVMECDEGLVTGFGLLLVY
NGLLALACFLCTFMVQSSAKTYNLARHITFAMLIYLMAWVFFIPAYTTAKGKFVSSIQLFASLVSVYGII
TAYFLPKCYIILLKPEFNSQSYCQSPMNNPPPTTE
>Chiloscyllium_punctatum_T1R4
MLHVHYWIVFCCILNGQYDTSASDRSRDPVRRFQAAGDYILGGFFPLYSTEVHWMSETRSESGKCES
FQLNGFHWFQAMKFAIEEINNSSTLLPGVTLGYDIQDTCLKSSIAIQSAISFLTVKDENRFELKCDYTN
YSTRVLASLGPSNSELSKVIARLFSFLLIPQISYAASSNLFNDRTNFPSFYRTVPTDEVQAAAMVSMVKT
FQWNWMAVIGTDNMYGRRGIEHFTKLASGTGICIAYEELIPLNLPGSKFHRKMVSVIKNIIYSRVNITA
VFADEHYAQTLMTIILEQNVTGKVWIASEAWVTSETVASSPNISSIGTILGVAIKSGHIPGFECYKSAAL
ALRDLPSLKSKRCGSEGKLLKEECNECLTQSVDEDTDGDSEELQRISFNVYAAVYTVAHALHCLLRC
NLGNCSRSTIYPWQLLKEIPQVNFTLHGRVIFFDKYGNPPTGYDIINWQWRSGNQIPEFKMIGEYIAQ
QKELRINTSLIQWNTPQNKIPGSNCSTSCEPGQIKMVKGFHSCCYECADCPAGTFQSDDNQCIPCLK
NEWSLLKSIKCQNKTIEFLRWSDTLGVLLVSLTTAGLLIIVAITSIFFINLNTPMVQLAGGKTCLVMLV
SMSISCCSLYFFLEKPNLLLCTIRQPIFSFGLTGCLSAMLVKSFHVSGLLRGTSFSAQWWPGVLKYLGV

CSLVLIQIILCSVWQSTSPPSVFANYNISVTVIVVECDEGSVFGFGLLLGYNGLLALACFLCTFMVQSSA
KTYNLARHITFAMLIYLMAWVFFIPAHAAVKGKFVSSIQLFTGLVSIYGIITAYFLPKCYIILLKPEFNSET
YCRSQMVNPPPNTESQ
>Rhincodon_typus_T1R4
MLHVHYWIVFCCILNGQHDTSASDRHRDPLRQFQAAGDYILGGLFPLYSTGVHWMTETKSEFGKCE
SFHLNGFYWLQAMKFAIEEINNSSTLLPGVTLGYDIQDTCLKSSIVIQSAISFLMAKDGNRFELKCDYT
DYSTRVLAALGPSNSELSKVIARLFSFLLIPQISYAASSNLFNDRTDFPSFYRTVPTDEVQAAAMVSMV
KTFQWNWVAVIGTDDMYGRRGIEHFAKLASRTGICIAYEELIPLNLPGSKFQRKMVSVIKNIIYSRVNI
TAVFADEQYVQILMTIIHEQNVTGKVWIASEAWVTSEAVASSPNISSIGTILGVAIKSGHMPGFECYKS
AALALRDLPSVKSQRCGSGGRLLEEECNECQTQSVDEDADSDGKDLERISFNVYAAVYTVAHALHRL
LRCDLGNCSKSTIYPWQLLKEMPQVNFTLHSRAIFFDKYGNPPTGYDIINWQWRSGNQIPEFKMIGE
YRAQHEELRINTSLIQWNTPQNKIPGSNCSTSCEPGQIKMVKGFHSCCYECADCPAGTFQSDDNQC
TPCLKNEWSLLKSIECQNKTIEFLRWTDTLAILLVSLTTAGLLIIAAIIGIFIINLNSPMVQLAGGKTCLV
MLVSMAISCCSLYCFMEKPNWLLCTIRQPIFSFGLTGCLSAMLVKSFHVSGLFRGTGFRAQWWPGVL
RYLGVCSLVFIQIILCSVWQSTSPPSVFANYNISVRVIVMECDEGFMTGFGLLLGYNGLLAVACFLCTF
MVQSSAKTYNLARHITFAMLNYLMAWVFFIPAYATAKGKFVSSIQLFAGLVSIYGIITAYFLPKCYIILLK
PEFNNKTYCQSPNDNPPPTTESQCHTVPAHCWR
>Homo_sapiens_GPRC6A
MAFLIILITCFVIILATSQPCQTPDDFVAATSPGHIIIGGLFAIHEKMLSSEDSPRRPQIQECVGFEISVFL
QTLAMIHSIEMINNSTLLSGVKLGYEIYDTCTEVTVAMAATLRFLSKFNCSRETVEFKCDYSSYMPRV
KAVIGSGYSEITMAVSRMLNLQLMPQVGYESTAEILSDKIRFPSFLRTVPSDFHQIKAMAHLIQKSGW
NWIGIITTDDDYGRLALNTFIIQAEANNVCIAFKEVLPAFLSDNTIEVRINRTLKKIILEAQVNVIVVFLR
QFHVFDLFNKAIEMNINKMWIASDNWSTATKITTIPNVKKIGKVVGFAFRRGNISSFHSFLQNLHLLP
SDSHKLLHEYAMHLSACAYVKDTDLSQCIFNHSQRTLAYKANKAIERNFVMRNDFLWDYAEPGLIHS
IQLAVFALGYAIRDLCQARDCQNPNAFQPWELLGVLKNVTFTDGWNSFHFDAHGDLNTGYDVVL
WKEINGHMTVTKMAEYDLQNDVFIIPDQETKNEFRNLKQIQSKCSKECSPGQMKKTTRSQHICCYE
CQNCPENHYTNQTDMPHCLLCNNKTHWAPVRSTMCFEKEVEYLNWNDSLAILLLILSLLGIIFVLVV
GIIFTRNLNTPVVKSSGGLRVCYVILLCHFLNFASTSFFIGEPQDFTCKTRQTMFGVSFTLCISCILTKS
LKILLAFSFDPKLQKFLKCLYRPILIIFTCTGIQVVICTLWLIFAAPTVEVNVSLPRVIILECEEGSILAFGT
MLGYIAILAFICFIFAFKGKYENYNEAKFITFGMLIYFIAWITFIPIYATTFGKYVPAVEIIVILISNYGILYC
TFIPKCYVIICKQEINTKSAFLKMIYSYSSHSVSSIALSPASLDSMSGNVTMTNPSSSGKSATWQKSKDL
QAQAFAHICRENATSVSKTLPRKRMSSI
>Mus_musculus_GPRC6A
MALLITVVTCFMIILDTSQSCHTPDDFVAITSPGHIMIGGLFAIHEKMLSSDDHPRRPQIQKCAGFEIS
VFLQTLAMIHSIEMINNSTLLSGVKLGYEIYDTCTEVTAAMAATLRFLSKFNCSRETVVFQCDYSSYM
PRVKAVIGAGYSETSIAVSRMLNLQLMPQVSYESTAEILSDKIRFPSFLRTVPSDFYQTKAMAHLIRQS

GWNWIGAITTDDDYGRLALNTFAIQAAENNVCIAFKEVLPAFLSDNTIEVRINQTLEKIIAEAQVNVIV
VFLRKFHVFNLFTKAIERKISKIWIASDNWSTATKIITIPNVKKLGKVVGFAFRRGNTSSFHSFLQTLHM
YPNDNNKPLHEFAMLVSACKYIKDGDLSQCISNYSQATLTYDTTKTIENHLFKRNDFLWHYTEPGLI
YSIQLAVFALGHAIRDLCQARDCKKPNAFQPWELLAVLKNVTFTDGRNSFHFDAHGDLNTGYDVVL
WKETNGLMTVTKMAEYDLQRDVFITTNQETKHEFRKLKQILSKCSKECSPGQMKKATGSQHSCCYE
CVSCPENHYSNETDMDHCLLCNNETHWAPVRSTTCFEKEVEYLDWDDSLALLLIALSLLGIAFVLAI
GIIFTRNLKTPVVKSSGGLVVCYVMLICHALNFASTGFFIGEPQDFACKTRQTLFGVSFTLCVSCILTK
SLKILLAFSFDPKLTMFLKCLYRPVPIVLTCTGIQVVICTLWLVLAAPSVEENISLPRVIILECEEGSALAF
GTMLGYITVLAFICFVFAFKGRKLPENYNEAKFLTFGMLIYFIAWITFIPVYTTTFGKYLPAVEIIVILISN
YGILCCIFFPKCYIILCKQKTNTKSAFLQMVYNYSAHSVDSLALSHVSLDSTSYDTATTNQSPGNKMT
ACQNDNHLPAQVLPHTGTAKTIKASKTLRQKRSSSI
>Gallus_gallus_GPRC6A
MALFSLALIPFVISSDAASACQNTDDFVGASSPGDIIIGGLFAVHSEMLQPEEHPIKPVIQNCAGFEIQIF
LQTLAMIHAIEMINNSTLLSGVTLGYEIYDTCAEVTKAMASALRFLSKSNTSKDIVEFKCNYSDYVPRI
KAVTGASYSEVSMAVSRLLALQLIPQVSPASSAEILSDKIRFPSFLRTIPSDFHQTRAMAHLICESGWNW
IGVIATDDDNGRFALESFGVQAMANSVCIAFKEMLPAYLSDNTFHTKVDRAVEKIVKETRVNVIVVF
MRQFHVLKLFKKAIERNVKKIWIASDNWSTAVKISTMPNIRKLGTVVGFGFKNKDLSTFQDFLRNLH
DRPTENNKFLLEYIMLLSVCAHLDNYDFQMCISSQSQYDLMQNVENKHQIWRDDFLNANIEPGFIHS
TILAVYAIAHAIKGQCKDRNCKNPSAFAPWELLEELKKVTIIDDDKEIKFDSKGDLSSGYDVLLWKEV
DGRMEITTMAEYDPENGYFIFEDEEKKKEFLDLKKVPSTCSQHCRPGQMKKVTESPHTCCYECVYCP
ENHYSNQTDMDYCYRCHNKTYWAPVNSTTCYRKTIHFLGWTDWFAIFLLLLSAFGVVLIFSISAIFTK
NLSTPVVKASGGLTVCYIILLSHFFIFLSTVFFIGEPTEFKCRTRQALFGISFALCISCILIKSLKILLAFSFD
PKLQNFLKCAYKPITIVFICTGIQVIICTFWLIFRTPFVKQNFSIPRAIILECNEGSVVAFGIMLGYIAALA
FICFICAFKGRKLPENYNEAKFITFGMLIYFIAWIVFIPVYVTTFGKYLPAVEIIVILISNYGILCCTFFPKC
YIIIYKQETNTKSAFLKMIYTYSSKSVGSIAVSQISLDSKSSSSRITESDSCNAEKSSVNGNCHFQVSGQTP
VKEKAVPKRATRTLSRKRLSSI
>Anolis_carolinensis_GPRC6A
MAIFGLWMLVFLVCLDISQSCQSPDDFVAASSPGDIVIGGLFAVHGKMLHSEEKPLQPVIAHCAGFEV
QVFLQSLAMIHAIEIINNSTLLPGIKLGYEIYDTCAEVTRAMAAALRFLSKFNASKDLVEFQCNYSDYT
PRVKAVIGATYSEVSMSVARILNLQLIPQVSHAATAEILSDKVRFPSFLRTVPSDSYQTKAMARLIHRSG
WNWIGIIATDDDFGRLAVESFGLQAMANNVCIAFKEMLPAYLSDSTIHGKINQALEKIVKETRVNVIV
VFLRQFHVMRLFRKAIEMNINKTWIASDNWSAAVKISTLPNISRLGKVVGFTFKSGNMSSFHQFLSNL
HKTPTGNYTIAREYAMLMSTCSHINNQDLSKCISNYSGEKLLKNRTQPSSQLWDEEFLIANIEPGFIHS
TMLAVHAIANAIRNQCKNRNCKDPYAFAPWELLEELKEVKFTDDDREVYFDSHGDINTGYDVLLWK
EIEGRIVITNMAEYDLEKDDFIFENKEDEIEFLNLKNVQSKCSRKCRPGQVKKVSASPHTCCYECVSCP
ENYYTNRSDMDYCILCNNRTHWAPVNSSTCYRKMVQYLNWNDWFAILLMVLSVLGIVLICAIIIIFTR

NVDTPVVKASGGLTVCYVILLCHFLTFVSTGFFVGKPRAYKCKTRQALFGISFALCISCILIKSLKILLAF
SFDPKLQKLLKSLYKPVPIVSFCTGIQVLICVVWITVRSPFVEENFSIRKIIIVECNEGSVVALGIMLGYIA
LLAFICFICAFKGRKLPENYNEAKFITFGMLIYFIAWIIFIPVYTTTFGKYLPAVEIIVILISNYGILCCTFLP
KCYIILYKQEANTKSAFLKMLYSYSSKSAGSLSVNQSSLDSKSETPPMPKAETCCKAKTEKNWVNGSC
HFQASGYREIRWEMQAVNIVGPTIPRRRLSSI
>Nanorana_parkeri_GPRC6A
MALYGMSVITGILISGIYCCVIPDDFVGARAHGDIIIGGLFAVHGRMMNSVRGYPHQPAIQNCAGFEM
QGFLQILAMAHTIELINNSSLIPGVKLGYEIYDTCSESTQAISATLRFLSTYNTSGDTLVFKCNYSDYTP
RVKAVIGDSYSEVSIAVATLLNTQLIPQVSHASSAEILSDKFRFPAFLRTIPNDSYQTRAMAKLINFSGW
NWIGLITMDDDYGRSAIESFGAQAININVCIAFKEVIPSHLSDSTVQSRIDKTIQTILKETSVNVIVAFLK
PSLIIKLFIKVMEKKIKKTWIASDSWSVSTGVSSIPDIEKIGQVIGFMFKSGDTTSFQEYLKNLNQQKFE
MNRFVDKYSVLVSDCSKGKYSDIYSCVTDSSKEAVVTSRRIKNKALGVDFLSATVQPGFVFSTQLAVT
AIAHAIQKLCLNRNCRNPNAFAPWELLQSLKAVNFTYKGRTLFFDSKGDASTGYDVLIWKKGPDGKI
NITPFAEYDTQKGVFRFSTKDKENEFTLLKEIRSRCSDECKPGQMKKTSASQHTCCYECVACPENHY
TNNKDMVYCLQCNNKTHWSPVNSTVCYLKKIEYLRWDDGFAIVLLLISFIGIFIIVAIALLFTKNFDTP
VVKASGGFLCYIILFSILLSFVSAVFFIGKPEDIKCKMRQTMFGISFTVAVACILLKSIKILLAFTFEPKVQS
ILKRLYKPFTLVFVCTGIQIIICTTWLVFWSPYTQENFSLPKTIILECDEGSTVAFGIMLGYIALLTFICFI
FAFRGRKLPENYNEAKFITFGMLIYFIAWITFIPIYATTFGIYLPAVEMIVILISNYGILSCTFLPKCYIILCK
QDTNTKSAFLKIIYKYSAKSASSLTVSHVSSSSLTLPEGSTTVPSVSSRPSVSCISNSFSFHERLVAADIPP
TKARCLQRKRLSSI
>Latimeria_chalumnae_GPRC6A
MKFLSKSNSTKKCVDVRCNYTNYIPTVKAVVGPGYSEISIAVSRILSFFLIPQSCVAPDDLVGARAPGD
VIIGGIFPVHTEVLNLSPERPEAPKCVGFDVRGFLLSLAMIHAIKTINNSTLLPNIKLGYEIYDTCSEAT
MAARVVLKMLADPSNHSVPFQCNYTDYKPRIKAVVGATYSEIAITVSRMLNLQLIPQVSSSSTAEILSD
KNRYPSFVRTVPHDLYQTRAMAQLIGQSGWNWIGVIYTDDDYGRFAIESFISEATKQNICIAFQEVLP
AYLSDDLVHSRIKQTSKKVADEKRVNVVVVFAKAAHVSKLFEDLIQMKVNKTWIASDSWSRSSKVFSV
PNVSKVGKVIGFTFRSGNISTFHNYLKKLKIDYNSDNRFIREFKMLVSNCSDAEESDLCRCNSVSSQDN
PSVHECFMSSAEPAFVYSTLLAVKSVAHAIKDLCKVDNCNNSFEFAPWQLLNAIKKVNFTDDGRSIYF
QKGDSNLGYDVIIWKAVGGTIDPSNIVAEYNLEKQDFFFENEAKKQELKSLQKIQSNCSDECKPGERK
TTAQSPHTCCYDCEVCSENQFTNETDMDHCLDCNNKTHWSPVNSSTCFEKTTEFLKWSDWYAIVL
VAISALGIVLALAVGALFAKNLNTPVVKASGGPLCFIILICLCLSFVSAMFFIGNPIDFQCKIRQVLFGISF
SLCVSCILLKSFKILIAFNFDLTIQKRLKRLYNPYIIVIICTGIQVIICIAWLVSDGPHVYENHLIPKIILLEC
SEGSNVGFGLMLGYIAVLALICFIFAFKGRKLPESYNEAKFITFGMLIYFISWISFVPVYVTTFGKYLPAV
EMIVILISNYGILCCHFFPKCYIILFKKEFSTKSAFQVNVFNYTLRSASNLPVGQMSLDDDRNVEPRLSI
TSINSEKPLFFISDFQKHKVSKCESYPSKDEVLFNCGANTGTRKRLTSV
>Takifugu_rubripes_GPRC6A

MAMVGFIINVHITLFFLILKIGRDTVASTSLPAATAPGDIIIGGIFPIHEDVDKETESFEPHIRPCIRFQQS
GFVLALAMINAIEDMNKSPPLADANITLGYRILDSCSDVSTALRATNDLMQQGNCNSSGSSSSCGQPI
MAVVGASYSETSIAIARQLTLPMIPQISYSSSAVLLSDKTHFPAFMRTIPNDKYQTTAMITLLSHYGWN
WVGIITTDGSYGLSALDQFVSQASAKGICVAFKSILPQSVSSQDTSSAITKTARTIYKNPKVQVIISFAKP
SQMKFLFHKLKSMMLKPGETNGEGRMRRVWVASDSWSTSRYIYGNLTLEDIGYVLGFTFKSGNVSSF
REYLEQLGAPEENIKINPFLQEFYMHMNATAVGSGEDKHVPEALRSLWEHVHADLIFSVEMAVSAIT
QAVATICRRTDCKTLGSVQPWQVLAALWMQEFKLRQKSYKFDSSGDINMGYEIVMWTSVTSEISVH
HVVAEYHPLYSNITFMEQHNFTTKQLLDDLKQVVSKCSNSCIPGQFKKTSEGQHTCCYECINCTENY
YSNSTDMDQCLSCDADTEWSPKGSSSCISKEQLFFSWNDIFAVVLLAFSALGILLCLLTSALFLYQRDT
PVVKAAGGPLSQAILFSLVVSYISAMLFVGEPSSLQCKARQVLFGISFTLCVSCILVKTLQILLAFQFNP
ALQNMLRKIYQPYAIITICVALQTATCICWLVLLSPYAHIIKQPTTLLQYCHEGSYVAFGVMLGYIAILA
FVCFICAFKGRKLPEQYNEAKFITFSMLLYLISWLLFVPIYVTTSGVYLPAVEMVVILISNYGILSCHFFP
KCYIIFFKKEQNTRSAFRKNLYEYSSKHMESSGSSGSNGQHFSCDLPPVPTLSPVTGPTLTSSYKPSVT
QQCHKRSISM
>Danio_rerio_GPRC6A
MDLMSFILLWAGLMKVAEASIAQFSQLGASAPGNIIIGGLFPIHEAVVPVNYTGNNSISAPEHPDCIRF
YTKGLNQALAMINAVEMANKSPMLSSLNITLGYRIYDTCSDVTTALRAVHDIMRPFSDCESPEDSSQP
VQPIMAVIGTTSSEISIAVARDLNLQMIPQISYASTATILSDKSRFPAFMRTVPSDEYQTCAMAKLLKSN
KWSWVGIIITDGDYGRSALEGFIQHTETEGICIAFKAILPDSLADQQKLNTDIENTLNIIENNPKVRVVI
SFAKSSQMQLLFKGLQSRNISNNMVWVASDNWSTAKHILNDGSITDIGKVLGFTFKSGNFTSFHQYL
KNLQFESEDEMNNSFLKEFLKLNAGNASNTVLELMKSTNLDKIFSIEMAVTAVANAVAKLCAERQCQ
DSTALQPWELLRQLRSITFENGGEMYKFDANGDINLGYDLFLWEGDQSDEHADDIIAEYDPTKGGF
HYIHNDLSEIKKVVSRCSNSCQPGQYKKTAEGQHTCCYECLTCVENHYSNITDADECSPCDSESMWS
LANSTECHPKVFEYFDWNSGFAIVLLILAALGVLLLFFMSALFFWQRHSPVVKAAGGPLCHLILVSLL
GSFISVVFFVGEPSDLTCRARQVIFGFSFTLCVSCILVKSLKILLAFEMNFELKELLCMLYKPYMIVSVG
MGVQIIICTVWLTLYKPFKDKEVQTESILLECNEGFYVMFWLMLGYIALLALFCFTFAYIGRKLPQKY
NEAKFITFSMVICLMAWIIFIPIHVTTSGKYVPAVEMVVILISNYGILSCHFLPKSYIILFKKEHNTKDAF
MKNVYEYARKSAENIKGLTGTEPQFKQENSVYTISNLSFVPEEKHE
>Lepisosteus_oculatus_GPRC6A
MSSWLLWLWFISRLCQARSAVPTLIGTIAPGDIVIGGLFPVHQGVVNRVNLSQPQAPACTKFDIGGFT
QLLAMIHAIEEINNSSLLDGLKLGYEIYDSCSDVTTAIRATMAFINTPGNCAGAHCSTTECNPKVKAV
VGDSHSEISIAVARLLNQEMIPQISYASSAVILSDKTRFPTFMRTIPNDNYQTKAMAKLIERSGWNWIG
VISTDGDYGRSAVDSFVSHAAKLGICVAFKEILPDLLSDRNIDTKINQTVETIRSNNKVKVIVSFARPLH
MIQILHFFNKQTLNKIWFASDGWSTSKDAFEKHNVTNVETVVGFTFKNGSLDEFYTYLKNLKSNAET
VKNNIFLKQYALYGSLNDTVNLTSLLEKDFPPLNDTGVKALIDNTYPDDVYSIQMTINAIAYALVNLC
KEKNCKNGSHVQPWELLDALKKTEFKQEGKTYMFNSDGDINSGYNVVLWKTFNGVINMYNSVAEY

VIENDTFTFSSNDTKRLLDDLRGVISKCSNSCTPGQVKKTAEGQHTCCYECINCTENQYSNNTDML
QCYNCDTSVEWSLPGSSSCTPKLLEYFSWNDGFAIFLLGIAALGMLVVVVIGIIFMVHRQTPVVKASG
GPICYVILLSLMISFISSFFFVGEPSNPRCKVRQVLFGLSFACCVSCILVKSLKIILAFQFNPEFKNFLKKT
YKPYVIVPCCVGIQVIICTMWLVFKSPEERKLPLSTTILVECEEGSYVAYGVMLGYIALLALICFICAFKG
RKLPEKYNEAKFITFGMLIYFISWVFFIPVYLTTTGKYLPAVEMVVILISNYGILCCHFFPKCYVILFKKE
NNTKDAFLQNIFEYSKKSEAIRNMKNNKAPEEDAITAEMLKYDEEELADTSNIWTLKENFIQLEEALT
RTGLTINMAKIKYMVMTRKGTITGAALLEIQGYQFKKVSTFKYLGSLISEKNENSLEIRERINAGNRCY
FSVQDLMRPEFCQGE
>Callorhinchus_milii_GPRC6A
MTLLVALAVLCTLSFNTTQSCKIPDDLKGAKAPGDIIIGGLFPVHEKVENLVDLKRPVNQNCKGFDV
RGFVRTLAMIHSIEMVNNSTLLPGVKLGYEIYDTCAEATVAMRAALRFLSNSSSNCVQVQCNYTDYL
PTVKAVVGSSLSEVSIAVARILSLYLMPQISYSSSAEILSDKTRFPAFLRTIPSDYYQTKAMAKLVHISQW
NWVGTISSDDDYGRSGIDNFIADAENLGVCIAFREVIPSYSSDKVTNDRIKRIVNTVVNQSSVNVIIVFA
KGSHVINLFKELSVHNVNKTWIASDSWSTNRNVTHLETIHKIGNIVGFTFKSRNLSKFENYVKTLPINS
AAGNTFLEDYHWLRSICADIQNNELETCISNLTQNPKFIRHSKHNKLTNRRSWEDDFLVNNIEPGFIS
SIHWSVIALAHALRNLLKCNEERCQKSFDFAPWMLLEELKKVNFTDDXEKFQFDSSGEFISGYDIVMW
KSVNGKMEFNHTVAEYNIQEDNFTIKDARFKILKKITSECSKHCQPGEIKQSSQGQHTCCYNCVRCN
NNTFSNTTDAQMCFVCLEDEWAPIKSAKCYKKVFSFLDWTDGFAIVLLAFAAFGIVLIIVIAVIFIKYM
DTPSVKANGGVMCFIMLFSLLCSFASVGFFIGTPTKITCKIRQPLFGISFALCVSCALTKSFKILLAFNFN
PAEQENLKHFYKPWAIKGICTGLQIVICTMWLVFDGPQPHKESKRFPKEILLECNEGSYAAFAVMLGY
IAFLSLICFMFAFKSRKLPENYNEAKFITFSMLIYFISWVTFVPVYVTTQGKYLPAVEMVTILSSTYGILG
CHFFPKCYIILFKKELNTTSSFLKNLYDYSLKSVTVITNSPISVNYSSRESISNSTKIINTTLASHIHFKNSN
CQTSVNSNYPIFANGRTNKFYRKRIASW

Maximum-likelihood tree constructed using RAxML (Extended Data Fig. 1):

((((((((((((Homo_sapiens_T1R1:0.16056928,Mus_musculus_T1R1:0.18335514)100:0.39172688,(((Gekko_japonicus_T
1R1:0.34578109,Pogona_vitticeps_T1R1:0.37875865)50:0.07091790,Anolis_carolinensis_T1R1:0.44827526)100:0.2375
6715,Gallus_gallus_T1R1:0.57683605)68:0.09265936)99:0.16421274,(Rhinatrema_bivittatum_T1R1:0.35908512,(Amb
ystoma_mexicanum_T1R1:0.35277936,(Nanorana_parkeri_T1R1a:0.21284045,Nanorana_parkeri_T1R1b:0.24895685)1
00:0.25802994)81:0.06611583)70:0.04934361)98:0.09850270,(Neoceratodus_forsteri_T1R1:0.25361070,Latimeria_chal
umnae_T1R1:0.37085088)66:0.06992583)89:0.07406624,(Polypterus_senegalus_T1R1:0.41417479,((Lepisosteus_ocula
tus_T1R1:0.19276250,Amia_calva_T1R1:0.23744085)98:0.08951678,(Danio_rerio_T1R1:0.32237541,Takifugu_rubripe
s_T1R1:0.42045789)100:0.22548078)99:0.12013289)100:0.25631671)96:0.12074664,(((((((Takifugu_rubripes_T1R2B
a:0.19687616,Takifugu_rubripes_T1R2Bb:0.20097914)100:0.50459316,(Danio_rerio_T1R2Ba:0.23331678,Danio_rerio
_T1R2Bb:0.13937142)100:0.32776554)100:0.30159801,Amia_calva_T1R2Ba:0.20141564)45:0.05728384,Lepisosteus_o
culatus_T1R2Ba:0.29677380)82:0.08430921,(Lepisosteus_oculatus_T1R2Bb:0.31938480,Amia_calva_T1R2Bb:0.28244
175)100:0.10039080)83:0.06952464,(Lepisosteus_oculatus_T1R2Bc:0.22000999,Amia_calva_T1R2Bc:0.32199124)100:
0.11588961)84:0.09036867,Polypterus_senegalus_T1R2B:0.51915877)100:0.47256988)42:0.04523479,(((((Homo_sapi
ens_T1R2A:0.20318754,Mus_musculus_T1R2A:0.20592353)100:0.40725168,(Gekko_japonicus_T1R2A:0.26380708,(P
ogona_vitticeps_T1R2A:0.34637107,Anolis_carolinensis_T1R2A:0.34044004)73:0.08005017)100:0.23304331)100:0.445
52743,(Latimeria_chalumnae_T1R2Aa:0.39569234,Latimeria_chalumnae_T1R2Ab:0.37907091)100:0.21579067)91:0.1
0980223,((Nanorana_parkeri_T1R2A:0.46800265,Ambystoma_mexicanum_T1R2A:0.35554962)64:0.06487477,Rhinatr
ema_bivittatum_T1R2A:0.25564545)100:0.42152172)74:0.08735337,(Amia_calva_T1R2A:0.74536483,Polypterus_sene
galus_T1R2A:0.65587518)100:0.60462401)69:0.05846734)52:0.03937127,(Protopterus_annectens_T1R5X:0.78144988,
(((Ambystoma_mexicanum_T1R5:0.42221763,Rhinatrema_bivittatum_T1R5:0.22094724)100:0.25579358,(Neoceratod
us_forsteri_T1R5b:0.18626813,(Neoceratodus_forsteri_T1R5a:0.05918115,Protopterus_annectens_T1R5a:0.19150628)
100:0.14065428)100:0.06681723)96:0.07512899,(Latimeria_chalumnae_T1R5a:0.17949962,(Latimeria_chalumnae_T1
R5b:0.11630959,Latimeria_chalumnae_T1R5c:0.04231055)100:0.18879744)100:0.14906786)100:0.30559658)86:0.093
56961)100:0.12101301,((((Chiloscyllium_punctatum_T1R6-1_pseudogene:0.14697684,Rhincodon_typus_T1R6-
1:0.06003315)100:0.09099318,Scyliorhinus_torazame_T1R6-1:0.09769618)100:0.20123076,Callorhinchus_milii_T1R6-
1:0.48677516)100:0.33725096,((((Chiloscyllium_punctatum_T1R6-2:0.07739437,Rhincodon_typus_T1R6-
2:0.06938432)100:0.10873386,Scyliorhinus_torazame_T1R6-2:0.10097620)100:0.32910849,Callorhinchus_milii_T1R6-
2:0.52132777)100:0.14942925,(Callorhinchus_milii_T1R6-3:0.43775875,(Rhincodon_typus_T1R6-
3:0.07508745,Chiloscyllium_punctatum_T1R6-
3:0.07225187)100:0.34072573)100:0.13078339)100:0.19693673)97:0.08316431)98:0.08019472,(((Anolis_carolinensis_
T1R7:0.51600733,Pogona_vitticeps_T1R7:0.25969567)62:0.07389982,Gekko_japonicus_T1R7:0.26915078)100:0.7745
4723,Ambystoma_mexicanum_T1R7:0.53255736)79:0.09909178)83:0.06131072,(Polypterus_senegalus_T1R8:0.622011
63,Protopterus_annectens_T1R8:0.62909057)100:0.24794371)92:0.07959172,(((((Pogona_vitticeps_T1R4:0.30677743,
Anolis_carolinensis_T1R4:0.29903168)100:0.13884835,(Gekko_Japonicus_T1R4:0.13087321,Madagascar_ground_geck

o_T1R4:0.15912347)100:0.17717989)100:0.48066767,(Ambystoma_mexicanum_T1R4:0.37836063,Rhinatrema_bivittat
um_T1R4:0.29970603)100:0.13045627)100:0.17270585,(((Latimeria_chalumnae_T1R4:0.32344272,(Neoceratodus_for
steri_T1R4:0.20949415,(Protopterus_annectens_T1R4a:0.26765235,(Protopterus_annectens_T1R4b:0.18031513,Proto
pterus_annectens_T1R4c:0.14568562)100:0.17248761)100:0.30069679)100:0.24136284)72:0.07481614,(Callorhinchus
_milii_T1R4:0.42764243,(Scyliorhinus_torazame_T1R4:0.12554065,(Rhincodon_typus_T1R4:0.04582680,Chiloscyllium
_punctatum_T1R4:0.06310836)100:0.07104094)100:0.31558512)100:0.29008625)45:0.03933447,Polypterus_senegalus
_T1R4:0.91933664)27:0.04728640)99:0.12920999,((((((Homo_sapiens_T1R3A:0.16032412,Mus_musculus_T1R3A:0.1
7982666)100:0.73494681,(Gallus_gallus_T1R3A:0.46158342,(Gekko_japonicus_T1R3A:0.20711524,(Pogona_vitticeps_
T1R3A:0.30482381,Anolis_carolinensis_T1R3A:0.26026120)99:0.09544270)100:0.20622589)100:0.11469866)94:0.106
21982,(Ambystoma_mexicanum_T1R3A1_pseudogene:0.26899929,Ambystoma_mexicanum_T1R3A2:0.22910987)100:
0.39320504)99:0.12560555,(Neoceratodus_forsteri_T1R3A:0.19204358,Protopterus_annectens_T1R3A:0.32590283)10
0:0.34298426)92:0.07866565,((((Ambystoma_mexicanum_T1R3B1:0.51054915,Nanorana_parkeri_T1R3B1:0.4828657
5)93:0.08259180,Rhinatrema_bivittatum_T1R3B1:0.36486390)100:0.20099490,((Neoceratodus_forsteri_T1R3B_pseud
ogene:0.44441139,Latimeria_chalumnae_T1R3Ba:0.36662085)71:0.06666093,Latimeria_chalumnae_T1R3Bb:0.527998
55)34:0.05459783)49:0.06483871,(((Rhinatrema_bivittatum_T1R3B2:0.37720465,Ambystoma_mexicanum_T1R3B2:0.
44600668)83:0.09540660,Nanorana_parkeri_T1R3B2:0.75688518)100:0.26453756,((((Takifugu_rubripes_T1R3Bb:0.0
4608944,Takifugu_rubripes_T1R3Ba:0.06672586)100:0.42859567,Danio_rerio_T1R3B:0.38514302)100:0.15253068,(A
mia_calva_T1R3B:0.21874580,Lepisosteus_oculatus_T1R3B:0.16134092)95:0.10187501)100:0.19521499,Polypterus_se
negalus_T1R3B:0.45865290)100:0.26458771)50:0.05226709)100:0.21747424)91:0.06494574,((Rhincodon_typus_T1R3
_pseudogene:0.16487704,Scyliorhinus_torazame_T1R3:0.13812536)100:0.43710705,Callorhinchus_milii_T1R3:0.52285
287)100:0.25161638)100:0.18928742)99:0.10977175,(Callorhinchus_milii_GPRC6A:0.31210454,((Lepisosteus_oculatu
s_GPRC6A:0.32639928,(Takifugu_rubripes_GPRC6A:0.40938917,Danio_rerio_GPRC6A:0.45232882)100:0.18446891)
100:0.23972248,(Latimeria_chalumnae_GPRC6A:0.35035367,(Nanorana_parkeri_GPRC6A:0.33824007,((Anolis_caroli
nensis_GPRC6A:0.18059515,Gallus_gallus_GPRC6A:0.20649986)99:0.06939927,(Mus_musculus_GPRC6A:0.1164391
4,Homo_sapiens_GPRC6A:0.10308844)100:0.21226208)100:0.10836495)100:0.11267026)95:0.08176029)100:0.14076
858)100:0.68613167);

Maximum-likelihood tree constructed using IQ-TREE (Extended Data Fig. 2):

(((((((((((Homo_sapiens_T1R1:0.17592999,Mus_musculus_T1R1:0.19093403)100:0.44958154,(((Gekko_japonicus_T
1R1:0.37890106,Pogona_vitticeps_T1R1:0.41399527)54:0.06668076,Anolis_carolinensis_T1R1:0.51588200)100:0.2608
4032,Gallus_gallus_T1R1:0.64462282)73:0.10401971)100:0.17376751,(Rhinatrema_bivittatum_T1R1:0.39312802,(Am
bystoma_mexicanum_T1R1:0.38346988,(Nanorana_parkeri_T1R1a:0.23257741,Nanorana_parkeri_T1R1b:0.26052564)
100:0.27870067)73:0.06958384)78:0.04243183)98:0.09551588,(Neoceratodus_forsteri_T1R1:0.26508772,Latimeria_ch
alumnae_T1R1:0.40161440)76:0.07511549)94:0.07816507,(Polypterus_senegalus_T1R1:0.46917858,((Lepisosteus_ocu
latus_T1R1:0.20803151,Amia_calva_T1R1:0.25296026)100:0.09757897,(Danio_rerio_T1R1:0.34107555,Takifugu_rubri
pes_T1R1:0.47451912)100:0.24369221)100:0.11872934)100:0.29002306)99:0.13780299,((((((Takifugu_rubripes_T1R
2Ba:0.22008146,Takifugu_rubripes_T1R2Bb:0.20179180)100:0.56647614,(Danio_rerio_T1R2Ba:0.24923721,Danio_rer
io_T1R2Bb:0.14141512)100:0.36817657)100:0.33036882,(Amia_calva_T1R2Ba:0.24416544,Lepisosteus_oculatus_T1R
2Ba:0.31181981)52:0.04217619)86:0.07207616,(Lepisosteus_oculatus_T1R2Bb:0.34791614,Amia_calva_T1R2Bb:0.299
74613)100:0.10981589)83:0.05957723,(Lepisosteus_oculatus_T1R2Bc:0.23661473,Amia_calva_T1R2Bc:0.33467530)10
0:0.12910385)87:0.10184786,Polypterus_senegalus_T1R2B:0.57334498)100:0.52490432)79:0.05426745,(((((Homo_sa
piens_T1R2A:0.22150972,Mus_musculus_T1R2A:0.20773799)100:0.46412916,(Gekko_japonicus_T1R2A:0.27953724,(
Pogona_vitticeps_T1R2A:0.37516605,Anolis_carolinensis_T1R2A:0.36109733)73:0.08472428)100:0.25721882)100:0.5
1420113,(Latimeria_chalumnae_T1R2Aa:0.41715049,Latimeria_chalumnae_T1R2Ab:0.42013069)100:0.23945410)95:0
.10505334,((Nanorana_parkeri_T1R2A:0.51255812,Ambystoma_mexicanum_T1R2A:0.37659763)60:0.06168801,Rhina
trema_bivittatum_T1R2A:0.27886578)100:0.46094390)87:0.08811433,(Amia_calva_T1R2A:0.89638472,Polypterus_sen
egalus_T1R2A:0.77082361)100:0.72690000)49:0.05403220)40:0.03805898,(Protopterus_annectens_T1R5X:0.8776186
8,(((Ambystoma_mexicanum_T1R5:0.45276831,Rhinatrema_bivittatum_T1R5:0.23465755)100:0.27889421,(Neocerato
dus_forsteri_T1R5b:0.18782600,(Neoceratodus_forsteri_T1R5a:0.05993189,Protopterus_annectens_T1R5a:0.20171371
)100:0.15147419)99:0.06645978)100:0.07813167,(Latimeria_chalumnae_T1R5a:0.19041329,(Latimeria_chalumnae_T1
R5b:0.12256075,Latimeria_chalumnae_T1R5c:0.04022187)100:0.19243496)100:0.15785915)100:0.32724425)82:0.097
14781)100:0.12423725,((((Chiloscyllium_punctatum_T1R6-1_pseudogene:0.14972859,Rhincodon_typus_T1R6-
1:0.06071432)100:0.09008392,Scyliorhinus_torazame_T1R6-1:0.10145044)100:0.20254093,Callorhinchus_milii_T1R6-
1:0.54583653)100:0.38128142,((((Chiloscyllium_punctatum_T1R6-2:0.07958353,Rhincodon_typus_T1R6-
2:0.06901207)100:0.11942336,Scyliorhinus_torazame_T1R6-2:0.09482314)100:0.37061066,Callorhinchus_milii_T1R6-
2:0.56780417)100:0.16380744,(Callorhinchus_milii_T1R6-3:0.47248459,(Rhincodon_typus_T1R6-
3:0.07410079,Chiloscyllium_punctatum_T1R6-
3:0.07620297)100:0.37114947)99:0.14743549)100:0.20491232)93:0.08611233)100:0.08817443,(((Anolis_carolinensis_
T1R7:0.57031664,Pogona_vitticeps_T1R7:0.27668709)63:0.08314453,Gekko_japonicus_T1R7:0.27615097)100:0.8820
4477,Ambystoma_mexicanum_T1R7:0.59230397)79:0.11081849)78:0.05912835,(Polypterus_senegalus_T1R8:0.685339
46,Protopterus_annectens_T1R8:0.71178555)100:0.30024991)92:0.08253438,((((((Pogona_vitticeps_T1R4:0.3303598
8,Anolis_carolinensis_T1R4:0.31686825)100:0.14571835,(Gekko_Japonicus_T1R4:0.13634811,Madagascar_ground_gec

ko_T1R4:0.16576644)100:0.18809361)100:0.57361762,(Ambystoma_mexicanum_T1R4:0.39938577,Rhinatrema_bivitta
tum_T1R4:0.32935284)97:0.09932798)100:0.21473691,((Neoceratodus_forsteri_T1R4:0.22764121,(Protopterus_annec
tens_T1R4a:0.28703870,(Protopterus_annectens_T1R4b:0.18888338,Protopterus_annectens_T1R4c:0.15099912)100:0.
18340735)100:0.32545284)100:0.26328305,(Latimeria_chalumnae_T1R4:0.34496354,(Callorhinchus_milii_T1R4:0.456
92881,(Scyliorhinus_torazame_T1R4:0.13068845,(Chiloscyllium_punctatum_T1R4:0.06409289,Rhincodon_typus_T1R4
:0.04731160)99:0.07485370)100:0.35463102)100:0.31418850)43:0.05396922)61:0.08598320)66:0.06609639,Polypteru
s_senegalus_T1R4:1.05282431)98:0.10810442,((((((Homo_sapiens_T1R3A:0.16959062,Mus_musculus_T1R3A:0.1873
3723)100:0.82791174,(Gallus_gallus_T1R3A:0.50294867,(Gekko_japonicus_T1R3A:0.21488413,(Pogona_vitticeps_T1
R3A:0.31952934,Anolis_carolinensis_T1R3A:0.28151905)98:0.09897459)100:0.23164078)99:0.13816778)96:0.1104598
5,(Ambystoma_mexicanum_T1R3A1_pseudogene:0.28932240,Ambystoma_mexicanum_T1R3A2:0.23799358)100:0.437
12720)100:0.13289879,(Neoceratodus_forsteri_T1R3A:0.20162324,Protopterus_annectens_T1R3A:0.34201148)100:0.3
7251024)96:0.08363389,((((Ambystoma_mexicanum_T1R3B1:0.56888057,Nanorana_parkeri_T1R3B1:0.52569306)89:
0.08312133,Rhinatrema_bivittatum_T1R3B1:0.38724173)100:0.23688234,((Neoceratodus_forsteri_T1R3B_pseudogene
:0.47690831,Latimeria_chalumnae_T1R3Ba:0.41616989)64:0.06209475,Latimeria_chalumnae_T1R3Bb:0.59249858)38:
0.05660345)60:0.06331576,(((Ambystoma_mexicanum_T1R3B2:0.50519960,Rhinatrema_bivittatum_T1R3B2:0.39536
509)91:0.10880129,Nanorana_parkeri_T1R3B2:0.84136726)100:0.30121441,((((Takifugu_rubripes_T1R3Ba:0.0718017
6,Takifugu_rubripes_T1R3Bb:0.04501753)100:0.47488613,Danio_rerio_T1R3B:0.42688668)99:0.15612527,(Amia_calv
a_T1R3B:0.23935705,Lepisosteus_oculatus_T1R3B:0.16423045)93:0.12029056)100:0.22598227,Polypterus_senegalus_
T1R3B:0.47732467)100:0.29780908)46:0.04438057)100:0.26438578)93:0.07106261,((Rhincodon_typus_T1R3_pseudo
gene:0.17579839,Scyliorhinus_torazame_T1R3:0.13686907)100:0.48165543,Callorhinchus_milii_T1R3:0.56530369)100
:0.26434796)100:0.22009066)99:0.10685588,(Callorhinchus_milii_GPRC6A:0.33827401,((Lepisosteus_oculatus_GPRC
6A:0.34581849,(Danio_rerio_GPRC6A:0.48696929,Takifugu_rubripes_GPRC6A:0.44660416)100:0.19423638)100:0.26
957958,(Latimeria_chalumnae_GPRC6A:0.35837938,(Nanorana_parkeri_GPRC6A:0.35433863,((Anolis_carolinensis_G
PRC6A:0.18088354,Gallus_gallus_GPRC6A:0.20388698)99:0.07235530,(Mus_musculus_GPRC6A:0.12170459,Homo_s
apiens_GPRC6A:0.10184179)100:0.22124034)100:0.10975323)100:0.14844037)76:0.07370149)99:0.15501676)100:0.8
0103961);

Bayesian tree constructed using MrBayes (Extended Data Fig. 3):

```
#NEXUS
[ID: 3955548243]
begin taxa;
	dimensions ntax=120;
	taxlabels
		Homo_sapiens_T1R1
		Mus_musculus_T1R1
		Gallus_gallus_T1R1
		Gekko_japonicus_T1R1
		Pogona_vitticeps_T1R1
		Anolis_carolinensis_T1R1
		Ambystoma_mexicanum_T1R1
		Nanorana_parkeri_T1R1a
		Nanorana_parkeri_T1R1b
		Rhinatrema_bivittatum_T1R1
		Neoceratodus_forsteri_T1R1
		Latimeria_chalumnae_T1R1
		Takifugu_rubripes_T1R1
		Danio_rerio_T1R1
		Lepisosteus_oculatus_T1R1
		Amia_calva_T1R1
		Polypterus_senegalus_T1R1
		Takifugu_rubripes_T1R2Bb
		Takifugu_rubripes_T1R2Ba
		Danio_rerio_T1R2Ba
		Danio_rerio_T1R2Bb
		Lepisosteus_oculatus_T1R2Ba
		Amia_calva_T1R2Ba
		Lepisosteus_oculatus_T1R2Bb
		Amia_calva_T1R2Bb
		Lepisosteus_oculatus_T1R2Bc
		Amia_calva_T1R2Bc
		Polypterus_senegalus_T1R2B
		Homo_sapiens_T1R2A
		Mus_musculus_T1R2A
		Gekko_japonicus_T1R2A
		Pogona_vitticeps_T1R2A
		Anolis_carolinensis_T1R2A
		Ambystoma_mexicanum_T1R2A
		Nanorana_parkeri_T1R2A
		Rhinatrema_bivittatum_T1R2A
		Latimeria_chalumnae_T1R2Ab
		Latimeria_chalumnae_T1R2Aa
		Amia_calva_T1R2A
		Polypterus_senegalus_T1R2A
		Ambystoma_mexicanum_T1R5
		Rhinatrema_bivittatum_T1R5
		Neoceratodus_forsteri_T1R5a
		Neoceratodus_forsteri_T1R5b
		Protopterus_annectens_T1R5a
		Protopterus_annectens_T1R5X
		Latimeria_chalumnae_T1R5a
		Latimeria_chalumnae_T1R5b
		Latimeria_chalumnae_T1R5c
		Callorhinchus_milii_T1R6-1
		Scyliorhinus_torazame_T1R6-1
		Rhincodon_typus_T1R6-1
		Chiloscyllium_punctatum_T1R6-1_pseudogene
		Callorhinchus_milii_T1R6-2
		Rhincodon_typus_T1R6-2
		Scyliorhinus_torazame_T1R6-2
```

Chiloscyllium_punctatum_T1R6-2
Rhincodon_typus_T1R6-3
Chiloscyllium_punctatum_T1R6-3
Callorhinchus_milii_T1R6-3
Gekko_japonicus_T1R7
Pogona_vitticeps_T1R7
Anolis_carolinensis_T1R7
Ambystoma_mexicanum_T1R7
Polypterus_senegalus_T1R8
Protopterus_annectens_T1R8
Homo_sapiens_T1R3A
Mus_musculus_T1R3A
Gallus_gallus_T1R3A
Gekko_japonicus_T1R3A
Pogona_vitticeps_T1R3A
Anolis_carolinensis_T1R3A
Ambystoma_mexicanum_T1R3A1_pseudogene
Ambystoma_mexicanum_T1R3A2
Neoceratodus_forsteri_T1R3A
Protopterus_annectens_T1R3A
Ambystoma_mexicanum_T1R3B1
Ambystoma_mexicanum_T1R3B2
Nanorana_parkeri_T1R3B2
Rhinatrema_bivittatum_T1R3B2
Nanorana_parkeri_T1R3B1
Rhinatrema_bivittatum_T1R3B1
Neoceratodus_forsteri_T1R3B_pseudogene
Latimeria_chalumnae_T1R3Ba
Latimeria_chalumnae_T1R3Bb
Takifugu_rubripes_T1R3Ba
Takifugu_rubripes_T1R3Bb
Danio_rerio_T1R3B
Lepisosteus_oculatus_T1R3B
Amia_calva_T1R3B
Polypterus_senegalus_T1R3B
Callorhinchus_milii_T1R3
Scyliorhinus_torazame_T1R3
Rhincodon_typus_T1R3_pseudogene
Gekko_Japonicus_T1R4
Madagascar_ground_gecko_T1R4
Pogona_vitticeps_T1R4
Anolis_carolinensis_T1R4
Ambystoma_mexicanum_T1R4
Rhinatrema_bivittatum_T1R4
Neoceratodus_forsteri_T1R4
Protopterus_annectens_T1R4a
Protopterus_annectens_T1R4b
Protopterus_annectens_T1R4c
Latimeria_chalumnae_T1R4
Polypterus_senegalus_T1R4
Callorhinchus_milii_T1R4
Scyliorhinus_torazame_T1R4
Chiloscyllium_punctatum_T1R4
Rhincodon_typus_T1R4
Homo_sapiens_GPRC6A
Mus_musculus_GPRC6A
Gallus_gallus_GPRC6A
Anolis_carolinensis_GPRC6A
Nanorana_parkeri_GPRC6A
Latimeria_chalumnae_GPRC6A
Takifugu_rubripes_GPRC6A
Danio_rerio_GPRC6A
Lepisosteus_oculatus_GPRC6A
Callorhinchus_milii_GPRC6A
;

```
end;
begin trees;
	translate
		1	Homo_sapiens_T1R1,
		2	Mus_musculus_T1R1,
		3	Gallus_gallus_T1R1,
		4	Gekko_japonicus_T1R1,
		5	Pogona_vitticeps_T1R1,
		6	Anolis_carolinensis_T1R1,
		7	Ambystoma_mexicanum_T1R1,
		8	Nanorana_parkeri_T1R1a,
		9	Nanorana_parkeri_T1R1b,
		10	Rhinatrema_bivittatum_T1R1,
		11	Neoceratodus_forsteri_T1R1,
		12	Latimeria_chalumnae_T1R1,
		13	Takifugu_rubripes_T1R1,
		14	Danio_rerio_T1R1,
		15	Lepisosteus_oculatus_T1R1,
		16	Amia_calva_T1R1,
		17	Polypterus_senegalus_T1R1,
		18	Takifugu_rubripes_T1R2Bb,
		19	Takifugu_rubripes_T1R2Ba,
		20	Danio_rerio_T1R2Ba,
		21	Danio_rerio_T1R2Bb,
		22	Lepisosteus_oculatus_T1R2Ba,
		23	Amia_calva_T1R2Ba,
		24	Lepisosteus_oculatus_T1R2Bb,
		25	Amia_calva_T1R2Bb,
		26	Lepisosteus_oculatus_T1R2Bc,
		27	Amia_calva_T1R2Bc,
		28	Polypterus_senegalus_T1R2B,
		29	Homo_sapiens_T1R2A,
		30	Mus_musculus_T1R2A,
		31	Gekko_japonicus_T1R2A,
		32	Pogona_vitticeps_T1R2A,
		33	Anolis_carolinensis_T1R2A,
		34	Ambystoma_mexicanum_T1R2A,
		35	Nanorana_parkeri_T1R2A,
		36	Rhinatrema_bivittatum_T1R2A,
		37	Latimeria_chalumnae_T1R2Ab,
		38	Latimeria_chalumnae_T1R2Aa,
		39	Amia_calva_T1R2A,
		40	Polypterus_senegalus_T1R2A,
		41	Ambystoma_mexicanum_T1R5,
		42	Rhinatrema_bivittatum_T1R5,
		43	Neoceratodus_forsteri_T1R5a,
		44	Neoceratodus_forsteri_T1R5b,
		45	Protopterus_annectens_T1R5a,
		46	Protopterus_annectens_T1R5X,
		47	Latimeria_chalumnae_T1R5a,
		48	Latimeria_chalumnae_T1R5b,
		49	Latimeria_chalumnae_T1R5c,
		50	Callorhinchus_milii_T1R6-1,
		51	Scyliorhinus_torazame_T1R6-1,
		52	Rhincodon_typus_T1R6-1,
		53	Chiloscyllium_punctatum_T1R6-1_pseudogene,
		54	Callorhinchus_milii_T1R6-2,
		55	Rhincodon_typus_T1R6-2,
		56	Scyliorhinus_torazame_T1R6-2,
		57	Chiloscyllium_punctatum_T1R6-2,
		58	Rhincodon_typus_T1R6-3,
		59	Chiloscyllium_punctatum_T1R6-3,
		60	Callorhinchus_milii_T1R6-3,
		61	Gekko_japonicus_T1R7,
		62	Pogona_vitticeps_T1R7,
```

63 Anolis_carolinensis_T1R7,
64 Ambystoma_mexicanum_T1R7,
65 Polypterus_senegalus_T1R8,
66 Protopterus_annectens_T1R8,
67 Homo_sapiens_T1R3A,
68 Mus_musculus_T1R3A,
69 Gallus_gallus_T1R3A,
70 Gekko_japonicus_T1R3A,
71 Pogona_vitticeps_T1R3A,
72 Anolis_carolinensis_T1R3A,
73 Ambystoma_mexicanum_T1R3A1_pseudogene,
74 Ambystoma_mexicanum_T1R3A2,
75 Neoceratodus_forsteri_T1R3A,
76 Protopterus_annectens_T1R3A,
77 Ambystoma_mexicanum_T1R3B1,
78 Ambystoma_mexicanum_T1R3B2,
79 Nanorana_parkeri_T1R3B2,
80 Rhinatrema_bivittatum_T1R3B2,
81 Nanorana_parkeri_T1R3B1,
82 Rhinatrema_bivittatum_T1R3B1,
83 Neoceratodus_forsteri_T1R3B_pseudogene,
84 Latimeria_chalumnae_T1R3Ba,
85 Latimeria_chalumnae_T1R3Bb,
86 Takifugu_rubripes_T1R3Ba,
87 Takifugu_rubripes_T1R3Bb,
88 Danio_rerio_T1R3B,
89 Lepisosteus_oculatus_T1R3B,
90 Amia_calva_T1R3B,
91 Polypterus_senegalus_T1R3B,
92 Callorhinchus_milii_T1R3,
93 Scyliorhinus_torazame_T1R3,
94 Rhincodon_typus_T1R3_pseudogene,
95 Gekko_Japonicus_T1R4,
96 Madagascar_ground_gecko_T1R4,
97 Pogona_vitticeps_T1R4,
98 Anolis_carolinensis_T1R4,
99 Ambystoma_mexicanum_T1R4,
100 Rhinatrema_bivittatum_T1R4,
101 Neoceratodus_forsteri_T1R4,
102 Protopterus_annectens_T1R4a,
103 Protopterus_annectens_T1R4b,
104 Protopterus_annectens_T1R4c,
105 Latimeria_chalumnae_T1R4,
106 Polypterus_senegalus_T1R4,
107 Callorhinchus_milii_T1R4,
108 Scyliorhinus_torazame_T1R4,
109 Chiloscyllium_punctatum_T1R4,
110 Rhincodon_typus_T1R4,
111 Homo_sapiens_GPRC6A,
112 Mus_musculus_GPRC6A,
113 Gallus_gallus_GPRC6A,
114 Anolis_carolinensis_GPRC6A,
115 Nanorana_parkeri_GPRC6A,
116 Latimeria_chalumnae_GPRC6A,
117 Takifugu_rubripes_GPRC6A,
118 Danio_rerio_GPRC6A,
119 Lepisosteus_oculatus_GPRC6A,
120 Callorhinchus_milii_GPRC6A
;

tree con_50_majrule = [&U] (1[&prob=1.00000000e+00,prob_stddev=0.00000000e+00,prob_range={1.00000000e+00,1.00000000e+00},prob(percent)="100",prob+-sd="100+-0"]:1.525656e-01[&length_mean=1.53306875e-01,length_median=1.52565600e-01,length_95%HPD={1.15509700e-01,1.90478500e-01}],2[&prob=1.00000000e+00,prob_stddev=0.00000000e+00,prob_range={1.00000000e+00,1.00000000e+00},prob(percent)="100",prob+-sd="100+-0"]:1.725923e-01[&length_mean=1.73180334e-01,length_median=1.72592300e-01,length_95%HPD={1.35276200e-01,2.13419900e-01}],((3[&prob=1.00000000e+00,prob_stddev=0.00000000e+00,prob_range={1.00000000e+00,1.00000000e+00},prob(percent)="100",prob+-sd="100+-

0"]:5.380719e-01[&length_mean=5.38871885e-01,length_median=5.38071900e-01,length_95%HPD={4.57738800e-01,6.22357800e-
01}],((4[&prob=1.00000000e+00,prob_stddev=0.00000000e+00,prob_range={1.00000000e+00,1.00000000e+00},prob(percent)="100",prob+-sd="100+-
0"]:3.189413e-01[&length_mean=3.19993572e-01,length_median=3.18941300e-01,length_95%HPD={2.62212400e-01,3.82719900e-
01}],5[&prob=1.00000000e+00,prob_stddev=0.00000000e+00,prob_range={1.00000000e+00,1.00000000e+00},prob(percent)="100",prob+-sd="100+-
0"]:3.538714e-01[&length_mean=3.54931215e-01,length_median=3.53871400e-01,length_95%HPD={2.91624100e-01,4.17533200e-
01}])[&prob=9.45847389e-01,prob_stddev=8.39088859e-04,prob_range={9.45254063e-01,9.46440714e-01},prob(percent)="95",prob+-sd="95+-
0"]:6.576081e-02[&length_mean=6.69464445e-02,length_median=6.57608100e-02,length_95%HPD={2.60311800e-02,1.09989900e-
01}],6[&prob=1.00000000e+00,prob_stddev=0.00000000e+00,prob_range={1.00000000e+00,1.00000000e+00},prob(percent)="100",prob+-sd="100+-
0"]:4.199212e-01[&length_mean=4.21460280e-01,length_median=4.19921200e-01,length_95%HPD={3.53507400e-01,4.92945300e-
01}])[&prob=1.00000000e+00,prob_stddev=0.00000000e+00,prob_range={1.00000000e+00,1.00000000e+00},prob(percent)="100",prob+-sd="100+-
0"]:2.238272e-01[&length_mean=2.24977990e-01,length_median=2.23827200e-01,length_95%HPD={1.58661500e-01,2.91245600e-
01}])[&prob=9.86560179e-01,prob_stddev=4.14830447e-04,prob_range={9.86266850e-01,9.86853509e-01},prob(percent)="99",prob+-sd="99+-
0"]:9.404247e-02[&length_mean=9.55216776e-02,length_median=9.40424700e-02,length_95%HPD={4.71755800e-02,1.48932900e-
01}],(((7[&prob=1.00000000e+00,prob_stddev=0.00000000e+00,prob_range={1.00000000e+00,1.00000000e+00},prob(percent)="100",prob+-sd="100+-
0"]:3.253840e-01[&length_mean=3.26024679e-01,length_median=3.25384000e-01,length_95%HPD={2.70150500e-01,3.81145700e-
01}],(8[&prob=1.00000000e+00,prob_stddev=0.00000000e+00,prob_range={1.00000000e+00,1.00000000e+00},prob(percent)="100",prob+-sd="100+-
0"]:2.032052e-01[&length_mean=2.04032591e-01,length_median=2.03205200e-01,length_95%HPD={1.60863600e-01,2.46208200e-
01}],9[&prob=1.00000000e+00,prob_stddev=0.00000000e+00,prob_range={1.00000000e+00,1.00000000e+00},prob(percent)="100",prob+-sd="100+-
0"]:2.285506e-01[&length_mean=2.29487177e-01,length_median=2.28550600e-01,length_95%HPD={1.84294200e-01,2.76371200e-
01}])[&prob=1.00000000e+00,prob_stddev=0.00000000e+00,prob_range={1.00000000e+00,1.00000000e+00},prob(percent)="100",prob+-sd="100+-
0"]:2.425976e-01[&length_mean=2.43703674e-01,length_median=2.42597600e-01,length_95%HPD={1.93560400e-01,2.97343100e-
01}])[&prob=9.92660098e-01,prob_stddev=4.05402483e-04,prob_range={9.92373435e-01,9.92946761e-01},prob(percent)="99",prob+-sd="99+-
0"]:6.219248e-02[&length_mean=6.30052042e-02,length_median=6.21924800e-02,length_95%HPD={2.87001300e-02,9.85268100e-
02}],10[&prob=1.00000000e+00,prob_stddev=0.00000000e+00,prob_range={1.00000000e+00,1.00000000e+00},prob(percent)="100",prob+-sd="100+-
0"]:3.303849e-01[&length_mean=3.31130098e-01,length_median=3.30384900e-01,length_95%HPD={2.78530400e-01,3.84525000e-
01}])[&prob=9.98833349e-01,prob_stddev=2.35699118e-04,prob_range={9.98666684e-01,9.99000013e-01},prob(percent)="100",prob+-sd="100+-
0"]:4.825047e-02[&length_mean=4.93168957e-02,length_median=4.82504700e-02,length_95%HPD={2.10813700e-02,8.02083800e-
02}],((11[&prob=1.00000000e+00,prob_stddev=0.00000000e+00,prob_range={1.00000000e+00,1.00000000e+00},prob(percent)="100",prob+-sd="100+-
0"]:2.351337e-01[&length_mean=2.35722091e-01,length_median=2.35133700e-01,length_95%HPD={1.87970800e-01,2.80521700e-
01}],12[&prob=1.00000000e+00,prob_stddev=0.00000000e+00,prob_range={1.00000000e+00,1.00000000e+00},prob(percent)="100",prob+-sd="100+-
0"]:3.442538e-01[&length_mean=3.44745702e-01,length_median=3.44253800e-01,length_95%HPD={2.90067600e-01,3.99163800e-
01}])[&prob=9.88860149e-01,prob_stddev=5.93961777e-04,prob_range={9.88440154e-01,9.89280143e-01},prob(percent)="99",prob+-sd="99+-
0"]:6.539982e-02[&length_mean=6.62694557e-02,length_median=6.53998200e-02,length_95%HPD={3.34355100e-02,9.98998700e-
02}],((((13[&prob=1.00000000e+00,prob_stddev=0.00000000e+00,prob_range={1.00000000e+00,1.00000000e+00},prob(percent)="100",prob+-sd="100+-
0"]:3.910458e-01[&length_mean=3.92454958e-01,length_median=3.91045800e-01,length_95%HPD={3.26807200e-01,4.58692200e-
01}],14[&prob=1.00000000e+00,prob_stddev=0.00000000e+00,prob_range={1.00000000e+00,1.00000000e+00},prob(percent)="100",prob+-sd="100+-
0"]:2.981265e-01[&length_mean=2.98793310e-01,length_median=2.98126500e-01,length_95%HPD={2.40196200e-01,3.56712600e-
01}])[&prob=1.00000000e+00,prob_stddev=0.00000000e+00,prob_range={1.00000000e+00,1.00000000e+00},prob(percent)="100",prob+-sd="100+-
0"]:2.097268e-01[&length_mean=2.10499834e-01,length_median=2.09726800e-01,length_95%HPD={1.57091400e-01,2.65030200e-
01}],(15[&prob=1.00000000e+00,prob_stddev=0.00000000e+00,prob_range={1.00000000e+00,1.00000000e+00},prob(percent)="100",prob+-sd="100+-
0"]:1.816520e-01[&length_mean=1.82336234e-01,length_median=1.81652000e-01,length_95%HPD={1.42461100e-01,2.26040300e-
01}],16[&prob=1.00000000e+00,prob_stddev=0.00000000e+00,prob_range={1.00000000e+00,1.00000000e+00},prob(percent)="100",prob+-sd="100+-
0"]:2.210414e-01[&length_mean=2.21744982e-01,length_median=2.21041400e-01,length_95%HPD={1.77826900e-01,2.65571500e-
01}])[&prob=1.00000000e+00,prob_stddev=0.00000000e+00,prob_range={1.00000000e+00,1.00000000e+00},prob(percent)="100",prob+-sd="100+-
0"]:8.723028e-02[&length_mean=8.79436849e-02,length_median=8.72302800e-02,length_95%HPD={5.23401400e-02,1.24645900e-
01}])[&prob=1.00000000e+00,prob_stddev=0.00000000e+00,prob_range={1.00000000e+00,1.00000000e+00},prob(percent)="100",prob+-sd="100+-
0"]:1.112686e-01[&length_mean=1.12186942e-01,length_median=1.11268600e-01,length_95%HPD={6.92732700e-02,1.56151800e-
01}],17[&prob=1.00000000e+00,prob_stddev=0.00000000e+00,prob_range={1.00000000e+00,1.00000000e+00},prob(percent)="100",prob+-sd="100+-
0"]:3.813255e-01[&length_mean=3.82596481e-01,length_median=3.81325500e-01,length_95%HPD={3.21889900e-01,4.47051000e-
01}])[&prob=1.00000000e+00,prob_stddev=0.00000000e+00,prob_range={1.00000000e+00,1.00000000e+00},prob(percent)="100",prob+-sd="100+-
0"]:2.378836e-01[&length_mean=2.39028781e-01,length_median=2.37883600e-01,length_95%HPD={1.87217100e-01,2.94553500e-
01}],((((((((18[&prob=1.00000000e+00,prob_stddev=0.00000000e+00,prob_range={1.00000000e+00,1.00000000e+00},prob(percent)="100",prob+-
sd="100+-0"]:1.906083e-01[&length_mean=1.91445953e-01,length_median=1.90608300e-01,length_95%HPD={1.45815000e-01,2.38671900e-
01}],19[&prob=1.00000000e+00,prob_stddev=0.00000000e+00,prob_range={1.00000000e+00,1.00000000e+00},prob(percent)="100",prob+-sd="100+-
0"]:1.823406e-01[&length_mean=1.82976626e-01,length_median=1.82340600e-01,length_95%HPD={1.36930000e-01,2.29167600e-
01}])[&prob=1.00000000e+00,prob_stddev=0.00000000e+00,prob_range={1.00000000e+00,1.00000000e+00},prob(percent)="100",prob+-sd="100+-
0"]:4.702412e-01[&length_mean=4.71608653e-01,length_median=4.70241200e-01,length_95%HPD={3.90758000e-01,5.54090500e-
01}],(20[&prob=1.00000000e+00,prob_stddev=0.00000000e+00,prob_range={1.00000000e+00,1.00000000e+00},prob(percent)="100",prob+-sd="100+-
0"]:2.166023e-01[&length_mean=2.17345858e-01,length_median=2.16602300e-01,length_95%HPD={1.71475200e-01,2.62584000e-
01}],21[&prob=1.00000000e+00,prob_stddev=0.00000000e+00,prob_range={1.00000000e+00,1.00000000e+00},prob(percent)="100",prob+-sd="100+-
0"]:1.321919e-01[&length_mean=1.32876779e-01,length_median=1.32191900e-01,length_95%HPD={9.46422000e-02,1.72325300e-
01}])[&prob=1.00000000e+00,prob_stddev=0.00000000e+00,prob_range={1.00000000e+00,1.00000000e+00},prob(percent)="100",prob+-sd="100+-
0"]:3.055205e-01[&length_mean=3.07075488e-01,length_median=3.05520500e-01,length_95%HPD={2.38520400e-01,3.76192400e-
01}])[&prob=1.00000000e+00,prob_stddev=0.00000000e+00,prob_range={1.00000000e+00,1.00000000e+00},prob(percent)="100",prob+-sd="100+-
0"]:2.786188e-01[&length_mean=2.79722689e-01,length_median=2.78618800e-01,length_95%HPD={2.15957800e-01,3.47071700e-

01}],23[&prob=1.00000000e+00,prob_stddev=0.00000000e+00,prob_range={1.00000000e+00,1.00000000e+00},prob(percent)="100",prob+-sd="100+-0"]:1.916247e-01[&length_mean=1.92810146e-01,length_median=1.91624700e-01,length_95%HPD={1.45200800e-01,2.41448000e-01}])[&prob=8.98601352e-01,prob_stddev=1.97987259e-03,prob_range={8.97201371e-01,9.00001333e-01},prob(percent)="90",prob+-sd="90+-0"]:5.597140e-02[&length_mean=5.69227137e-02,length_median=5.59714000e-02,length_95%HPD={2.41382200e-02,8.99543100e-02}],22[&prob=1.00000000e+00,prob_stddev=0.00000000e+00,prob_range={1.00000000e+00,1.00000000e+00},prob(percent)="100",prob+-sd="100+-0"]:2.748163e-01[&length_mean=2.75559151e-01,length_median=2.74816300e-01,length_95%HPD={2.28273600e-01,3.23764100e-01}])[&prob=1.00000000e+00,prob_stddev=0.00000000e+00,prob_range={1.00000000e+00,1.00000000e+00},prob(percent)="100",prob+-sd="100+-0"]:7.824052e-02[&length_mean=7.90838705e-02,length_median=7.82405200e-02,length_95%HPD={4.75984600e-02,1.11589300e-01}],(24[&prob=1.00000000e+00,prob_stddev=0.00000000e+00,prob_range={1.00000000e+00,1.00000000e+00},prob(percent)="100",prob+-sd="100+-0"]:2.999570e-01[&length_mean=3.00538662e-01,length_median=2.99957000e-01,length_95%HPD={2.49880200e-01,3.50476800e-01}],25[&prob=1.00000000e+00,prob_stddev=0.00000000e+00,prob_range={1.00000000e+00,1.00000000e+00},prob(percent)="100",prob+-sd="100+-0"]:2.647271e-01[&length_mean=2.65670875e-01,length_median=2.64727100e-01,length_95%HPD={2.18059500e-01,3.15448700e-01}])[&prob=1.00000000e+00,prob_stddev=0.00000000e+00,prob_range={1.00000000e+00,1.00000000e+00},prob(percent)="100",prob+-sd="100+-0"]:9.365822e-02[&length_mean=9.45422845e-02,length_median=9.36582200e-02,length_95%HPD={6.12293600e-02,1.30829800e-01}])[&prob=1.00000000e+00,prob_stddev=0.00000000e+00,prob_range={1.00000000e+00,1.00000000e+00},prob(percent)="100",prob+-sd="100+-0"]:6.626051e-02[&length_mean=6.70917601e-02,length_median=6.62605100e-02,length_95%HPD={3.75308200e-02,9.78180400e-02}],(26[&prob=1.00000000e+00,prob_stddev=0.00000000e+00,prob_range={1.00000000e+00,1.00000000e+00},prob(percent)="100",prob+-sd="100+-0"]:2.083219e-01[&length_mean=2.08796653e-01,length_median=2.08321900e-01,length_95%HPD={1.65045300e-01,2.52281400e-01}],27[&prob=1.00000000e+00,prob_stddev=0.00000000e+00,prob_range={1.00000000e+00,1.00000000e+00},prob(percent)="100",prob+-sd="100+-0"]:2.995262e-01[&length_mean=3.00279105e-01,length_median=2.99526200e-01,length_95%HPD={2.50077800e-01,3.53777600e-01}])[&prob=1.00000000e+00,prob_stddev=0.00000000e+00,prob_range={1.00000000e+00,1.00000000e+00},prob(percent)="100",prob+-sd="100+-0"]:1.093029e-01[&length_mean=1.09792881e-01,length_median=1.09302900e-01,length_95%HPD={7.27016500e-02,1.46751600e-01}])[&prob=9.99973334e-01,prob_stddev=1.88559294e-05,prob_range={9.99960001e-01,9.99986667e-01},prob(percent)="100",prob+-sd="100+-0"]:8.752799e-02[&length_mean=8.86282800e-02,length_median=8.75279900e-02,length_95%HPD={4.33650300e-02,1.35126200e-01}],28[&prob=1.00000000e+00,prob_stddev=0.00000000e+00,prob_range={1.00000000e+00,1.00000000e+00},prob(percent)="100",prob+-sd="100+-0"]:4.751849e-01[&length_mean=4.76306699e-01,length_median=4.75184900e-01,length_95%HPD={4.03586000e-01,5.52525800e-01}])[&prob=1.00000000e+00,prob_stddev=0.00000000e+00,prob_range={1.00000000e+00,1.00000000e+00},prob(percent)="100",prob+-sd="100+-0"]:4.359948e-01[&length_mean=4.36860156e-01,length_median=4.35994800e-01,length_95%HPD={3.63372300e-01,5.11828400e-01}],((((((29[&prob=1.00000000e+00,prob_stddev=0.00000000e+00,prob_range={1.00000000e+00,1.00000000e+00},prob(percent)="100",prob+-sd="100+-0"]:1.939600e-01[&length_mean=1.94490731e-01,length_median=1.93960000e-01,length_95%HPD={1.45710200e-01,2.42447900e-01}],30[&prob=1.00000000e+00,prob_stddev=0.00000000e+00,prob_range={1.00000000e+00,1.00000000e+00},prob(percent)="100",prob+-sd="100+-0"]:1.921810e-01[&length_mean=1.92827923e-01,length_median=1.92181000e-01,length_95%HPD={1.45508800e-01,2.41699000e-01}])[&prob=1.00000000e+00,prob_stddev=0.00000000e+00,prob_range={1.00000000e+00,1.00000000e+00},prob(percent)="100",prob+-sd="100+-0"]:3.809955e-01[&length_mean=3.82003725e-01,length_median=3.80995500e-01,length_95%HPD={3.08005100e-01,4.57823600e-01}],(31[&prob=1.00000000e+00,prob_stddev=0.00000000e+00,prob_range={1.00000000e+00,1.00000000e+00},prob(percent)="100",prob+-sd="100+-0"]:2.444734e-01[&length_mean=2.44923069e-01,length_median=2.44473400e-01,length_95%HPD={1.92180300e-01,2.97595300e-01}],(32[&prob=1.00000000e+00,prob_stddev=0.00000000e+00,prob_range={1.00000000e+00,1.00000000e+00},prob(percent)="100",prob+-sd="100+-0"]:3.232830e-01[&length_mean=3.24174085e-01,length_median=3.23283000e-01,length_95%HPD={2.67454900e-01,3.81627700e-01}],33[&prob=1.00000000e+00,prob_stddev=0.00000000e+00,prob_range={1.00000000e+00,1.00000000e+00},prob(percent)="100",prob+-sd="100+-0"]:3.180854e-01[&length_mean=3.19003685e-01,length_median=3.18085400e-01,length_95%HPD={2.62594000e-01,3.78655300e-01}])[&prob=9.95560059e-01,prob_stddev=5.46821953e-04,prob_range={9.95173398e-01,9.95946721e-01},prob(percent)="100",prob+-sd="100+-0"]:7.977248e-02[&length_mean=8.05405937e-02,length_median=7.97724800e-02,length_95%HPD={4.11375900e-02,1.20912000e-01}])[&prob=1.00000000e+00,prob_stddev=0.00000000e+00,prob_range={1.00000000e+00,1.00000000e+00},prob(percent)="100",prob+-sd="100+-0"]:2.181884e-01[&length_mean=2.19518376e-01,length_median=2.18188400e-01,length_95%HPD={1.53415900e-01,2.84076800e-01}])[&prob=1.00000000e+00,prob_stddev=0.00000000e+00,prob_range={1.00000000e+00,1.00000000e+00},prob(percent)="100",prob+-sd="100+-0"]:4.151186e-01[&length_mean=4.16318750e-01,length_median=4.15118600e-01,length_95%HPD={3.33471200e-01,4.99204400e-01}],(37[&prob=1.00000000e+00,prob_stddev=0.00000000e+00,prob_range={1.00000000e+00,1.00000000e+00},prob(percent)="100",prob+-sd="100+-0"]:3.483174e-01[&length_mean=3.50032323e-01,length_median=3.48317400e-01,length_95%HPD={2.90259000e-01,4.16391800e-01}],38[&prob=1.00000000e+00,prob_stddev=0.00000000e+00,prob_range={1.00000000e+00,1.00000000e+00},prob(percent)="100",prob+-sd="100+-0"]:3.684328e-01[&length_mean=3.69230502e-01,length_median=3.68432800e-01,length_95%HPD={3.04896500e-01,4.37229000e-01}])[&prob=1.00000000e+00,prob_stddev=0.00000000e+00,prob_range={1.00000000e+00,1.00000000e+00},prob(percent)="100",prob+-sd="100+-0"]:2.004770e-01[&length_mean=2.01606681e-01,length_median=2.00477000e-01,length_95%HPD={1.41311900e-01,2.62237900e-01}])[&prob=9.99973334e-01,prob_stddev=3.77118588e-05,prob_range={9.99946667e-01,1.00000000e+00},prob(percent)="100",prob+-sd="100+-0"]:1.019997e-01[&length_mean=1.03127486e-01,length_median=1.01999700e-01,length_95%HPD={5.48882000e-02,1.53170600e-01}],((34[&prob=1.00000000e+00,prob_stddev=0.00000000e+00,prob_range={1.00000000e+00,1.00000000e+00},prob(percent)="100",prob+-sd="100+-0"]:3.326372e-01[&length_mean=3.33285004e-01,length_median=3.32637200e-01,length_95%HPD={2.76661900e-01,3.91980700e-01}],35[&prob=1.00000000e+00,prob_stddev=0.00000000e+00,prob_range={1.00000000e+00,1.00000000e+00},prob(percent)="100",prob+-sd="100+-0"]:4.264868e-01[&length_mean=4.27492118e-01,length_median=4.26486800e-01,length_95%HPD={3.60251600e-01,4.95958200e-01}])[&prob=9.59707204e-01,prob_stddev=5.09110094e-04,prob_range={9.59347209e-01,9.60067199e-01},prob(percent)="96",prob+-sd="96+-0"]:6.070113e-02[&length_mean=6.15495285e-02,length_median=6.07011300e-02,length_95%HPD={2.17943300e-02,1.02159500e-01}],36[&prob=1.00000000e+00,prob_stddev=0.00000000e+00,prob_range={1.00000000e+00,1.00000000e+00},prob(percent)="100",prob+-sd="100+-0"]:2.411233e-01[&length_mean=2.41765078e-01,length_median=2.41123300e-01,length_95%HPD={1.88427800e-01,2.95424900e-01}])[&prob=1.00000000e+00,prob_stddev=0.00000000e+00,prob_range={1.00000000e+00,1.00000000e+00},prob(percent)="100",prob+-sd="100+-0"]:3.856374e-01[&length_mean=3.86672152e-01,length_median=3.85637400e-01,length_95%HPD={3.16060000e-01,4.60350100e-01}])[&prob=9.87966827e-01,prob_stddev=2.47955472e-03,prob_range={9.86213517e-01,9.89720137e-01},prob(percent)="99",prob+-sd="99+-

0"]:7.141240e-02[&length_mean=7.27083858e-02,length_median=7.14124000e-02,length_95%HPD={2.89736500e-02,1.17423000e-
01}],(39[&prob=1.00000000e+00,prob_stddev=0.00000000e+00,prob_range={1.00000000e+00,1.00000000e+00},prob(percent)="100",prob+-sd="100+-
0"]:6.834261e-01[&length_mean=6.85179575e-01,length_median=6.83426100e-01,length_95%HPD={5.71116500e-01,8.05100400e-
01}],40[&prob=1.00000000e+00,prob_stddev=0.00000000e+00,prob_range={1.00000000e+00,1.00000000e+00},prob(percent)="100",prob+-sd="100+-
0"]:6.028898e-01[&length_mean=6.04445114e-01,length_median=6.02889800e-01,length_95%HPD={4.87685900e-01,7.15693600e-
01}])[&prob=1.00000000e+00,prob_stddev=0.00000000e+00,prob_range={1.00000000e+00,1.00000000e+00},prob(percent)="100",prob+-sd="100+-
0"]:5.491824e-01[&length_mean=5.50482173e-01,length_median=5.49182400e-01,length_95%HPD={4.40337500e-01,6.60360100e-
01}])[&prob=9.88446821e-01,prob_stddev=6.69385494e-04,prob_range={9.87973494e-01,9.88920148e-01},prob(percent)="99",prob+-sd="99+-
0"]:6.131322e-02[&length_mean=6.27395595e-02,length_median=6.13132200e-02,length_95%HPD={2.32571100e-02,1.06190700e-
01}],(((((41[&prob=1.00000000e+00,prob_stddev=0.00000000e+00,prob_range={1.00000000e+00,1.00000000e+00},prob(percent)="100",prob+-sd="100+-
0"]:3.966967e-01[&length_mean=3.97466122e-01,length_median=3.96696700e-01,length_95%HPD={3.36976400e-01,4.61012500e-
01}],42[&prob=1.00000000e+00,prob_stddev=0.00000000e+00,prob_range={1.00000000e+00,1.00000000e+00},prob(percent)="100",prob+-sd="100+-
0"]:2.058648e-01[&length_mean=2.06624410e-01,length_median=2.05864800e-01,length_95%HPD={1.59072100e-01,2.53366600e-
01}])[&prob=1.00000000e+00,prob_stddev=0.00000000e+00,prob_range={1.00000000e+00,1.00000000e+00},prob(percent)="100",prob+-sd="100+-
0"]:2.403805e-01[&length_mean=2.41366869e-01,length_median=2.40380500e-01,length_95%HPD={1.89434800e-01,2.97543800e-
01}],((43[&prob=1.00000000e+00,prob_stddev=0.00000000e+00,prob_range={1.00000000e+00,1.00000000e+00},prob(percent)="100",prob+-sd="100+-
0"]:5.531698e-02[&length_mean=5.58769408e-02,length_median=5.53169800e-02,length_95%HPD={3.51080700e-02,7.73469900e-
02}],45[&prob=1.00000000e+00,prob_stddev=0.00000000e+00,prob_range={1.00000000e+00,1.00000000e+00},prob(percent)="100",prob+-sd="100+-
0"]:1.806507e-01[&length_mean=1.81188596e-01,length_median=1.80650700e-01,length_95%HPD={1.49169100e-01,2.16034900e-
01}])[&prob=1.00000000e+00,prob_stddev=0.00000000e+00,prob_range={1.00000000e+00,1.00000000e+00},prob(percent)="100",prob+-sd="100+-
0"]:1.323968e-01[&length_mean=1.33028487e-01,length_median=1.32396800e-01,length_95%HPD={1.00191300e-01,1.66477700e-
01}],44[&prob=1.00000000e+00,prob_stddev=0.00000000e+00,prob_range={1.00000000e+00,1.00000000e+00},prob(percent)="100",prob+-sd="100+-
0"]:1.737092e-01[&length_mean=1.74228495e-01,length_median=1.73709200e-01,length_95%HPD={1.38437000e-01,2.11524500e-
01}])[&prob=1.00000000e+00,prob_stddev=0.00000000e+00,prob_range={1.00000000e+00,1.00000000e+00},prob(percent)="100",prob+-sd="100+-
0"]:6.393313e-02[&length_mean=6.48722927e-02,length_median=6.39331300e-02,length_95%HPD={3.59187400e-02,9.40453600e-
02}])[&prob=1.00000000e+00,prob_stddev=0.00000000e+00,prob_range={1.00000000e+00,1.00000000e+00},prob(percent)="100",prob+-sd="100+-
0"]:6.806775e-02[&length_mean=6.90049940e-02,length_median=6.80677500e-02,length_95%HPD={3.83998100e-02,1.00947700e-
01}],(47[&prob=1.00000000e+00,prob_stddev=0.00000000e+00,prob_range={1.00000000e+00,1.00000000e+00},prob(percent)="100",prob+-sd="100+-
0"]:1.679289e-01[&length_mean=1.68613781e-01,length_median=1.67928900e-01,length_95%HPD={1.32555800e-01,2.06251200e-
01}],(48[&prob=1.00000000e+00,prob_stddev=0.00000000e+00,prob_range={1.00000000e+00,1.00000000e+00},prob(percent)="100",prob+-sd="100+-
0"]:1.100206e-01[&length_mean=1.10509983e-01,length_median=1.10020600e-01,length_95%HPD={8.56700600e-02,1.35386900e-
01}],49[&prob=1.00000000e+00,prob_stddev=0.00000000e+00,prob_range={1.00000000e+00,1.00000000e+00},prob(percent)="100",prob+-sd="100+-
0"]:4.014156e-02[&length_mean=4.07250911e-02,length_median=4.01415600e-02,length_95%HPD={2.42579300e-02,5.83704900e-
02}])[&prob=1.00000000e+00,prob_stddev=0.00000000e+00,prob_range={1.00000000e+00,1.00000000e+00},prob(percent)="100",prob+-sd="100+-
0"]:1.760703e-01[&length_mean=1.76601702e-01,length_median=1.76070300e-01,length_95%HPD={1.38454900e-01,2.15583000e-
01}])[&prob=1.00000000e+00,prob_stddev=0.00000000e+00,prob_range={1.00000000e+00,1.00000000e+00},prob(percent)="100",prob+-sd="100+-
0"]:1.362559e-01[&length_mean=1.37019493e-01,length_median=1.36255900e-01,length_95%HPD={9.92639900e-02,1.76308400e-
01}])[&prob=1.00000000e+00,prob_stddev=0.00000000e+00,prob_range={1.00000000e+00,1.00000000e+00},prob(percent)="100",prob+-sd="100+-
0"]:2.849516e-01[&length_mean=2.85858955e-01,length_median=2.84951600e-01,length_95%HPD={2.21893500e-01,3.49465400e-
01}],46[&prob=1.00000000e+00,prob_stddev=0.00000000e+00,prob_range={1.00000000e+00,1.00000000e+00},prob(percent)="100",prob+-sd="100+-
0"]:7.068503e-01[&length_mean=7.08325475e-01,length_median=7.06850300e-01,length_95%HPD={6.13326400e-01,8.07000800e-
01}])[&prob=9.98353355e-01,prob_stddev=3.67690624e-04,prob_range={9.98093359e-01,9.98613352e-01},prob(percent)="100",prob+-sd="100+-
0"]:8.899741e-02[&length_mean=8.98314093e-02,length_median=8.89974100e-02,length_95%HPD={4.18193400e-02,1.37166200e-
01}],(((50[&prob=1.00000000e+00,prob_stddev=0.00000000e+00,prob_range={1.00000000e+00,1.00000000e+00},prob(percent)="100",prob+-sd="100+-
0"]:4.603851e-01[&length_mean=4.61174897e-01,length_median=4.60385100e-01,length_95%HPD={3.89115500e-01,5.35469000e-
01}],(51[&prob=1.00000000e+00,prob_stddev=0.00000000e+00,prob_range={1.00000000e+00,1.00000000e+00},prob(percent)="100",prob+-sd="100+-
0"]:9.301698e-02[&length_mean=9.36482344e-02,length_median=9.30169800e-02,length_95%HPD={6.62614300e-02,1.23271400e-
01}],(52[&prob=1.00000000e+00,prob_stddev=0.00000000e+00,prob_range={1.00000000e+00,1.00000000e+00},prob(percent)="100",prob+-sd="100+-
0"]:5.712968e-02[&length_mean=5.76346288e-02,length_median=5.71296800e-02,length_95%HPD={3.94064400e-02,7.79575800e-
02}],53[&prob=1.00000000e+00,prob_stddev=0.00000000e+00,prob_range={1.00000000e+00,1.00000000e+00},prob(percent)="100",prob+-sd="100+-
0"]:1.390652e-01[&length_mean=1.39665414e-01,length_median=1.39065200e-01,length_95%HPD={1.10850800e-01,1.68718900e-
01}])[&prob=1.00000000e+00,prob_stddev=0.00000000e+00,prob_range={1.00000000e+00,1.00000000e+00},prob(percent)="100",prob+-sd="100+-
0"]:8.529612e-02[&length_mean=8.59300410e-02,length_median=8.52961200e-02,length_95%HPD={5.71390100e-02,1.15080500e-
01}])[&prob=1.00000000e+00,prob_stddev=0.00000000e+00,prob_range={1.00000000e+00,1.00000000e+00},prob(percent)="100",prob+-sd="100+-
0"]:1.814211e-01[&length_mean=1.82315964e-01,length_median=1.81421100e-01,length_95%HPD={1.28272700e-01,2.34581800e-
01}])[&prob=1.00000000e+00,prob_stddev=0.00000000e+00,prob_range={1.00000000e+00,1.00000000e+00},prob(percent)="100",prob+-sd="100+-
0"]:3.133067e-01[&length_mean=3.14348159e-01,length_median=3.13306700e-01,length_95%HPD={2.47757700e-01,3.81783700e-
01}],((54[&prob=1.00000000e+00,prob_stddev=0.00000000e+00,prob_range={1.00000000e+00,1.00000000e+00},prob(percent)="100",prob+-sd="100+-
0"]:4.890305e-01[&length_mean=4.90809772e-01,length_median=4.89030500e-01,length_95%HPD={4.16488000e-01,5.67091300e-
01}],((55[&prob=1.00000000e+00,prob_stddev=0.00000000e+00,prob_range={1.00000000e+00,1.00000000e+00},prob(percent)="100",prob+-sd="100+-
0"]:6.582237e-02[&length_mean=6.62665503e-02,length_median=6.58223700e-02,length_95%HPD={4.70691700e-02,8.54483100e-
02}],57[&prob=1.00000000e+00,prob_stddev=0.00000000e+00,prob_range={1.00000000e+00,1.00000000e+00},prob(percent)="100",prob+-sd="100+-
0"]:7.337310e-02[&length_mean=7.39696474e-02,length_median=7.33731000e-02,length_95%HPD={5.39145000e-02,9.48166600e-
02}])[&prob=1.00000000e+00,prob_stddev=0.00000000e+00,prob_range={1.00000000e+00,1.00000000e+00},prob(percent)="100",prob+-sd="100+-
0"]:1.019570e-01[&length_mean=1.02376795e-01,length_median=1.01957000e-01,length_95%HPD={7.18534200e-02,1.33679100e-

01}],56[&prob=1.00000000e+00,prob_stddev=0.00000000e+00,prob_range={1.00000000e+00,1.00000000e+00},prob(percent)="100",prob+-sd="100+-
0"]:9.551678e-02[&length_mean=9.62291496e-02,length_median=9.55167800e-02,length_95%HPD={6.65023600e-02,1.26685100e-
01}])[&prob=1.00000000e+00,prob_stddev=0.00000000e+00,prob_range={1.00000000e+00,1.00000000e+00},prob(percent)="100",prob+-sd="100+-
0"]:3.015242e-01[&length_mean=3.02189660e-01,length_median=3.01524200e-01,length_95%HPD={2.41982200e-01,3.62377100e-
01}])[&prob=1.00000000e+00,prob_stddev=0.00000000e+00,prob_range={1.00000000e+00,1.00000000e+00},prob(percent)="100",prob+-sd="100+-
0"]:1.386034e-01[&length_mean=1.39744172e-01,length_median=1.38603400e-01,length_95%HPD={9.07138500e-02,1.93255200e-
01}],((58[&prob=1.00000000e+00,prob_stddev=0.00000000e+00,prob_range={1.00000000e+00,1.00000000e+00},prob(percent)="100",prob+-sd="100+-
0"]:7.142285e-02[&length_mean=7.19825319e-02,length_median=7.14228500e-02,length_95%HPD={4.93231700e-02,9.67823400e-
02}],59[&prob=1.00000000e+00,prob_stddev=0.00000000e+00,prob_range={1.00000000e+00,1.00000000e+00},prob(percent)="100",prob+-sd="100+-
0"]:6.821061e-02[&length_mean=6.87543848e-02,length_median=6.82106100e-02,length_95%HPD={4.51136400e-02,9.24175300e-
02}])[&prob=1.00000000e+00,prob_stddev=0.00000000e+00,prob_range={1.00000000e+00,1.00000000e+00},prob(percent)="100",prob+-sd="100+-
0"]:3.161890e-01[&length_mean=3.16774903e-01,length_median=3.16189000e-01,length_95%HPD={2.57134200e-01,3.75580000e-
01}],60[&prob=1.00000000e+00,prob_stddev=0.00000000e+00,prob_range={1.00000000e+00,1.00000000e+00},prob(percent)="100",prob+-sd="100+-
0"]:4.021682e-01[&length_mean=4.03010317e-01,length_median=4.02168200e-01,length_95%HPD={3.37701100e-01,4.69854000e-
01}])[&prob=1.00000000e+00,prob_stddev=0.00000000e+00,prob_range={1.00000000e+00,1.00000000e+00},prob(percent)="100",prob+-sd="100+-
0"]:1.201029e-01[&length_mean=1.21012082e-01,length_median=1.20102900e-01,length_95%HPD={7.61456600e-02,1.66228800e-
01}])[&prob=1.00000000e+00,prob_stddev=0.00000000e+00,prob_range={1.00000000e+00,1.00000000e+00},prob(percent)="100",prob+-sd="100+-
0"]:1.765552e-01[&length_mean=1.77464404e-01,length_median=1.76555200e-01,length_95%HPD={1.25920200e-01,2.29922300e-
01}])[&prob=9.99993333e-01,prob_stddev=9.42796471e-06,prob_range={9.99986667e-01,1.00000000e+00},prob(percent)="100",prob+-sd="100+-
0"]:7.869279e-02[&length_mean=7.98023325e-02,length_median=7.86927900e-02,length_95%HPD={4.11144000e-02,1.19534500e-
01}],(((61[&prob=1.00000000e+00,prob_stddev=0.00000000e+00,prob_range={1.00000000e+00,1.00000000e+00},prob(percent)="100",prob+-sd="100+-
0"]:2.478400e-01[&length_mean=2.49682383e-01,length_median=2.47840000e-01,length_95%HPD={1.87601200e-01,3.17970700e-
01}],(62[&prob=1.00000000e+00,prob_stddev=0.00000000e+00,prob_range={1.00000000e+00,1.00000000e+00},prob(percent)="100",prob+-sd="100+-
0"]:2.420417e-01[&length_mean=2.43373891e-01,length_median=2.42041700e-01,length_95%HPD={1.94287600e-01,2.98217900e-
01}],63[&prob=1.00000000e+00,prob_stddev=0.00000000e+00,prob_range={1.00000000e+00,1.00000000e+00},prob(percent)="100",prob+-sd="100+-
0"]:4.786547e-01[&length_mean=4.79208312e-01,length_median=4.78654700e-01,length_95%HPD={4.05489700e-01,5.57816100e-
01}])[&prob=9.02354635e-01,prob_stddev=6.31673636e-04,prob_range={9.01907975e-01,9.02801296e-01},prob(percent)="90",prob+-sd="90+-
0"]:7.253972e-02[&length_mean=7.40031930e-02,length_median=7.25397200e-02,length_95%HPD={2.69635700e-02,1.24273000e-
01}])[&prob=1.00000000e+00,prob_stddev=0.00000000e+00,prob_range={1.00000000e+00,1.00000000e+00},prob(percent)="100",prob+-sd="100+-
0"]:7.054465e-01[&length_mean=7.06467324e-01,length_median=7.05446500e-01,length_95%HPD={6.01186100e-01,8.14503800e-
01}],64[&prob=1.00000000e+00,prob_stddev=0.00000000e+00,prob_range={1.00000000e+00,1.00000000e+00},prob(percent)="100",prob+-sd="100+-
0"]:4.823086e-01[&length_mean=4.83722835e-01,length_median=4.82308600e-01,length_95%HPD={4.02345600e-01,5.66902100e-
01}])[&prob=9.99813336e-01,prob_stddev=3.77118588e-05,prob_range={9.99786670e-01,9.99840002e-01},prob(percent)="100",prob+-sd="100+-
0"]:1.013804e-01[&length_mean=1.02847319e-01,length_median=1.01380400e-01,length_95%HPD={4.70466000e-02,1.60809700e-
01}],((65[&prob=1.00000000e+00,prob_stddev=0.00000000e+00,prob_range={1.00000000e+00,1.00000000e+00},prob(percent)="100",prob+-sd="100+-
0"]:5.604261e-01[&length_mean=5.61933556e-01,length_median=5.60426100e-01,length_95%HPD={4.70927100e-01,6.57790700e-
01}],66[&prob=1.00000000e+00,prob_stddev=0.00000000e+00,prob_range={1.00000000e+00,1.00000000e+00},prob(percent)="100",prob+-sd="100+-
0"]:5.825094e-01[&length_mean=5.84205107e-01,length_median=5.82509400e-01,length_95%HPD={4.94433900e-01,6.80888900e-
01}])[&prob=1.00000000e+00,prob_stddev=0.00000000e+00,prob_range={1.00000000e+00,1.00000000e+00},prob(percent)="100",prob+-sd="100+-
0"]:2.237325e-01[&length_mean=2.24681532e-01,length_median=2.23732500e-01,length_95%HPD={1.55349100e-01,2.94513400e-
01}],((((((((67[&prob=1.00000000e+00,prob_stddev=0.00000000e+00,prob_range={1.00000000e+00,1.00000000e+00},prob(percent)="100",prob+-
sd="100+-0"]:1.571336e-01[&length_mean=1.57696006e-01,length_median=1.57133600e-01,length_95%HPD={1.11380700e-01,2.03136400e-
01}],68[&prob=1.00000000e+00,prob_stddev=0.00000000e+00,prob_range={1.00000000e+00,1.00000000e+00},prob(percent)="100",prob+-sd="100+-
0"]:1.660819e-01[&length_mean=1.66554479e-01,length_median=1.66081900e-01,length_95%HPD={1.21287400e-01,2.14145000e-
01}])[&prob=1.00000000e+00,prob_stddev=0.00000000e+00,prob_range={1.00000000e+00,1.00000000e+00},prob(percent)="100",prob+-sd="100+-
0"]:6.873808e-01[&length_mean=6.88977454e-01,length_median=6.87380800e-01,length_95%HPD={5.93845800e-01,7.86458900e-
01}],(69[&prob=1.00000000e+00,prob_stddev=0.00000000e+00,prob_range={1.00000000e+00,1.00000000e+00},prob(percent)="100",prob+-sd="100+-
0"]:4.178369e-01[&length_mean=4.19006168e-01,length_median=4.17836900e-01,length_95%HPD={3.52306800e-01,4.89084900e-
01}],(70[&prob=1.00000000e+00,prob_stddev=0.00000000e+00,prob_range={1.00000000e+00,1.00000000e+00},prob(percent)="100",prob+-sd="100+-
0"]:1.950543e-01[&length_mean=1.95748838e-01,length_median=1.95054300e-01,length_95%HPD={1.53024400e-01,2.40110000e-
01}],(71[&prob=1.00000000e+00,prob_stddev=0.00000000e+00,prob_range={1.00000000e+00,1.00000000e+00},prob(percent)="100",prob+-sd="100+-
0"]:2.858238e-01[&length_mean=2.86442637e-01,length_median=2.85823800e-01,length_95%HPD={2.37446600e-01,3.34571700e-
01}],72[&prob=1.00000000e+00,prob_stddev=0.00000000e+00,prob_range={1.00000000e+00,1.00000000e+00},prob(percent)="100",prob+-sd="100+-
0"]:2.406842e-01[&length_mean=2.41501771e-01,length_median=2.40684200e-01,length_95%HPD={1.96372800e-01,2.88426100e-
01}])[&prob=1.00000000e+00,prob_stddev=0.00000000e+00,prob_range={1.00000000e+00,1.00000000e+00},prob(percent)="100",prob+-sd="100+-
0"]:8.977013e-02[&length_mean=9.05865647e-02,length_median=8.97701300e-02,length_95%HPD={5.69776700e-02,1.26712800e-
01}])[&prob=1.00000000e+00,prob_stddev=0.00000000e+00,prob_range={1.00000000e+00,1.00000000e+00},prob(percent)="100",prob+-sd="100+-
0"]:1.987446e-01[&length_mean=1.99547110e-01,length_median=1.98744600e-01,length_95%HPD={1.46249800e-01,2.51597400e-
01}])[&prob=1.00000000e+00,prob_stddev=0.00000000e+00,prob_range={1.00000000e+00,1.00000000e+00},prob(percent)="100",prob+-sd="100+-
0"]:1.082431e-01[&length_mean=1.09452984e-01,length_median=1.08243100e-01,length_95%HPD={6.55257800e-02,1.55853000e-
01}])[&prob=1.00000000e+00,prob_stddev=0.00000000e+00,prob_range={1.00000000e+00,1.00000000e+00},prob(percent)="100",prob+-sd="100+-
0"]:1.018951e-01[&length_mean=1.03120226e-01,length_median=1.01895100e-01,length_95%HPD={5.50897600e-02,1.51406700e-
01}],(73[&prob=1.00000000e+00,prob_stddev=0.00000000e+00,prob_range={1.00000000e+00,1.00000000e+00},prob(percent)="100",prob+-sd="100+-
0"]:2.556016e-01[&length_mean=2.56526012e-01,length_median=2.55601600e-01,length_95%HPD={2.04071700e-01,3.12903400e-
01}],74[&prob=1.00000000e+00,prob_stddev=0.00000000e+00,prob_range={1.00000000e+00,1.00000000e+00},prob(percent)="100",prob+-sd="100+-

0"]:2.087500e-01[&length_mean=2.09764160e-01,length_median=2.08750000e-01,length_95%HPD={1.61223500e-01,2.59469600e-01}])[&prob=1.00000000e+00,prob_stddev=0.00000000e+00,prob_range={1.00000000e+00,1.00000000e+00},prob(percent)="100",prob+-sd="100+-0"]:3.564079e-01[&length_mean=3.57562458e-01,length_median=3.56407900e-01,length_95%HPD={2.91718500e-01,4.28002800e-01}])[&prob=1.00000000e+00,prob_stddev=0.00000000e+00,prob_range={1.00000000e+00,1.00000000e+00},prob(percent)="100",prob+-sd="100+-0"]:1.212067e-01[&length_mean=1.22377171e-01,length_median=1.21206700e-01,length_95%HPD={7.45083400e-02,1.72001000e-01}],(75[&prob=1.00000000e+00,prob_stddev=0.00000000e+00,prob_range={1.00000000e+00,1.00000000e+00},prob(percent)="100",prob+-sd="100+-0"]:1.806297e-01[&length_mean=1.81404410e-01,length_median=1.80629700e-01,length_95%HPD={1.38918000e-01,2.26278900e-01}],76[&prob=1.00000000e+00,prob_stddev=0.00000000e+00,prob_range={1.00000000e+00,1.00000000e+00},prob(percent)="100",prob+-sd="100+-0"]:2.997855e-01[&length_mean=3.00534917e-01,length_median=2.99785500e-01,length_95%HPD={2.47290500e-01,3.53345500e-01}])[&prob=1.00000000e+00,prob_stddev=0.00000000e+00,prob_range={1.00000000e+00,1.00000000e+00},prob(percent)="100",prob+-sd="100+-0"]:3.020860e-01[&length_mean=3.02936417e-01,length_median=3.02086000e-01,length_95%HPD={2.41741400e-01,3.62913200e-01}])[&prob=1.00000000e+00,prob_stddev=0.00000000e+00,prob_range={1.00000000e+00,1.00000000e+00},prob(percent)="100",prob+-sd="100+-0"]:7.418306e-02[&length_mean=7.51117280e-02,length_median=7.41830600e-02,length_95%HPD={4.05965400e-02,1.12673000e-01}],((((77[&prob=1.00000000e+00,prob_stddev=0.00000000e+00,prob_range={1.00000000e+00,1.00000000e+00},prob(percent)="100",prob+-sd="100+-0"]:4.728676e-01[&length_mean=4.73707212e-01,length_median=4.72867600e-01,length_95%HPD={4.02196700e-01,5.49742800e-01}],81[&prob=1.00000000e+00,prob_stddev=0.00000000e+00,prob_range={1.00000000e+00,1.00000000e+00},prob(percent)="100",prob+-sd="100+-0"]:4.441254e-01[&length_mean=4.45604876e-01,length_median=4.44125400e-01,length_95%HPD={3.73042800e-01,5.17647000e-01}])[&prob=9.99880002e-01,prob_stddev=1.31991506e-04,prob_range={9.99786670e-01,9.99973334e-01},prob(percent)="100",prob+-sd="100+-0"]:7.585303e-02[&length_mean=7.69338257e-02,length_median=7.58530300e-02,length_95%HPD={3.41234600e-02,1.20085400e-01}],82[&prob=1.00000000e+00,prob_stddev=0.00000000e+00,prob_range={1.00000000e+00,1.00000000e+00},prob(percent)="100",prob+-sd="100+-0"]:3.389164e-01[&length_mean=3.39995561e-01,length_median=3.38916400e-01,length_95%HPD={2.80155600e-01,3.99472100e-01}])[&prob=1.00000000e+00,prob_stddev=0.00000000e+00,prob_range={1.00000000e+00,1.00000000e+00},prob(percent)="100",prob+-sd="100+-0"]:1.856998e-01[&length_mean=1.86772173e-01,length_median=1.85699800e-01,length_95%HPD={1.36826800e-01,2.39194700e-01}],((83[&prob=1.00000000e+00,prob_stddev=0.00000000e+00,prob_range={1.00000000e+00,1.00000000e+00},prob(percent)="100",prob+-sd="100+-0"]:4.014126e-01[&length_mean=4.02056794e-01,length_median=4.01412600e-01,length_95%HPD={3.34183900e-01,4.70820500e-01}],84[&prob=1.00000000e+00,prob_stddev=0.00000000e+00,prob_range={1.00000000e+00,1.00000000e+00},prob(percent)="100",prob+-sd="100+-0"]:3.465520e-01[&length_mean=3.47228596e-01,length_median=3.46552000e-01,length_95%HPD={2.86924000e-01,4.08061700e-01}])[&prob=9.54527273e-01,prob_stddev=2.92266906e-04,prob_range={9.54320609e-01,9.54733937e-01},prob(percent)="95",prob+-sd="95+-0"]:6.304308e-02[&length_mean=6.39817720e-02,length_median=6.30430800e-02,length_95%HPD={2.63957400e-02,1.03303900e-01}],85[&prob=1.00000000e+00,prob_stddev=0.00000000e+00,prob_range={1.00000000e+00,1.00000000e+00},prob(percent)="100",prob+-sd="100+-0"]:4.840001e-01[&length_mean=4.84809794e-01,length_median=4.84000100e-01,length_95%HPD={4.11564400e-01,5.58041900e-01}])[&prob=5.52552633e-01,prob_stddev=1.81016922e-03,prob_range={5.51272650e-01,5.53832616e-01},prob(percent)="55",prob+-sd="55+-0"]:4.919761e-02[&length_mean=5.00969812e-02,length_median=4.91976100e-02,length_95%HPD={1.84116200e-02,8.44890500e-02}])[&prob=9.37374168e-01,prob_stddev=7.33495654e-03,prob_range={9.32187571e-01,9.42560766e-01},prob(percent)="94",prob+-sd="94+-1"]:5.651478e-02[&length_mean=5.76378708e-02,length_median=5.65147800e-02,length_95%HPD={2.54550800e-02,9.31945400e-02}],(((78[&prob=1.00000000e+00,prob_stddev=0.00000000e+00,prob_range={1.00000000e+00,1.00000000e+00},prob(percent)="100",prob+-sd="100+-0"]:4.116523e-01[&length_mean=4.12289250e-01,length_median=4.11652300e-01,length_95%HPD={3.44361000e-01,4.83421600e-01}],80[&prob=1.00000000e+00,prob_stddev=0.00000000e+00,prob_range={1.00000000e+00,1.00000000e+00},prob(percent)="100",prob+-sd="100+-0"]:3.499468e-01[&length_mean=3.51109870e-01,length_median=3.49946800e-01,length_95%HPD={2.89374000e-01,4.15904800e-01}])[&prob=9.99673338e-01,prob_stddev=1.22563541e-04,prob_range={9.99586672e-01,9.99760003e-01},prob(percent)="100",prob+-sd="100+-0"]:8.958054e-02[&length_mean=9.05114480e-02,length_median=8.95805400e-02,length_95%HPD={4.45563800e-02,1.40755300e-01}],79[&prob=1.00000000e+00,prob_stddev=0.00000000e+00,prob_range={1.00000000e+00,1.00000000e+00},prob(percent)="100",prob+-sd="100+-0"]:6.999343e-01[&length_mean=7.01143019e-01,length_median=6.99934300e-01,length_95%HPD={6.06526000e-01,7.99064600e-01}])[&prob=1.00000000e+00,prob_stddev=0.00000000e+00,prob_range={1.00000000e+00,1.00000000e+00},prob(percent)="100",prob+-sd="100+-0"]:2.384513e-01[&length_mean=2.39853806e-01,length_median=2.38451300e-01,length_95%HPD={1.80796100e-01,3.05710800e-01}],((((86[&prob=1.00000000e+00,prob_stddev=0.00000000e+00,prob_range={1.00000000e+00,1.00000000e+00},prob(percent)="100",prob+-sd="100+-0"]:6.269271e-02[&length_mean=6.32264757e-02,length_median=6.26927100e-02,length_95%HPD={4.13713600e-02,8.62195400e-02}],87[&prob=1.00000000e+00,prob_stddev=0.00000000e+00,prob_range={1.00000000e+00,1.00000000e+00},prob(percent)="100",prob+-sd="100+-0"]:4.494923e-02[&length_mean=4.54608218e-02,length_median=4.49492300e-02,length_95%HPD={2.52069200e-02,6.56362000e-02}])[&prob=1.00000000e+00,prob_stddev=0.00000000e+00,prob_range={1.00000000e+00,1.00000000e+00},prob(percent)="100",prob+-sd="100+-0"]:4.027115e-01[&length_mean=4.03492026e-01,length_median=4.02711500e-01,length_95%HPD={3.36464800e-01,4.69651300e-01}],88[&prob=1.00000000e+00,prob_stddev=0.00000000e+00,prob_range={1.00000000e+00,1.00000000e+00},prob(percent)="100",prob+-sd="100+-0"]:3.545106e-01[&length_mean=3.55343756e-01,length_median=3.54510600e-01,length_95%HPD={2.92283200e-01,4.18986300e-01}])[&prob=1.00000000e+00,prob_stddev=0.00000000e+00,prob_range={1.00000000e+00,1.00000000e+00},prob(percent)="100",prob+-sd="100+-0"]:1.427534e-01[&length_mean=1.43787098e-01,length_median=1.42753400e-01,length_95%HPD={9.65538500e-02,1.93297600e-01}],(89[&prob=1.00000000e+00,prob_stddev=0.00000000e+00,prob_range={1.00000000e+00,1.00000000e+00},prob(percent)="100",prob+-sd="100+-0"]:1.481506e-01[&length_mean=1.48722089e-01,length_median=1.48150600e-01,length_95%HPD={1.11668700e-01,1.86118300e-01}],90[&prob=1.00000000e+00,prob_stddev=0.00000000e+00,prob_range={1.00000000e+00,1.00000000e+00},prob(percent)="100",prob+-sd="100+-0"]:2.084164e-01[&length_mean=2.09055125e-01,length_median=2.08416400e-01,length_95%HPD={1.65580400e-01,2.50716400e-01}])[&prob=1.00000000e+00,prob_stddev=0.00000000e+00,prob_range={1.00000000e+00,1.00000000e+00},prob(percent)="100",prob+-sd="100+-0"]:1.037862e-01[&length_mean=1.04359864e-01,length_median=1.03786200e-01,length_95%HPD={6.58943700e-02,1.44531100e-01}])[&prob=1.00000000e+00,prob_stddev=0.00000000e+00,prob_range={1.00000000e+00,1.00000000e+00},prob(percent)="100",prob+-sd="100+-0"]:1.820967e-01[&length_mean=1.83122714e-01,length_median=1.82096700e-01,length_95%HPD={1.27998500e-01,2.39809300e-01}],91[&prob=1.00000000e+00,prob_stddev=0.00000000e+00,prob_range={1.00000000e+00,1.00000000e+00},prob(percent)="100",prob+-sd="100+-0"]:4.155823e-01[&length_mean=4.16771687e-01,length_median=4.15582300e-01,length_95%HPD={3.46555800e-01,4.86596300e-

01}])[&prob=1.00000000e+00,prob_stddev=0.00000000e+00,prob_range={1.00000000e+00,1.00000000e+00},prob(percent)="100",prob+-sd="100+-
0"]:2.552067e-01[&length_mean=2.56443968e-01,length_median=2.55206700e-01,length_95%HPD={1.93895800e-01,3.20024400e-
01}])[&prob=9.50560659e-01,prob_stddev=5.10995687e-03,prob_range={9.46947374e-01,9.54173944e-01},prob(percent)="95",prob+-sd="95+-
1"]:4.748185e-02[&length_mean=4.85328119e-02,length_median=4.74818500e-02,length_95%HPD={1.73443400e-02,8.36169800e-
02}])[&prob=1.00000000e+00,prob_stddev=0.00000000e+00,prob_range={1.00000000e+00,1.00000000e+00},prob(percent)="100",prob+-sd="100+-
0"]:2.011318e-01[&length_mean=2.02302796e-01,length_median=2.01131800e-01,length_95%HPD={1.51260200e-01,2.52111500e-
01}])[&prob=9.99913334e-01,prob_stddev=8.48516824e-05,prob_range={9.99853335e-01,9.99973334e-01},prob(percent)="100",prob+-sd="100+-
0"]:5.842979e-02[&length_mean=5.96986600e-02,length_median=5.84297900e-02,length_95%HPD={2.41522200e-02,9.68241200e-
02}],(92[&prob=1.00000000e+00,prob_stddev=0.00000000e+00,prob_range={1.00000000e+00,1.00000000e+00},prob(percent)="100",prob+-sd="100+-
0"]:4.780464e-01[&length_mean=4.79218185e-01,length_median=4.78046400e-01,length_95%HPD={3.99135100e-01,5.60140200e-
01}],(93[&prob=1.00000000e+00,prob_stddev=0.00000000e+00,prob_range={1.00000000e+00,1.00000000e+00},prob(percent)="100",prob+-sd="100+-
0"]:1.332502e-01[&length_mean=1.34039459e-01,length_median=1.33250200e-01,length_95%HPD={9.85148600e-02,1.70613900e-
01}],94[&prob=1.00000000e+00,prob_stddev=0.00000000e+00,prob_range={1.00000000e+00,1.00000000e+00},prob(percent)="100",prob+-sd="100+-
0"]:1.512068e-01[&length_mean=1.51832305e-01,length_median=1.51206800e-01,length_95%HPD={1.14205600e-01,1.90964200e-
01}])[&prob=1.00000000e+00,prob_stddev=0.00000000e+00,prob_range={1.00000000e+00,1.00000000e+00},prob(percent)="100",prob+-sd="100+-
0"]:4.042074e-01[&length_mean=4.04872800e-01,length_median=4.04207400e-01,length_95%HPD={3.31320500e-01,4.81613500e-
01}])[&prob=1.00000000e+00,prob_stddev=0.00000000e+00,prob_range={1.00000000e+00,1.00000000e+00},prob(percent)="100",prob+-sd="100+-
0"]:2.293658e-01[&length_mean=2.30349940e-01,length_median=2.29365800e-01,length_95%HPD={1.67226700e-01,2.92528300e-
01}])[&prob=1.00000000e+00,prob_stddev=0.00000000e+00,prob_range={1.00000000e+00,1.00000000e+00},prob(percent)="100",prob+-sd="100+-
0"]:1.742566e-01[&length_mean=1.75258064e-01,length_median=1.74256600e-01,length_95%HPD={1.28462300e-01,2.24219600e-
01}],((((95[&prob=1.00000000e+00,prob_stddev=0.00000000e+00,prob_range={1.00000000e+00,1.00000000e+00},prob(percent)="100",prob+-sd="100+-
0"]:1.211953e-01[&length_mean=1.21780578e-01,length_median=1.21195300e-01,length_95%HPD={9.15110300e-02,1.53767400e-
01}],96[&prob=1.00000000e+00,prob_stddev=0.00000000e+00,prob_range={1.00000000e+00,1.00000000e+00},prob(percent)="100",prob+-sd="100+-
0"]:1.507245e-01[&length_mean=1.51274555e-01,length_median=1.50724500e-01,length_95%HPD={1.17332400e-01,1.84768400e-
01}])[&prob=1.00000000e+00,prob_stddev=0.00000000e+00,prob_range={1.00000000e+00,1.00000000e+00},prob(percent)="100",prob+-sd="100+-
0"]:1.625617e-01[&length_mean=1.63382329e-01,length_median=1.62561700e-01,length_95%HPD={1.18164600e-01,2.12880900e-
01}],(97[&prob=1.00000000e+00,prob_stddev=0.00000000e+00,prob_range={1.00000000e+00,1.00000000e+00},prob(percent)="100",prob+-sd="100+-
0"]:2.869114e-01[&length_mean=2.87550226e-01,length_median=2.86911400e-01,length_95%HPD={2.36675500e-01,3.39407600e-
01}],98[&prob=1.00000000e+00,prob_stddev=0.00000000e+00,prob_range={1.00000000e+00,1.00000000e+00},prob(percent)="100",prob+-sd="100+-
0"]:2.777710e-01[&length_mean=2.78246587e-01,length_median=2.77771000e-01,length_95%HPD={2.28237800e-01,3.29048700e-
01}])[&prob=1.00000000e+00,prob_stddev=0.00000000e+00,prob_range={1.00000000e+00,1.00000000e+00},prob(percent)="100",prob+-sd="100+-
0"]:1.333420e-01[&length_mean=1.34015688e-01,length_median=1.33342000e-01,length_95%HPD={8.73829300e-02,1.81595500e-
01}])[&prob=1.00000000e+00,prob_stddev=0.00000000e+00,prob_range={1.00000000e+00,1.00000000e+00},prob(percent)="100",prob+-sd="100+-
0"]:4.567414e-01[&length_mean=4.57635153e-01,length_median=4.56741400e-01,length_95%HPD={3.81282600e-01,5.33496300e-
01}],(99[&prob=1.00000000e+00,prob_stddev=0.00000000e+00,prob_range={1.00000000e+00,1.00000000e+00},prob(percent)="100",prob+-sd="100+-
0"]:3.493205e-01[&length_mean=3.50190528e-01,length_median=3.49320500e-01,length_95%HPD={2.90001100e-01,4.10835900e-
01}],100[&prob=1.00000000e+00,prob_stddev=0.00000000e+00,prob_range={1.00000000e+00,1.00000000e+00},prob(percent)="100",prob+-sd="100+-
0"]:2.779588e-01[&length_mean=2.78369523e-01,length_median=2.77958800e-01,length_95%HPD={2.25399300e-01,3.34321400e-
01}])[&prob=1.00000000e+00,prob_stddev=0.00000000e+00,prob_range={1.00000000e+00,1.00000000e+00},prob(percent)="100",prob+-sd="100+-
0"]:1.146879e-01[&length_mean=1.15627080e-01,length_median=1.14687900e-01,length_95%HPD={6.79264500e-02,1.65491300e-
01}])[&prob=1.00000000e+00,prob_stddev=0.00000000e+00,prob_range={1.00000000e+00,1.00000000e+00},prob(percent)="100",prob+-sd="100+-
0"]:1.634067e-01[&length_mean=1.64309779e-01,length_median=1.63406700e-01,length_95%HPD={1.12127500e-01,2.16052600e-
01}],((((101[&prob=1.00000000e+00,prob_stddev=0.00000000e+00,prob_range={1.00000000e+00,1.00000000e+00},prob(percent)="100",prob+-sd="100+-
0"]:1.972243e-01[&length_mean=1.97946447e-01,length_median=1.97224300e-01,length_95%HPD={1.53026600e-01,2.44569600e-
01}],(102[&prob=1.00000000e+00,prob_stddev=0.00000000e+00,prob_range={1.00000000e+00,1.00000000e+00},prob(percent)="100",prob+-sd="100+-
0"]:2.466801e-01[&length_mean=2.47487350e-01,length_median=2.46680100e-01,length_95%HPD={2.02813800e-01,2.95646100e-
01}],(103[&prob=1.00000000e+00,prob_stddev=0.00000000e+00,prob_range={1.00000000e+00,1.00000000e+00},prob(percent)="100",prob+-sd="100+-
0"]:1.691706e-01[&length_mean=1.69780729e-01,length_median=1.69170600e-01,length_95%HPD={1.36624200e-01,2.04344600e-
01}],104[&prob=1.00000000e+00,prob_stddev=0.00000000e+00,prob_range={1.00000000e+00,1.00000000e+00},prob(percent)="100",prob+-sd="100+-
0"]:1.379430e-01[&length_mean=1.38280860e-01,length_median=1.37943000e-01,length_95%HPD={1.07192000e-01,1.70878200e-
01}])[&prob=1.00000000e+00,prob_stddev=0.00000000e+00,prob_range={1.00000000e+00,1.00000000e+00},prob(percent)="100",prob+-sd="100+-
0"]:1.630045e-01[&length_mean=1.63815370e-01,length_median=1.63004500e-01,length_95%HPD={1.24474200e-01,2.04219800e-
01}])[&prob=1.00000000e+00,prob_stddev=0.00000000e+00,prob_range={1.00000000e+00,1.00000000e+00},prob(percent)="100",prob+-sd="100+-
0"]:2.771193e-01[&length_mean=2.77701587e-01,length_median=2.77119300e-01,length_95%HPD={2.24185600e-01,3.33737200e-
01}])[&prob=1.00000000e+00,prob_stddev=0.00000000e+00,prob_range={1.00000000e+00,1.00000000e+00},prob(percent)="100",prob+-sd="100+-
0"]:2.155770e-01[&length_mean=2.16437426e-01,length_median=2.15577000e-01,length_95%HPD={1.64701000e-01,2.67759300e-
01}],105[&prob=1.00000000e+00,prob_stddev=0.00000000e+00,prob_range={1.00000000e+00,1.00000000e+00},prob(percent)="100",prob+-sd="100+-
0"]:3.036546e-01[&length_mean=3.04517640e-01,length_median=3.03654600e-01,length_95%HPD={2.50133000e-01,3.59310100e-
01}])[&prob=9.62240503e-01,prob_stddev=1.73474551e-03,prob_range={9.61013853e-01,9.63467154e-01},prob(percent)="96",prob+-sd="96+-
0"]:6.993096e-02[&length_mean=7.06624435e-02,length_median=6.99309600e-02,length_95%HPD={3.18033400e-02,1.09682800e-
01}],(107[&prob=1.00000000e+00,prob_stddev=0.00000000e+00,prob_range={1.00000000e+00,1.00000000e+00},prob(percent)="100",prob+-sd="100+-
0"]:3.990705e-01[&length_mean=4.00221195e-01,length_median=3.99070500e-01,length_95%HPD={3.35736100e-01,4.68301000e-
01}],(108[&prob=1.00000000e+00,prob_stddev=0.00000000e+00,prob_range={1.00000000e+00,1.00000000e+00},prob(percent)="100",prob+-sd="100+-
0"]:1.184642e-01[&length_mean=1.18881074e-01,length_median=1.18464200e-01,length_95%HPD={8.57850100e-02,1.53763600e-
01}],(109[&prob=1.00000000e+00,prob_stddev=0.00000000e+00,prob_range={1.00000000e+00,1.00000000e+00},prob(percent)="100",prob+-sd="100+-

0"]:5.912365e-02[&length_mean=5.94353950e-02,length_median=5.91236500e-02,length_95%HPD={4.20889600e-02,7.74074500e-
02}],110[&prob=1.00000000e+00,prob_stddev=0.00000000e+00,prob_range={1.00000000e+00,1.00000000e+00},prob(percent)="100",prob+-sd="100+-
0"]:4.430033e-02[&length_mean=4.48127986e-02,length_median=4.43003300e-02,length_95%HPD={2.95686400e-02,6.11682600e-
02}])[&prob=1.00000000e+00,prob_stddev=0.00000000e+00,prob_range={1.00000000e+00,1.00000000e+00},prob(percent)="100",prob+-sd="100+-
0"]:6.857458e-02[&length_mean=6.93943894e-02,length_median=6.85745800e-02,length_95%HPD={4.15162300e-02,9.82733700e-
02}])[&prob=1.00000000e+00,prob_stddev=0.00000000e+00,prob_range={1.00000000e+00,1.00000000e+00},prob(percent)="100",prob+-sd="100+-
0"]:2.888365e-01[&length_mean=2.89848062e-01,length_median=2.88836500e-01,length_95%HPD={2.30593300e-01,3.51998600e-
01}])[&prob=1.00000000e+00,prob_stddev=0.00000000e+00,prob_range={1.00000000e+00,1.00000000e+00},prob(percent)="100",prob+-sd="100+-
0"]:2.600954e-01[&length_mean=2.61191810e-01,length_median=2.60095400e-01,length_95%HPD={2.00181000e-01,3.24549300e-
01}])[&prob=8.26762310e-01,prob_stddev=8.93771054e-03,prob_range={8.20442394e-01,8.33082226e-01},prob(percent)="83",prob+-sd="83+-
1"]:5.395160e-02[&length_mean=5.44703305e-02,length_median=5.39516000e-02,length_95%HPD={7.08269800e-03,1.00615900e-
01}],106[&prob=1.00000000e+00,prob_stddev=0.00000000e+00,prob_range={1.00000000e+00,1.00000000e+00},prob(percent)="100",prob+-sd="100+-
0"]:8.529927e-01[&length_mean=8.54109700e-01,length_median=8.52992700e-01,length_95%HPD={7.44286900e-01,9.66560100e-
01}])[&prob=6.62204504e-01,prob_stddev=1.94216073e-03,prob_range={6.60831189e-01,6.63577819e-01},prob(percent)="66",prob+-sd="66+-
0"]:4.636721e-02[&length_mean=4.73281250e-02,length_median=4.63672100e-02,length_95%HPD={2.39523000e-03,8.73125900e-
02}])[&prob=1.00000000e+00,prob_stddev=0.00000000e+00,prob_range={1.00000000e+00,1.00000000e+00},prob(percent)="100",prob+-sd="100+-
0"]:1.182329e-01[&length_mean=1.18653824e-01,length_median=1.18232900e-01,length_95%HPD={7.26356600e-02,1.65459100e-
01}])[&prob=1.00000000e+00,prob_stddev=0.00000000e+00,prob_range={1.00000000e+00,1.00000000e+00},prob(percent)="100",prob+-sd="100+-
0"]:1.013831e-01[&length_mean=1.02347820e-01,length_median=1.01383100e-01,length_95%HPD={6.16519900e-02,1.42400600e-
01}],((((((111[&prob=1.00000000e+00,prob_stddev=0.00000000e+00,prob_range={1.00000000e+00,1.00000000e+00},prob(percent)="100",prob+-
sd="100+-0"]:9.722979e-02[&length_mean=9.78534282e-02,length_median=9.72297900e-02,length_95%HPD={7.23517900e-02,1.24142300e-
01}],112[&prob=1.00000000e+00,prob_stddev=0.00000000e+00,prob_range={1.00000000e+00,1.00000000e+00},prob(percent)="100",prob+-sd="100+-
0"]:1.113956e-01[&length_mean=1.11835360e-01,length_median=1.11395600e-01,length_95%HPD={8.46127600e-02,1.38566400e-
01}])[&prob=1.00000000e+00,prob_stddev=0.00000000e+00,prob_range={1.00000000e+00,1.00000000e+00},prob(percent)="100",prob+-sd="100+-
0"]:1.994637e-01[&length_mean=2.00016775e-01,length_median=1.99463700e-01,length_95%HPD={1.61712500e-01,2.40975500e-
01}],(113[&prob=1.00000000e+00,prob_stddev=0.00000000e+00,prob_range={1.00000000e+00,1.00000000e+00},prob(percent)="100",prob+-sd="100+-
0"]:1.953750e-01[&length_mean=1.96067606e-01,length_median=1.95375000e-01,length_95%HPD={1.59778400e-01,2.33502000e-
01}],114[&prob=1.00000000e+00,prob_stddev=0.00000000e+00,prob_range={1.00000000e+00,1.00000000e+00},prob(percent)="100",prob+-sd="100+-
0"]:1.696190e-01[&length_mean=1.69988684e-01,length_median=1.69619000e-01,length_95%HPD={1.35339300e-01,2.05130700e-
01}])[&prob=1.00000000e+00,prob_stddev=0.00000000e+00,prob_range={1.00000000e+00,1.00000000e+00},prob(percent)="100",prob+-sd="100+-
0"]:6.649322e-02[&length_mean=6.71537618e-02,length_median=6.64932200e-02,length_95%HPD={4.08375200e-02,9.54533100e-
02}])[&prob=1.00000000e+00,prob_stddev=0.00000000e+00,prob_range={1.00000000e+00,1.00000000e+00},prob(percent)="100",prob+-sd="100+-
0"]:1.010381e-01[&length_mean=1.01672694e-01,length_median=1.01038100e-01,length_95%HPD={6.58517900e-02,1.37629700e-
01}],115[&prob=1.00000000e+00,prob_stddev=0.00000000e+00,prob_range={1.00000000e+00,1.00000000e+00},prob(percent)="100",prob+-sd="100+-
0"]:3.133422e-01[&length_mean=3.14100939e-01,length_median=3.13342200e-01,length_95%HPD={2.62697200e-01,3.65844300e-
01}])[&prob=1.00000000e+00,prob_stddev=0.00000000e+00,prob_range={1.00000000e+00,1.00000000e+00},prob(percent)="100",prob+-sd="100+-
0"]:1.067203e-01[&length_mean=1.07763865e-01,length_median=1.06720300e-01,length_95%HPD={6.99162600e-02,1.46810600e-
01}],116[&prob=1.00000000e+00,prob_stddev=0.00000000e+00,prob_range={1.00000000e+00,1.00000000e+00},prob(percent)="100",prob+-sd="100+-
0"]:3.250179e-01[&length_mean=3.25866498e-01,length_median=3.25017900e-01,length_95%HPD={2.72649400e-01,3.80367800e-
01}])[&prob=1.00000000e+00,prob_stddev=0.00000000e+00,prob_range={1.00000000e+00,1.00000000e+00},prob(percent)="100",prob+-sd="100+-
0"]:7.840500e-02[&length_mean=7.92085780e-02,length_median=7.84050000e-02,length_95%HPD={4.20986800e-02,1.18157800e-
01}],((117[&prob=1.00000000e+00,prob_stddev=0.00000000e+00,prob_range={1.00000000e+00,1.00000000e+00},prob(percent)="100",prob+-sd="100+-
0"]:3.720183e-01[&length_mean=3.72914615e-01,length_median=3.72018300e-01,length_95%HPD={3.07232900e-01,4.41072200e-
01}],118[&prob=1.00000000e+00,prob_stddev=0.00000000e+00,prob_range={1.00000000e+00,1.00000000e+00},prob(percent)="100",prob+-sd="100+-
0"]:4.209047e-01[&length_mean=4.22436222e-01,length_median=4.20904700e-01,length_95%HPD={3.53855900e-01,4.97262400e-
01}])[&prob=1.00000000e+00,prob_stddev=0.00000000e+00,prob_range={1.00000000e+00,1.00000000e+00},prob(percent)="100",prob+-sd="100+-
0"]:1.700442e-01[&length_mean=1.71215217e-01,length_median=1.70044200e-01,length_95%HPD={1.17324900e-01,2.25554800e-
01}],119[&prob=1.00000000e+00,prob_stddev=0.00000000e+00,prob_range={1.00000000e+00,1.00000000e+00},prob(percent)="100",prob+-sd="100+-
0"]:3.025523e-01[&length_mean=3.03402205e-01,length_median=3.02552300e-01,length_95%HPD={2.46476000e-01,3.63499700e-
01}])[&prob=1.00000000e+00,prob_stddev=0.00000000e+00,prob_range={1.00000000e+00,1.00000000e+00},prob(percent)="100",prob+-sd="100+-
0"]:2.180380e-01[&length_mean=2.18813848e-01,length_median=2.18038000e-01,length_95%HPD={1.62108400e-01,2.76582400e-
01}])[&prob=1.00000000e+00,prob_stddev=0.00000000e+00,prob_range={1.00000000e+00,1.00000000e+00},prob(percent)="100",prob+-sd="100+-
0"]:1.262181e-01[&length_mean=1.27389827e-01,length_median=1.26218100e-01,length_95%HPD={7.98251400e-02,1.77438900e-
01}],120[&prob=1.00000000e+00,prob_stddev=0.00000000e+00,prob_range={1.00000000e+00,1.00000000e+00},prob(percent)="100",prob+-sd="100+-
0"]:2.885026e-01[&length_mean=2.89343054e-01,length_median=2.88502600e-01,length_95%HPD={2.29524400e-01,3.49390800e-
01}])[&prob=1.00000000e+00,prob_stddev=0.00000000e+00,prob_range={1.00000000e+00,1.00000000e+00},prob(percent)="100",prob+-sd="100+-
0"]:6.218279e-01[&length_mean=6.22847986e-01,length_median=6.21827900e-01,length_95%HPD={5.28839200e-01,7.17195800e-
01}])[&prob=9.99993333e-01,prob_stddev=9.42796471e-06,prob_range={9.99986667e-01,1.00000000e+00},prob(percent)="100",prob+-sd="100+-
0"]:7.201020e-02[&length_mean=7.31344966e-02,length_median=7.20102000e-02,length_95%HPD={3.58983700e-02,1.12208500e-
01}])[&prob=9.99946667e-01,prob_stddev=1.88559294e-05,prob_range={9.99933334e-01,9.99960001e-01},prob(percent)="100",prob+-sd="100+-
0"]:6.109504e-02[&length_mean=6.20193777e-02,length_median=6.10950400e-02,length_95%HPD={2.77281900e-02,9.86314200e-
02}])[&prob=1.00000000e+00,prob_stddev=0.00000000e+00,prob_range={1.00000000e+00,1.00000000e+00},prob(percent)="100",prob+-sd="100+-
0"]:6.855702e-02[&length_mean=6.95993954e-02,length_median=6.85570200e-02,length_95%HPD={3.52178300e-02,1.04832500e-
01}])[&prob=1.00000000e+00,prob_stddev=0.00000000e+00,prob_range={1.00000000e+00,1.00000000e+00},prob(percent)="100",prob+-sd="100+-
0"]:1.067070e-01[&length_mean=1.07696473e-01,length_median=1.06707000e-01,length_95%HPD={7.23364100e-02,1.46485400e-

01}])[&prob=9.30207597e-01,prob_stddev=1.60275400e-04,prob_range={9.30094265e-01,9.30320929e-01},prob(percent)="93",prob+-sd="93+-
0"]:3.839225e-02[&length_mean=3.95015853e-02,length_median=3.83922500e-02,length_95%HPD={1.35171700e-02,6.72000900e-
02}])[&prob=9.35880855e-01,prob_stddev=2.56440640e-03,prob_range={9.34067546e-01,9.37694164e-01},prob(percent)="94",prob+-sd="94+-
0"]:4.058067e-02[&length_mean=4.17375882e-02,length_median=4.05806700e-02,length_95%HPD={1.27979900e-02,7.06596000e-
02}])[&prob=1.00000000e+00,prob_stddev=0.00000000e+00,prob_range={1.00000000e+00,1.00000000e+00},prob(percent)="100",prob+-sd="100+-
0"]:1.085652e-01[&length_mean=1.09464790e-01,length_median=1.08565200e-01,length_95%HPD={6.93766000e-02,1.50300900e-
01}])[&prob=1.00000000e+00,prob_stddev=0.00000000e+00,prob_range={1.00000000e+00,1.00000000e+00},prob(percent)="100",prob+-sd="100+-
0"]:7.041992e-02[&length_mean=7.11910095e-02,length_median=7.04199200e-02,length_95%HPD={3.76289500e-02,1.04229500e-
01}])[&prob=1.00000000e+00,prob_stddev=0.00000000e+00,prob_range={1.00000000e+00,1.00000000e+00},prob(percent)="100",prob+-sd="100+-
0"]:8.535294e-02[&length_mean=8.62132944e-02,length_median=8.53529400e-02,length_95%HPD={5.08787900e-02,1.22416000e-
01}])[&prob=1.00000000e+00,prob_stddev=0.00000000e+00,prob_range={1.00000000e+00,1.00000000e+00},prob(percent)="100",prob+-sd="100+-
0"]:1.517918e-01[&length_mean=1.52528915e-01,length_median=1.51791800e-01,length_95%HPD={1.04196400e-01,1.99601500e-
01}])[&prob=1.00000000e+00,prob_stddev=0.00000000e+00,prob_range={1.00000000e+00,1.00000000e+00},prob(percent)="100",prob+-sd="100+-
0"]:3.669543e-01[&length_mean=3.67533447e-01,length_median=3.66954300e-01,length_95%HPD={3.04288700e-01,4.34017300e-01}]);
end;
